# Diphosphinoboranes as Intramolecular Frustrated Lewis Pairs: P–B–P Bond Systems for the Activation of Dihydrogen, Carbon Dioxide, and Phenyl Isocyanate

**DOI:** 10.1021/acs.inorgchem.0c03563

**Published:** 2021-03-04

**Authors:** Natalia Szynkiewicz, Anna Ordyszewska, Jarosław Chojnacki, Rafał Grubba

**Affiliations:** Department of Inorganic Chemistry, Faculty of Chemistry, Gdańsk University of Technology, 11/12 Gabriela Narutowicza Str. 80-233 Gdańsk, Poland

## Abstract

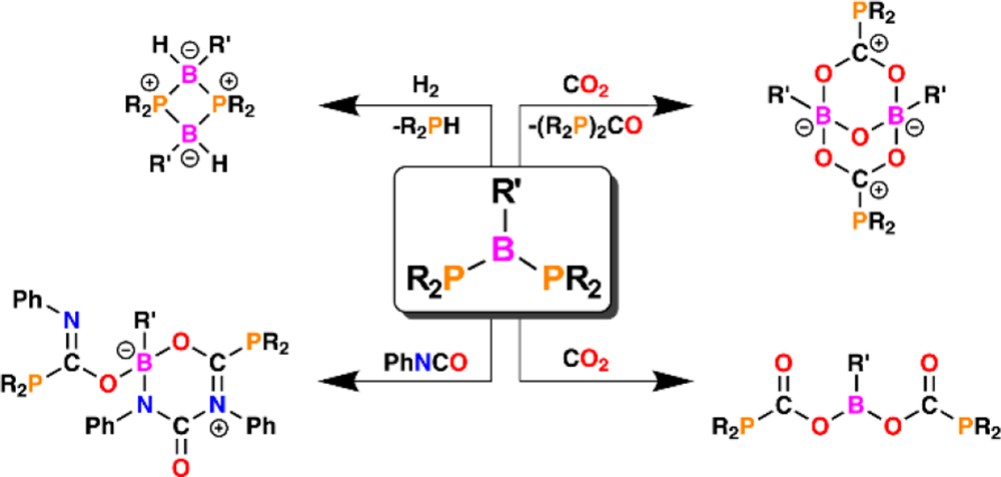

Herein, we present the first example of the activation of small molecules by P–B–P bond systems. The reactivity study involves reactions of two selected diphosphinoboranes, (*t*-Bu_2_P)_2_BPh (**1′**) and (Cy_2_P)_2_BN*i*Pr_2_ (**2**), that differ in terms of their structural and electronic properties for the activation of dihydrogen, carbon dioxide, and phenyl isocyanate. Diphosphinoborane **1′** activates H_2_ under very mild conditions in the absence of a catalyst with the formation of the dimer (*t*-Bu_2_PB(Ph)H)_2_ and *t*-Bu_2_PH. Conversely, diphosphinoborane **2** did not react with H_2_ under the same conditions. The reaction of **1′** with CO_2_ led to the formation of a compound with an unusual structure, where two phosphinoformate units were coordinated to the PhBOBPh moiety. In addition, **2** reacted with CO_2_ to insert two CO_2_ molecules into the P–B bonds of the parent diphosphinoborane. Both diphosphinoboranes activated PhNCO, yielding products resulting from the addition of two and/or three PhNCO molecules and the formation of new P–C, B–O, B–N, and C–N bonds. The products of the activation of small molecules by diphosphinoboranes were characterized with nuclear magnetic resonance (NMR) and infrared (IR) spectroscopy, single-crystal X-ray diffraction, and elemental analysis. Additionally, the reaction mechanisms of the activation of small molecules by diphosphinoboranes were elucidated by theoretical methods.

## Abstract 2

The first example of the activation of dihydrogen, carbon dioxide, and phenyl isocyanate by diphosphinoboranes is presented.

## 1 Introduction

**Chart 1:**
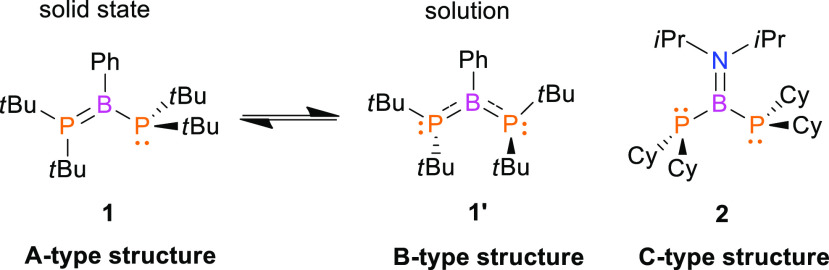
Structures of Diphosphinoboranes **1/1**′ and **2** Selected for Reactivity Studies

Utilizing the main group elements for the activation of small molecules is constantly gaining attention. This is a competitive method compared to the application of expensive and toxic transition metal complexes. Some of the attractive compounds in this area are those containing low-valent phosphorus and boron atoms, namely, phosphinoboranes and diphosphinoboranes. The chemistry of phosphinoboranes was first explored mainly by Paine and Nöth^1^ as well as Power,^2^ but until recently, reactivity studies were limited to reactions leading to their oxidation or P–B bond dissociation. Although diphosphinoboranes have also been known since the middle of the 20th century,^3,4^ their chemistry is a relatively unexplored area of research. There are only several reports on the synthesis^3−7^ and even fewer on the isolation and structural properties^8−10^ of these species. Recently, we reported^11^ the synthesis and characterization of a new family of diphosphinoboranes with the general formula R_2_PB(R′′)PR′_2_. We also showed that it is possible to tune their properties depending on the substituents on phosphorus and boron atoms. Diphosphinoboranes were obtained in the salt elimination reactions of lithium phosphides R_2_PLi (R_2_P = *t*-Bu_2_P, Cy_2_P, Ph_2_P, and *t*-BuPhP) and dibromoboranes R′BBr_2_ (R′ = N*i*Pr_2_ and Ph). It is also possible to synthesize these species with diversified phosphanyl groups. The obtained P–B–P compounds can be classified into three groups depending on their structural and electronic properties, which were further elucidated by density functional theory (DFT) calculations: (A) in which P atoms carry strong electron-donating substituents and B atoms possess electron-accepting phenyl groups, which leads to a structure with one double and one single P–B bond and diversified planar and pyramidal geometries of P atoms; (B) in which the P–B distances are comparable and both phosphorus atoms are pyramidal; and (C) in which B atoms are attached to amino groups with strong donor abilities that allow obtaining compounds with two very long P–B bonds and two pyramidal P atoms (A**-**, B**-**, and C**-**type structures are presented in Chart 1).

Boron reagents are widely used in organic synthesis, e.g., to functionalize unsaturated sites by hydroboration^12^ and diboration^13−15^ or to activate C–H bonds, as in borylation reactions.^16^ Hence, both the expanding commercial applications of organoboron compounds and the increasing interest in small-molecule activation have prompted studies on the corresponding phosphinoboration reaction.^17^ A relatively weak π-bonding for the P–B moiety results in the presence of an accessible lone pair and a vacant p-orbital on the phosphorus and boron atoms, respectively, which may act as reactive centers.^18,19^ Indeed, the Stephan group demonstrated H_2_ cleavage^20^ and dehydrogenation of ammonia borane^18^ with phosphinoboranes with the formula R_2_P–B(C_6_F_5_)_2_ (R = *t*-Bu, Cy, and Mes). Then, the Su group showed that *t*-Bu_2_P–B(biphenyl) not only cleaves H_2_ but also undergoes 1,2-addition reactions with benzophenone, dimethylbutadiene, and acetonitrile.^21^ The Westcott group synthesized phosphinoboronate esters of the form R_2_PBpin (R = Ph and Cy)^17^ and Ph_2_PBcat and, in cooperation with the Stephan group, explored the broad applicability of the phosphinoboration reaction, reporting on 1,2-additions to a wide range of unsaturated organic species: aldehydes, ketones, imines,^17,22^*N*-heterocycles,^23^ heteroallenes,^24^ diazobenzene,^25^ diazomethanes,^26,27^ acyl chlorides,^28^ and alkynes.^29^ In addition, they described the formation of R_2_PCO_2_BR′_2_ species in the stoichiometric reaction of R_2_PBpin, R_2_PBMes, and R_2_PBcat with CO_2_ (R = *t*-Bu, Ph, and Mes), of which Bcat-containing B/P reagents provide access to diphospha-ureas as a result of double 1,2-phosphaaddition to CO_2_.^24,30^ Our scientific interests have met at this point. Recently, we reported on BPh_3_-supported diphosphination of CO_2_ and CS_2_ by applying P–P bond systems of the form *t*-Bu_2_PP(NRR′) (R, R′ = *i*Pr, Et).^31,32^ As a continuation of our research program targeting species capable of fixing and/or functionalizing small molecules, we decided to investigate systems bearing analogous structural motifs, in which we replaced one phosphorus atom with a boron atom. We found that diaminophosphinoboranes RR′PB(N*i*Pr_2_)_2_ (R and R′ = *t*-Bu and Ph) react with CO_2_ to form RR′P–C(O)–O–B(N*i*Pr_2_)_2_ species, following the previously described reactivity path,^30^ and we showed that the more nucleophilic the P atom of RR′PB(N*i*Pr_2_)_2_, the faster the complete conversion into the product.^33^ Despite the suppressed Lewis acidity of the B(N*i*Pr_2_)_2_ moiety, *t*-Bu_2_PB(N*i*Pr_2_)_2_ also activates SO_2_ and N_2_O to cleanly and quantitatively afford RR′P–S(O)–O–B(N*i*Pr_2_)_2_ and *t*-Bu_2_P–O–B(iPr_2_N)_2_,^34^ respectively, while remaining unreactive toward H_2_.^33^ As elucidated from the works of Stephan and Westcott as well as our study on P–B bond systems, depending on the electronic features of BRR′ fragments and, consequently, the P–B bond order, the reactivity of phosphinoboranes changes. The presence of a single P–B bond, resulting in an accessible P-lone pair and increased nucleophilicity of the P-center, is the key factor for the activation of CO_2_, while the high Lewis acidity of the boron center, leading to the multiple P–B bond character, facilitates heterolytic cleavage of H_2_.^17,20,30,33^ Having synthesized a series of the above-described diphosphinoboranes that include systems differing in P–B–P bonding,^11^ we decided to test their reactivity toward small molecules.

## 2 Results and Discussion

From the large family of diphosphinoboranes recently synthesized by our group,^11^ we selected compounds **1/1**′ and **2** for reactivity studies (Chart 1).

Although both species possess a P–B–P skeleton, they differ significantly in terms of structural and electronic features. The former exhibits significant π-interactions between P-lone pairs and the boron Lewis acidic center, which is manifested by flattening of the phosphanyl groups and significant shortening of the P–B bonds. Interestingly, the X-ray structure of **1** shows a localized P=B double bond. Although the X-ray analysis of **1** indicated that the interactions between P and B atoms lead to a structure with one double and one single PB bond, nuclear magnetic resonance (NMR) analysis, as well as DFT calculations, revealed that this is not the most energetically favorable conformation.^11^ Computational studies elucidated that both **1** and lowest-energy **1′** (Chart 1), in which P–B distances are comparable and both phosphorus atoms are pyramidal (see Figures S42 and S45), are energetically accessible at the crystallization temperature.^11^ Otherwise, in the case of diphosphinoborane **2**, the B atom is substituted by the amino group, and in this case, the N-lone pair and P-lone pairs compete for donation to the empty p-orbital of boron. As a result of this interaction, due to better orbital matching, a double B=N bond is formed, and in contrast to compound **1**, both P atoms show pyramidal geometries. The P–B bonds are essentially single bonds. The main aim of this work was to investigate the influence of the structural features of **1/1′** and **2** on the reactivity of these compounds with small molecules. Therefore, we performed reactions of the mentioned diphosphinoboranes with dihydrogen, carbon dioxide, and phenyl isocyanate.

**Scheme 1:**
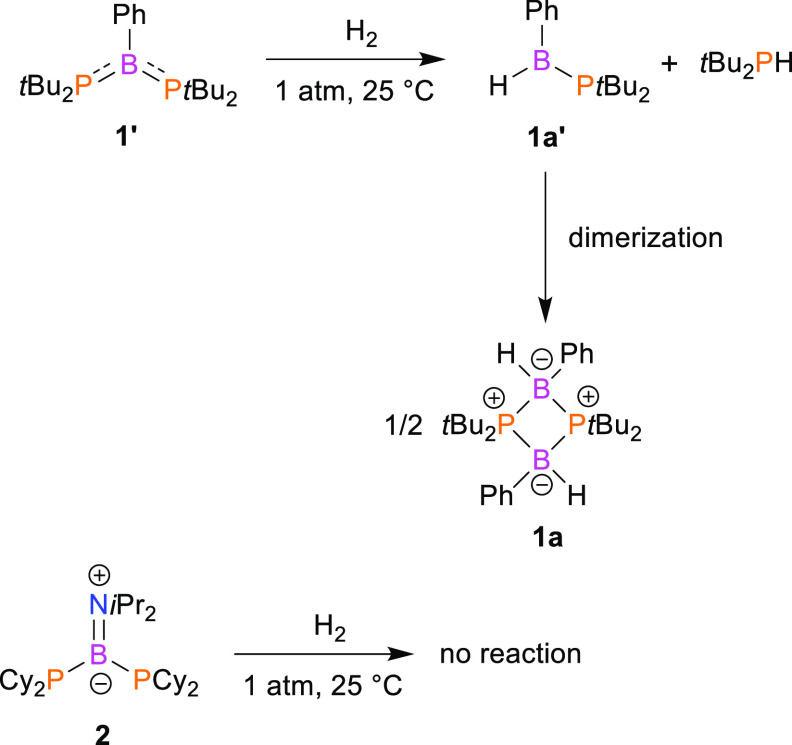
Reactions of **1** and **2** with Dihydrogen

Diphosphinoborane **1′** reacts with H_2_ (1 atm) at room temperature in the absence of a catalyst (Scheme 1). According to the ^31^P and ^11^B spectroscopic results, the reaction mixture contains only two main products in a molar ratio of 1:1, namely, the dimer (*t*-Bu_2_P–B(Ph)H)_2_ (**1a**) and free *t*-Bu_2_PH. This reaction is relatively slow; the complete conversion of **1′** into the products was observed after 2 weeks. Compound **1a** gives rise to broad resonances at 27.7 ppm and −15.8 ppm in the ^31^P{^1^H} and ^11^B spectra, respectively. Moreover, the ^1^H spectra show a characteristic broad doublet at 3.88 ppm (^1^*J*_HB_ = 105 Hz), attributed to the B–H proton. In addition, the infrared (IR) spectrum of **1a** consists of a band at 2352 cm^–1^, which is characteristic of a B–H function. The dimeric structure of **1a** in solution is confirmed by the presence of pseudotriplets at 1.42 ppm in the ^1^H spectra (*t*-Bu groups) and at 36.3 ppm in the ^13^C{^1^H} spectra (C atoms directly bound to P), where the signal splitting results from the virtual coupling of P atoms with the H and C atoms of *t*-Bu groups, respectively. In contrast to previously reported reactions of R_2_P=B(C_6_F_5_)_2_ (R = Cy and *t*-Bu) with H_2_, which led to phosphane-borane adducts (R_2_PH)·(HB(C_6_F_5_)_2_),^18,20^ in the reaction of **1′** with H_2,_ we did not spectroscopically detect the formation of analogous (*t*-Bu_2_PH)·(HB(Ph)P*t*-Bu_2_) adducts.

**Figure 1:**
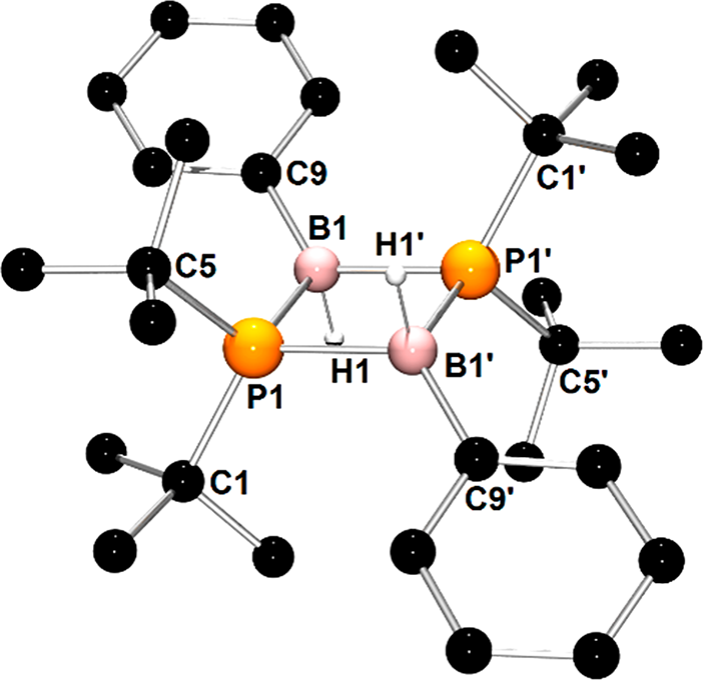
X-ray structure of **1a** showing the atom-numbering scheme. All H atoms except B−H have been omitted for clarity. One molecule of the two present in the asymmetric unit was selected.

Analytically pure dimer **1a** was isolated almost quantitatively (98% yield) as an air- and moisture-stable white solid by evaporation of the solvent and *t*-Bu_2_PH under high vacuum. The crystals of **1a** that were suitable for X-ray analysis were obtained at −20 °C from a concentrated CH_2_Cl_2_ solution. Single-crystal X-ray analysis confirmed the constitution of **1a** as a phosphinoborane dimer (Figure 1). The structures of phosphinoborane dimers with the general formula (R_2_P–B(R′)H)_2_ are very rare. These dimeric species were obtained previously by dehydroboration of phosphinoboranes, and the crystal structures were reported only for (*t*-Bu_2_P–B(Cy)H)_2_,^35^ (*t*-Bu_2_P–B(*t*-Bu)H)_2_,^35^ and (*t*-Bu_2_P–B(*i*Bu)H)_2_.^36^ The most characteristic structural feature of **1a** is the four-membered planar B1–P1–B1′–P1′ ring, which constitutes the core of the whole molecule. As expected, the geometries around the P and B atoms are pseudotetrahedral. For **1a**, the average P–B distance is 2.020 Å, whereas the average P–B–P and B–P–B angles are 91.55 and 88.45°, respectively. These metric parameters are very close to those reported for the mentioned (R_2_P–B(R′)H)_2_ dimers.^35,36^ Compound **1a** can be formally seen as a product of the addition of H_2_ molecule to *cyclo*-(PR_2_)_2_(BR′)_2_ singlet diradical. The synthesis, structure, and reactivity of such stable diradicals were investigated by the Bertrand group^37−40^ however, the reaction of *cyclo*-(PR_2_)_2_(BR′)_2_ species with dihydrogen was not tested.

In contrast to **1′**, diphosphinoborane **2** did not react with H_2_ under the same reaction conditions. The lack of reactivity of **2** toward H_2_ resulted from the quenched Lewis acidity of the boron center due to the strong π-donation from the directly bound nitrogen atom.

**Figure 2:**
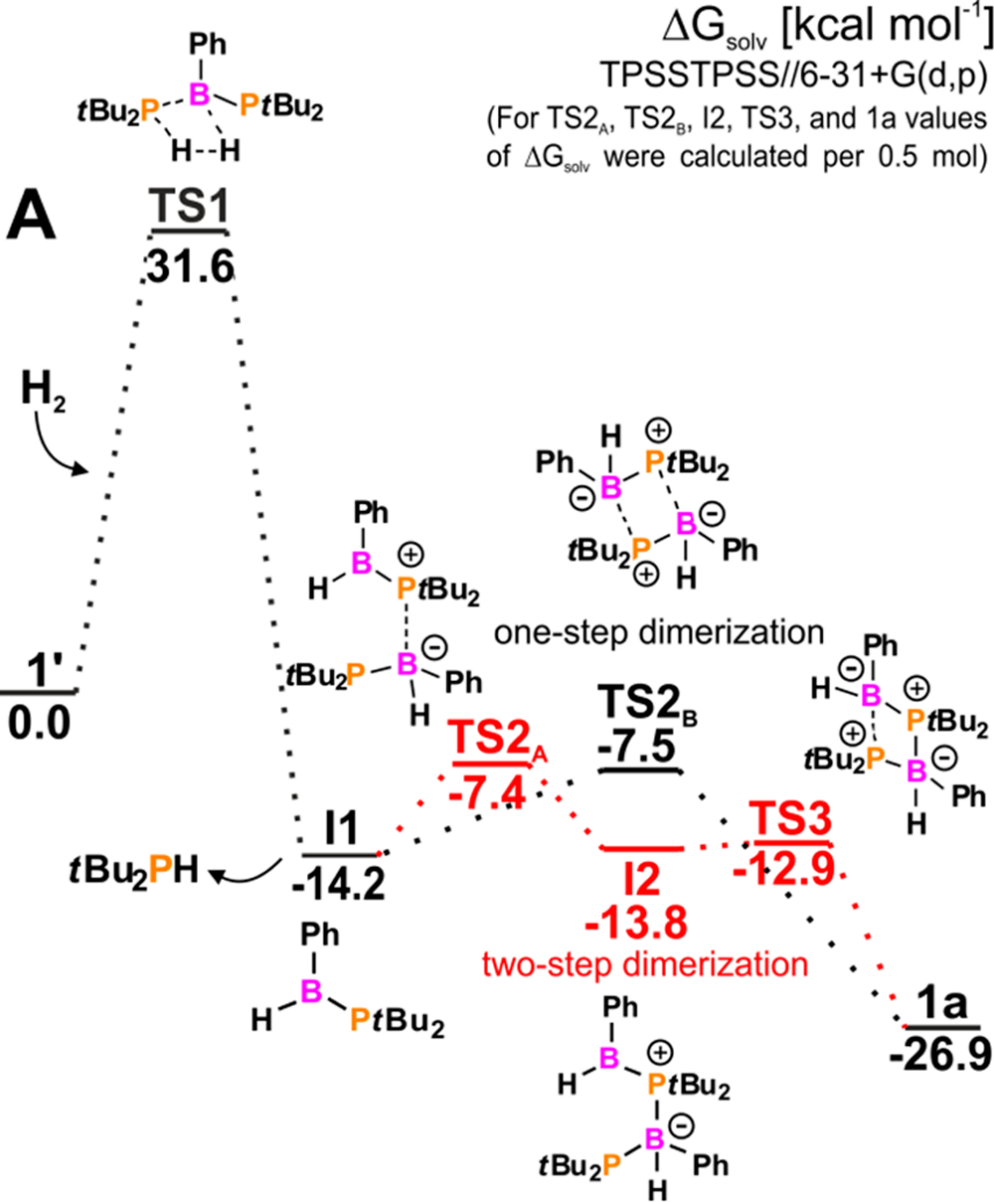
Gibbs free energy profile of reaction **1′** with H_2_. For **TS2**_**A**_, **TS2**_**B**_, **I2**, **TS3**, and **1a** values of Δ*G*_solv_ were calculated per 0.5 mol. Solvation effects were included as single-point calculations using the PCM-SMD model.

For further insight into the reactivity of **1** with dihydrogen, the reaction mechanism was studied by theoretical methods. The assumption that **1′** is present in the solution conformer, which reacts at room temperature, was additionally confirmed in the way it activates dihydrogen molecules. The calculations of the reaction mechanism confirmed that obtained product **1a** must result from the interaction of H_2_ with nearly a single P–B bond present in the structure of **1′** (with a PB Wiberg bond order of 1.183; see Figure S42). Herein, the H_2_ molecule is activated in a one-step transformation (Figure 2) involving the formation of a transition state similar to that described by Stephan et al.^20^ In the transition state, the H_2_ molecule is coordinated to the boron atom so that the H–H bond acts as a Lewis base, and simultaneously, the H atom closer to the phosphorus is inserted into the P–B bond (Figures 2 and S43). This is also the rate-determining step with an energy barrier of 31.6 kcal mol^–1^. Unlike the activation of H_2_ by *t*-Bu_2_P=B(C_6_F_5_)_2_, which leads to the hydrogenation of the double P=B bond to give *t*-Bu_2_PH-BH(C_6_F_5_)_2_,^20^ the reaction of H_2_ with **1′** splits the single P–B bond to form two products: **I1** (**1a′**) and *t*-Bu_2_PH (Figure 2, path **A**). The presence of both electrophilic B atoms and nucleophilic P atoms in **I1** (**1a′**) facilitates head-to-tail two-step dimerization to yield four-membered cycle **1a** (Figure 2). An alternative reaction mechanism assumes the activation of the H_2_ molecule by **1** either via addition to the P=B bond (path **B**, Figure S44) or via insertion into the P–B bond (path **C**, Figure S44). The first mechanism leads to the formation of adduct **1a″**, analogous to that reported by Stephan et al., while the second mechanism proceeds in the same way as described for **1′** but with a higher energy barrier of 39.5 kcal mol^–1^. Comparing the described reaction pathways **A**, **B**, and **C**, note that all are thermodynamically privileged with free energy values of −26.9, −16.4, and −25.6 kcal mol^–1^, respectively (Figures 2 and S44). Nevertheless, path **B** may be excluded because of the structure of the final product (**1a″**), while path **C** is kinetically inaccessible (with an energy barrier Δ*G*^⧧^ of 39.5 kcal mol^–1^) because of the less electrophilic B atoms in **1** than in **1′** (with values of condensed electrophilic Fukui functions *f*_E_ = 0.117 and *f*_E_ = 0.136, respectively). Hence, we found that the nature of BP bonding in **1** and **1′** is a crucial factor determining the mechanism of H_2_ activation.

**Scheme 2:**
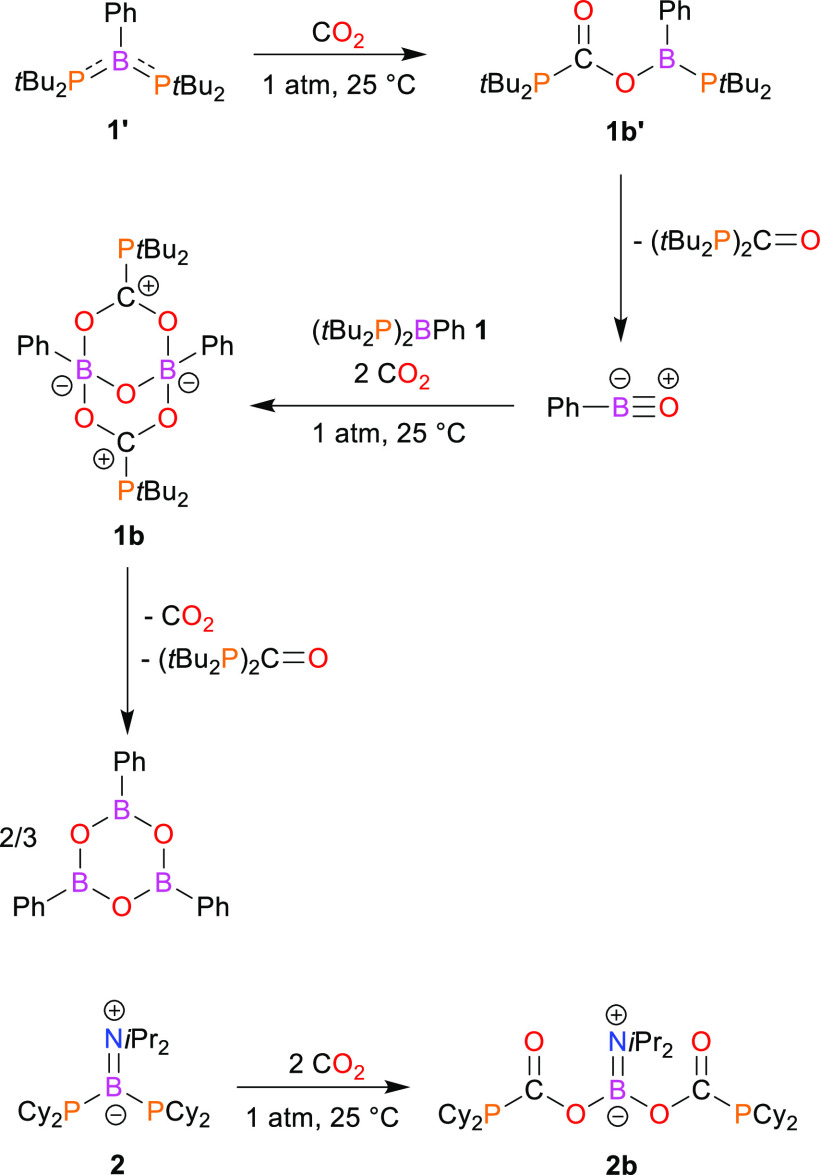
Reactions of **1′** and **2** with Carbon Dioxide

Next, we investigated the reactions of **1′** and **2** with carbon dioxide. Diphosphinoborane **1′** reacts in toluene with gaseous CO_2_ (1 atm) at room temperature with the formation of **1b** and diphospha-urea (*t*-Bu_2_P)_2_C=O in a molar ratio of 1:1 (Scheme 2). Monitoring of the reaction progress by ^31^P and ^11^B spectroscopy revealed a complete conversion of **1′** into the mentioned products after 16 h; **1b** shows a singlet at 46.4 ppm in the ^31^P{^1^H} spectra and a broad signal at 6.7 ppm in the ^11^B spectra. Furthermore, the ^13^C{^1^H} spectrum of **1b** exhibits a very characteristic doublet at 197.7 ppm, where coupling to the P atom has a value of 45.4 Hz. This suggests that the CO_2_ moiety is directly bonded to the phosphanyl group via a carbon atom. The spectral data of (*t*-Bu_2_P)_2_C=O are in agreement with those reported in the literature.^30^

**Figure 3:**
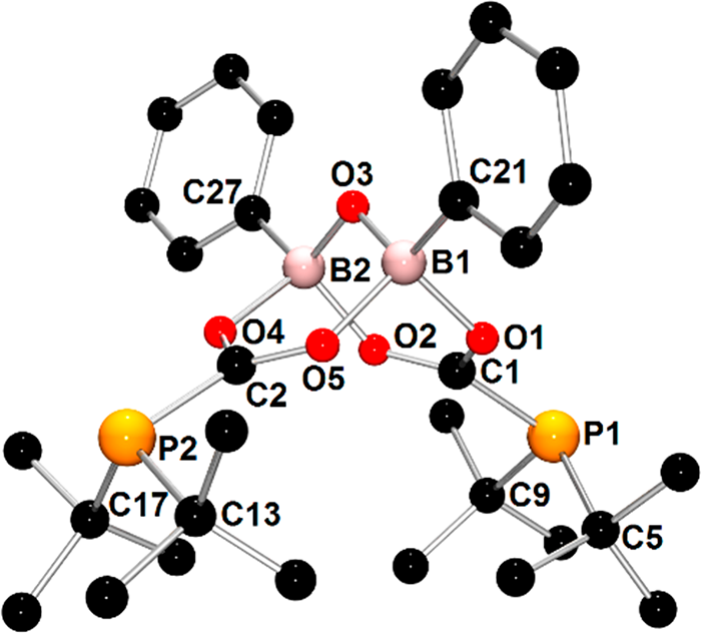
X-ray structure of **1b** showing the atom-numbering scheme. The H atoms are omitted for clarity. One molecule of the two present in the asymmetric unit was selected.

The evaporation of the solvent followed by crystallization from pentane at a low temperature gave a mixture of colorless (**1b**) and yellow crystals ((*t*-Bu_2_P)_2_C=O). The separation of the mixture components by fractional crystallization was unsuccessful. The X-ray analysis provided important information about the structural features of **1b** (Figure 3). Compound **1b** can be formally seen as an adduct of **1′** with phenyl oxoborane PhBO and two molecules of CO_2_. Hence, we assume the initial formation of intermediate **1b′** that results from the insertion of one CO_2_ molecule into the P–B bond of **1′**. Then, **1b′** eliminates (*t*-Bu_2_P)_2_C=O with the formation of phenyl oxoborane (PhBO). In the second step, PhBO reacts either with parent **1′** or with **1b′** followed by the fixation of either two or one CO_2_ molecule(s).

Organyl oxoboranes (RBOs) have been intensively studied because of their important role in Suzuki cross-coupling reactions.^41^ Monomeric RBOs are highly reactive species, and they exist in a stable cyclotrimeric form known as boroxine (RBO)_3_.^42^ Monomeric RBOs were obtained by flash pyrolysis of the corresponding boroxine and isolation in an argon matrix^43^ or using the cross-molecular beams technique via the reaction of boronyl radicals with benzene in the gas phase.^44^ The oxoboryl group can be stabilized in the coordination sphere of platinum - oxidative addition of dibromo(trimethylsiloxy)borane to [Pt(PCy_3_)_2_] leads to the formation of *trans*-[(Cy_3_P)_2_BrPt(B≡O)].^45^

The molecular structure of **1b** consists of two phosphinoformate moieties (*t*-Bu_2_PCO_2_) coordinated to the PhBOBPh unit. Each of the boron atoms is tetracoordinated and is bound to a bridging oxygen atom, two oxygen atoms of *t*-Bu_2_PCO_2_ groups, and a carbon atom of the phenyl substituent. The geometries around the carbon atoms of the two CO_2_ moieties are almost planar, whereas the geometries around the P atoms are pyramidal. The P–C bond lengths with an average value of 1.842 Å are comparable with those reported for the products of insertion of CO_2_ molecules into P–B bonds^30,33^ and are typical for single covalent P–C bonds.^46^ The average C–O bond distance (1.280 Å) is between the expected bond lengths for single and double covalent bonds (the sum of the single and double covalent bond radii for C and O is 1.38 and 1.24 Å, respectively),^46,47^ indicating delocalized π-bonding within CO_2_ fragments. The distances between the B and O atoms of CO_2_ units (average 1.587 Å) are significantly longer than typical single B–O covalent bonds (1.48 Å).^46^ Otherwise, B–O–B bonds (average 1.405 Å) are significantly shorter than the single covalent bond and approach the value expected for the double B=O bond (1.35 Å),^47^ which suggests an additional π-interaction between the lone pair of the bridging O atom and two boron centers. Compound **1b** is stable under a CO_2_ atmosphere; however, it slowly decomposes under argon to (PhBO)_3_, (*t*-Bu_2_P)_2_C=O, and CO_2_ (Scheme 2).

Next, we studied the reaction of **2** with CO_2_ under the same conditions. In contrast to a reaction involving **1′**, diphosphinoborane **2** reacts with CO_2_, yielding only one product, **2b** (Scheme 2). The complete conversion of **2** into **2b** was observed after 24 h. The ^31^P{^1^H} spectrum of the reaction mixture reveals only one singlet at 13.6 ppm, which is strongly shifted downfield in comparison to the signals of the parent diphosphinoborane (−43.3 and −44.1 ppm). Likewise, the ^11^B resonance of **2b** (22.2 ppm) differs significantly from the corresponding signal in the ^11^B spectrum of **2** (52.4 ppm). Interestingly, the ^11^B chemical shift of **2b** has a similar value to those observed for products of the insertion of CO_2_ molecules into P–B bonds in diaminophosphinoboranes (approximately 27 ppm).^33^ Furthermore, the ^13^C{^1^H} spectrum of **2b** consists of a doublet at 179.9 (^1^*J*_CP_ = 26.3 Hz), attributed to the C atom of the C=O group. The presence of carbonyl functional groups in the structure of the product was additionally confirmed by the signal at 1674 cm^–1^ in the IR spectrum of **2b**. All these spectroscopic data collectively suggest that two CO_2_ molecules are inserted into two P–B bonds of **2**, giving the product of two equivalent Cy_2_PC(=O)O moieties. Product **2b** was isolated as a colorless oil by evaporation of the solvent under reduced pressure in high yield (95%). In contrast to **1b**, **2b** is stable under an argon atmosphere, and we did not observe regeneration of **2** or the formation of any decomposition products even under high-vacuum conditions.

**Figure 4:**
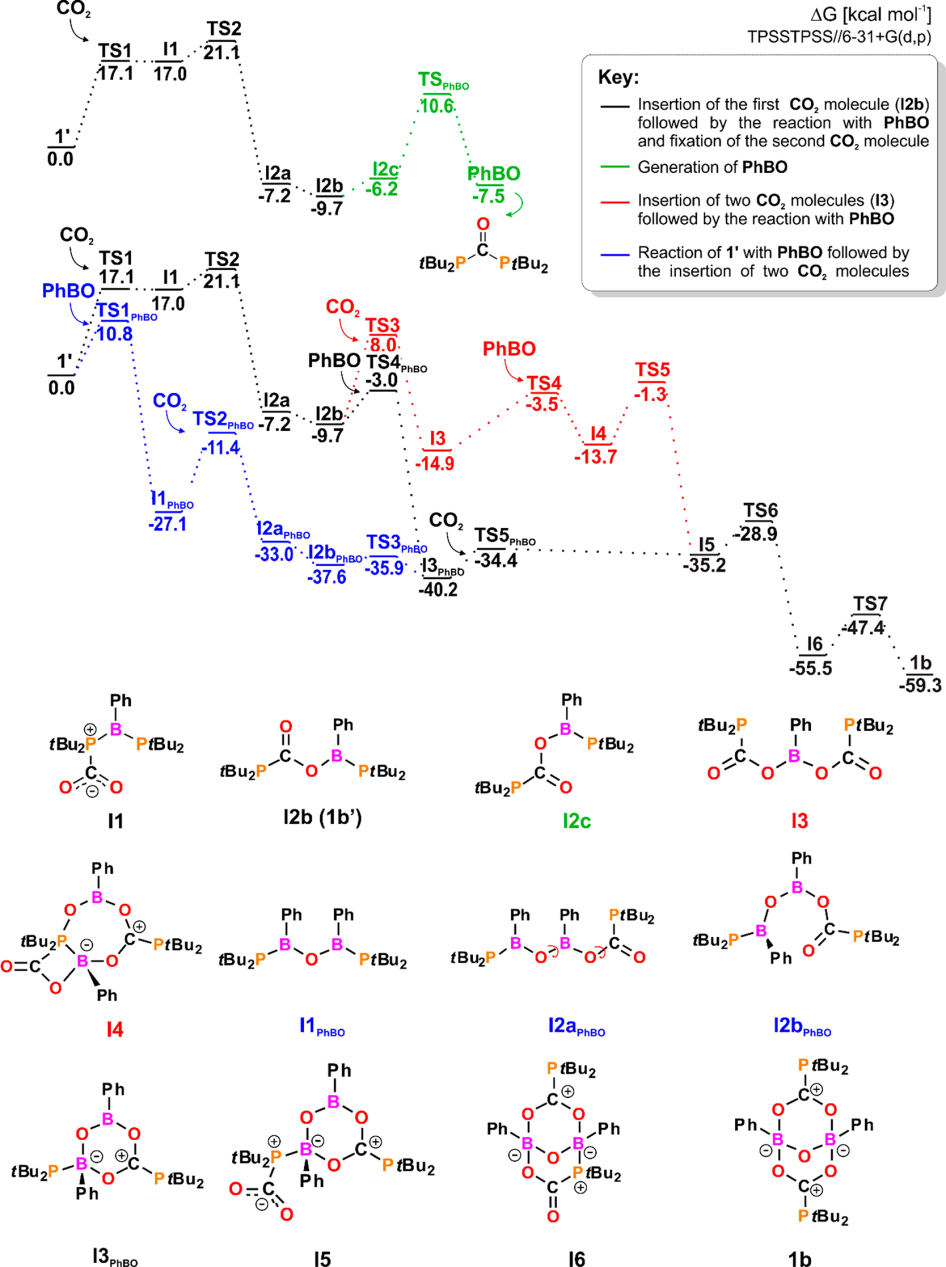
Gibbs free energy profile of reaction **1** with CO_2_.

To elucidate the differences in the reactivity of **1′** and **2** toward CO_2_, we investigated their reaction mechanism using DFT methods. The reaction of **1′** with CO_2_ starts with a nucleophilic attack of the P-lone pair on the **C**O_2_ carbon atom, resulting in intermediate **1′**-CO_2_ adduct **I1** (Figure 4; see Figures S79–S91 for the structures of TS). The second, rate-limiting step of the reaction with an energy barrier of 21.1 kcal mol^–1^, proceeds via the simultaneous formation of B–O and cleavage of the P–B bond, followed by a rotation about the C–O bond to give **I2b** (**1b′** on Scheme 2). Unlike **I2a**, in which the empty p-orbital of the B atom interacts with the P-lone pair, in **I2b**, a stronger donor–acceptor interaction involving the O-lone pair accounts for the lower energy of the latter. Next, the reaction may proceed in one of two ways: either toward the insertion of a second CO_2_ molecule (**I3**) or toward the formation of highly reactive **PhBO**. Both paths have similar kinetic accessibility with energy barriers of 17.7 and 20.3 kcal mol^–1^, respectively. The first path (red) involves the formation of a four-membered PCOB ring in the transition state as a result of the simultaneous interaction of both P- and B-centers with CO_2_ to give **I3**. The second path (green) starts with rotation about the B–O bond (**I2c**), which facilitates nucleophilic attack of the P atom on the **C**=O atom to yield phospha-urea with the elimination of **PhBO**. Once **PhBO** is formed, it may react either with substrate **1′** (blue path) or with the products of single (**I2b**, black path) or double (**I3**, red path) CO_2_ insertion into the P–B bond, with energy barriers of 15.7, 6.7, and 13.6 kcal mol^–1^, respectively (Figure 4). The attack of the highly electrophilic B atom of PhBO on **1′** incorporates the **PhBO** molecule into the P–B bond, providing a new P–B–O–B–P structural motif in **I1**_**PhBO**_. Subsequent one-step fixation of the CO_2_ molecule followed by cyclization of the obtained product (**I2b**_**PhBO**_, resulting from rotation about the B–O bonds in **I2a**_**PhBO**_) gives six-membered ring intermediate **I3**_**PhBO**_. At this point, the blue and black reaction paths merge, as **I3**_**PhBO**_ may also be formed in the direct reaction of **I2b** with **PhBO**. The presence of an active P–B bond in **I3**_**PhBO**_ together with the high nucleophilicity of the P-atom (*f*_N_ = 0.269, the most nucleophilic one in the overall reaction) facilitates the low-barrier fixation of a second CO_2_ molecule, which upon binding to the **P***t*-Bu_2_ atom, gives adduct **I5**. Herein, the red and black reaction paths join as they proceed via the same intermediate, **I5**. Incorporation of **PhBO** into the structure of **I3** leads to the formation of new B–O and P–O bonds in **I4**. **I4** features two joined rings, four- and seven-membered rings, which subsequently rearrange to give more energetically favorable **I5**. Through the attachment of the C=**O** atom to the tricoordinated B atom, **I5** transforms into bicyclic intermediate **I6**, in which by replacing the P–B bond with the B–O bond, final product **1b** is generated in an exergonic process with a free energy value of −59.3 kcal mol^–1^. Given that the considered reaction paths lead to the same intermediates, and thus to **1b**, we assume that activation of CO_2_ by **1′** proceeds via all three mechanisms simultaneously. All transformations following rate-limiting step **TS2** are kinetically accessible since the values of the respective energy barriers are lower than that of **TS2**. The experimentally observed decomposition of **1b** (Scheme 2) may be justified based on the thermodynamics, as rearrangement into (PhBO)_3_ liberates a significant amount of energy (free energy value of −114.7 kcal mol^–1^).

**Figure 5:**
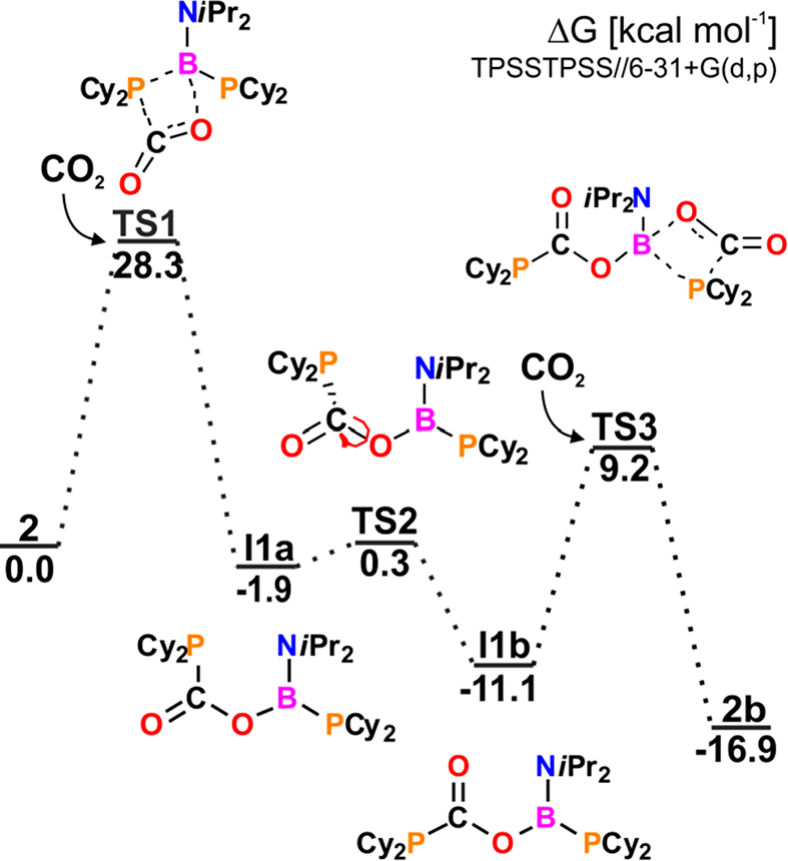
Gibbs free energy profile of reaction **2** with CO_2_.

Although suppressed acidity of the boron center in **2** (with the value of *f*_E_ = 0.079 compared to *f*_E_ = 0.136 in **1′**) precludes a reaction with H_2_, our previous studies revealed that the nucleophilicity of the P center (*f*_N_ = 0.157 and 0.138 in **2**) rather than electrophilicity of the B center is a crucial factor in predicting the reactivity of the system toward CO_2_. Unlike **1′**, the reaction of **2** with CO_2_ proceeds via a simple, three-step mechanism (Figure 5). The initial step involves the simultaneous formation of B–O and P–C bonds and cleavage of the P–B bond in rate-determining transition state **TS1** (with an energy barrier of 28.3 kcal mol^–1^) followed by a rotation about the C–O bond to give **I1b**. Subsequent fixation of the second CO_2_ molecule proceeds through an analogous four-membered PCOB transition state, **TS2**, to finally form **2b**. Fixation of two CO_2_ molecules by **2** is a thermodynamically favored reaction with a free energy value of −16.9 kcal mol^–1^. Similar to the reaction of **1′** with CO_2_, initially formed intermediate **I1a** transforms into **I1b**, in which the interaction of the empty p-orbital of B with the P-lone pair orbital is replaced by an interaction with the O-lone pair.

**Scheme 3:**
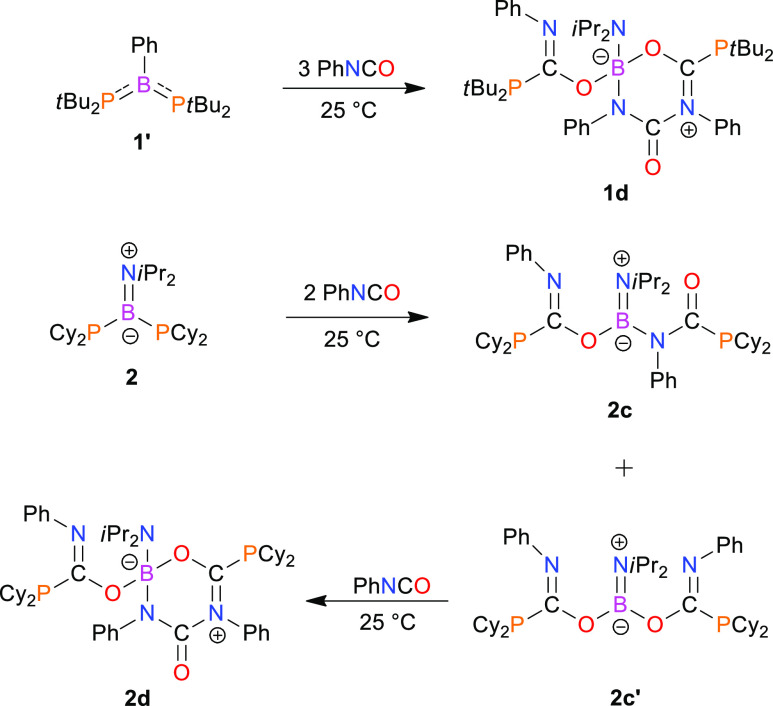
Reactions of **1′** and **2** with Phenyl Isocyanate

Then, we probed the reactions of **1′** and **2** with phenyl isocyanate using different stoichiometries of substrates. Diphosphinoborane **1′** reacts with 2 equiv of PhNCO in toluene at room temperature to form a mixture of several products and starting material **1′**. All attempts to isolate a pure product of double PhNCO-addition to **1′** from the reaction mixture were unsuccessful. After that, we conducted an experiment involving **1′** and 3 equiv of PhNCO. According to ^31^P and ^11^B NMR spectra, only one product **1d** was formed within 24 h (Scheme 3). This reaction is very clean, and pure **1d** was isolated by the evaporation of the solvent from the reaction mixture, in high yield (99%). The ^31^P{^1^H} chemical shifts of **1d** (41.9 ppm, 29.1 ppm) are high-field-shifted in comparison to the corresponding resonance of parent **1′** (54.7 ppm) and indicate the presence of two inequivalent P atoms. The significant difference in ^11^B chemical shifts of **1′** (68.6 ppm) and **1d** (6.2 ppm) is in agreement with different coordination numbers of boron atoms in both compounds.

**Figure 6:**
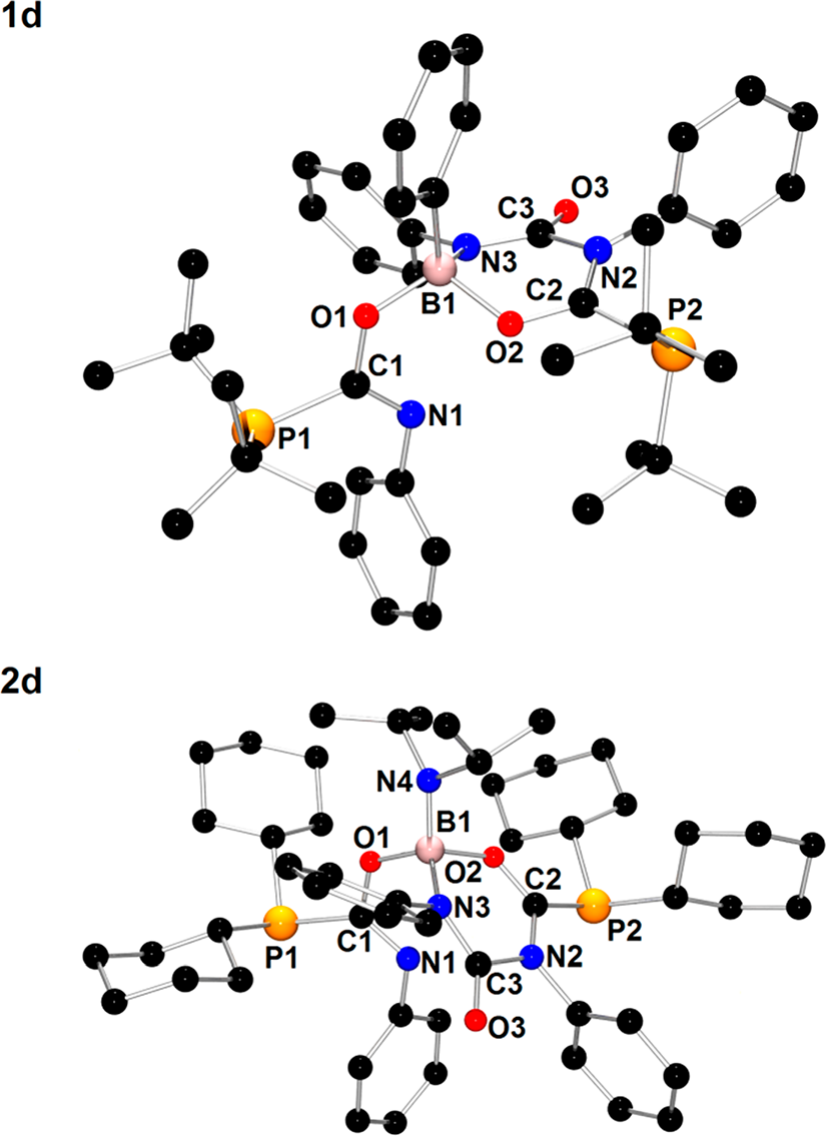
X-ray structures of **1d** and **2d** showing the atom-numbering scheme. The H atoms are omitted for clarity.

X-ray quality yellow crystals of **1d** were grown from a toluene solution at −20 °C. The molecular structure of **1d** (Figure 6) reveals the formation of several new chemical bonds, in particular, two P–C bonds, two B–O bonds, one B–N bond, and one C–N bond. One PhNCO molecule was inserted into a P–B bond of **1′** with the formation of *t*-Bu_2_P1C1(=N1Ph)O1 unit connected to the B1 atom via an oxygen atom. The most striking structural feature of **1d** is a six-membered ring composed of a B1 atom, O2, C2, and N2 atoms of the second PhNCO moiety, and C3 and N3 atoms of the third PhNCO unit. Although there are almost planar geometries around C2, N2, C3, and N3 atoms, the mentioned six-membered ring is not planar because of the coordination of O2 and N3 atoms to pseudotetrahedral B1 atom. Additionally, the B1 atom is coordinated by the carbon atom of the phenyl group and O1 atom of the *t*-Bu_2_P1C1(=N1Ph)O1 moiety. The bond lengths within the B1–O2–C2–N2–C3–N3 six-membered ring, such as B1–O2 (1.575(2) Å), B1–N3 (1.547(2) Å), and C3–N2 (1.483(2) Å), span the range of values characteristic of single covalent bonds,^46^ whereas the short distances of the bonds C2–O2 (1.274(2)Å), C2–N2 (1.337(2)Å), and C3–N3 (1.336(2)Å) suggest the partial multiple bond character of these bonds.^46,47^

**Figure 7:**
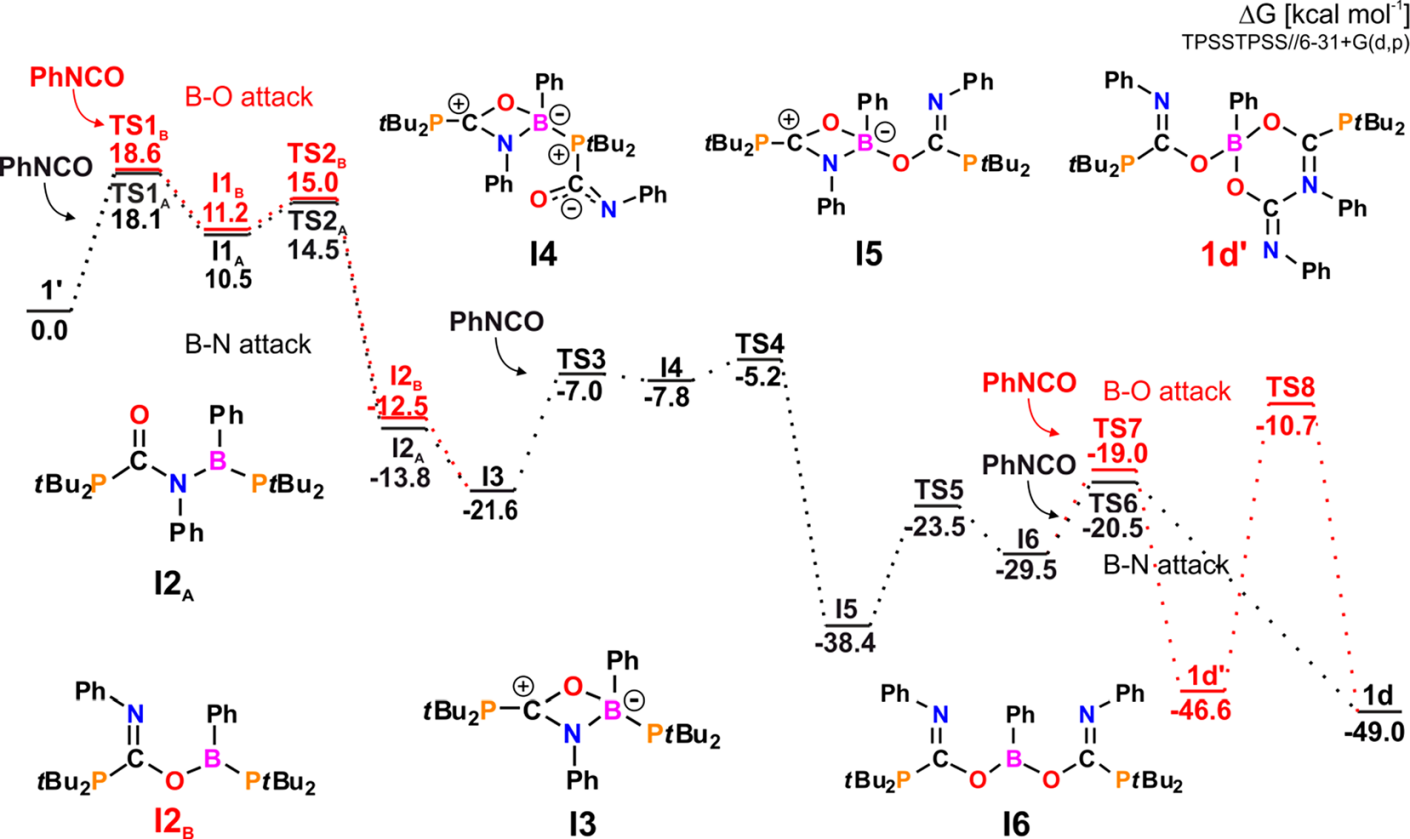
Gibbs free energy profile of reaction **1′** with PhNCO.

According to the Gibbs free energy profile, in the reaction of **1′** with PhNCO, all accessible reaction paths lead to final product **1d** with the calculated free energy Δ*G*°_298_ of −49.0 kcal mol^–1^ (Figure 7; see Figures S102–S111 for the structures of TS). First, the rate-determining step (with Δ*G*^⧧^_298_ of 18.1 kcal mol^–1^) of the P-nucleophilic attack of **1′** on PhN**C**O results in P–C bond formation, followed by binding of nitrogen to the boron atom to give **I2**_**A**_. Although PhNCO may also be inserted into the P–B bond through the PhN**C**=**O** unit, giving **I2**_**B**_ (red path), both **I2**_**A**_ and **I2**_**B**_ transform into a species possessing four-membered COBN ring **I3** in a barrierless step. Consequently, due to increased steric hindrance around the tetracoordinated B atom, fixation of the second isocyanate molecule proceeds only via a more accessible PhN**C**=**O** fragment to form P–C and B–O bonds in **I5**. Binding of the third PhNCO molecule involves ring-opening in **I5** in the first step and further reaction of the obtained **I6** either with Ph**N**=**C**O or with the PhN**C**=**O** fragment to form **1d** and **1d′**, respectively. However, as the generation of **1d′** and the subsequent transformation of **1d′** to **1d** are not thermodynamically or kinetically favorable, we assume that PhNCO is inserted solely via the mechanism described by the black reaction path (through **TS6**).

**Figure 8:**
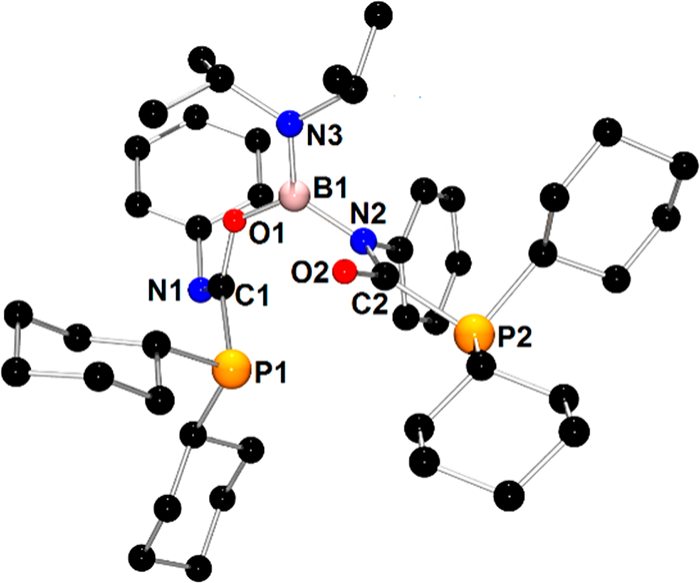
X-ray structure of **2c** showing the atom-numbering scheme. The H atoms are omitted for clarity.

The weaker Lewis acidic properties of the boron center of **2** than that of **1′** influence the product of the reaction of **2** with PhNCO. Unlike **1′**, diphosphinoborane **2** reacts with 2 equiv of PhNCO in toluene at room temperature, yielding **2c** as the main product (Scheme 3). The NMR and IR spectroscopic data indicate that PhNCO molecules were inserted into both P–B bonds of **2**. The ^31^P{^1^H} NMR spectrum of **2c** shows two sharp singlets at 7.6 and 2.5 ppm, whereas the ^11^B NMR spectrum consists of only a broad singlet at 24.9 ppm. The ^31^P{^1^H} and ^11^B data of **2c** differ significantly from the spectroscopic data of parent **2**, which indicates changes in the coordination environment of P and B atoms. Additionally, the insertion of two PhNCO molecules into P–B bonds is confirmed by the presence of two doublets in the ^13^C{^1^H} spectrum of **2c** at 183.4 and 161.7 ppm with couplings to P atoms with values of 31.8 and 22.7 Hz, respectively. Furthermore, characteristic bands attributed to C=O, C=N, and NCO groups are observed in the IR spectrum of **2c** at 1629, 1612, and 1589 cm^–1^, respectively. X-ray-quality crystals were obtained from a concentrated toluene solution at −20 °C. The molecular structure of **2c** is presented in Figure 8. The X-ray analysis is in agreement with the spectroscopic data of **2c** in solution and confirms the insertion of two PhNCO molecules into the P–B bonds of **2**. Interestingly, two PhNCO units are bound to boron atoms in a different mode. The first one is connected to the boron atom via an oxygen atom, whereas the second one coordinates to boron via a nitrogen atom. Similar to parent **2**, the planar geometry around the B1 atom and N3 atom of the N*i*Pr_2_ group and the very short B1–N3 distance (1.396(7) Å) indicate that the multiple bond character of this bond is retained. The geometry around the N2 atom directly bound to boron is also planar; however, the B1–N2 distance (1.493(8) Å) is in the range of a single covalent bond. The relatively short B1–O1 distance with a value of (1.389(7) Å) indicates an additional interaction between the lone pair of the oxygen atom and the boron center. The presence of C1=N1 (1.278(8) Å) and C2=O2 (1.214(7) Å) double bonds implies the planar geometry around C1 and C2 atoms. The geometries around P1 and P2 atoms are pyramidal, and P1–C1 (1.862(6) Å) and P2–C2 (1.882(6) Å) distances span the typical range for single P–C bonds.^46^

Although **2c** may be cleanly isolated in high yield as an analytically pure crystalline product, the ^31^P{^1^H} NMR spectra of the reaction mixture revealed the presence of an additional product attributable to the low-intensity broad signal at 1.68 ppm. This result suggested the simultaneous formation of second isomer **2c′** that is formed together with **2c** by evaporation of the solvent. Indeed, elemental analysis of the obtained white solid confirmed that the ratio of elements within the mixed sample of **2c** and **2c′** is identical to that calculated for **2c**, which indicates that **2c** and **2c′** have the same chemical formula. We assumed that in this case the insertion of two PhNCO molecules leads to the formation of two B–O bonds instead of B–N and B–O as in **2c** (Scheme 3). Due to the complex nature of the NMR spectra of the **2c/2c′** mixture together with the lower intensity and broadness of the **2c′** signals as well as the fact that we did not isolate **2c′** in a pure crystalline form, we were not able to fully characterize **2c′**. Nevertheless, the assumption that **2c′** exists in the solution in the form of species bearing two B–O bonds was indirectly confirmed in the reaction of **2** with an excess of PhNCO.

The reaction of **2** with a 6-fold excess of PhNCO led to the formation of a mixture of **2c** and **2d** in a molar ratio of 3:5 (Scheme 3). Monitoring of the reaction progress by ^31^P{^1^H} NMR revealed that the molar ratio of the initially formed mixture of **2c** and **2c′** changes with time in an interesting way. The broad signal of **2c′** disappears with an increasing intensity of sharp signals of **2d**, while the intensity of **2c** remains unchanged. An analogous experiment with an excess of PhNCO conducted for isolated, pure isomer **2c** showed that **2d** is not formed (even after over a month of stirring at room temperature). Hence, we assume that **2d** resulting from the addition of three molecules of PhNCO to diphosphinoborane **2** is formed solely via intermediate **2c′** (Scheme 3). **2c** and **2d** insert PhNCO in competitive reactions rather than in consecutive reactions, as **2c** does not transform into **2d**. Analytically pure **2d** was precipitated from the concentrated reaction mixture at −20 °C as a crystalline yellow solid in 47% yield. The identity of **2d** was unambiguously confirmed by NMR, IR spectroscopy, and single-crystal X-ray diffraction studies. The spectroscopic and structural properties of **2d** are analogous to those observed for **1d**. The structural and spectroscopic data of **2d** are presented in the Supporting Information. The X-ray structural analysis of **2d** confirmed the addition of three molecules of PhNCO as observed previously for **1d** (Figure 6). Considering the bonding in **2d**, we note that **2d** results from the insertion of the third PhNCO molecule into the structure of **2c′** rather than **2c**. In the structure of **2d**, there are two Cy_2_PC(=NPh)–O–B fragments that must be formed through the attachment of phosphorus to the PhN**C**O atom and boron to the PhNC**O** atom. The third molecule is bound through the Ph**N**=**C**O fragment to B and N atoms in **2c′** with the formation of B–N and C–N bonds, respectively.

**Figure 9:**
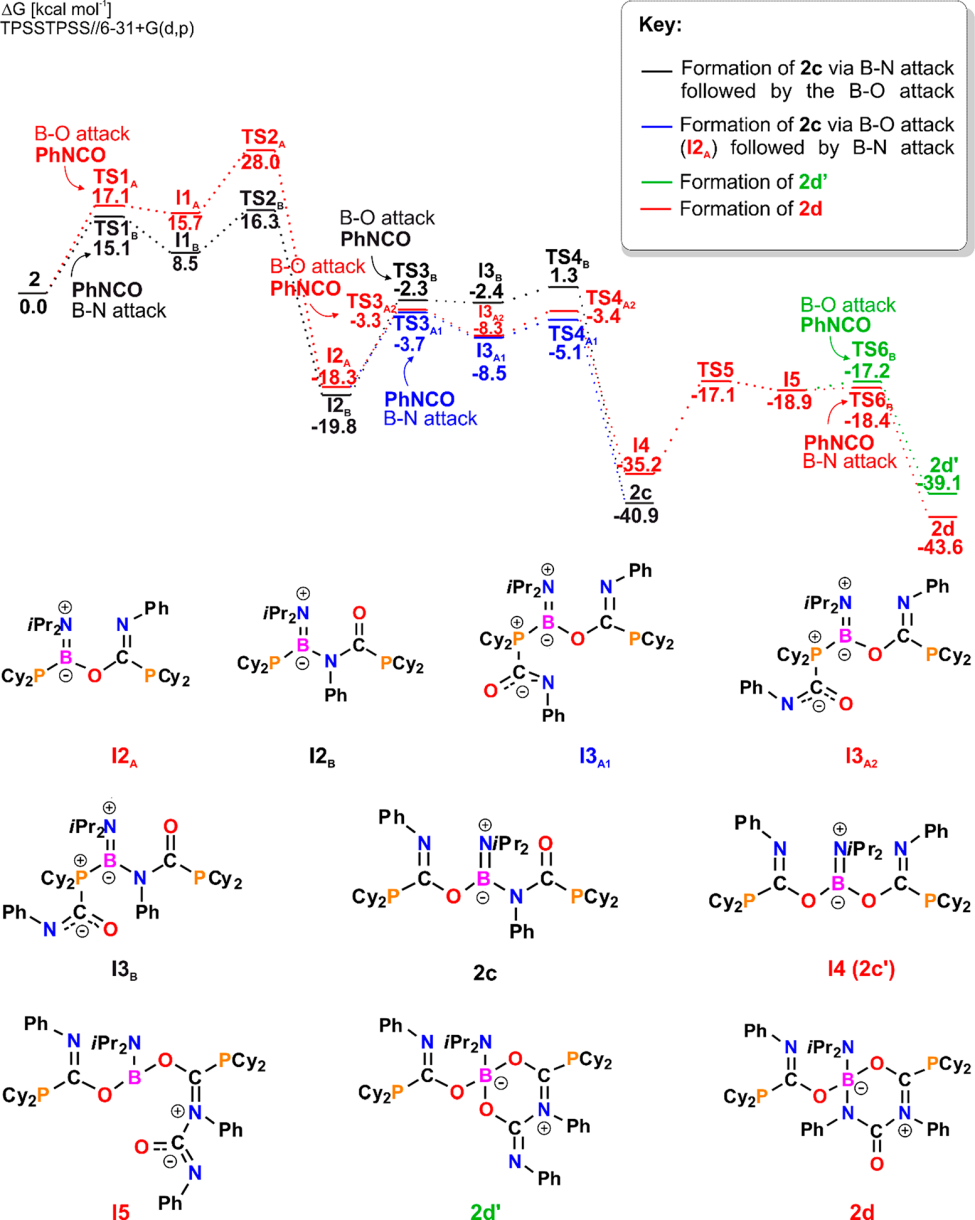
Gibbs free energy profile of reaction **2** with PhNCO.

To understand the differences in reactivity of **1′** and **2** toward PhNCO, we studied reactions of **2** with PhNCO by theoretical methods. While consecutive insertion of PhNCO molecules into the structure of **1′** cleanly yields **1d**, the reaction of **2** with PhNCO gives two competitive products: **2c** and **2d**. According to the Gibbs free energy profile (Figure 9; see Figures S130–S142 for the structures of TS), the reaction leading to the formation of **2c** (black path) is faster than that of **2d** (red path) with energy barriers of 21.1 and 28 kcal mol^–1^, respectively, and, simultaneously, less thermodynamically favorable (with Δ*G*^o^_298_ of −40.9 kcal mol^–1^ compared to −43.6 kcal mol^–1^ for **2d**). In each case, fixation of the first and second PhNCO molecules proceeds analogously via the initial attachment of phosphorus to the carbon atom, giving a P–C bond followed by the formation of a B–O or B–N bond. An optimal mechanism of **2c** formation involves the insertion of PhNCO molecules into the P–B bonds via Ph**N**=**C**O (**I2**_**B**_) and subsequently through the PhN**C**=**O** fragment (**2c**); the opposite order is less favorable. Attachment of boron to oxygen atoms during the insertion of the second PhNCO is the rate-determining step of the reaction (**TS4**_**B**_). This may result from the low Lewis acidity of the boron center in **2**. Similarly, during the generation of **2d**, which involves the double insertion of the isocyanate molecule through the PhN**C**=**O** fragment, giving two B–O bonds, the formation of the first one is the rate-determining step of the reaction (**TS2**_**A**_). Once **2c′** is formed (**I4**), it may bind the third PhNCO molecule via a C–N bond (**I5**), followed by the nucleophilic attack of nitrogen (**TS6**_**B**_) or oxygen (**TS6**_**A**_, green path) on the boron atom with the formation of **2d** and **2d′**, respectively. The formation of **2d** is both thermodynamically and kinetically favorable compared with the formation of **2d′**; however, it is worth mentioning that the ^31^P{^1^H} NMR spectrum of the reaction mixture contains weak signals that may be attributed to **2d′** (Figure S36). Differences in the Lewis acidity of the boron atoms of **1′** and **2** may also explain why **1′** tends to form cyclic intermediates with tetracoordinate B atoms, while the generation of these species was not observed during the relaxed scan of the potential energy surface of **2** reacting with PhNCO.

## 3 Conclusions

Our reactivity study revealed that diphosphinoboranes can be successfully applied for the activation of small molecules. The unique structure and electronic properties of diphosphinoboranes that possess P–B–P bonding provide new possibilities for designing systems for small molecule activation. Despite the presence of two direct P–B bonds, the diphosphinoboranes can be seen as intramolecular frustrated Lewis pairs because of two Lewis basic centers and one Lewis acidic center in these molecules. As shown by mechanistic studies, the reactivity of species with the P–B–P skeleton can be controlled by tuning the nucleophilic/electrophilic properties of the P and B reactive centers. The presence of an electrophilic boron center is necessary for the activation of dihydrogen, where the crucial reaction step is the formation of a Lewis acid–base adduct between the boron center and the H–H bond. Otherwise, for the activation of carbon dioxide and isocyanates, the presence of a nucleophilic P atom possessing an accessible lone pair is required, and in this case, the initial, crucial reaction step is the formation of the Lewis acid–base adduct with a P–C bond. Diphosphinoborane **1′** satisfies both conditions and activates either dihydrogen or carbon dioxide/phenyl isocyanate. To the best of our knowledge, this is the first example of a compound with a P–B bond exhibiting such versatile reactivity. Moreover, diphosphinoborane **2** satisfies only the second condition (the electron-donating amino group significantly reduces the electrophilic properties of the boron atom), which is manifested by the lack of reactivity toward dihydrogen and the high reactivity toward carbon dioxide and phenyl isocyanate. The diversity and unique structural properties of the obtained reaction products confirm that diphosphinoboranes can be used as activators of strong chemical bonds and as a source of R_2_P and RB building blocks in chemical syntheses.

## 4 Experimental Section

### 4.1 General Information

All manipulations were carried out under a dry argon atmosphere by using flame-dried Schlenk-type glassware on a vacuum line or in a glovebox. In the reactions with gaseous reagents, the toluene solution of the substrate was slowly frozen in a liquid nitrogen bath, evacuated to 0.01 Torr, and backfilled with H_2_ or CO_2_ (1 atm). Then, the cooling bath was removed and the reaction mixture was allowed to warm to room temperature. During thawing of the reaction mixture, the Schlenk flask was opened to a line connected with a pressure-relief system to avoid overpressure in the vessel. Solvents were dried by standard procedures over Na(K)/K/Na/benzophenone and distilled under argon. 1D (^31^P, ^13^C, ^11^B, and ^1^H) and 2D NMR spectra in C_6_D_6_, toluene-*d*_8_, or CDCl_3_ solution were recorded on a Bruker AV400 MHz spectrometer (external standard TMS for ^1^H and ^13^C; 85% H_3_PO_4_ for ^31^P; BF_3_·Et_2_O for ^11^B) at ambient or lower temperature. Reaction progress was monitored by ^31^P{^1^H} and ^11^B NMR spectra of reaction mixtures. The FTIR spectra of crystalline products were recorded using a Nicolet iS50 FT-IR spectrometer equipped with the Specac Quest single-reflection diamond attenuated total reflectance (ATR) accessory. Spectral analysis was carried out by using the OMNIC software package. Diphosphinoboranes **1** and **2** were synthesized via procedure described in.^11^

Diffraction data of **1a**, **1b**, **1d**, **2c**, and **2d** were collected on a STOE diffractometer (STOE & Cie GmbH, Darmstadt, Germany) equipped with an image plate detector system IPDS 2T using Cu Kα (λ = 1.54186 Å) radiation and a graphite monochromator. Good quality single-crystal specimens of **1a**, **1b**, **1d**, **2c**, and **2d** were manually selected for the X-ray diffraction experiments. The investigated crystal was monitored via thermostat in a nitrogen stream at 120 K using CryoStream-800 device (Oxford CryoSystem, UK) during the entire experiment. The structures of **1a**, **1b**, **1d**, **2c**, and **2d** were solved with the SHELXS or SHELXT^48^ structure solution programs run under Olex2^49^ using Direct Methods or Intrinsic Phasing and refined with the ShelXL^50^ refinement package. Non-hydrogen atoms were refined with anisotropic displacement parameters. Positions of the C–H hydrogen atoms were calculated geometrically and taken into account with isotropic temperature factors and refined as constrained using standard riding model.

Crystallographic data for all structures reported in this paper have been deposited with the Cambridge Crystallographic Data Centre as supplementary publication No. CCDC 2013366 (**1a**), 2013367 (**1b**), 2013368 (**1d**), 2013369 (**2c**), and 2013370 (**2d**). Copies of the data can be obtained free of charge on application to CCDC, 12 Union Road, Cambridge CB2 1EZ, UK (Fax: (+44) 1223–336–033; E mail: deposit@ccdc.cam.ac.uk).

### 4.2 Preparation of **1a**

A deep orange solution of **1** (189 mg, 0.5 mmol) in toluene (4 mL) was slowly frozen in a liquid nitrogen bath, evacuated to 0.01 Torr, and backfilled with H_2_ (1 atm). The solution was allowed to warm to room temperature and stirred for 14 days yielding a white precipitate. ^31^P{^1^H} (CDCl_3_) NMR of the colorless reaction mixture revealed complete conversion of **1** into **1a** and *t*-Bu_2_PH (1:1 molar ratio). The solvent and *t*-Bu_2_PH were evaporated and the residue was dried under vacuum (0.01 Torr) giving **1a** as a white, air- and moisture-stable solid. Yield 98% (115 mg, 0.245 mmol). X-ray quality crystals (colorless blocks) were grown from a concentrated CH_2_Cl_2_ solution at −20 °C.

**NMR**: ^31^P{^1^H} NMR (CDCl_3_) δ 27.7 (broad s). ^11^B NMR (CDCl_3_) δ −15.8 (broad m). ^1^H NMR (CDCl_3_) δ 7.49 (m, 2H, *o*-C*H*), 7.14 (m, 2H, *m*-C*H*), 7.04 (m, 1H, *p*-C*H*), 3.88 (broad m, ^1^*J*_BH_ = 105.2 Hz, 2H, B*H*), 1.42 (*pseudo*-t, *N* = 6.2 Hz, 36H, CC*H*_3_).* ^13^C{^1^H} NMR (CDCl_3_) δ 133.9 (*pseudo*-t, *N* = 12.7 Hz, *ortho*-CH),* 127.1 (s, *meta*-CH), 124.6 (s, *para*-CH), 36.3 (*pseudo*-t, *N* = 7.3 Hz, *C*(CH_3_)_3_),* 31.7 (s, CH_3_). Aromatic *ipso*-C atom directly bound with boron atom was not detected in the ^13^C{^1^H}. *Virtual coupling of P atoms with H or C atoms, respectively.

Elemental analysis Calcd. for C_28_H_48_B_2_P_2_: C, 71.82; H, 10.33. Found: C, 71.49; H, 10.10.

IR (solid) υ̃ = 3074, 3044, 3016, 2999, 2969, 2898, 2867, **2352 (B–H)**, 1587, 1485, 1460, 1428, 1390, 1363, 1175, 979, 900, 816, 727, 701, 454, 437 cm^–1^.

### 4.3 Preparation of **1b**

A deep orange solution of **1** (189 mg, 0.5 mmol) in toluene (4 mL) was slowly frozen in a liquid nitrogen bath, evacuated to 0.01 Torr, and backfilled with CO_2_ (1 atm). The solution was allowed to warm to room temperature and stirred for 16 h. ^31^P{^1^H} of the yellow reaction mixture revealed complete conversion of **1** into **1b** and *t*-Bu_2_PC(O)P*t*-Bu_2_. The solvent was evaporated, and the residue was dried under vacuum (0.01 Torr) giving a 1:1 molar ratio mixture of *t*-Bu_2_PC(O)P*t*-Bu_2_ and **1b** as a yellowish solid. Yield 95%: 209 mg, assuming 1:1 molar ratio of products: 0.238 mmol of **1b** (135 mg) and 0.238 mmol of *t*-Bu_2_PC(O)P*t*-Bu_2_ (74 mg). The solid was dissolved in 1 cm^3^ of pentane and left at −20 °C to afford mixture of yellow (*t*-Bu_2_PC(O)P*t*-Bu_2_) and colorless (**1b**) X-ray quality crystals which were dried in vacuum.

**NMR**: ^31^P{^1^H} NMR (toluene-*d*_8_) δ 46.4 (s). ^11^B NMR (toluene-*d*_8_) δ 6.7 (broad s). ^1^H NMR (toluene-*d*_8_) δ 8.19 (m, 2H, *o*-C*H*), 7.44 (m, 2H, *m*-C*H*), 7.33 (m, 1H, *p*-C*H*), 1.17 (d, ^3^*J*_PH_ = 12.3, 36H, C(C*H*_3_)_3_). ^13^C{^1^H} NMR (toluene-*d*_8_) δ 197.7 (d, ^1^*J*_CP_ = 45.4 Hz, *C*=O), 132.0 (s, *ortho*-CH), 128.1 (s, *para*-CH), 127.5 (s, *meta*-CH), 34.0 (d, ^1^*J*_CP_ = 21.8 Hz, *C*(CH_3_)_3_), 29.6 (d, ^2^*J*_CP_ = 12.7 Hz, C*(CH*_3_*)*_3_). The aromatic *ipso*-C atom directly bound with boron atom was not detected in the ^13^C{^1^H} spectra.

### 4.4 Preparation of **2b**

A solution of **2** (253 mg, 0.5 mmol) in toluene (4 mL) was slowly frozen in a liquid nitrogen bath, evacuated to 0.01 Torr, and backfilled with CO_2_ (1 atm). The solution was allowed to warm to room temperature and stirred for 24 h. ^31^P{^1^H} of the colorless reaction mixture revealed complete conversion of **2** into **2b**. The solvent was evaporated and the residue was dried under vacuum (0.01 Torr) giving **2b** as a colorless oil. Yield 95% (291 mg, 0.490 mmol).

**NMR**: ^31^P{^1^H} NMR (C_6_D_6_) δ 13.6 (s). ^11^B NMR (C_6_D_6_) δ 22.2 (broad s). ^1^H NMR (C_6_D_6_) δ 3.44 (sept, ^3^*J*_HH_ = 6.8 Hz, 2H, C*H*CH_3_), 2.24 (overlapped m, 4H, C*H*_2_), 2.16 (overlapped m, 4H, C*H*CH_2_), 1.91 (m, 4H, C*H*_2_), 1.83–1.68 (overlapped m, 9H, C*H*_2_), 1.66–1.50 (overlapped m, 13H, C*H*_2_), 1.31–1.20 (overlapped m, 10H, C*H*_2_), 1.10 (d, ^3^*J*_HH_ = 6.8 Hz, 12H, CHC*H*_3_). ^13^C{^1^H} NMR (C_6_D_6_) δ 179.9 (d, ^1^*J*_CP_ = 26.3 Hz, C = O), 45.1 (s, *CH*CH_3_), 32.6 (d, ^1^*J*_CP_ = 12.7 Hz, *CH*CH_2_), 30.9 (d, ^2^*J*_CP_ = 10.9 Hz, CH_2_), 29.6 (d, ^2^*J*_CP_ = 10.9 Hz, CH_2_), 27.4 (d, ^3^*J*_CP_ = 10.0 Hz, CH_2_), 27.2 (d, ^3^*J*_CP_ = 9.1 Hz, CH_2_), 26.2 (broad s, CH_2_), 22.4 (s, CH*CH*_3_).

IR (oil) υ̃ = 2920, 2849, 2335, 2261, **1674 (C**=**O)**, 1625, 1494, 1446, 1382, 1338, 1269, 1208, 1176, 1143, 1112, 1000, 887, 851 cm^–1^

### 4.5 Preparation of **1d**

To a solution of **1** (189 mg, 0.5 mmol) in toluene (4 mL) was added dropwise at room temperature PhNCO (179 mg, 1.5 mmol). The solution was stirred for 24 h. ^31^P{^1^H} of the colorless reaction mixture revealed complete conversion of **1** to **1d**. The solvent was evaporated, and the residue was dried under vacuum (0.01 Torr) giving **1d** as a white solid. Yield 99% (364 mg, 0.495 mmol). X-ray quality crystals (yellowish blocks) were grown from a toluene solution at −20 °C.

**NMR**: ^31^P{^1^H} NMR (toluene-*d*_8_) δ 41.9 (s, P1), 29.1 (s, P2). ^11^B NMR (toluene-*d*_8_) δ 6.2 (broad s). ^1^H NMR (toluene-*d*_8_) δ 8.18 (m, 2H, *o*-C*H*, B–Ph), 7.69 (m, 2H, *o*-C*H*, PhNCO), 7.45 (m, 2H, *m*-C*H*, B–Ph), 7.32 (overlapped m, 1H, *m*-C*H*, B–Ph), 7.30 (overlapped m, 2H, *o*-C*H*, PhNCO), 7.12–7.07 (overlapped m, 5H, Ar–C*H*, PhNCO), 7.04–6.92 (overlapped m, 6H, Ar–C*H*, PhNCO), 1.51 (d, ^3^*J*_PH_ = 11.4, 9H, C(C*H*_3_)_3,_*t*-Bu_2_P2), 1.25 (d, ^3^*J*_PH_ = 12.3, 9H, C(C*H*_3_)_3,_*t*-Bu_2_P1), 0.96 (d, ^3^*J*_PH_ = 12.1, 9H, C(C*H*_3_)_3,_*t*-Bu_2_P2), 0.82 (d, ^3^*J*_PH_ = 12.6, 9H, C(C*H*_3_)_3_, *t*-Bu_2_P1). ^13^C{^1^H} NMR (toluene-*d*_8_) δ 187.8 (d, ^1^*J*_CP_ = 68.1 Hz, N=C–O), 167.1 (d, ^1^*J*_CP_ = 60.0 Hz, N=C–O), 150.2 (d, ^3^*J*_CP_ = 12.7 Hz, C=O), 149.8 (s, *ipso*-C, PhNCO), 140.5 (s, *ipso*-C, PhNCO), 137.7 (d, ^3^*J*_CP_ = 5.4 Hz, *ipso*-C, PhNCO), 132.8 (s, *ortho*-CH, B–Ph), 129.0 (s, *ortho*-CH, PhNCO), 128.6 (s, *ortho*-CH, PhNCO), 128.4 (s, *ortho*-CH, PhNCO), 128.1 (s, *m*-/*p*-CH, PhNCO), 128.0 (s, *m*-/*p*-CH, PhNCO), 127.9 (s, *para*-CH, B–Ph), 127.8 (s, *meta*-CH, B–Ph), 125.6 (s, *m*-/*p*-CH, PhNCO), 122.8 (s, *m*-/*p*-CH, PhNCO), 122.7 (s, *m*-/*p*-CH, PhNCO), 122.1 (s, *m*-/*p*-CH, PhNCO), 35.2 (d, ^1^*J*_CP_ = 27.2 Hz, *C*(CH_3_)_3_, *t*-Bu_2_P1), 34.7 (d, ^1^*J*_CP_ = 28.2 Hz, *C*(CH_3_)_3_, *t*-Bu_2_P1), 32.8 (d, ^1^*J*_CP_ = 27.2 Hz, *C*(CH_3_)_3_, *t*-Bu_2_P2), 32.1 (d, ^1^*J*_CP_ = 26.3 Hz, *C*(CH_3_)_3_, *t*-Bu_2_P2), 31.0 (d, ^2^*J*_CP_ = 15.4 Hz, C*(CH*_3_*)*_3_, *t*-Bu_2_P2), 30.4 (d, ^2^*J*_CP_ = 14.5 Hz, C*(CH*_3_*)*_3_, *t*-Bu_2_P1), 30.0 (d, ^2^*J*_CP_ = 16.3 Hz, C*(CH*_3_*)*_3_, *t*-Bu_2_P2), 29.2 (d, ^2^*J*_CP_ = 15.4 Hz, C*(CH*_3_*)*_3_, *t*-Bu_2_P1). The aromatic *ipso*-C atom directly bound with boron atom was not detected in the ^13^C{^1^H}.

IR (solid) υ̃ = 2967, 2942, 2892, 2861, **1729 (C=O)**, **1587 (C**=**N)**, **1505 (OCN)**, 1492, 1470, 1432, 1381, 1366, 1318, 1252, 1228, 1173, 1158, 1101, 1070, 1005, 924, 911, 901, 764, 750, 739, 691, 634, 609 cm^–1^.

Elemental Analysis calcd for C_43_H_56_BN_3_O_3_P_2_: C, 70.20; H, 7.67; N, 5.71. Found: C, 70.31; H, 7.624; N, 5.70.

### 4.6 Preparation of **2c**

To a solution of **2** (253 mg, 0.5 mmol) in toluene (4 mL) was added dropwise at room temperature PhNCO (119 mg, 1.0 mmol). The solution was stirred for 24 h. ^31^P{^1^H} of the colorless reaction mixture revealed complete conversion of **2** to **2c**. The solvent was evaporated, and the residue was dried under vacuum (0.01 Torr) giving mixture of **2c** and **2c′** as a white solid. Yield 98% (361 mg, 0.485 mmol). X-ray quality crystals of pure **2c** (colorless blocks) were grown from a concentrated toluene solution at −20 °C. Yield 70% (260 mg, 0.350 mmol).

**NMR**: ^31^P{^1^H} NMR (toluene-*d*_8_, 298 K) δ 7.6 (s, P1), 2.5 (s, P2). ^11^B NMR (toluene-*d*_8_, 298 K) δ 24.9 (broad s). ^1^H NMR (toluene-*d*_8_, 298 K) δ 7.58 (broad m, 2H, Ar–C*H*), 7.24 (overlapped m, 3H, Ar–C*H*), 7.03 (m, 2H, Ar–C*H*), 6.85 (m, 1H, Ar–C*H*), 6.15 (broad m, 2H, Ar–C*H*), 4.03 (broad m, C*H*CH_3_), 3.78 (broad m, C*H*CH_3_), 2.97 (broad m, 1H, C*H*CH_2_), 2.46 (broad m, 3H, C*H*CH_2_), 2.31–2.00 (broad m, overlapped, 4H,* C*H*_2_) 1.96–1.54 (overlapped m, 18H, C*H*_2_) 1.53–0.84 (overlapped m, 30H, C*H*_2_ and C*H*_3_). *Signals in this range are overlapped by toluene-*d*_8_ residual signal and were not integrated. ^13^C{^1^H} NMR (toluene-*d*_8_, 248 K) δ 183.4 (d, ^1^*J*_CP_ = 31.8 Hz, C=O), 161.7 (d, ^1^*J*_CP_ = 22.7 Hz, C=N), 149.8 (s, *ipso*-C), 137.4 (s, *ipso*-C, overlapped), 129.0 (s, *Ar*–C), 128.6 (s, *Ar*–C), 128.4 (s, *Ar*–C), 128.1 (s, *Ar*–C), 125.2 (s, *Ar*–C), 122.5 (s, *Ar*–C), 120.3 (s, *Ar*–C), 48.0 (s, *CH*CH_3_), 43.5 (s, *CH*CH_3_), 34.2 (broad m, *CH*CH_2_), 34.0 (broad m *CH*CH_2_), 31.4 (broad m, CH_2_), 31.3 (broad m, CH_2_), 30.6 (broad m, CH_2_), 30.0 (broad m, CH_2_), 29.8 (broad m, CH_2_), 29.1 (broad m, CH_2_), 28.3 (broad m, CH_2_), 27.9 (broad m, CH_2_), 27.5 (broad m, CH_2_), 26.9 (broad m, CH_2_), 26.4 (broad m, CH_2_), 23.7 (broad m, CH*CH*_3_), 21.8 (broad m, CH*CH*_3_).

IR (solid) υ̃ = 2919, 2848, **1629 (C**=**N)**, **1612 (C**=**O)**, **1589(C**=**O/N)**, 1488, 1479, 1446, 1364, 1324, 1311, 1263, 1232, 1206, 1154, 1129, 1092, 1016, 1000, 885, 852, 736, 712, 693 cm^–1^.

Elemental analysis calcd for C_44_H_68_BN_3_O_2_P_2_: C, 71.05; H, 9.21; N, 5.65. Found: C, 70.89; H, 9.169; N, 5.60.

### 4.7 Preparation of **2d**

To a solution of **2** (253 mg, 0.5 mmol) in toluene (4 mL) an excess of PhNCO (357 mg, 3.0 mmol) was added dropwise at room temperature. The solution was stirred for 4 days. ^31^P{^1^H} of the yellow reaction mixture revealed complete conversion of **2** to equilibrium mixture of **2c** and **2d** in 3:5 molar ratio. The reaction mixture was concentrated and left at −20 °C to afford X-ray quality crystals of **2d**. The solution was separated and the crystalline residue was dried under vacuum (0.01 Torr) giving **2d** as analytically pure yellow solid. Yield 47% (203 mg, 0.235 mmol).

**NMR**: ^31^P{^1^H} NMR (toluene-*d*_8_, 298 K) δ 18.2 (s, P1), 2.0 (s, P2). ^11^B NMR (toluene-*d*_8_, 298 K) δ 6.6 (broad s). ^1^H NMR (toluene-*d*_8_, 248 K) δ 7.92 (m, 2H, *o*-C*H*), 7.42–7.34 (overlapped m, 3H, Ar–C*H*), 7.27 (m, 2H, Ar–C*H*), 7.17–7.11 (overlapped m, 2H, Ar–C*H*), 7.08–7.05 (overlapped m, 1H, Ar–C*H*), 7.03–6.96 (overlapped m, 3H, Ar–C*H*), 6.93–6.85 (overlapped m, 2H, Ar–C*H*), 3.53 (broad m, 1H, C*H*), 3.29 (broad m, 1H, C*H*), 2.87 (broad m, 1H, C*H*CH_2_), 2.54 (broad m, 1H, C*H*CH_2_), 2.30 (broad m, 1H, C*H*CH_2_), 2.22 (broad m, 1H, C*H*CH_2_), 1.83–1.68 (m, overlapped, 52H, C*H*_2_ and C*H*_3_). ^13^C{^1^H} NMR (toluene-*d*_8_, 248 K) δ 188.6 (d, ^1^*J*_CP_ = 50.0 Hz, N=C–O), 166.7 (d, ^1^*J*_CP_ = 46.3 Hz, N=C–O), 153.1 (s, *ipso*-C), 150.4 (d, ^3^*J*_CP_ = 11.8 Hz, C=O), 142.3 (s, *ipso*-C), 136.3 (s, *ipso*-C), 131.4 (s, *Ar*–C), 130.6 (s, *Ar*–C), 129.2 (s, *Ar*–C), 129.0 (s, *Ar*–C), 128.8 (s, *Ar*–C), 128.8 (s, *Ar*–C), 128.7 (s, *Ar*–C), 128.1 (s, *Ar*–C), 127.9 (s, *Ar*–C), 127.8 (s, *Ar*–C), 127.4 (s, *Ar*–C), 125.2 (s, *Ar*–C), 124.4 (s, *Ar*–C), 122.7 (s, *Ar*–C), 122.4 (s, *Ar*–C), 46.3 (s, *CH*CH_3_), 43.0 (s, *CH*CH_3_), 34.5 (d, ^1^*J*_CP_ = 18.2 Hz, *CH*CH_2_), 34.1 (d, ^1^*J*_CP_ = 19.1 Hz, *CH*CH_2_), 33.4 (d, ^1^*J*_CP_ = 17.3 Hz, *CH*CH_2_), 32.9 (d, ^1^*J*_CP_ = 17.3 Hz, *CH*CH_2_), 30.9 (d, ^2^*J*_CP_ = 10.9 Hz, CH_2_), 29.6 (d, ^2^*J*_CP_ = 10.9 Hz, CH_2_), 27.4 (d, ^3^*J*_CP_ = 10.0 Hz, CH_2_), 27.2 (d, ^3^*J*_CP_ = 9.1 Hz, CH_2_), 31.9–25.6 (broad multiplets, CH_2_), 24.8 (broad m, CH*CH*_3_), 22.7 (broad m, CH*CH*_3_).

IR (solid) υ̃ = 2922, 2851, **1719 (C**=**O)**, **1593 (C**=**N)**, **1532 (OCN)**, 1492, 1446, 1388, 1300, 1239, 1179, 1165, 1127, 1079, 1060, 1027, 939, 908, 809, 762, 694, 603 cm^–1^.

Elemental analysis calcd for C_51_H_73_BN_4_O_3_P_2_: C, 70.99; H, 8.53; N, 6.49. Found: C, 70.90; H, 8.435; N, 6.43.

## Supporting Information Available

Crystallographic details, spectroscopic details, and computational details (PDF)

## Accession Codes

CCDC 2013366–2013370 contain the supplementary crystallographic data for this paper. These data can be obtained free of charge via www.ccdc.cam.ac.uk/data_request/cif, or by emailing data_request@ccdc.cam.ac.uk, or by contacting The Cambridge Crystallographic Data Centre, 12 Union Road, Cambridge CB2 1EZ, UK; fax: +44 1223 336033.
