# Supplementary material for: Diphosphinoboranes as Intramolecular Frustrated Lewis Pairs: P–B–P Bond Systems for the Activation of Dihydrogen, Carbon Dioxide, and Phenyl Isocyanate
Source: Inorg Chem. 2021 Mar 4;60(6):3794–806. doi: 10.1021/acs.inorgchem.0c03563 (PMC8041279; doi:10.1021/acs.inorgchem.0c03563)
Supplement: Supplementary file 1 — ic0c03563_si_001.pdf [file ic0c03563_si_001.pdf]

Supplementary Material for

Diphosphinoboranes as Intramolecular Frustrated Lewis Pairs: P-B-P Bond Systems for the Activation of Dihydrogen, Carbon Dioxide, and Phenyl Isocyanate

Natalia Szynkiewicz, Anna Ordyszewska, Jarosław Chojnacki

*and Rafał Grubba**

*Department of Inorganic Chemistry, Faculty of Chemistry,

Gdańsk University of Technology, G. Narutowicza St. 11/12. PL-80-233, Gdansk, Poland.

CONTENTS:

X-ray structures analysis 3

Single crystal X-ray structure analysis of 1a 5

Single crystal X-ray structure analysis of 1b 6

Single crystal X-ray structure analysis of 1d 7

Single crystal X-ray structure analysis of 2c 8

Single crystal X-ray structure analysis of 2d 9

Spectroscopic data 10

NMR spectra of isolated compounds 10

NMR spectra of 1a 10

NMR spectra of 1b 13

NMR spectra of 1d 15

NMR spectra of 2b 18

NMR spectra of 2c 20

NMR spectra of 2d 23

IR spectra of isolated compounds 26

DFT calculations 28

General methods 28

A Gibbs free-energy profiles 33

Optimized structures, Hirshfeld atomic charges and Cartesian coordinates 35

Substrates 35

Formation of 1a 42

Formation of 1b 62

Formation of 1d 114

Formation of 2b 155

Formation of 2c and 2d 167

References 231

X-ray structures analysis

TABLE S1. CRYSTAL DATA AND STRUCTURE REFINEMENT FOR **1A**, **1B** AND **1D**

	**1a**	**1b**	**1d**
CCDC	2013366	2013367	2013368
Empirical formula	$2(C_{14}H_{24}BP)$	$C_{30}H_{46}B_2O_5P_2$	$C_{43}H_{57}BN_3O_3P_2$
M_r [g mol^{-1}]	468.22	570.23	736.66
Crystal system	Triclinic	Monoclinic	Triclinic
Space group	P-1	$P2_1$	P-1
a [Å]	10.2820 (15)	11.292 (5)	11.1491 (8)
b [Å]	11.3478 (17)	19.982 (7)	11.8587 (7)
c [Å]	12.696 (2)	14.287 (5)	16.1221 (11)
α [°]	88.628 (13)	90	81.595 (5)
β [°]	68.234 (13)	89.97 (3)	89.184 (5)
γ [°]	88.270 (12)	90	77.874 (5)
V [Å^3]	1375.0 (4)	3224 (2)	2061.3 (2)
Z	2	4	2
Calculated density [Mg m^{-3}]	1.131	1.175	1.187
T [K]	120	120	120
μ [mm^{-1}]	1.51	1.50	1.27
Crystal size/mm^3	0.09 × 0.03 × 0.03	0.35 × 0.23 × 0.09	0.31 × 0.14 × 0.11
λ [Å]	1.54186 (CuKα)	1.54186 (CuKα)	1.54186 (CuKα)
F(000)	512	1224	790
S	1.42	1.08	1.02
R_{int}	0.121	0.088	0.015
No. of measured, independent, observed [I > 2σ(I)] reflections	10613, 4466, 3516	21171, 9807, 8982	18560, 6536, 6233
Final R indices [I>2σ(I)]	R_1 = 0.117	R_1 = 0.098	R_1 = 0.032
	wR_2 = 0.296	wR_2 = 0.254	wR_2 = 0.081
R indices (all data)	R_1 = 0.134	R_1 = 0.107	R_1 = 0.034
	wR_2 = 0.337	wR_2 = 0.271	wR_2 = 0.082
Largest diff. peak/hole / e Å^{-3}	1.02/-1.01	0.73/-0.46	0.28/-0.24

TABLE S2. CRYSTAL DATA AND STRUCTURE REFINEMENT FOR **2C** AND **2D**

	**2c**	**2d**
CCDC	2013369	2013370
Empirical formula	$C_{44}H_{68}BN_3O_2P_2$	$C_{51}H_{73}BN_4O_3P_2$
M_r [g mol^{-1}]	743.76	862.87
Crystal system	Orthorhombic	Triclinic
Space group	$P2_12_12$	P-1
a [Å]	18.5006 (10)	11.6800 (3)
b [Å]	28.8906 (14)	17.0835 (5)
c [Å]	9.7625 (4)	18.6905 (6)
α [°]	90	102.427 (2)
β [°]	90	105.175 (2)
γ [°]	90	109.863 (2)
V [Å^3]	5218.0 (4)	3189.56 (17)
Z	4	2
Calculated density [Mg m^{-3}]	0.947	0.898
T [K]	120	120
μ [mm^{-1}]	0.99	0.88
Crystal size/mm^3	0.36 × 0.06 × 0.04	0.25 × 0.09 × 0.08
λ [Å]	1.54186 (CuKα)	1.54186 (CuKα)
F(000)	1616	932
S	1.06	1.06
R_{int}	0.077	0.026
No. of measured, independent, observed [I > 2σ(I)] reflections	30822, 9203, 7390	30596, 10439, 8564
Final R indices [I>2σ(I)]	R_1 = 0.074	R_1 = 0.052
	wR_2 = 0.196	wR_2 = 0.158
R indices (all data)	R_1 = 0.094	R_1 = 0.061
	wR_2 = 0.221	wR_2 = 0.166
Largest diff. peak/hole / e Å^{-3}	0.35/-0.47	0.49/-0.33

Single crystal X-ray structure analysis of 1a

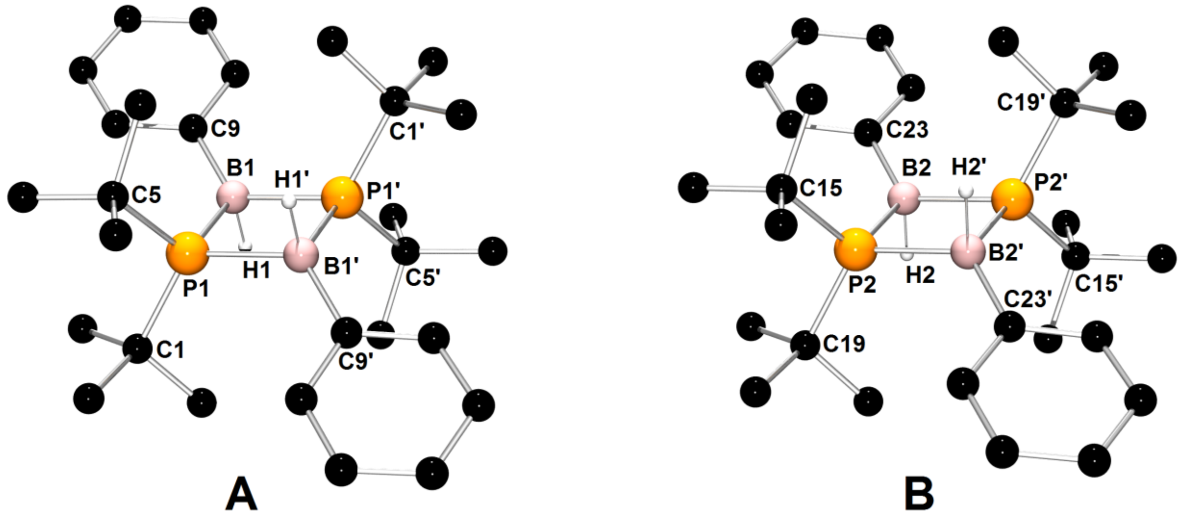

FIG. S1. MOLECULAR STRUCTURE OF **1A** (A) AND (B)

TABLE S3. SELECTED STRUCTURAL PARAMETERS OF **1A_A**

Bond lengths [Å]		Bond angles [°]		Dihedrals [°]	
P1-B1	2.018(6)	B1-P1-B1'	88.6(2)	C1-P1-B1-C9	102.4(4)
P1-B1'	2.021(5)	H1-B1-C9	112(3)	C5-P1-B1-H1	-146(3)
B1-H1	1.13(5)	C1-P1-C5	108.3(2)		
P1-C1	1.902(4)	C5-P1-B1	121.2(2)		
P1-C5	1.903(5)				
B1-C9	1.600(6)				

TABLE S4. SELECTED STRUCTURAL PARAMETERS OF **1A_B**

Bond lengths [Å]		Bond angles [°]		Dihedrals [°]	
P2-B2	2.023(5)	B2-P2-B2'	88.3(2)	C15-P2-B2-H2	-154(2)
P2-B2'	2.020(4)	H2-B2-C23	112(2)	C19-P2-B2-C23	-99.4(4)
B2-H2	1.15(4)	C15-P2-C19	108.5(2)		
P2-C15	1.902(5)	C19-P2-B2	108.9(2)		
P2-C19	1.908(4)				
B2-C23	1.589(6)				

Single crystal X-ray structure analysis of 1b

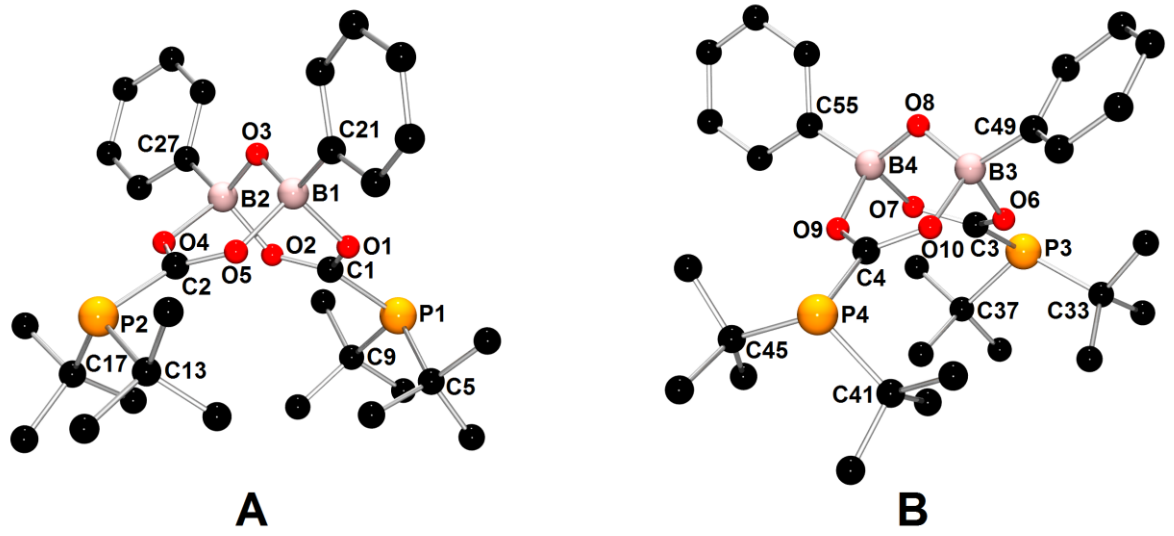

FIG. S2. MOLECULAR STRUCTURE OF **1B** (A) AND (B)

TABLE S5. SELECTED STRUCTURAL PARAMETERS OF **1B** (A)

**Bond lengths [Å]**				**Bond angles [°]**			
B1-O1	1.59(2)	O5-C2	94.47(4)	B1-O3-B2	113(1)	P1-C1-O1	115.4(9)
B1-O3	1.6384(9)	P1-C1	105.15(5)	O1-B1-O3	109(1)	P2-C2-O5	120(1)
B1-O5	1.478(1)	P1-C5	111.36(6)	O3-B1-O5	108(1)	P1-C1-O2	121.0(9)
B2-O2	1.437(2)	P1-C9	111.55(5)	O1-B1-O5	100.5(9)	P2-C2-O4	117(1)
B2-O3	1.404(2)	P2-C2	105.81(6)	O2-B2-O3	111(1)		
B2-O4	1.449(2)	P2-C13	96.41(4)	O2-B2-O4	101.2(9)		
O1-C1	1.898(1)	P2-C17	115.6(1)	O3-B2-O4	108(1)		
O2-C1	1.885(1)	B1-C21	118.8(1)	O1-C1-O2	123(1)		
O4-C2	1.484(2)	B2-C27	125.5(1)	O4-C2-O5	123(1)		

TABLE S6. SELECTED STRUCTURAL PARAMETERS OF **1B** (B)

**Bond lengths [Å]**				**Bond angles [°]**			
B3-O6	1.62(2)	O10-C4	1.30(2)	B3-O8-B4	114(1)	P3-C3-O6	122(1)
B3-O10	1.56(2)	P3-C3	1.83(1)	O6-B3-O8	107(1)	P4-C4-O9	123(1)
B3-O8	1.41(2)	P3-C33	1.87(1)	O8-B3-O10	109(1)	P3-C3-O7	116.7(9)
B4-O7	1.58(2)	P3-C37	1.92(1)	O6-B3-O10	101(1)	P4-C4-O10	113.5(9)
B4-O8	1.42(2)	P4-C4	1.84(1)	O7-B4-O8	108(1)		
B4-O9	1.59(2)	P4-C41	1.89(2)	O8-B4-O9	109(1)		
O6-C3	1.26(2)	P4-C45	1.88(2)	O7-B4-O9	99.8(9)		
O7-C3	1.29(2)	B3-C49	1.56(2)	O6-C3-O7	122(1)		
O9-C4	1.27(2)	B4-C55	1.57(2)	O9-C4-O10	123(1)		

Single crystal X-ray structure analysis of 1d

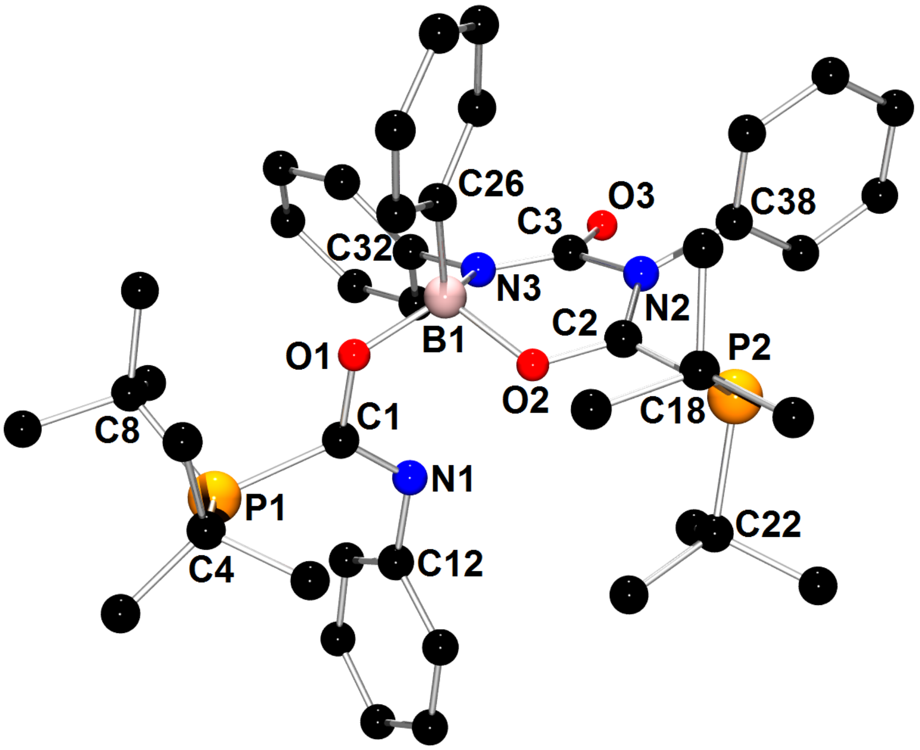

FIG. S3. MOLECULAR STRUCTURE OF **1D**

TABLE S7. SELECTED STRUCTURAL PARAMETERS OF **1D**

**Bond lengths [Å]**		**Bond angles [°]**		**Dihedrals [°]**	
B1-O1	1.451(2)	O1-B1-O2	107.5(1)	N1-C1-O1-B1	3.4(2)
B1-O2	1.575(2)	O1-B1-N3	114.2(1)	P1-C1-O1-B1	-179.5(1)
B1-N3	1.547(2)	O2-B1-N3	104.3(1)	P1-C1-N1-C12	3.7(2)
O1-C1	1.352(1)	B1-N3-C3	121.7(1)	O2-C2-P2-C22	59.7(1)
O2-C2	1.274(2)	N2-C3-N3	113.9(1)		
O3-C3	1.214(2)	N2-C3-O3	116.8(1)		
N1-C1	1.274(2)	C2-N2-C3	122.5(1)		
N2-C2	1.337(2)	O2-C2-N2	119.6(1)		
N2-C3	1.483(2)	P2-C2-O2	122.0(1)		
N3-C3	1.336(2)	P2-C2-N2	118.3(1)		
P1-C1	1.882(1)	B1-O1-C1	125.3(1)		
P2-C2	1.879(2)	O1-C1-N1	120.8(1)		
B1-C26	1.614(2)	P1-C1-N1	123.7(1)		
N1-C12	1.420(2)	P1-C1-O1	115.40(9)		
N2-C38	1.459(2)				
N3-C32	1.446(2)				
P1-C4	1.894(2)				
P1-C8	1.892(2)				
P2-C18	1.889(2)				
P2-C22	1.898(1)				

Single crystal X-ray structure analysis of 2c

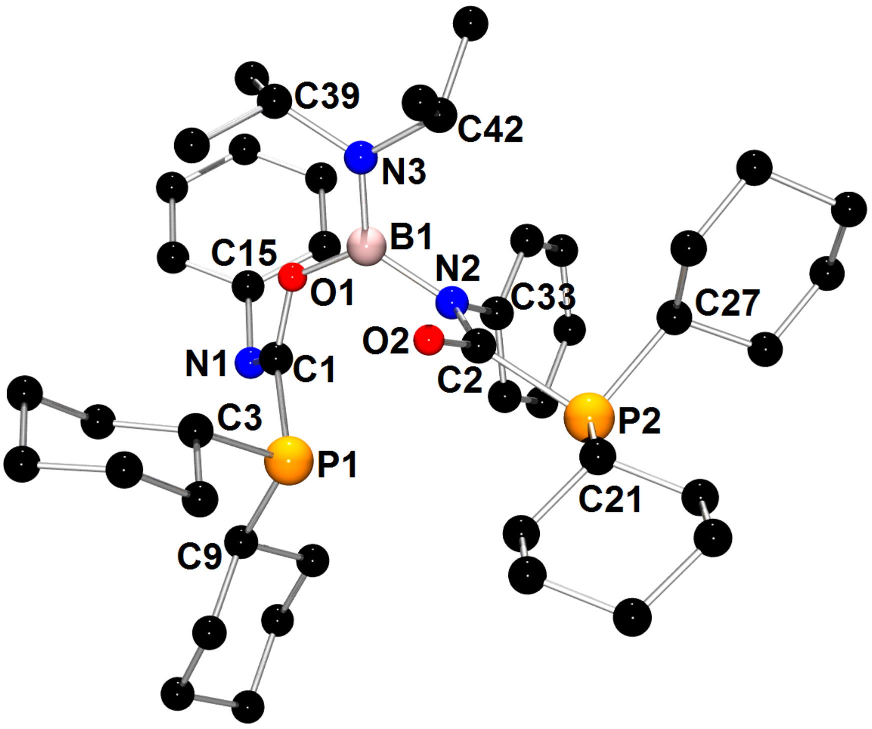

FIG. S4. MOLECULAR STRUCTURE OF **2C**

TABLE S8. SELECTED STRUCTURAL PARAMETERS OF **2C**

**Bond lengths [Å]**		**Bond angles [°]**		**Dihedrals [°]**	
B1-O1	1.389(7)	B1-N2-C2	118.1(5)	B1-N2-C2-O2	2.9(8)
B1-N2	1.493(8)	B1-O1-C1	130.8(4)	B1-O1-C1-P1	50.3(7)
B1-N3	1.396(7)	B1-N3-C42	121.5(5)	N1-C1-O1-B1	-132.6(6)
O1-C1	1.371(7)	B1-N3-C39	123.1(5)	C1-O1-B1-N2	12.4(8)
O2-C2	1.214(7)	O1-B1-N2	119.2(5)	N2-B1-N3-C42	-5.8(8)
N1-C1	1.278(8)	O1-B1-N3	117.3(5)		
N2-C2	1.378(8)	O1-C1-N1	121.0(5)		
P1-C1	1.862(6)	O2-C2-N2	119.8(5)		
P2-C2	1.882(6)	N2-B1-N3	123.2(5)		
N1-C15	1.424(7)	P1-C1-N1	122.9(4)		
N2-C33	1.433(8)	P1-C1-O1	116.0(4)		
N3-C39	1.487(7)	P2-C2-O2	121.7(4)		
N3-C42	1.474(8)	P2-C2-N2	118.5(4)		
P1-C3	1.883(6)	C39-N3-C42	115.2(5)		
P1-C9	1.835(6)				
P2-C21	1.855(7)				
P2-C27	1.892(8)				

Single crystal X-ray structure analysis of 2d

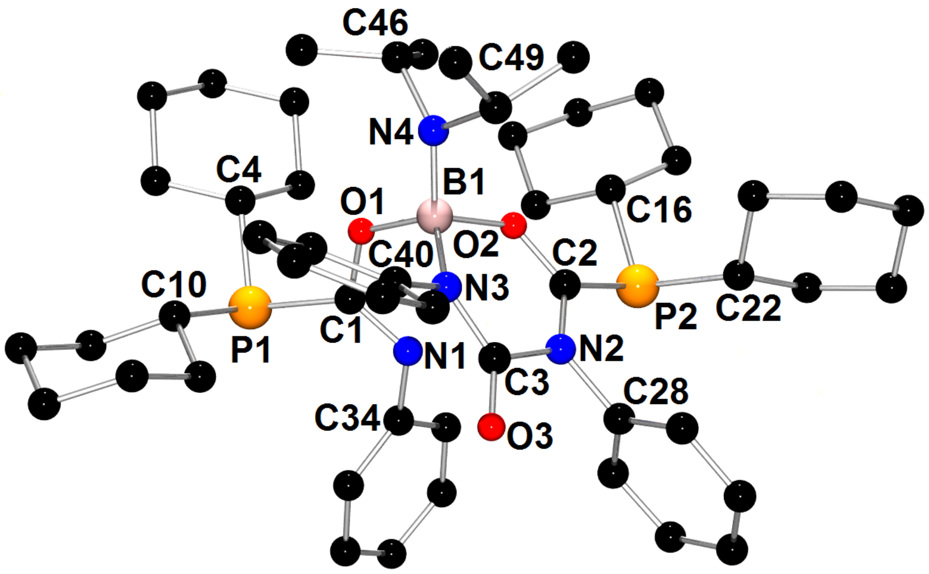

FIG. S5. MOLECULAR STRUCTURE OF **2D**

TABLE S9. SELECTED STRUCTURAL PARAMETERS OF **2D**

Bond lengths [Å]		Bond angles [°]		Dihedrals [°]	
B1-O1	1.488(3)	O1-B1-O2	105.7(2)	B1-O1-C1-N1	-9.8(3)
B1-O2	1.592(3)	O1-B1-N3	112.2(2)	P1-C1-O1-B1	172.6(2)
B1-N3	1.551(2)	O2-B1-N3	104.5(2)		
B1-N4	1.459(3)	B1-O1-C1	124.9(2)		
P1-C1	1.882(2)	B1-O2-C2	126.8(2)		
P2-C2	1.868(3)	B1-N3-C3	125.1(2)		
O1-C1	1.347(3)	O1-C1-N1	120.0(2)		
O2-C2	1.270(3)	O2-C2-N2	120.9(2)		
O3-C3	1.212(2)	O3-C3-N2	116.8(2)		
N1-C1	1.282(3)	O3-C3-N3	128.1(2)		
N2-C3	1.464(3)	P1-C1-O1	116.6(2)		
N2-C2	1.341(2)	P1-C1-N1	123.3(2)		
N3-C3	1.354(3)	P2-C2-O2	117.8(2)		
P1-C4	1.863(2)	P2-C2-N2	121.0(2)		
P1-C10	1.876(3)	N2-C3-N3	115.0(2)		
P2-C16	1.858(2)	C1-N1-C34	122.2(2)		
P2-C22	1.863(2)	C2-N2-C3	123.7(2)		
N1-C34	1.416(3)	C46-N4-C49	113.8(2)		
N2-C28	1.450(3)	B1-N4-C49	118.7(2)		
N3-C40	1.447(3)	B1-N4-C46	124.5(2)		
N4-C46	1.473(3)				
N4-C49	1.474(3)				

Spectroscopic data

NMR spectra of isolated compounds

NMR spectra of 1a

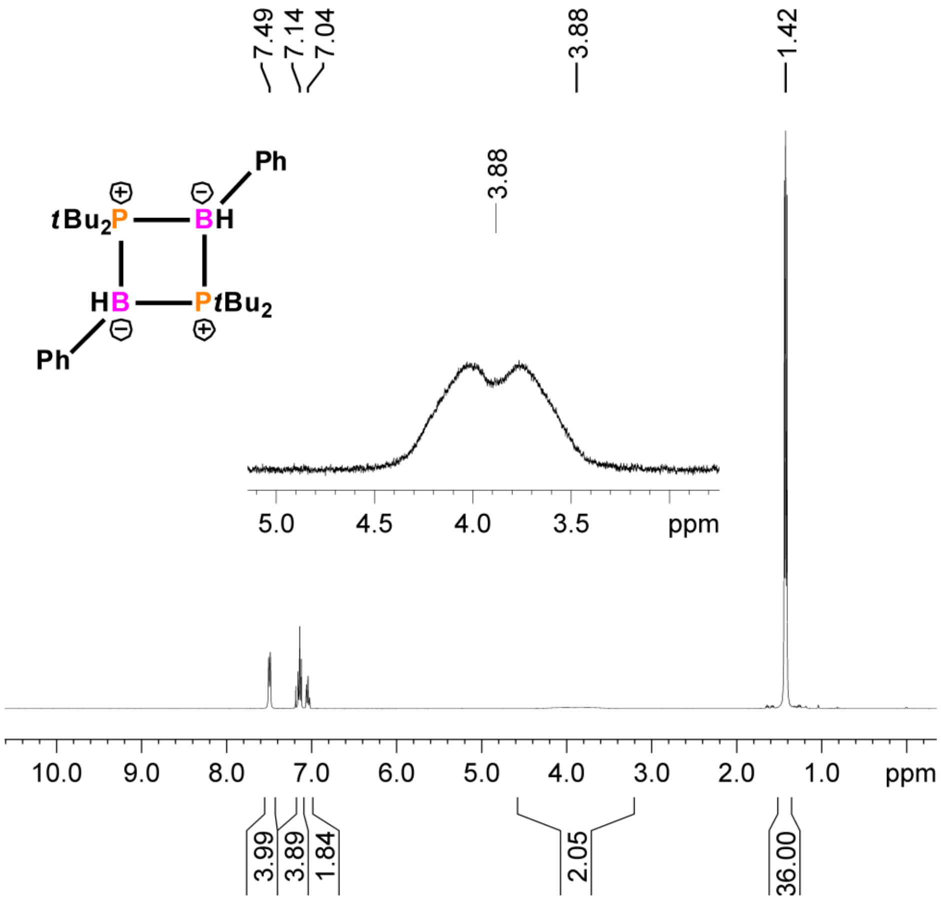

FIG. S6. ^{1}H NMR ($CDCl_3$) SPECTRUM OF **1A**

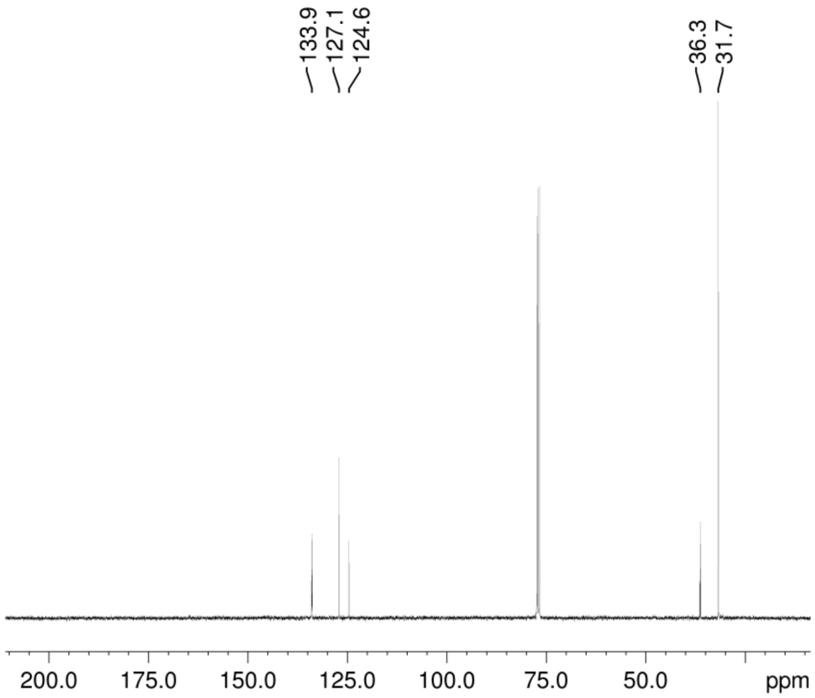

FIG. S7. $^{13}C\{^1H\}$ NMR ($CDCl_3$) SPECTRUM OF **1A**

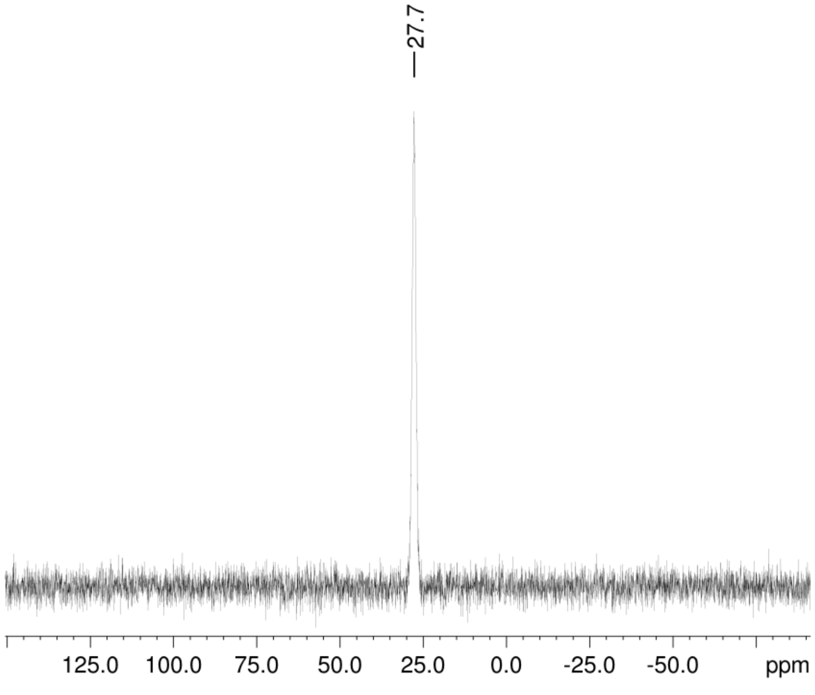

FIG. S8. $^{31}P\{^1H\}$ NMR ($CDCl_3$) SPECTRUM OF **1A**

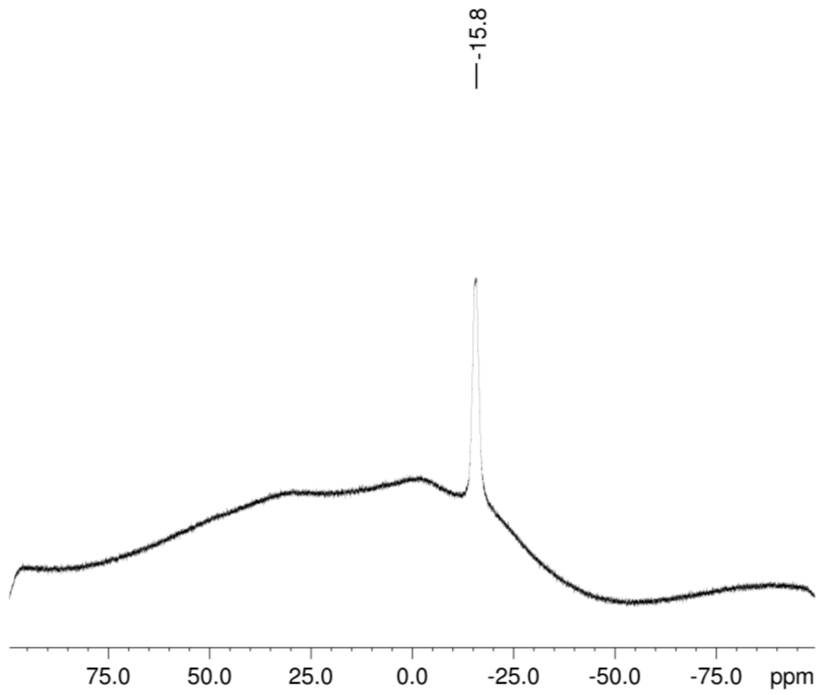

FIG. S9. ^{11}B NMR ($CDCl_3$) SPECTRUM OF **1A**

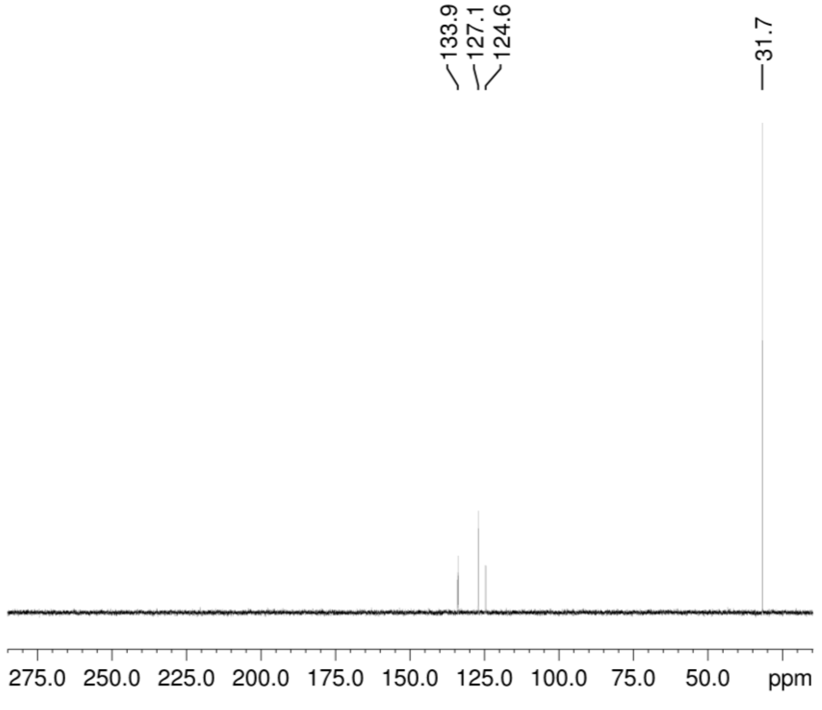

FIG. S10. ^{135}DEPT NMR ($CDCl_3$) SPECTRUM OF **1A**

NMR spectra of 1b

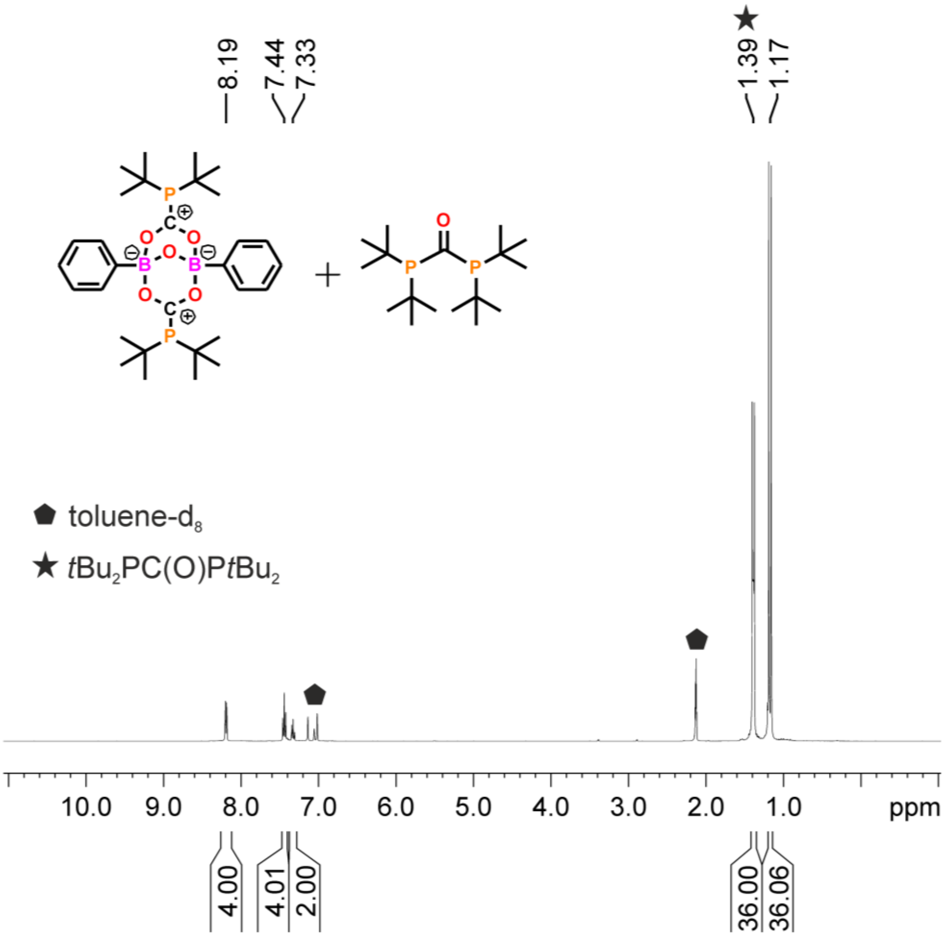

FIG. S11. ^{1}H NMR (TOLUENE-D$_8$) SPECTRUM OF **1B**

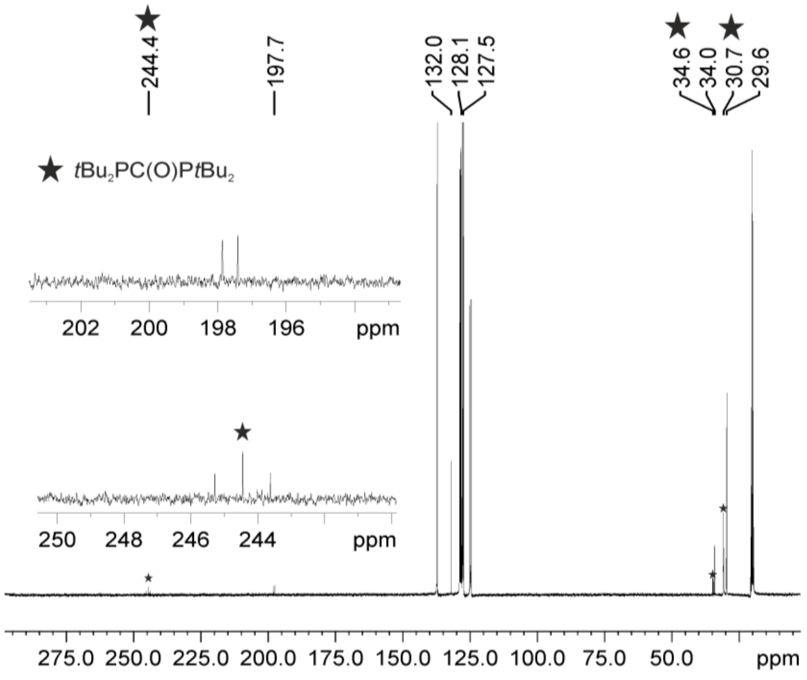

FIG. S12. $^{13}C\{^1H\}$ NMR (TOLUENE-D$_8$) SPECTRUM OF **1B**

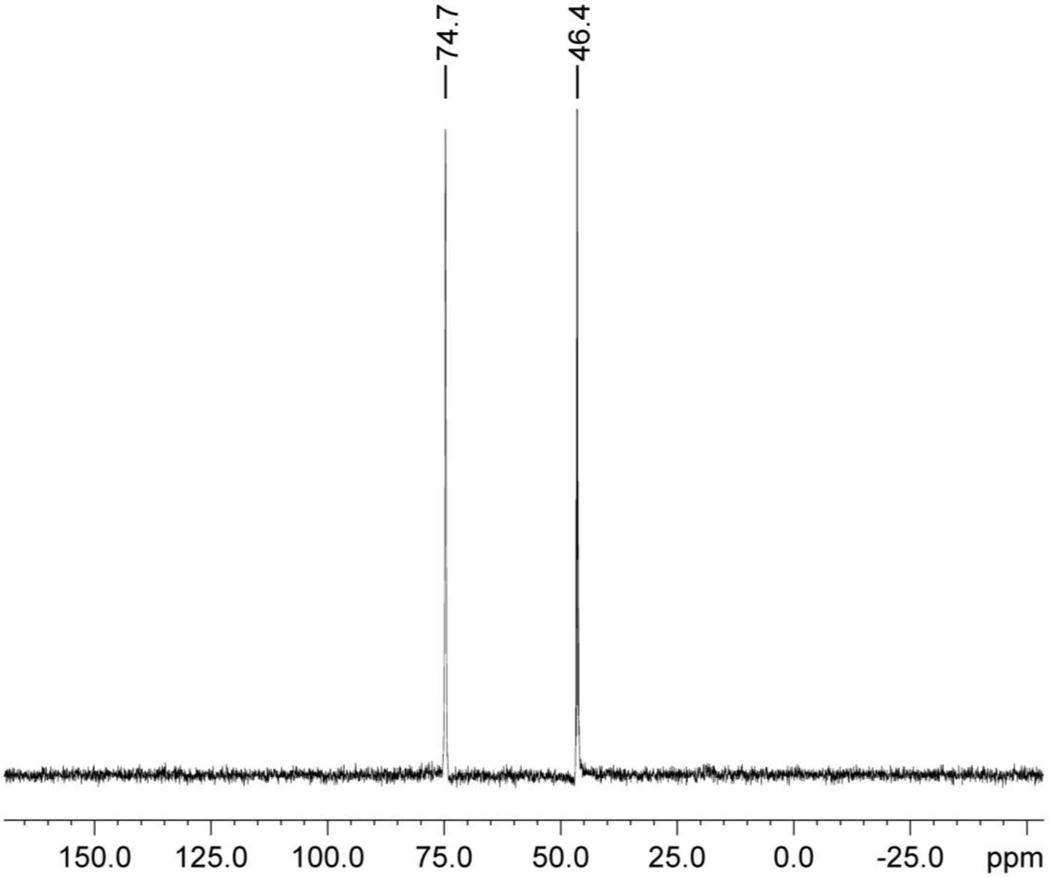

FIG. S13. $^{31}P\{^{1}H\}$ NMR (TOLUENE-D_8) SPECTRUM OF **1B**

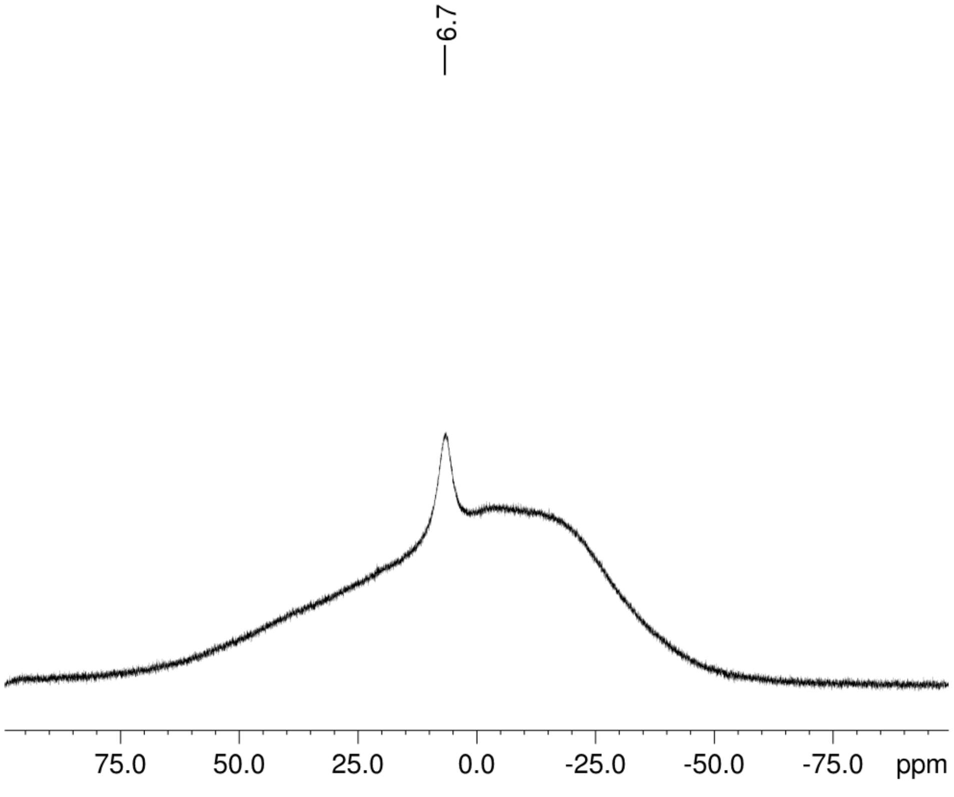

FIG. S14. ^{11}B NMR (TOLUENE-D_8) SPECTRUM OF **1B**

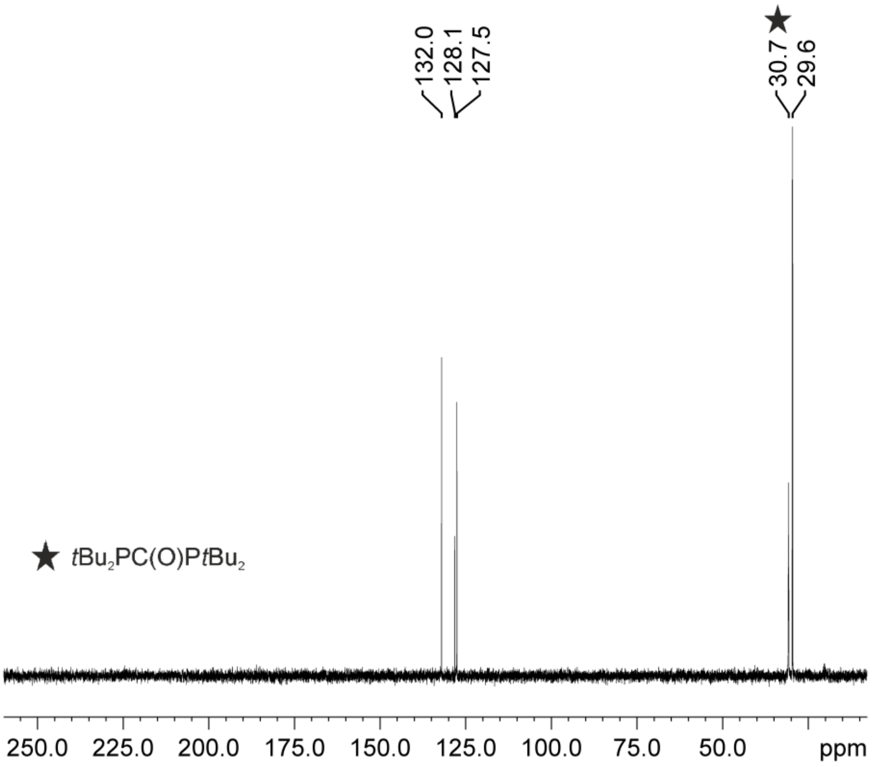

FIG. S15. [135]DEPT NMR (TOLUENE-D$_8$) SPECTRUM OF **1B**

NMR spectra of 1d

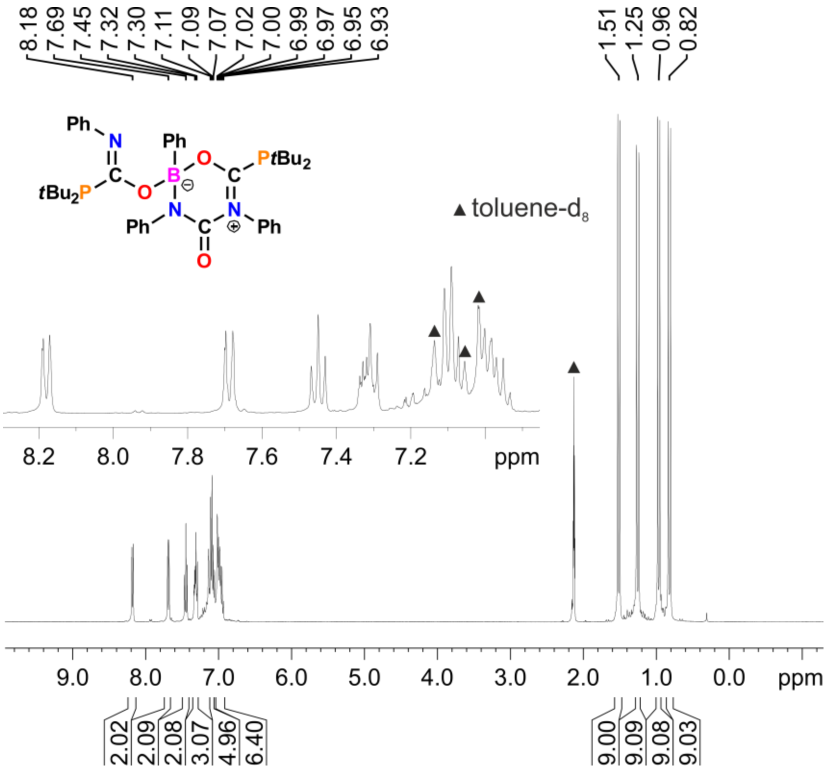

FIG. S16. ^{1}H NMR (TOLUENE-D$_8$) SPECTRUM OF **1D**

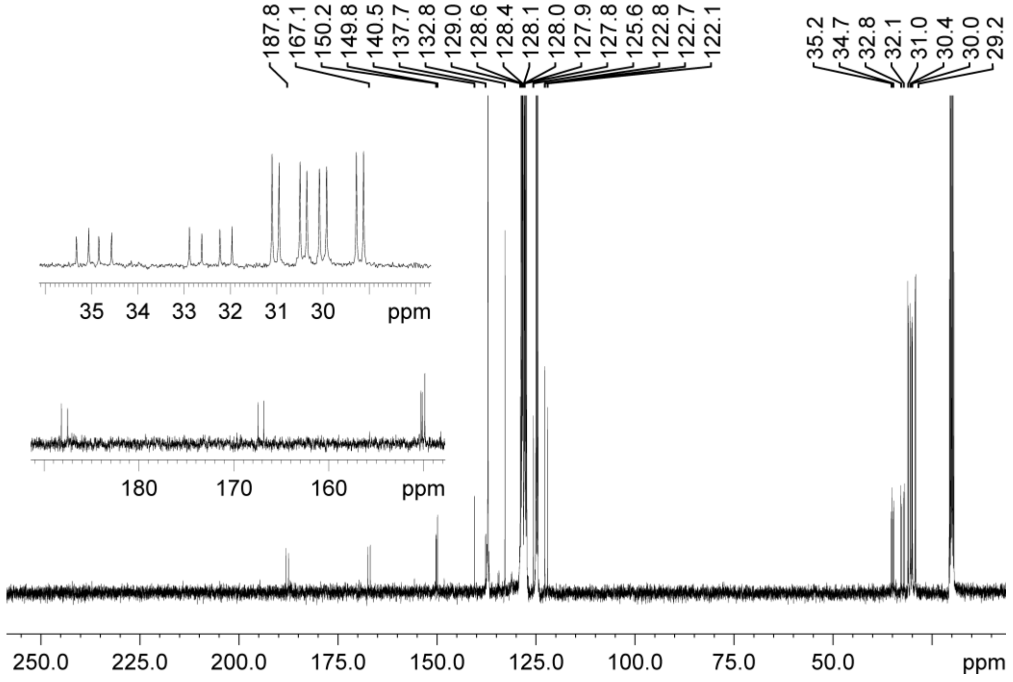

FIG. S17. $^{13}C\{^{1}H\}$ NMR (TOLUENE-D_8) SPECTRUM OF **1D**

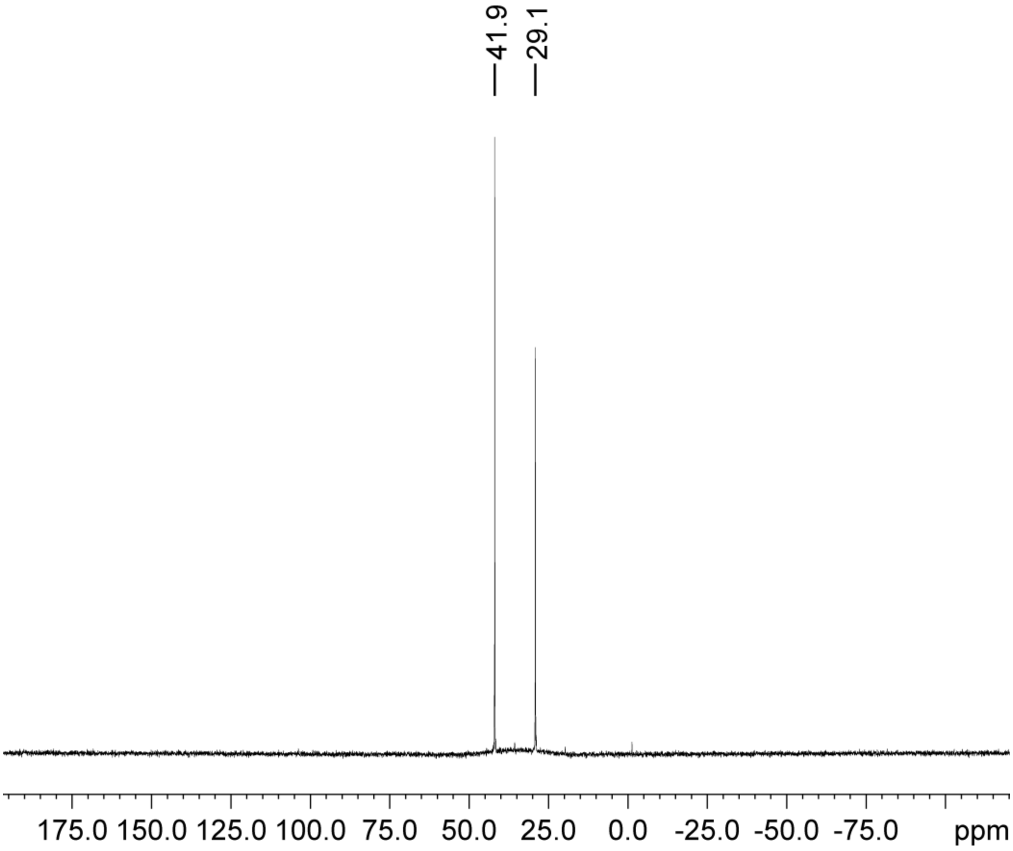

FIG. S18. $^{31}P\{^{1}H\}$ NMR (TOLUENE-D_8) SPECTRUM OF **1D**

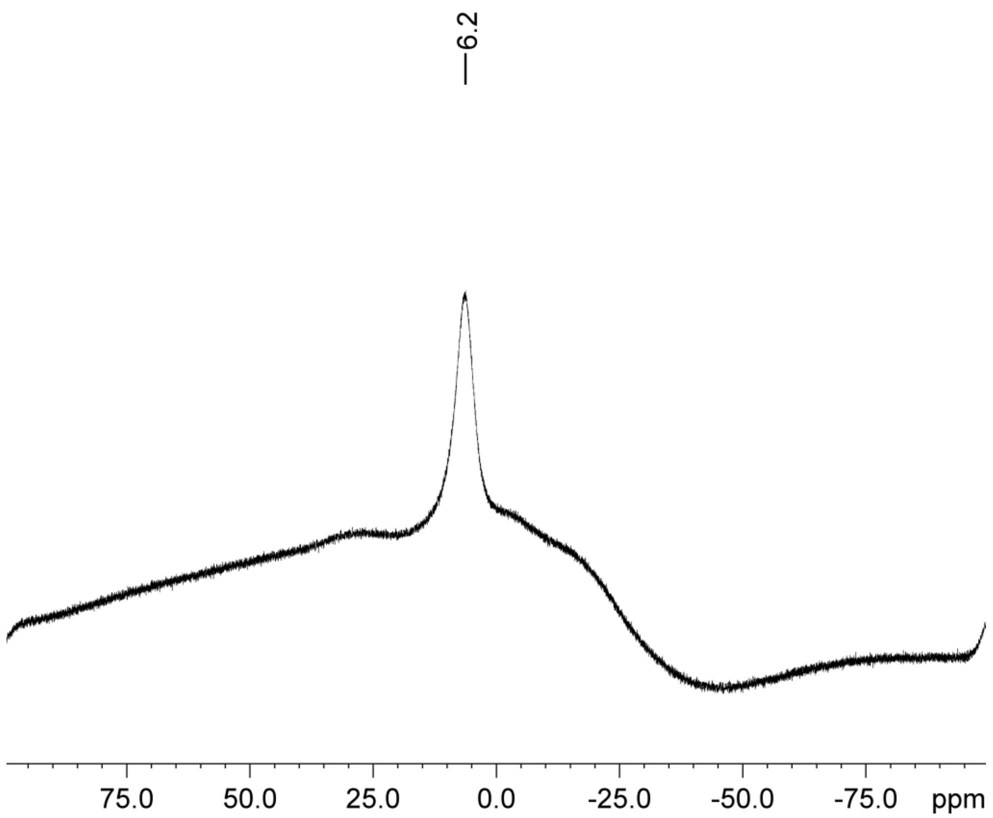

FIG. S19. ^{11}B NMR (TOLUENE-D_8) SPECTRUM OF **1D**

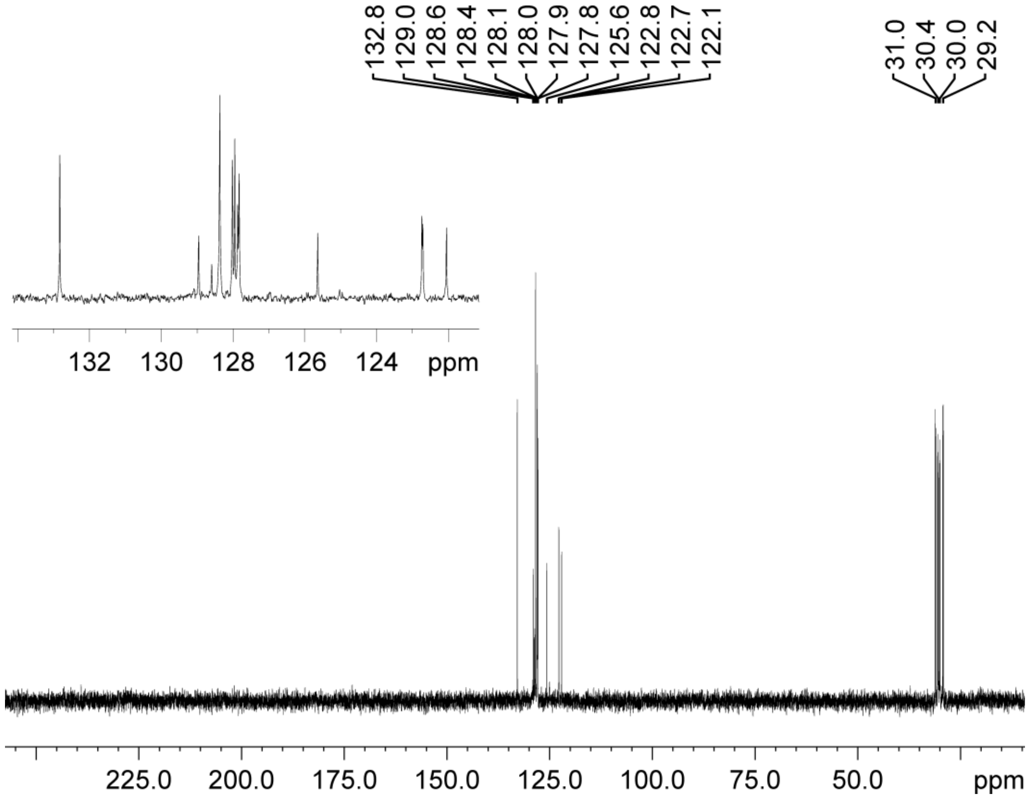

FIG. S20. ^{135}DEPT NMR (TOLUENE-D_8) SPECTRUM OF **1D**

NMR spectra of 2b

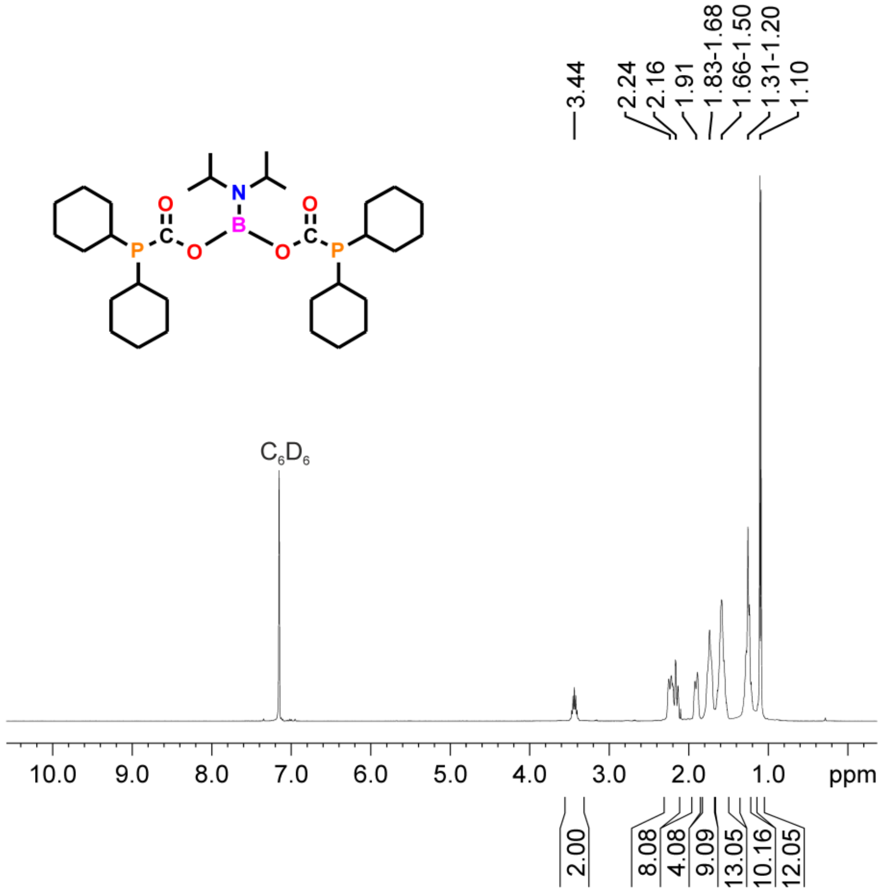

FIG. S21. ^{1}H NMR (C_6D_6) SPECTRUM OF **2B**

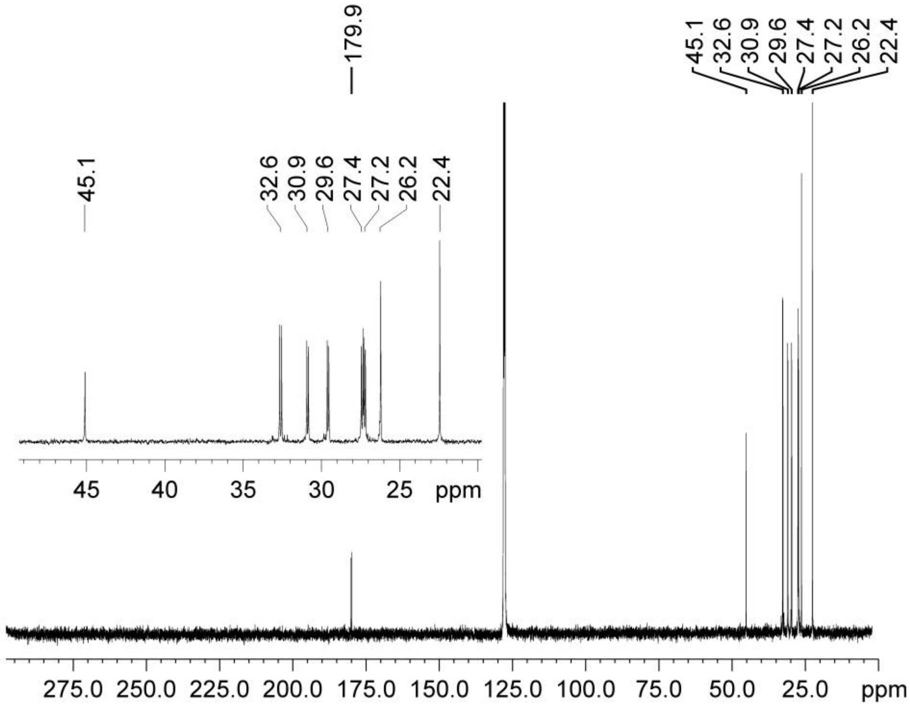

FIG. S22. ^{13}C{^{1}H} NMR (C_6D_6) SPECTRUM OF **2B**

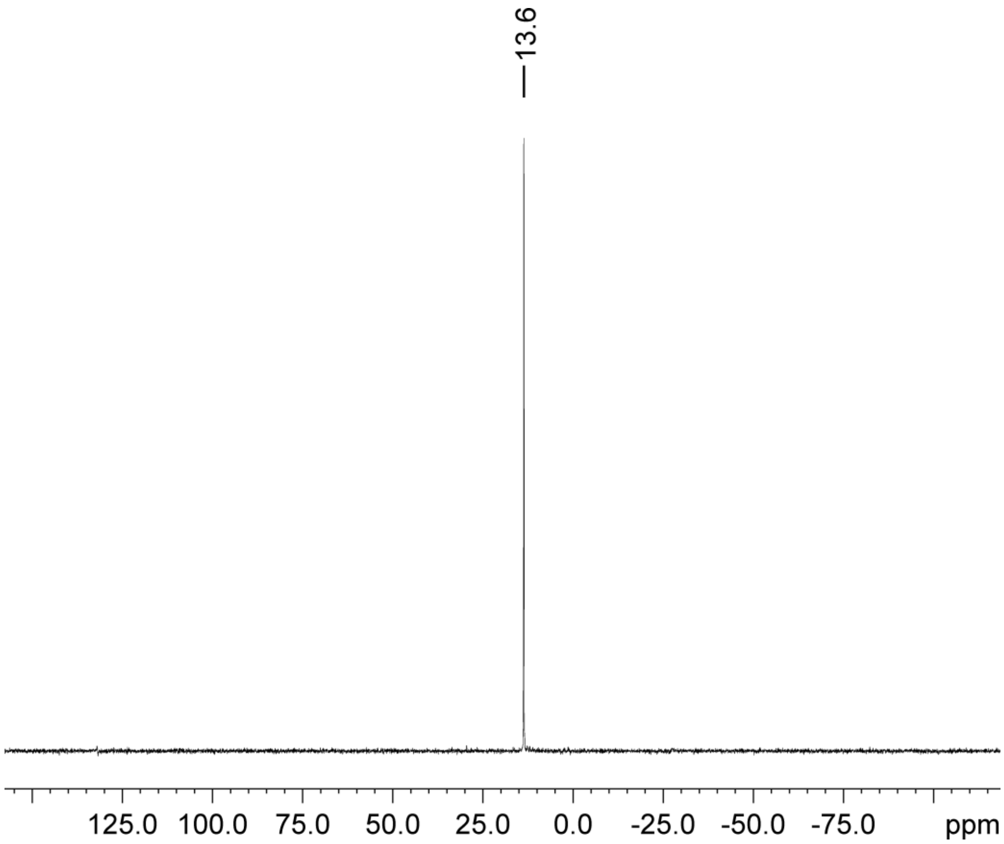

FIG. S23. $^{31}P\{^{1}H\}$ NMR (C_6D_6) SPECTRUM OF **2B**

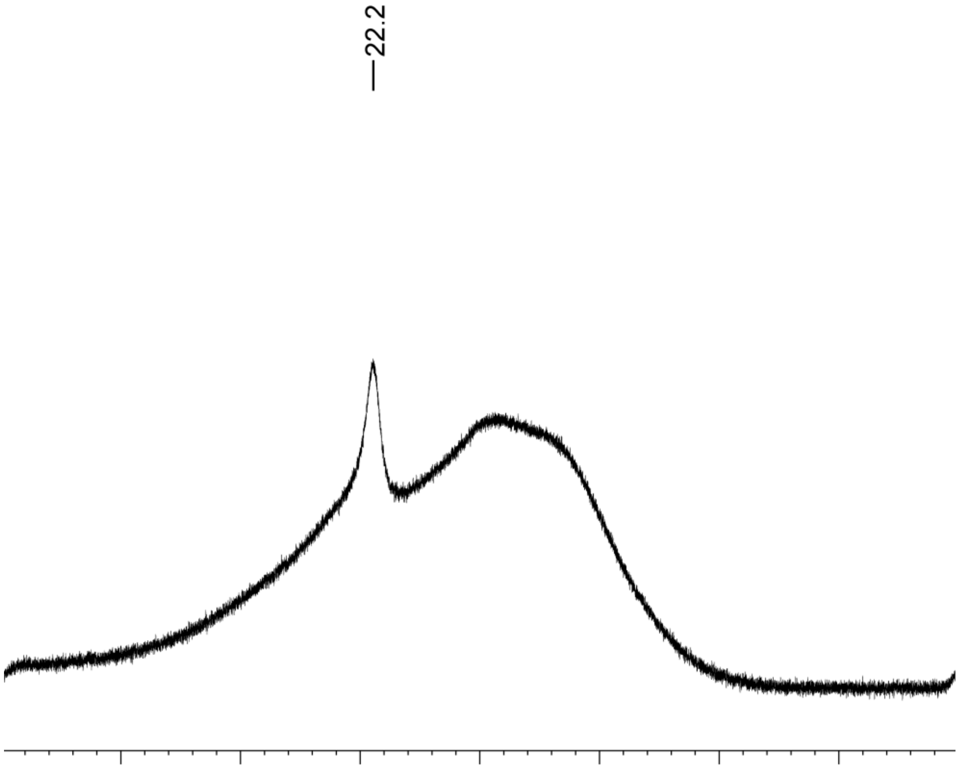

FIG. S24. ^{11}B NMR (C_6D_6) SPECTRUM OF **2B**

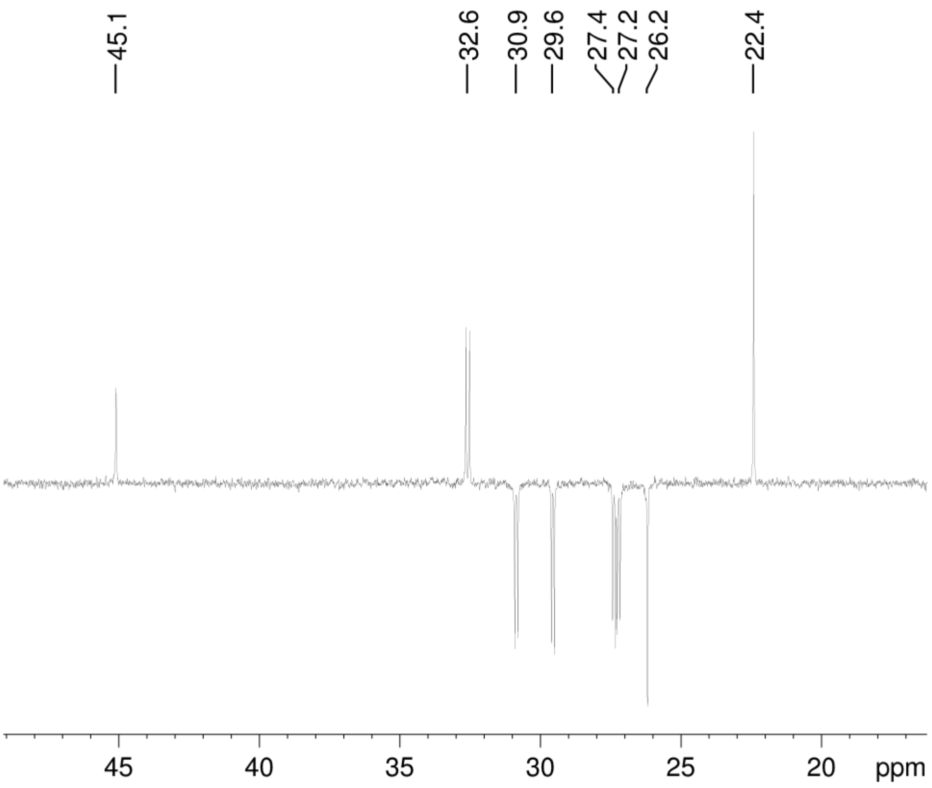

FIG. S25. [135]DEPT NMR (C_6D_6) SPECTRUM OF **2B**

NMR spectra of 2c

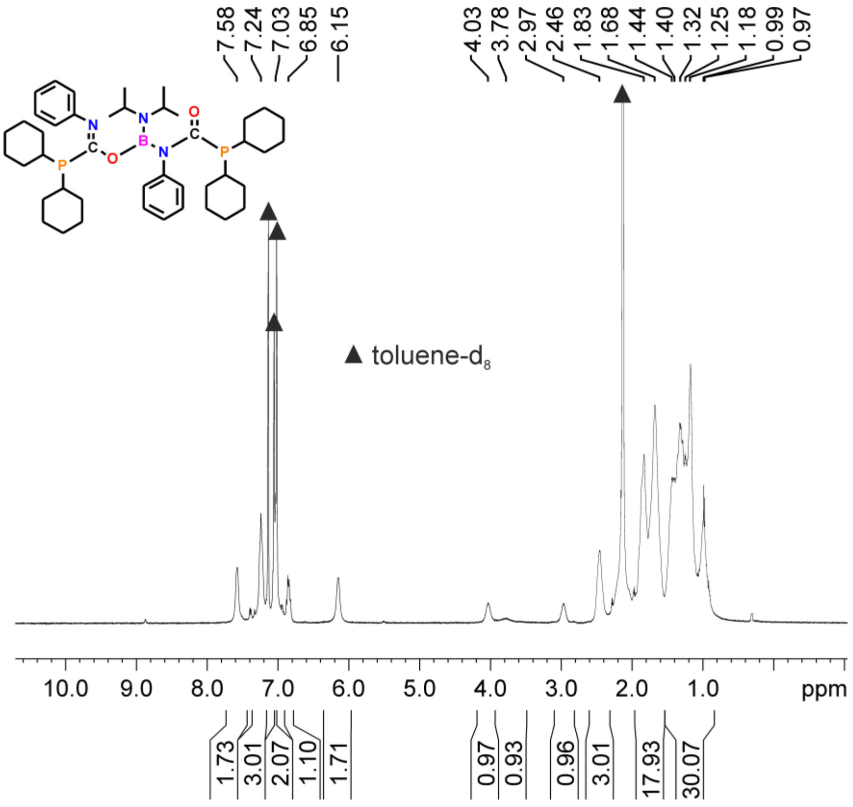

FIG. S26. ^{1}H NMR (TOLUENE-D_8, 298K) SPECTRUM OF **2C**

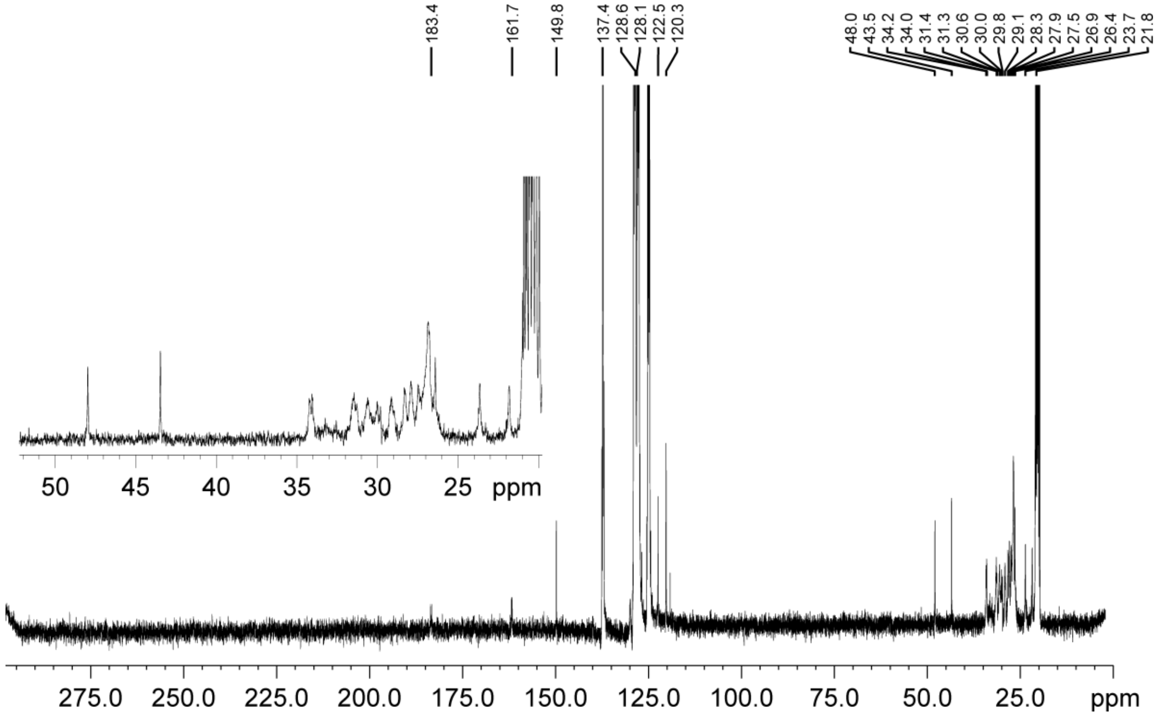

FIG. S27. $^{13}C\{^1H\}$ NMR (TOLUENE-D$_8$, 248K) SPECTRUM OF **2C**

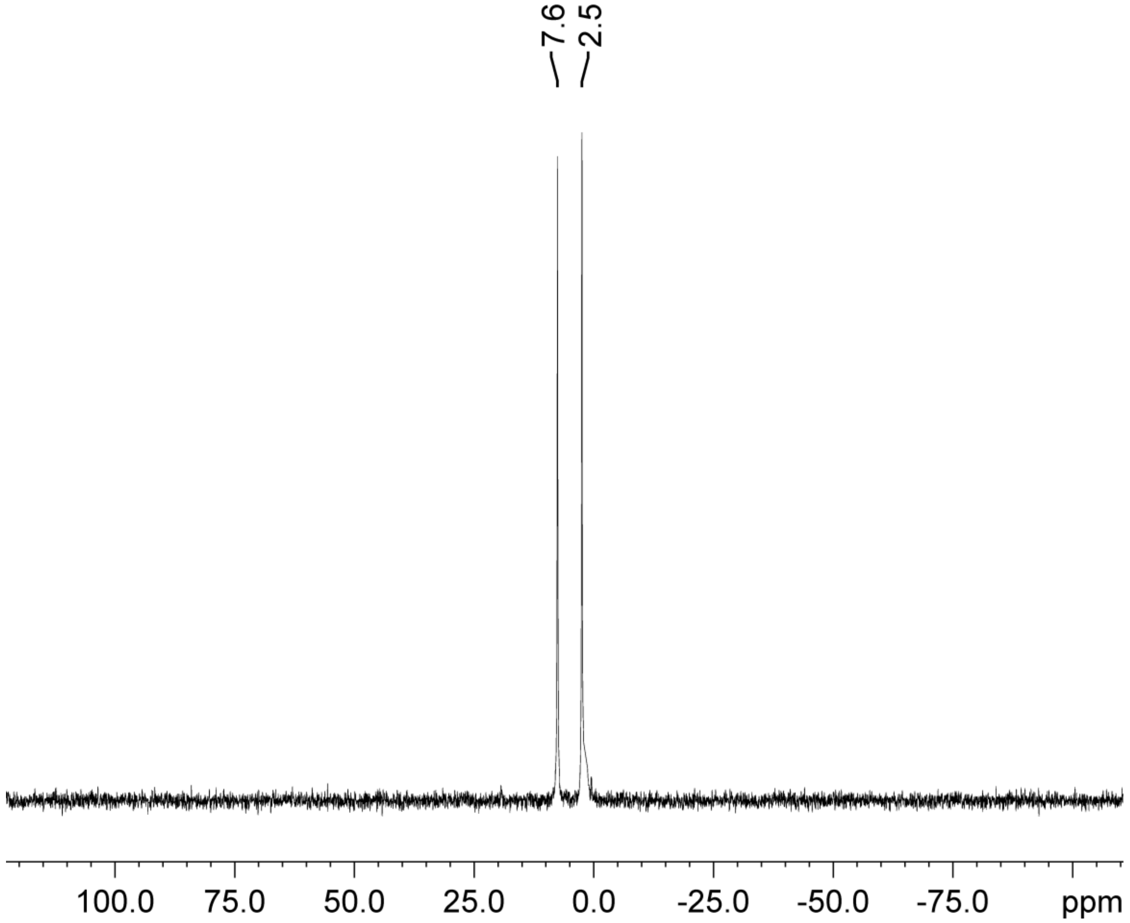

FIG. S28. $^{31}P\{^1H\}$ NMR (TOLUENE-D$_8$, 298K) SPECTRUM OF **2C**

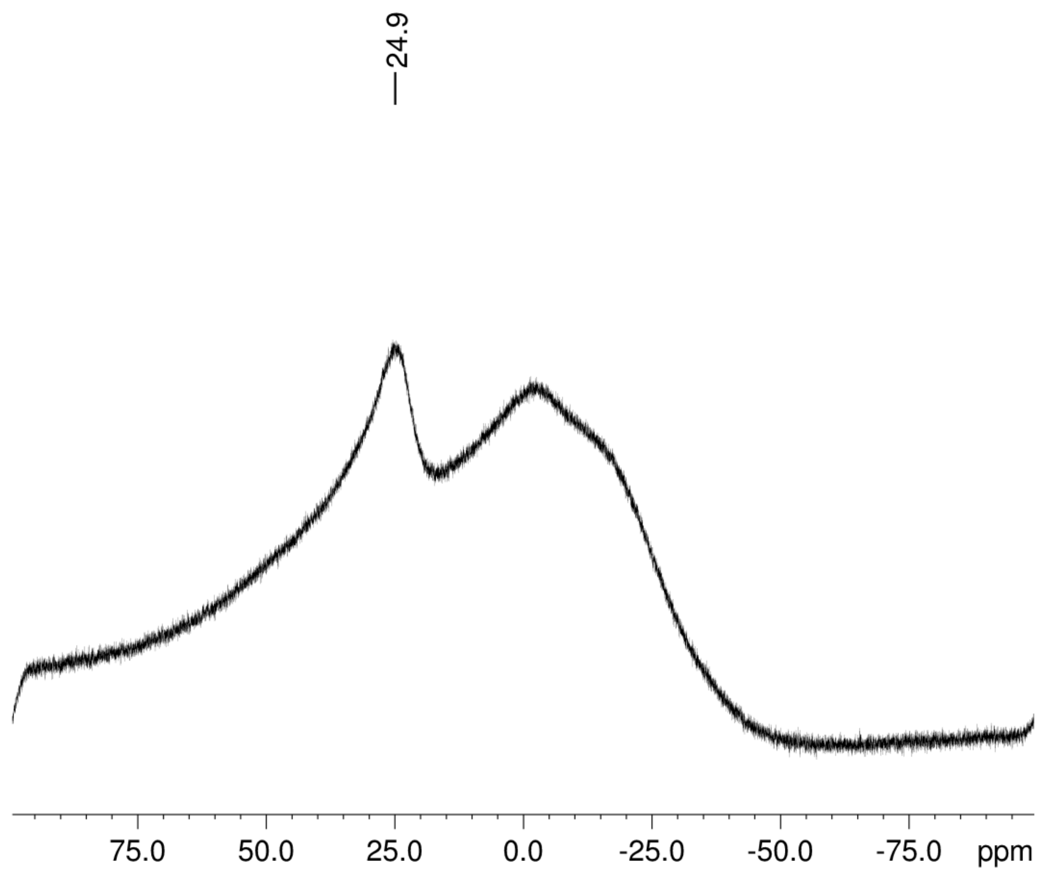

FIG. S29. ^{11}B NMR (TOLUENE-D_8, 298K) SPECTRUM OF **2C**

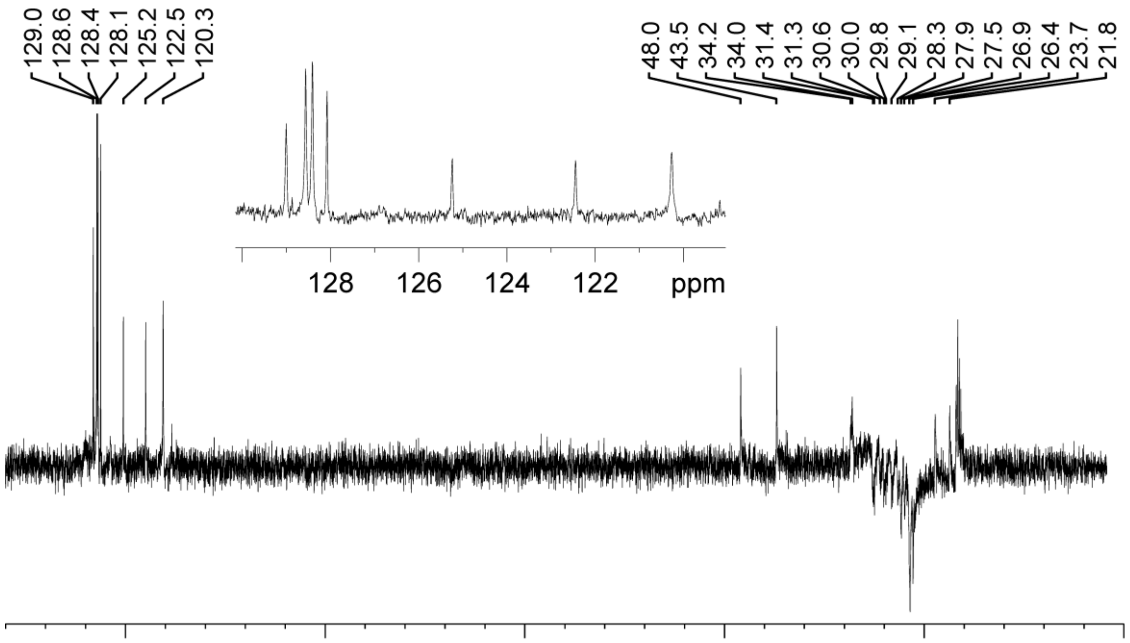

FIG. S30. ^{135}DEPT NMR (TOLUENE-D_8, 248K) SPECTRUM OF **2C**

NMR spectra of 2d

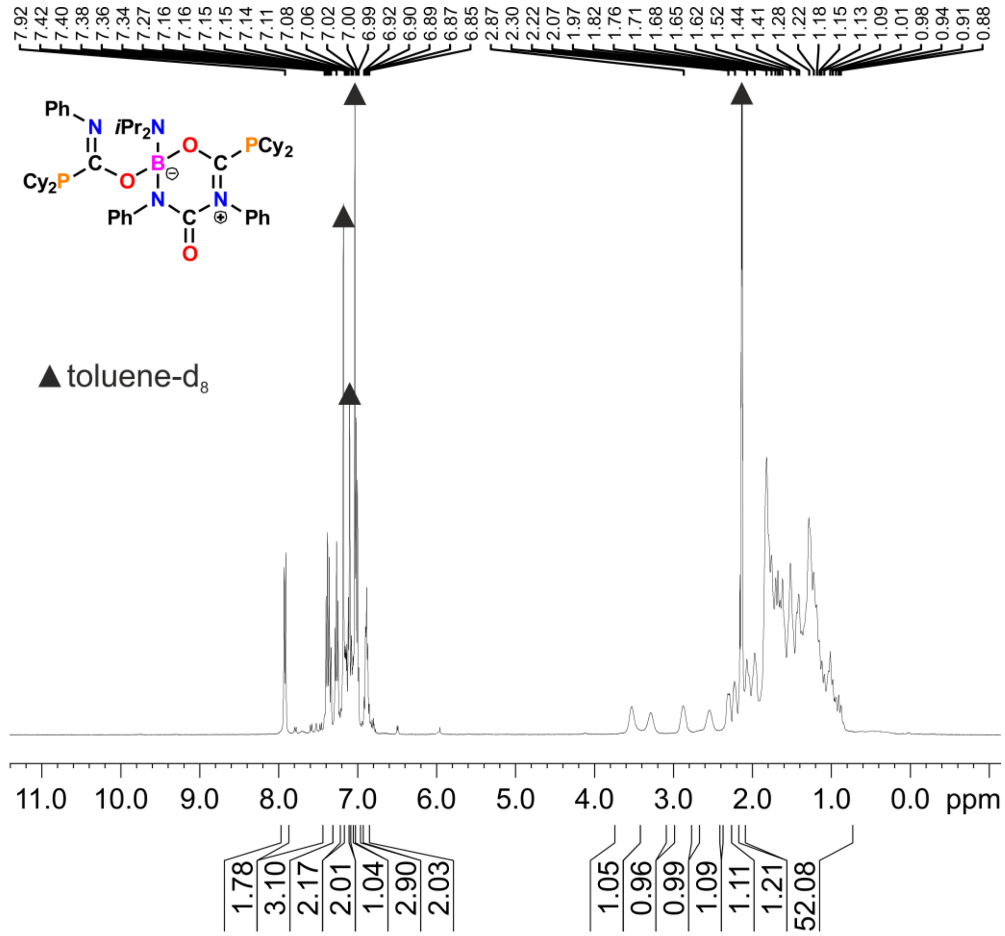

FIG. S31. ^{1}H NMR (TOLUENE-D$_8$, 298K) SPECTRUM OF **2D**

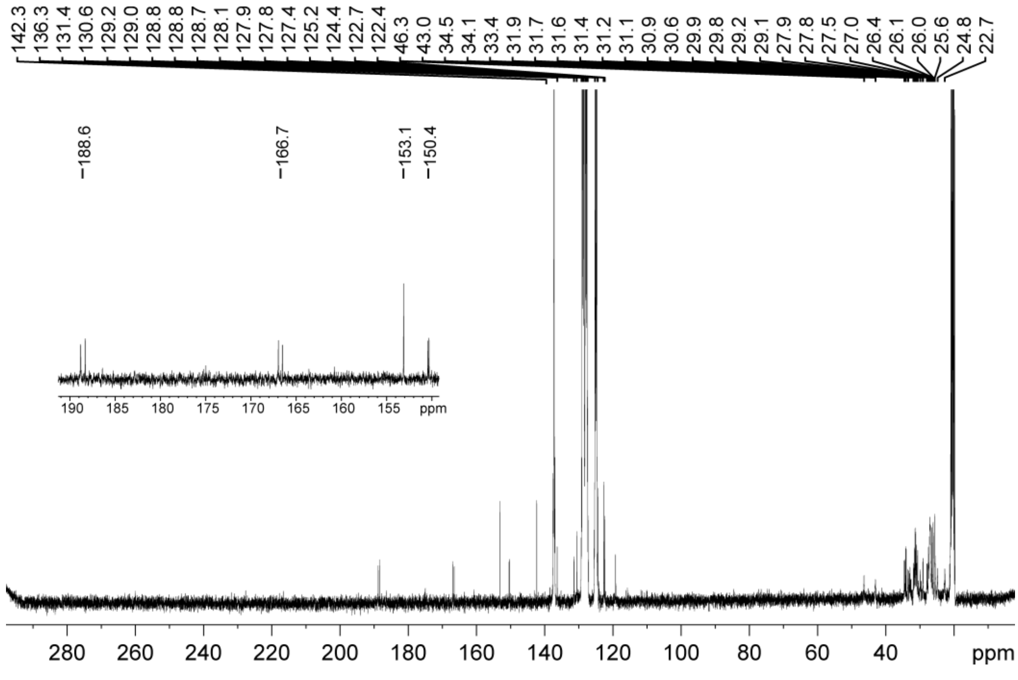

FIG. S32. [13]C{[1]H} (TOLUENE-D$_8$, 248K) SPECTRUM OF **2D**

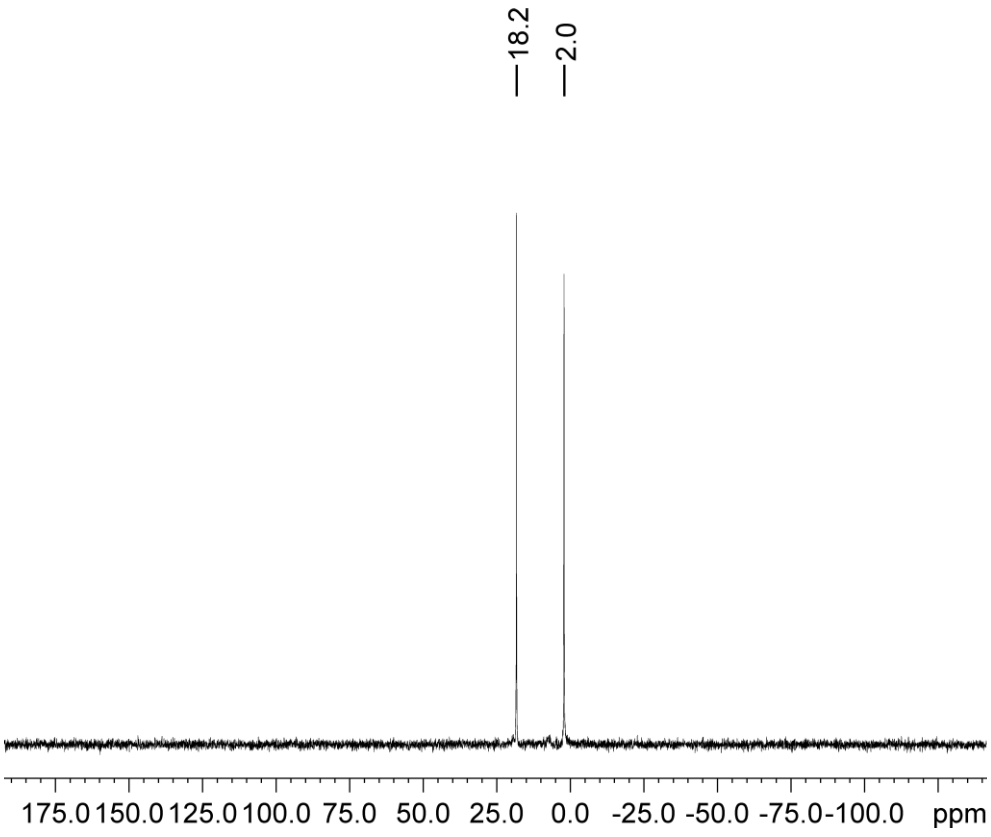

FIG. S33. $^{31}P\{^{1}H\}$ (TOLUENE-D_8, 298K) SPECTRUM OF **2D**

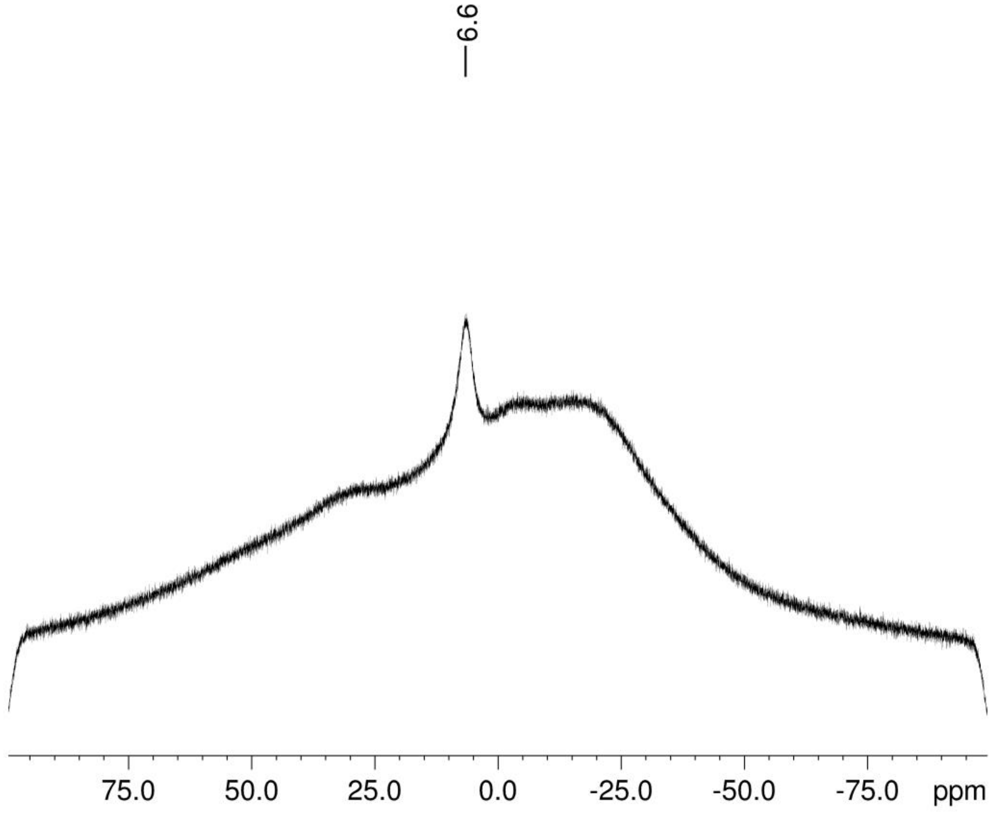

FIG. S34. ^{11}B NMR (TOLUENE-D_8, 298K) SPECTRUM OF **2D**

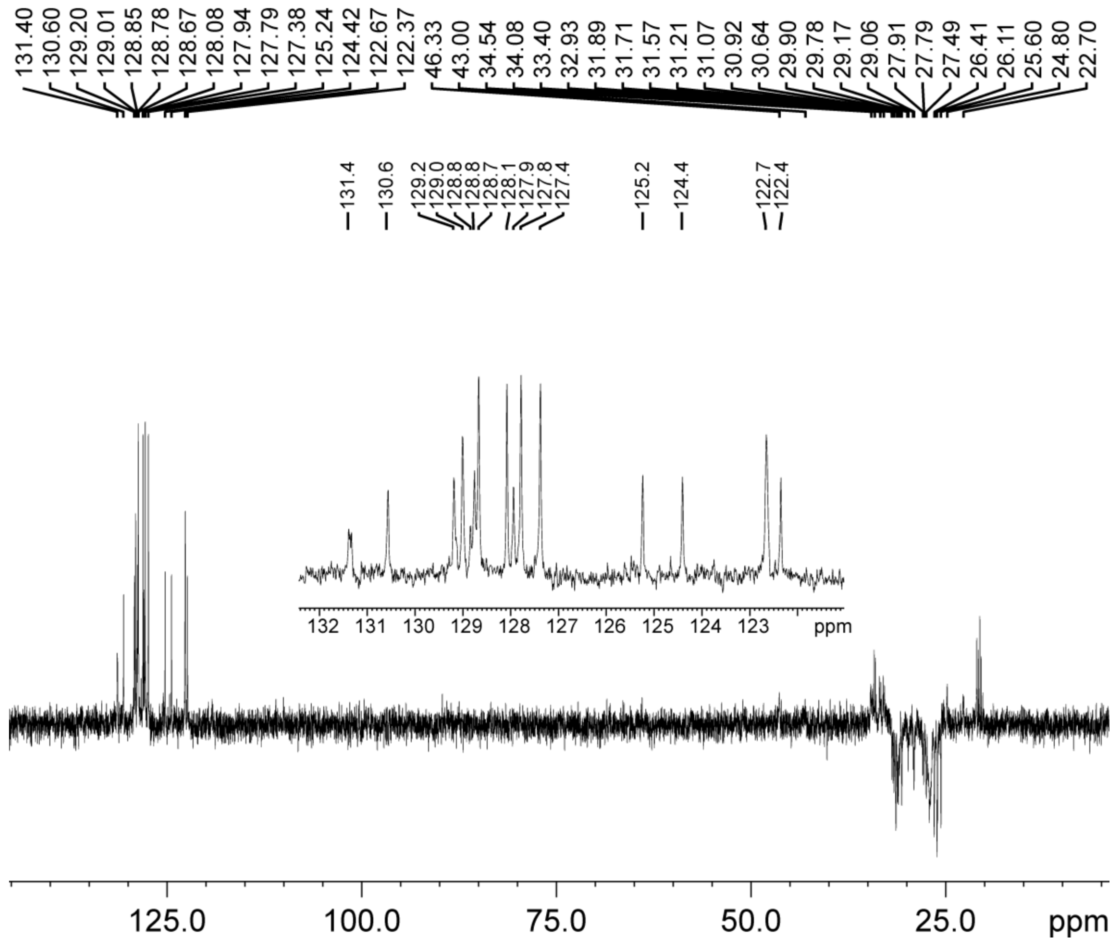

FIG. S35. ^{135}DEPT NMR (TOLUENE-$D_{8,}$ 248K) SPECTRUM OF **2D**

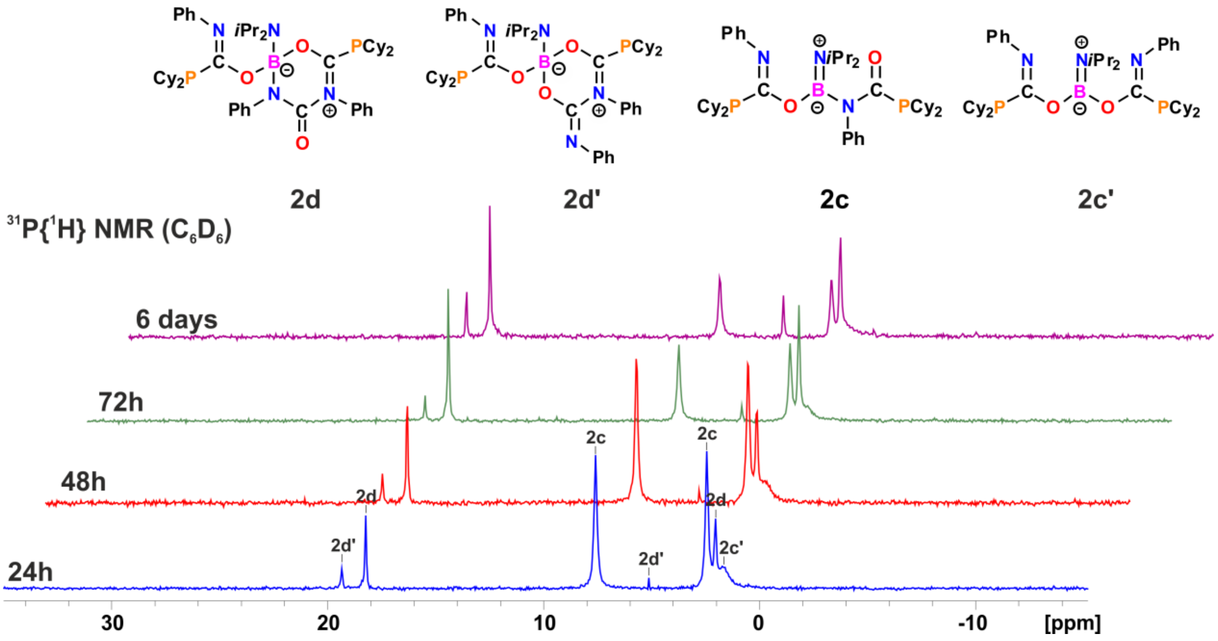

FIG. S36. ^{31}P{^{1}H} NMR (C_6D_6) SPECTRUM OF REACTION MIXTURE **2D**

IR spectra of isolated compounds

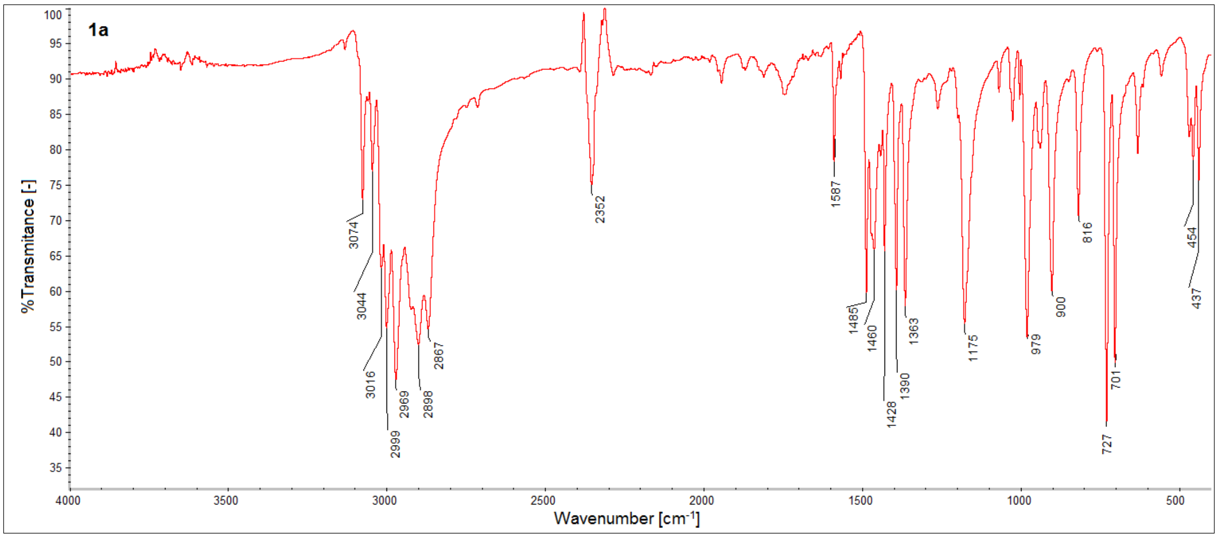

FIG. S37. IR SPECTRUM OF SOLID **1A**

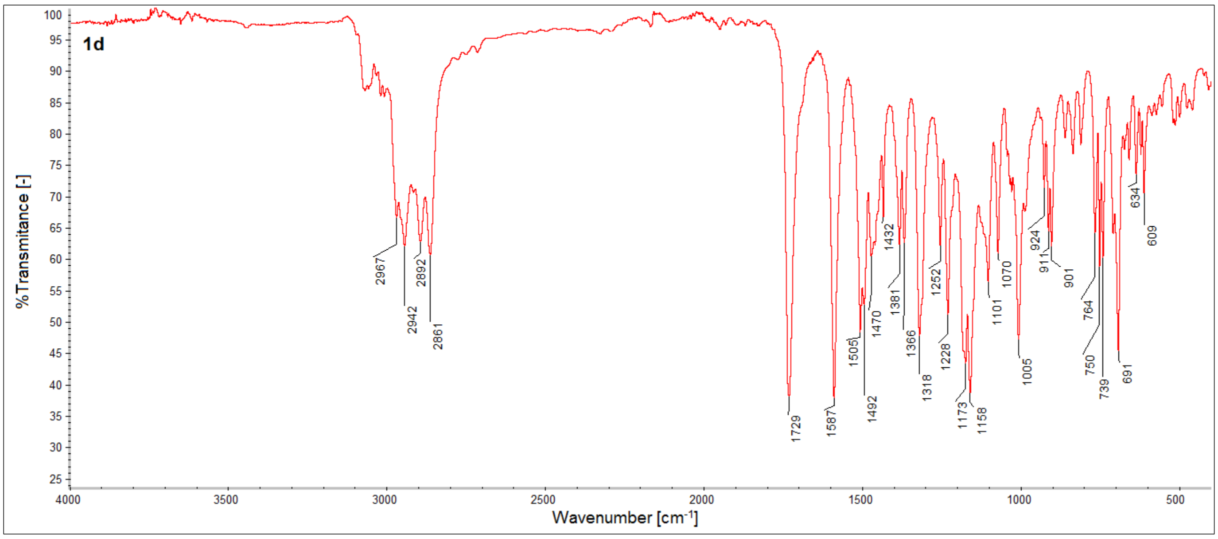

FIG. S38. IR SPECTRUM OF SOLID **1D**

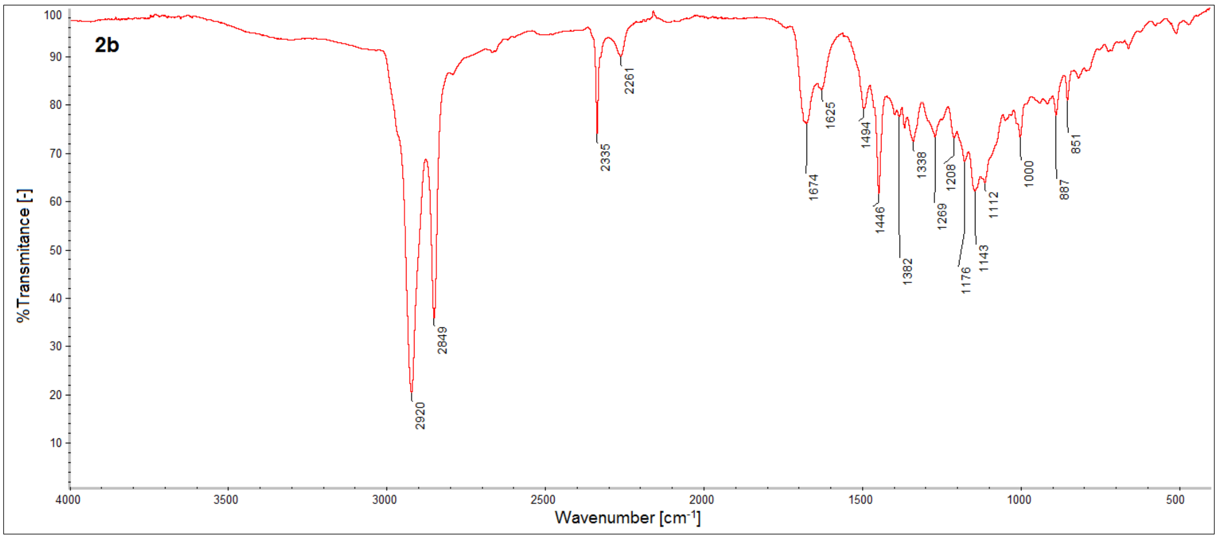

FIG. S39. IR SPECTRUM OF OIL **2B**

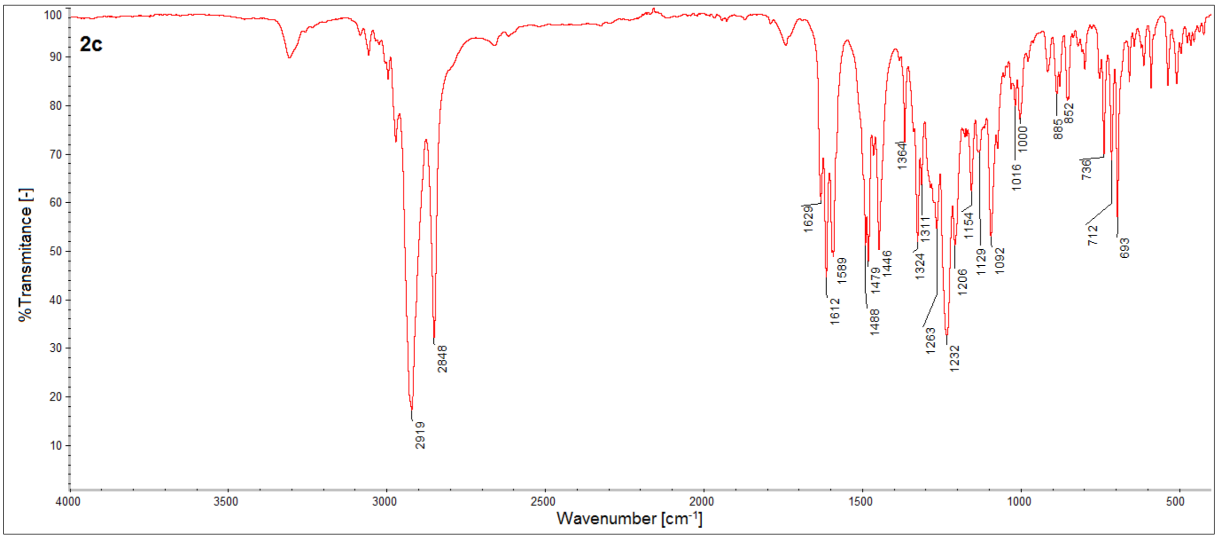

FIG. S40. IR SPECTRUM OF SOLID **2C**

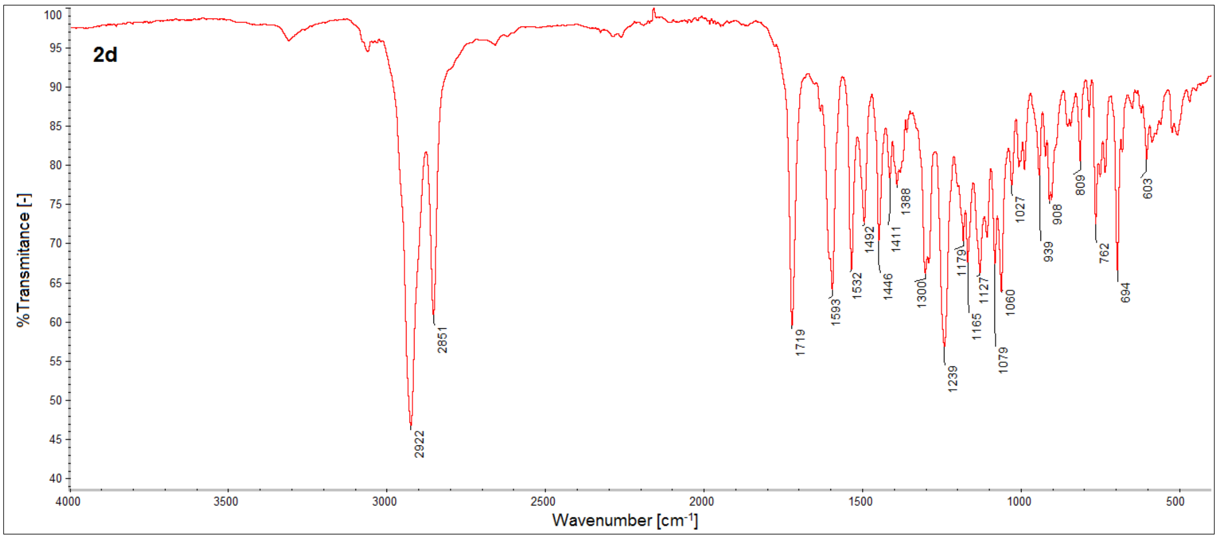

FIG. S41. IR SPECTRUM OF SOLID **2D**

DFT calculations

General methods

All calculations presented in the paper were performed using the Gaussian 09[1] program package. Molecular geometries of all compounds were optimized using density functional theory at the TPSSTPSS functional by Tao *et al*[2] with 6-31+G(d,p) basis set. The TPSSTPSS exchange-correlation functional has been chosen, as it has good overall performance for the description of main-group element compounds. By adding GD3BJ keyword that includes D3 version of Grimme dispersion with Becke-Johnson[3] damping into calculations it also accounts well for long-range and dispersion interactions. Molecular geometries were energy-optimized and the most stable (the lowest energy) conformer was identified during the potential energy surface scanning. Nature of the final gas-phase geometries as local minima (no imaginary frequencies) or transition states (one imaginary frequency) on the potential energy surface was then validated by harmonic frequency calculations at the same level of theory. Values of calculated energies, enthalpies and Gibbs free-energies derived from thermochemical calculations were corrected for the zero-point energy (ZPE). Scans of potential energy surface along the RR'**P-C**O_2/PhN**C**O/H_2 and R**B-O**CO/PhN**CO**/H_2 bonds were performed to establish local minima corresponding to transition products and confirm proposed mechanism of the reaction. Local maxima related to transition states were also established and validated by IRC calculations (to confirm that a located saddle points lie on the minimum energy path between assumed minima) and used to determine energy barriers between respective transformations. Values of energy barriers $\Delta G^{\ddagger}$ and $\Delta H^{\ddagger}$ of reactions **1a-2d** were determined as the difference between energy of rate-determining transition state and rate-determining intermediate as described in [4]. For reactions of hydrogen activation (paths A, B and C) solvation effects were included as single point calculations using the PCM-SMD model.[5] The quantum mechanical tunneling was not included into our DFT-based approach and the resulting energy barriers for H_2 activation may be overestimated.[6,7]

Condensed Fukui functions[8] and dual descriptors[8,9] were determined using optimized structures to single point calculations on diphosphinoboranes **1, 1'** and **2** and related intermediates and products for *N*, *N-1* and *N+1* electron states at TPSSTPSS/6-31+G(d,p) level of theory. Condensed to atom parameters were calculated using partial charges derived *via* Hirshfeld population analysis. NBO analysis was performed for at TPSSTPSS//Def2TZVP level of theory by applying the NBO 3.1[10] module built-in Gaussian 09.

TABLE S10. SELECTED COMPUTATIONAL PARAMETERS OBTAINED FOR CONSIDERED SYSTEMS (IN ATOMIC UNITS A.U.): $\mathbf{E_0}$ - ELECTRONIC ENERGY; $\mathbf{E_{0+...}}$ - SUM OF ELECTRONIC AND: $\mathbf{E_{ZPE}}$ - ZERO-POINT ENERGIES, $\mathbf{E_{THERM}}$ - THERMAL ENERGIES, **H** – THERMAL ENTHALPIES, **G** - THERMAL FREE ENERGIES CALCULATED AT TPSSTPSS//6-31+G(d,p) LEVEL OF THEORY.

**Substrates**							
Compound	E_{electr} [A.U.]	$\varepsilon_0 + E_{ZPE}$ [A.U.]	$\varepsilon_0 + E_{therm}$ [A.U.]	ε_0 + H [A.U.]	ε_0 + G [A.U.]	ΔH [kcal mol^{-1}]	ΔG [kcal mol^{-1}]
1	-1570.949110	-1570.368400	-1570.335632	-1570.334688	-1570.428653	-	-
1'	-1570.950780	-1570.369877	-1570.337168	-1570.336224	-1570.429995	-	-
2	-1940.994011	-1940.158294	-1940.118906	-1940.117961	-1940.229044	-	-
PhNCO	-399.841622	-399.739475	-399.732237	-399.731293	-399.771924	-	-
CO_2	-188.617512	-188.606279	-188.603617	-188.602673	-188.627020	-	-
CS_2	-834.521371	-834.514556	-834.511428	-834.510484	-834.537516	-	-
H_2	-1.178361	-1.168174	-1.165813	-1.164869	-1.179656	-	-
tBu_2PH	-657.732206	-657.520494	-657.507378	-657.506434	-657.557858	-	-
**Reaction 1' + H_2 - path A**							
Compound	E_{electr} [A.U.]	$\varepsilon_0 + E_{ZPE}$ [A.U.]	$\varepsilon_0 + E_{therm}$ [A.U.]	ε_0 + H [A.U.]	ε_0 + G [A.U.]	ΔH	ΔG [kcal

						[kcalmol^{-1}]	mol^{-1}]
TS1	-1572.094063	-1571.497631	-1571.464843	-1571.463899	-1571.556744	23.82	33.88
I1 (1a’)	-914.361925	-914.018640	-913.999238	-913.998294	-914.065159	-2.33	-8.56
TS2$_A$	-1828.742809	-1828.050228	-1828.010413	-1828.009469	-1828.121331	-12.90	-11.36
TS2$_B$	-1828.743001	-1828.010139	-1828.009195	-1828.119581	-1828.119581	-83.42	-10.24
I2	-1828.762475	-1828.068260	-1828.028962	-1828.028018	-1828.135517	-24.78	-20.45
TS3	-1828.761074	-1828.066968	-1828.028363	-1828.027419	-1828.133329	-24.40	-19.05
1a	-1828.811996	-1828.115345	-1828.076961	-1828.076017	-1828.180341	-55.52	-49.15
**Reaction 1 + H_2 - path B**							
Compound	E_{electr} [A.U.]	$\varepsilon_0 + E_{ZPE}$ [A.U.]	$\varepsilon_0 + E_{therm}$ [A.U.]	ε_0 + H [A.U.]	ε_0 + G [A.U.]	ΔH [kcalmol^{-1}]	ΔG [kcal.mol^{-1}]
TS1	-1572.088020	-1571.493064	-1571.460125	-1571.459181	-1571.553104	25.86	35.35
I1	-1572.169133	-1571.568903	-1571.535942	-1571.534998	-1571.628960	-22.70	-13.22
TS2	-1572.164087	-1571.563722	-1571.531415	-1571.530471	-1571.623094	-19.80	-9.47
1a”	-1572.172193	-1571.571993	-1571.539103	-1571.538159	-1571.632041	-24.72	-15.20
**Reaction 1 + H_2 - path C**							
Compound	E_{electr} [A.U.]	$\varepsilon_0 + E_{ZPE}$ [A.U.]	$\varepsilon_0 + E_{therm}$ [A.U.]	ε_0 + H [A.U.]	ε_0 + G [A.U.]	ΔH [kcalmol^{-1}]	ΔG [kcal mol^{-1}]
TS1	-1572.094063	-1571.497631	-1571.464843	-1571.463899	-1571.556744	32.78	41.71
I1 (1a’)	-914.361925	-914.018640	-913.999238	-913.998294	-914.065159	-3.31	-9.42
TS2$_A$	-1828.742809	-1828.050228	-1828.010413	-1828.009469	-1828.121331	-14.87	-13.08
TS2$_B$	-1828.743001	-1828.010139	-1828.009195	-1828.119581	-1828.119581	-85.38	-11.96
I2	-1828.762475	-1828.068260	-1828.028962	-1828.028018	-1828.135517	-26.75	-22.17
TS3	-1828.761074	-1828.066968	-1828.028363	-1828.027419	-1828.133329	-26.37	-20.77
1a	-1828.811996	-1828.115345	-1828.076961	-1828.076017	-1828.180341	-57.49	-50.87
**Reaction 1' + CO_2**							
Compound	E_{electr} [A.U.]	$\varepsilon_0 + E_{ZPE}$ [A.U.]	$\varepsilon_0 + E_{therm}$ [A.U.]	ε_0 + H [A.U.]	ε_0 + G [A.U.]	ΔH [kcalmol^{-1}]	ΔG [kcal mol^{-1}]
TS1	-1759.561362	-1758.967199	-1758.931744	-1758.930799	-1759.030323	5.19	17.09
I1	-1759.562474	-1758.967380	-1758.931589	-1758.930645	-1759.030509	5.28	16.97
TS2	-1759.556546	-1758.962080	-1758.927099	-1758.926155	-1759.023998	8.16	21.14
I2a	-1759.597925	-1759.003294	-1758.967592	-1758.966648	-1759.068318	-17.77	-7.24
I2b	-1759.599273	-1759.005182	-1758.969162	-1758.968218	-1759.072136	-18.78	-9.68
I2c	-1759.594687	-1759.000963	-1758.964977	-1758.964033	-1759.066682	-16.10	-6.19
TS3	-1948.209183	-1947.602042	-1947.563486	-1947.562541	-1947.671497	-13.43	8.03
I3	-1948.244742	-1947.636699	-1947.597884	-1947.596940	-1947.707364	-35.46	-14.94
TS4	-2280.124971	-2279.418666	-2279.373174	-2279.372229	-2279.496420	-39.63	-3.51
I4	-2280.145654	-2279.437125	-2279.392282	-2279.391338	-2279.512248	-51.86	-13.65
TS5	-2280.123714	-2279.416882	-2279.371796	-2279.370852	-2279.493033	-38.75	-1.35
I5	-2280.175290	-2279.467178	-2279.421701	-2279.420756	-2279.545928	-70.70	-35.22
TS6	-2280.170212	-2279.461745	-2279.417389	-2279.416445	-2279.536095	-67.94	-28.92
I6	-2280.210894	-2279.501999	-2279.457330	-2279.456386	-2279.577624	-93.52	-55.52
TS7	-2280.194323	-2279.486812	-2279.442155	-2279.441211	-2279.564961	-83.80	-47.41
TS$_{PhBO}$	-1759.570448	-1758.977280	-1758.942066	-1758.941121	-1759.040436	-1.42	10.62
PhBO	-331.872604	-331.776111	-331.769721	-331.768777	-331.806896	-5.29	-7.49
*t*Bu$_2$PC(O)P*t*Bu$_2$	-1427.703120	-1427.207747	-1427.179319	-1427.178375	-1427.261810		
TS1$_{PhBO}$	-1902.827524	-1902.149782	-1902.109502	-1902.108558	-1902.220093	-2.28	10.76
I1$_{PhBO}$	-1902.887465	-1902.207665	-1902.167461	-1902.166517	-1902.279183	-39.39	-27.08
TS2$_{PhBO}$	-2091.501036	-2090.808295	-2090.765561	-2090.764616	-2090.881651	-36.46	-11.36
I2a$_{PhBO}$	-2091.531968	-2090.838278	-2090.795176	-2090.794231	-2090.915456	-55.43	-33.01
I2b$_{PhBO}$	-2091.540673	-2090.847036	-2090.803988	-2090.803044	-2090.922696	-61.07	-37.64
TS3$_{PhBO}$	-2091.538498	-2090.844967	-2090.802792	-2090.801847	-2090.919996	-60.31	-35.91
I3$_{PhBO}$	-2091.548542	-2090.853901	-2090.811770	-2090.810826	-2090.926675	-66.06	-40.19

$TS4_{PhBO}$	-2091.483438	-2090.792216	-2090.748459	-2090.747515	-2090.870140	-25.51	-3.99
$TS5_{PhBO}$	-2280.172655	-2279.465924	-2279.420475	-2279.419531	-2279.544703	-69.92	-34.43
1b	-2280.215040	-2279.506137	-2279.461593	-2279.460649	-2279.583459	-96.25	-59.25
$[Ph\text{-}B\text{-}O]_3$	-995.806643	-995.509406	-995.489766	-995.488822	-995.560729	-135.67	-114.72
**Reaction 2 + CO_2**							
Compound	E_{electr} [A.U.]	ε_0 + E_{ZPE} [A.U.]	ε_0 + E_{therm} [A.U.]	ε_0 + H [A.U.]	ε_0 + G [A.U.]	ΔH [kcalmol^{-1}]	ΔG [kcal mol^{-1}]
TS1	-2129.588306	-2128.73923	-2128.69756	-2128.696616	-2128.811831	15.38	28.33
$I1_A$	-2129.633138	-2128.783486	-2128.741186	-2128.740242	-2128.859068	-12.56	-1.92
TS2	-2129.630366	-2128.781492	-2128.739841	-2128.738897	-2128.855588	-11.70	0.30
I1B	-2129.647235	-2128.797515	-2128.755096	-2128.754152	-2128.873416	-21.46	-11.11
TS3	-2318.250228	-2317.38792	-2317.342726	-2317.341782	-2317.468784	-11.83	9.16
2b	-2318.288557	-2317.425544	-2317.379828	-2317.378884	-2317.509508	-35.59	-16.92
**Reaction 1' + PhNCO**							
Compound	E_{electr} [A.U.]	ε_0 + E_{ZPE} [A.U.]	ε_0 + E_{therm} [A.U.]	ε_0 + H [A.U.]	ε_0 + G [A.U.]	ΔH [kcalmol^{-1}]	ΔG [kcal mol^{-1}]
$TS1_A$	-1970.785489	-1970.101242	-1970.060466	-1970.059522	-1970.173583	5.12	18.15
$TS1_B$	-1970.784744	-1970.100443	-1970.059652	-1970.058708	-1970.172831	5.64	18.63
$I1_A$	-1970.800198	-1970.114123	-1970.073345	-1970.072401	-1970.185558	-3.13	10.48
$I1_B$	-1970.801063	-1970.114547	-1970.074054	-1970.07311	-1970.184497	-3.58	11.16
$TS2_A$	-1970.798324	-1970.111875	-1970.072301	-1970.071357	-1970.1793	-2.46	14.48
$TS2_B$	-1970.796064	-1970.110024	-1970.070337	-1970.069392	-1970.178465	-1.20	15.02
$I2_A$	-1970.836772	-1970.151644	-1970.110828	-1970.109884	-1970.223466	-27.13	-13.80
$I2_B$	-1970.834739	-1970.149078	-1970.108327	-1970.107383	-1970.221386	-25.53	-12.47
I3	-1970.853105	-1970.166071	-1970.126048	-1970.125104	-1970.235601	-36.88	-21.57
TS3	-2370.696498	-2369.905882	-2369.858086	-2369.857142	-2369.984747	-37.35	-6.98
I4	-2370.698476	-2369.907237	-2369.859043	-2369.858099	-2369.986044	-37.97	-7.81
TS4	-2370.694202	-2369.903663	-2369.855979	-2369.855034	-2369.981935	-36.00	-5.18
I5	-2370.742354	-2369.951436	-2369.903122	-2369.902177	-2370.033867	-66.19	-38.44
TS5	-2370.721191	-2369.930683	-2369.883097	-2369.882153	-2370.010502	-53.37	-23.48
I6	-2370.729325	-2369.938459	-2369.890182	-2369.889238	-2370.019851	-57.91	-29.46
TS6	-2770.58047	-2769.686953	-2769.630558	-2769.629614	-2769.777804	-63.72	-20.52
TS7	-2770.57651	-2769.683432	-2769.626898	-2769.625954	-2769.77536	-61.38	-18.95
TS8	-2770.568572	-2769.673261	-2769.617824	-2769.616879	-2769.762496	-55.57	-10.71
I7 (1d')	-2770.625361	-2769.728324	-2769.672808	-2769.671863	-2769.818516	-90.78	-46.59
1d	-2770.629374	-2769.732021	-2769.676421	-2769.675477	-2769.822355	-93.09	-49.04
**Reaction 2 + PhNCO**							
Compound	E_{electr} [A.U.]	ε_0 + E_{ZPE} [A.U.]	ε_0 + E_{therm} [A.U.]	ε_0 + H [A.U.]	ε_0 + G [A.U.]	ΔH [kcalmol^{-1}]	ΔG [kcal mol^{-1}]
$TS1_A$	-2340.836071	-2339.896004	-2339.849221	-2339.848277	-2339.974226	0.63	17.12
$TS1_B$	-2340.836711	-2339.897358	-2339.850128	-2339.849184	-2339.977459	0.04	15.05
$I1_A$	-2340.836169	-2339.895813	-2339.848306	-2339.847362	-2339.976482	1.21	15.68
$I1_B$	-2340.848413	-2339.907493	-2339.860257	-2339.859313	-2339.987751	-6.44	8.46
$TS2_A$	-2340.820208	-2339.879787	-2339.833525	-2339.832581	-2339.957316	10.68	27.95
$TS2_B$	-2340.837533	-2339.896984	-2339.850492	-2339.849548	-2339.97552	-0.19	16.30
$I2_A$	-2340.889525	-2339.948358	-2339.901181	-2339.900237	-2340.029471	-32.65	-18.25
$I2_B$	-2340.893465	-2339.951915	-2339.904864	-2339.90392	-2340.031929	-35.01	-19.83
$TS3_{A1}$	-2740.730685	-2739.686251	-2739.631002	-2739.630058	-2739.778621	-31.71	-3.67
$TS3_{A2}$	-2740.729938	-2739.68546	-2739.630221	-2739.629276	-2739.777978	-31.20	-3.26
$I3_{A1}$	-2740.741144	-2739.694617	-2739.639516	-2739.638571	-2739.786226	-37.16	-8.54
$I3_{A2}$	-2740.741258	-2739.694629	-2739.639557	-2739.638612	-2739.785789	-37.18	-8.26
$TS4_{A1}$	-2740.737609	-2739.691207	-2739.636951	-2739.636006	-2739.780874	-35.51	-5.11
$TS4_{A2}$	-2740.733493	-2739.687467	-2739.633062	-2739.632118	-2739.778276	-33.02	-3.45

TS3$_B$	-2740.735503	-2739.688778	-2739.634479	-2739.633535	-2739.776518	-33.93	-2.32
I3$_B$	-2740.735822	-2739.688471	-2739.633729	-2739.632785	-2739.776676	-33.45	-2.42
TS4$_B$	-2740.728888	-2739.682576	-2739.628416	-2739.627472	-2739.770919	-30.05	1.26
2c	-2740.790427	-2739.744226	-2739.688983	-2739.688039	-2739.836783	-68.83	-40.91
2c' (I4)	-2740.781558	-2739.735148	-2739.680001	-2739.679057	-2739.82792	-63.08	-35.24
TS5	-3140.616919	-3139.467925	-3139.404656	-3139.403712	-3139.571528	-58.83	-17.11
I5	-3140.624188	-3139.472996	-3139.409996	-3139.409051	-3139.574301	-62.25	-18.88
TS6$_A$	-3140.623186	-3139.471958	-3139.409823	-3139.408879	-3139.571702	-62.14	-17.22
2d	-3140.666058	-3139.513732	-3139.451684	-3139.45074	-3139.612957	-88.95	-43.64
TS6$_B$	-3140.623154	-3139.472264	-3139.409947	-3139.409002	-3139.573566	-62.22	-18.41
2d'	-3140.654944	-3139.503794	-3139.441197	-3139.440253	-3139.605819	-82.23	-39.06

TABEL S11. SELECTED COMPUTATIONAL PARAMETERS OBTAINED FOR SYSTEMS INVOLVED IN H_2 ACTIVATION (IN ATOMIC UNITS A.U.): $\mathbf{E_0}$ - ELECTRONIC ENERGY; $\mathbf{E_{0+...}}$ - SUM OF ELECTRONIC AND: $\mathbf{E_{ZPE}}$ - ZERO-POINT ENERGIES, $\mathbf{E_{THERM}}$ - THERMAL ENERGIES, **H** – THERMAL ENTHALPIES, **G** - THERMAL FREE ENERGIES CALCULATED AT TPSSTPSS//6-31+G(d,p) LEVEL OF THEORY INCLUDING SOLVATION EFFECTS AS SINGLE POINT CALCULATIONS USING THE PCM-SMD MODEL.

**Substrates**							
Compound	E_{electr} [A.U.]	$\varepsilon_0 + E_{ZPE}$ [A.U.]	$\varepsilon_0 + E_{therm}$ [A.U.]	ε_0 + H [A.U.]	ε_0 + G [A.U.]	ΔH [kcal mol^{-1}]	ΔG [kcal mol^{-1}]
1	-1570.969239	-1570.391064	-1570.359540	-1570.358596	-1570.449425	-	-
1'	-1570.968493	-1570.389768	-1570.356676	-1570.355732	-1570.451564	-	-
H_2	-1.178361	-1.168174	-1.165813	-1.164869	-1.179656	-	-
tBu_2PH	-657.780081	-657.530386	-657.517101	-657.516157	-657.567946	-	-
**Reaction 1' + H_2 - path A**							
Compound	E_{electr} [A.U.]	$\varepsilon_0 + E_{ZPE}$ [A.U.]	$\varepsilon_0 + E_{therm}$ [A.U.]	ε_0 + H [A.U.]	ε_0 + G [A.U.]	ΔH [kcal mol^{-1}]	ΔG [kcal mol^{-1}]
TS1	-1572.114778	-1571.519967	-1571.486931	-1571.485986	-1571.579690	24.00	31.63
I1	-914.378965	-914.034733	-914.015003	-914.014059	-914.083241	-4.32	-14.16
TS2$_A$	-1828.767304	-1828.076950	-1828.038530	-1828.037586	-1828.145278	-14.71	-14.73
TS2$_B$	-1828.768663	-1828.077576	-1828.038515	-1828.037571	-1828.145492	-14.70	-14.87
I2	-1828.787459	-1828.095733	-1828.055866	-1828.054922	-1828.165219	-25.81	-27.50
TS3	-1828.786320	-1828.094480	-1828.055437	-1828.054493	-1828.162682	-25.54	-25.88
1a	-1828.838144	-1828.143820	-1828.106783	-1828.105839	-1828.206422	-58.42	-53.89
**Reaction 1 + H_2 - path B**							
Compound	E_{electr} [A.U.]	$\varepsilon_0 + E_{ZPE}$ [A.U.]	$\varepsilon_0 + E_{therm}$ [A.U.]	ε_0 + H [A.U.]	ε_0 + G [A.U.]	ΔH [kcal mol^{-1}]	ΔG [kcal mol^{-1}]
TS1	-1572.109197	-1571.516822	-1571.484164	-1571.483220	-1571.576872	23.94	34.80
I1$_A$	-1572.191303	-1571.593834	-1571.561085	-1571.560140	-1571.654158	-25.32	-14.69
TS2	-1572.187974	-1571.589692	-1571.557942	-1571.556997	-1571.648010	-23.31	-10.75
1a"	-1572.194188	-1571.596409	-1571.563839	-1571.562895	-1571.656897	-27.08	-16.44
**Reaction 1 + H_2 - path C**							
Compound	E_{electr} [A.U.]	$\varepsilon_0 + E_{ZPE}$ [A.U.]	$\varepsilon_0 + E_{therm}$ [A.U.]	ε_0 + H [A.U.]	ε_0 + G [A.U.]	ΔH [kcal mol^{-1}]	ΔG [kcal mol^{-1}]
TS1	-1572.101974	-1571.507402	-1571.473969	-1571.473025	-1571.569538	30.47	39.50
I1	-914.378965	-914.034733	-914.015003	-914.014059	-914.083241	-6.16	-12.79
TS2$_A$	-1828.767304	-1828.076950	-1828.038530	-1828.037586	-1828.145278	-18.38	-11.99
TS2$_B$	-1828.768663	-1828.077576	-1828.038515	-1828.037571	-1828.145492	-18.37	-12.13
I2	-1828.787459	-1828.095733	-1828.055866	-1828.054922	-1828.165219	-29.20	-23.14
TS3	-1828.786320	-1828.094480	-1828.055437	-1828.054493	-1828.162682	-18.37	-12.13
1a	-1828.838144	-1828.143820	-1828.106783	-1828.105839	-1828.206422	-62.08	-51.15

Philicity of phosphorus and boron centres

TABLE S12. VALUES OF NUCLEOPHILIC (f_N), ELECTROPHILIC (f_E) FUKUI FUNCTIONS AND DUAL DESCRIPTOR (Δf) CALCULATED USING PARTIAL CHARGES DERIVED VIA HIRSHFELD POPULATION ANALYSIS

**Reaction 1' + H_2 - path A**												
Compound	P1			P2			B1			B2		
	f_N	f_E	Δf	f_N	f_E	Δf	f_N	f_E	Δf	f_N	f_E	Δf
1	0.166	0.037	-0.129	0.098	0.102	0.003	0.026	0.117	0.092	-	-	-
1'	0.141	0.058	-0.082	0.141	0.058	-0.082	0.027	0.136	0.110	-	-	-
I1 (1a')	0.242	0.095	-0.148	-	-	-	0.052	0.170	0.118	-	-	-
I2	0.231	-0.007	-0.238	-0.001	0.020	0.021	0.022	0.013	-0.009	-0.001	0.156	0.157
1a	0.015	0.003	-0.012	0.041	0.004	-0.038	0.016	0.004	-0.012	0.016	0.004	-0.012
**Reaction 1' + CO_2**												
Compound	P1			P2			B1			B2		
	f_N	f_E	Δf	f_N	f_E	Δf	f_N	f_E	Δf	f_N	f_E	Δf
I1	0.052	0.022	-0.030	0.104	0.105	0.001	0.038	0.096	0.059	-	-	-
I2a	0.091	0.035	-0.055	0.189	0.063	-0.127	0.035	0.085	0.050	-	-	-
I2b	0.096	0.043	-0.053	0.190	0.056	-0.134	0.027	0.096	0.069	-	-	-
I2c	0.078	0.022	-0.056	0.193	0.032	-0.161	0.014	0.117	0.103	-	-	-
I3	0.114	0.030	-0.085	0.114	0.030	-0.085	0.003	0.068	0.065	-	-	-
I4	0.037	0.000	-0.037	0.025	0.076	0.051	0.025	0.001	-0.023	0.016	0.040	0.024
I5	0.026	0.074	0.048	0.081	-0.002	-0.083	0.015	0.000	-0.016	0.007	0.066	0.059
I6	0.074	0.058	-0.015	0.029	0.020	-0.009	0.008	0.005	-0.004	0.009	0.014	0.005
$I1_{PhBO}$	0.145	0.038	-0.107	0.154	0.034	-0.120	0.020	0.037	0.017	0.024	0.077	0.053
$I2a_{PhBO}$	0.184	0.041	-0.143	0.086	0.026	-0.060	0.029	0.055	0.026	0.006	0.045	0.040
$I2b_{PhBO}$	0.191	0.038	-0.153	0.082	0.019	-0.063	0.030	0.063	0.033	0.005	0.061	0.057
$I3_{PhBO}$	0.014	0.065	0.052	0.269	0.011	-0.258	0.035	-0.001	-0.036	0.011	0.063	0.052
1b	0.083	0.062	-0.021	0.064	0.062	-0.002	0.008	0.011	0.004	0.008	0.011	0.003
PhBO	-	-	-	-	-	-	0.038	0.139	0.100	-	-	-
**Reaction 2 + CO_2**												
Compound	P1			P2			B			B2		
	f_N	f_E	Δf	f_N	f_E	Δf	f_N	f_E	Δf	f_N	f_E	Δf
2	0.138	0.038	-0.100	0.157	0.035	-0.121	0.026	0.079	0.053	-	-	-
$I1_A$	0.076	0.053	-0.023	0.170	0.023	-0.147	0.013	0.024	0.010	-	-	-
$I1_B$	0.091	0.069	-0.021	0.181	0.021	-0.160	0.017	0.019	0.002	-	-	-
2b	0.154	0.047	-0.106	0.133	0.050	-0.083	0.002	0.022	0.020	-	-	-
**Reaction 1' + PhNCO**												
Compound	P1			P2			B			B2		
	f_N	f_E	Δf	f_N	f_E	Δf	f_N	f_E	Δf	f_N	f_E	Δf
$I1_A$	0.010	0.022	0.012	0.043	0.104	0.062	0.019	0.094	0.075	-	-	-
$I1_B$	0.010	0.024	0.014	0.048	0.105	0.057	0.018	0.096	0.078	-	-	-
$I2_A$	0.082	0.034	-0.048	0.146	0.037	-0.108	0.016	0.106	0.090	-	-	-
$I2_B$	0.076	0.023	-0.053	0.130	0.066	-0.064	0.021	0.094	0.073	-	-	-
I3	0.060	0.056	-0.004	0.217	0.018	-0.199	0.027	0.002	-0.025	-	-	-
I4	0.028	0.062	0.035	0.041	0.005	-0.036	0.005	0.006	0.002	-	-	-
I5	0.086	0.012	-0.074	0.023	0.044	0.021	0.004	0.012	0.008	-	-	-
I6	0.084	0.019	-0.065	0.084	0.019	-0.065	0.005	0.071	0.066	-	-	-
I7	0.105	0.009	-0.097	0.046	0.042	-0.004	0.004	0.006	0.002	-	-	-
1d	0.090	0.007	-0.083	0.024	0.041	0.017	0.002	0.008	0.005	-	-	-
**Reaction 2 + PhNCO**												
Compound	P1			P2			B1			B2		
	f_N	f_E	Δf	f_N	f_E	Δf	f_N	f_E	Δf	f_N	f_E	Δf
$I1_A$	0.036	0.035	-0.001	0.139	0.038	-0.100	0.010	0.099	0.089	-	-	-
$I1_B$	0.012	0.041	0.028	0.099	0.039	-0.060	0.005	0.104	0.099	-	-	-
$I2_A$	0.095	0.059	-0.036	0.142	0.018	-0.123	0.007	0.002	-0.006	-	-	-
$I2_B$	0.085	0.033	-0.052	0.158	0.022	-0.136	0.008	0.022	0.014	-	-	-
$I3_A$	0.048	0.063	0.015	0.008	0.012	0.003	0.001	0.005	0.004	-	-	-
$I3_B$	0.040	0.039	0.000	0.026	0.010	-0.017	0.001	0.036	0.035	-	-	-
2c	0.039	0.022	-0.018	0.088	0.024	-0.064	0.004	0.010	0.006	-	-	-
I4 (2c')	0.060	0.037	-0.023	0.073	0.016	-0.057	0.001	0.013	0.012	-	-	-
I5	0.047	0.020	-0.027	0.019	0.034	0.015	-0.036	0.015	0.051	-	-	-
2d	0.053	0.011	-0.041	0.016	0.068	0.052	0.014	0.004	-0.010	-	-	-
2d'	0.071	0.011	-0.061	0.027	0.066	0.039	0.003	0.005	0.002	-	-	-

A Gibbs free-energy profiles

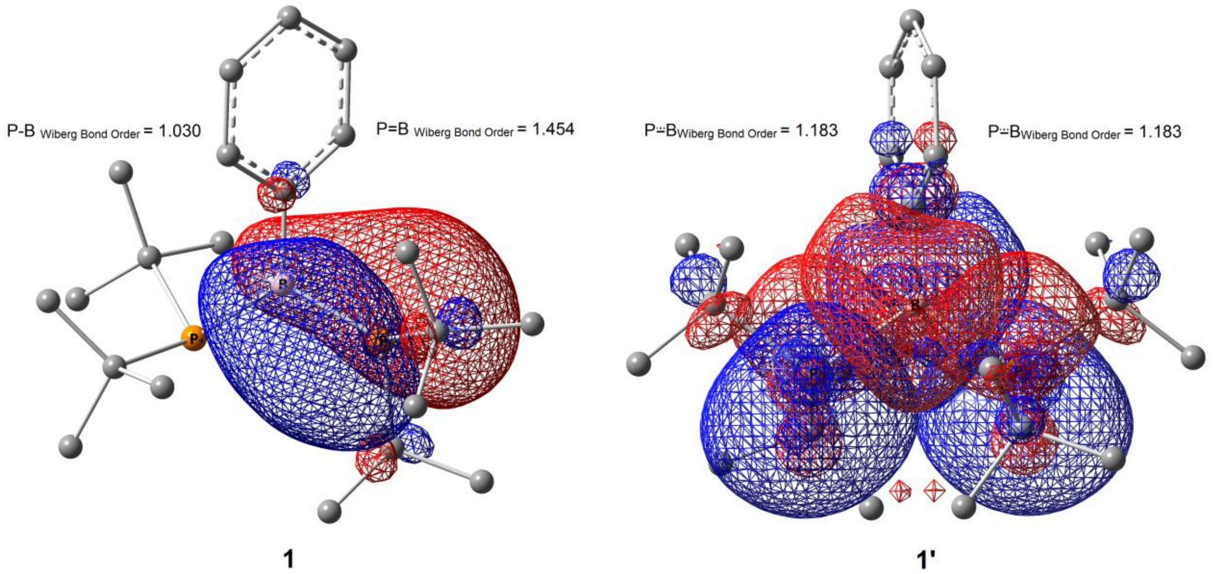

FIG. S42. VISUALIZATION OF NBO ORBITALS INVOLVED IN INTERACTION WITH H_2 MOLECULE TOGETHER WITH PB WIBERG BOND ORDERS

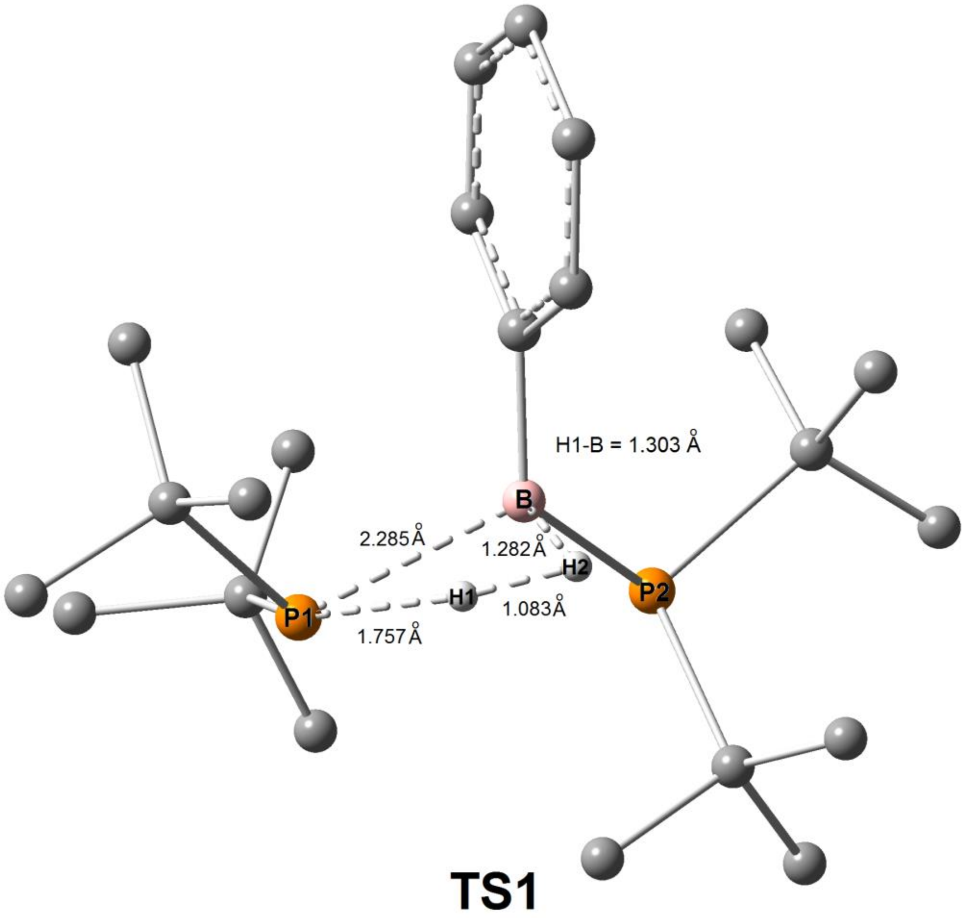

FIG. S43. AN OPTIMIZED STRUCTURE OF TS1 IN THE REACTION OF **1'** WITH H_2

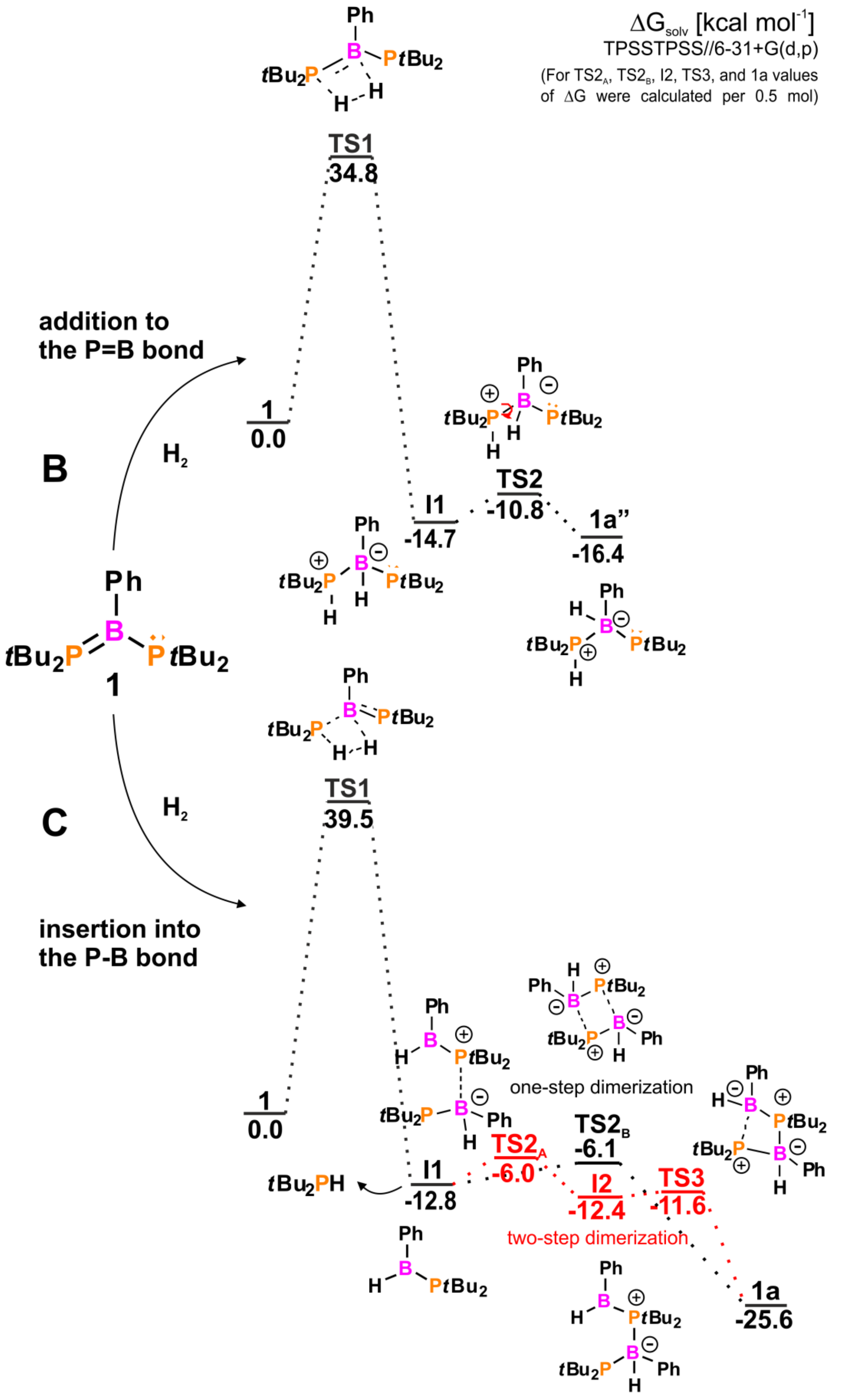

FIG. S44. A GIBBS FREE-ENERGY PROFILE OF REACTION OF **1** WITH **H_2** (AN ALTERNATIVE REACTION MECHANISMS **B** AND **C**)

Optimized structures, Hirshfeld atomic charges and Cartesian coordinates

Hirshfeld atomic charges for all optimized structures of substrates, intermediates, transition states and products were presented in Figures S43-S55. Hydrogen atoms were omitted for clarity.

Substrates

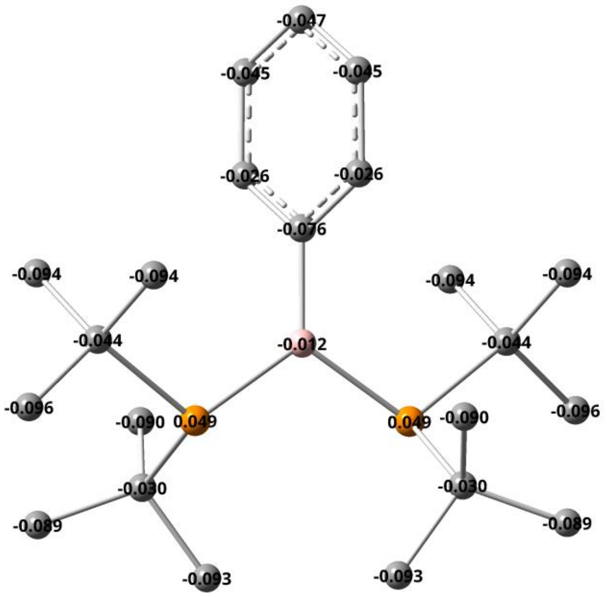

FIG. S45. OPTIMIZED STRUCTURE **1'** (THE LOWEST-ENERGRTIC CONFORMER)

B	0.00000000	0.15216700	-0.00000100
C	0.00000100	1.72426100	0.00000000
P	-1.56769100	-0.90772900	0.34327800
C	0.56805200	2.46264300	1.06464500
H	1.00655500	1.92942600	1.90662300
P	1.56769200	-0.90773100	-0.34328100
C	0.55305900	3.86340100	1.07691000
H	0.98214400	4.40084400	1.92149200
C	-0.00000100	4.57146400	0.00000200
H	-0.00000100	5.66006900	0.00000300
C	-0.55305900	3.86340200	-1.07690700
H	-0.98214400	4.40084600	-1.92148900
C	-0.56805100	2.46264400	-1.06464400
H	-1.00655300	1.92942700	-1.90662200
C	-1.95144100	-1.80072600	-1.33421400
C	-3.40739300	-2.31041900	-1.34674200
H	-3.61329400	-2.96309700	-0.48988200
H	-3.55693700	-2.89974200	-2.26409900
H	-4.13958200	-1.49597700	-1.35640100
C	-1.71781400	-0.87875200	-2.55032400
H	-2.33639200	0.02478600	-2.49631800
H	-1.97711400	-1.41356200	-3.47818900
H	-0.66371100	-0.58048900	-2.61416800
C	-1.02896900	-3.03156800	-1.44171900
H	0.02984100	-2.74648600	-1.41909500
H	-1.22743700	-3.53814000	-2.39911200
H	-1.22122300	-3.74089400	-0.62736800
C	-3.04155500	0.19096000	0.89603600

C	-2.50663800	1.05474000	2.06320700
H	-1.98814700	0.43891500	2.81126800
H	-3.35770200	1.54388600	2.56069200
H	-1.82306000	1.83448800	1.71834700
C	-4.12896800	-0.73762700	1.49230100
H	-4.62437900	-1.35201300	0.73519000
H	-4.89993700	-0.11719700	1.97455500
H	-3.70156400	-1.40523200	2.25127400
C	-3.66534400	1.11124800	-0.17046100
H	-2.93368000	1.83136000	-0.54991400
H	-4.49654800	1.67769700	0.27871500
H	-4.07274700	0.54638000	-1.01714200
C	3.04155700	0.19096100	-0.89603400
C	2.50663800	1.05473800	-2.06320700
H	1.98815100	0.43891100	-2.81126700
H	3.35770300	1.54388700	-2.56069200
H	1.82305800	1.83448500	-1.71834800
C	3.66534100	1.11125300	0.17046200
H	2.93367400	1.83136200	0.54991500
H	4.49654300	1.67770500	-0.27871400
H	4.07274600	0.54638500	1.01714300
C	4.12897300	-0.73762300	-1.49229700
H	4.62438800	-1.35200300	-0.73518500
H	4.89993800	-0.11719000	-1.97455600
H	3.70157100	-1.40523200	-2.25126700
C	1.95143900	-1.80073000	1.33421100
C	1.71780900	-0.87875800	2.55032300
H	2.33638200	0.02478400	2.49631700
H	1.97711300	-1.41356800	3.47818700
H	0.66370500	-0.58050100	2.61416800
C	3.40739200	-2.31042100	1.34674200
H	3.61329200	-2.96310400	0.48988600
H	3.55693800	-2.89973800	2.26410200
H	4.13958000	-1.49597800	1.35639400
C	1.02897000	-3.03157300	1.44171500
H	-0.02984100	-2.74649400	1.41909900
H	1.22744400	-3.53815000	2.39910500
H	1.22122100	-3.74089500	0.62736000

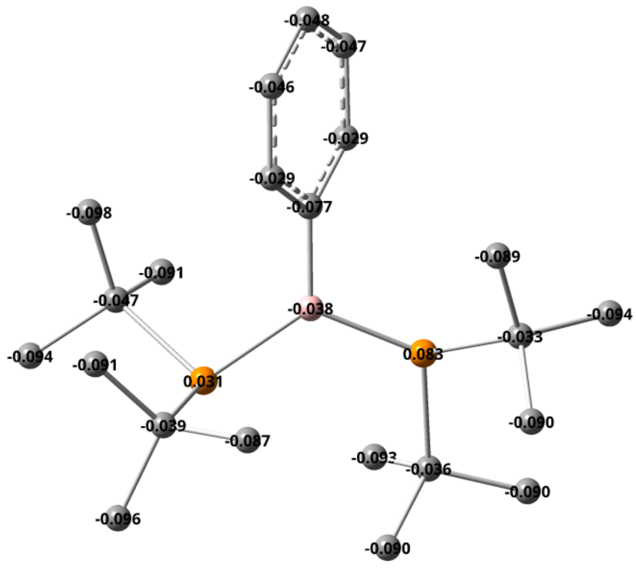

FIG. S46. OPTIMIZED STRUCTURE **1** (X-RAY)

B	-0.02917600	0.25829900	-0.12264200
C	-0.44569400	1.77388900	-0.09464200
P	-1.26094500	-1.25666900	-0.18133000
C	-1.12838500	2.36815100	0.99326500
H	-1.36165000	1.76740400	1.87014800
P	1.76442100	-0.07647000	-0.48819900
C	-1.49376600	3.71792700	0.98390300
H	-2.00409200	4.14599800	1.84574600
C	-1.21318800	4.51778800	-0.13580000
H	-1.51096700	5.56482700	-0.15092800
C	-0.54800900	3.95400600	-1.23282600
H	-0.32418200	4.56278700	-2.10793100
C	-0.15845500	2.60723000	-1.20411200
H	0.36931300	2.18091000	-2.05640700
C	-2.78336700	-0.70002100	-1.20662700
C	-3.78192200	-1.87856200	-1.23128100
H	-3.28326600	-2.81610600	-1.51024000
H	-4.57593100	-1.67627600	-1.96673400
H	-4.26445700	-2.02371500	-0.25618200
C	-3.52356900	0.60070200	-0.83623300
H	-3.98398800	0.55460400	0.15474300
H	-4.32981400	0.76762700	-1.56886900
H	-2.85759100	1.46743400	-0.86699100
C	-2.19013900	-0.52501800	-2.62693700
H	-1.45971900	0.29497900	-2.65353000
H	-2.99705700	-0.28082500	-3.33467300
H	-1.69371100	-1.44100500	-2.97259300
C	-1.72786200	-1.52825400	1.67977100
C	-0.50598100	-1.16445900	2.55173800
H	0.39881000	-1.68737800	2.22320000
H	-0.70546800	-1.45126600	3.59633400
H	-0.29636300	-0.08675400	2.52841000
C	-2.01196000	-3.04088600	1.83567500
H	-2.85943500	-3.35526400	1.21426100
H	-2.25521700	-3.26913800	2.88568200
H	-1.14049700	-3.63954300	1.54365300
C	-2.95206800	-0.74366000	2.18596700
H	-2.82382000	0.33814900	2.08148200
H	-3.10210400	-0.96825800	3.25429200
H	-3.86764800	-1.03327300	1.65792200
C	3.03113400	0.87727900	0.58649600
C	2.37345300	2.14699500	1.16478500
H	2.00875300	2.82100500	0.38335500
H	3.13407200	2.68164000	1.75360800
H	1.53462300	1.90538400	1.82578700
C	3.48452400	0.00228600	1.77760700
H	2.62854500	-0.28515900	2.39990100
H	4.18260000	0.58408000	2.39919400
H	4.00436300	-0.90777500	1.46112900
C	4.24699000	1.31633900	-0.26146000
H	4.80243200	0.46871200	-0.67293200
H	4.93654300	1.89388800	0.37282000
H	3.92786500	1.95640800	-1.09272900
C	2.30758300	-1.86902200	-0.85817500
C	1.99523100	-2.87421200	0.26995000
H	2.47050700	-2.59495900	1.21720600
H	2.36811400	-3.87002100	-0.01733200

H	0.91210600	-2.94583600	0.41967600
C	3.81417800	-1.91455900	-1.19899700
H	4.06476200	-1.21820800	-2.00889800
H	4.05676900	-2.93110400	-1.54177700
H	4.45487400	-1.69784500	-0.33710200
C	1.53864600	-2.28715600	-2.13646000
H	0.45761400	-2.29411700	-1.96166600
H	1.85036400	-3.30505500	-2.41698200
H	1.76343800	-1.61206000	-2.97155800

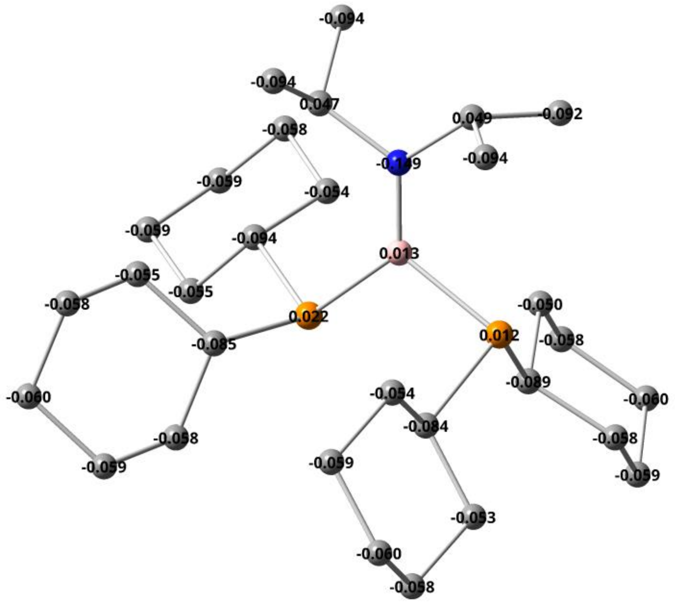

FIG. S47. OPTIMIZED STRUCTURE **2**

P	0.76337400	0.13949500	-0.69634800
P	-2.07693800	-0.80076100	0.66490100
N	0.38650300	-0.47530700	2.29163100
C	-2.49450300	0.84531500	-0.18283500
H	-1.67863100	1.09018300	-0.88444600
C	-3.79913000	0.80608300	-1.00256500
H	-3.70899200	0.07384800	-1.81492500
H	-4.62872700	0.47605900	-0.35635700
C	0.84795100	2.05079300	-0.59714800
H	0.00758600	2.34036300	0.05192000
C	-1.90191800	-1.99940200	-0.79113800
H	-1.52972100	-1.43089400	-1.65904300
C	2.58008400	-0.39769800	-0.70358200
H	3.16943000	0.12899100	0.06494200
C	-0.86487100	-3.08983900	-0.45042100
H	0.09542900	-2.62876700	-0.18376200
H	-1.20503300	-3.64834700	0.43698200
C	1.76607200	-0.00689100	2.59956300
H	2.18203700	0.28201200	1.63315500
C	2.12799300	2.65094700	0.01843700
H	2.29226400	2.25631000	1.02796600
H	3.00190700	2.36124300	-0.58443500
C	-3.24337800	-2.68082400	-1.14182200
H	-3.59843600	-3.23460900	-0.25773900
H	-4.01623800	-1.93801400	-1.37419400
C	2.68428100	-1.91420400	-0.43975700

H	2.22231300	-2.16774300	0.52372500
H	2.11099200	-2.44647100	-1.21507600
C	-0.34048000	-0.96592500	3.51304700
H	0.39923100	-0.92243400	4.32131300
C	0.58844700	2.65412300	-1.99780000
H	1.38493800	2.32673900	-2.68413400
H	-0.35202600	2.25340900	-2.40084900
C	4.68191300	-0.54473000	-2.13511100
H	5.10735300	-0.32207700	-3.12433400
H	5.27079000	0.02607300	-1.39843300
C	-2.57418800	1.94076200	0.90318200
H	-3.35782500	1.66461400	1.62683000
H	-1.63090700	1.98318800	1.46940600
C	-1.50428400	-0.04869000	3.92435000
H	-1.17374700	0.99498800	3.98632800
H	-1.87649200	-0.35445800	4.91192000
H	-2.32757500	-0.11869200	3.20583800
C	-2.00409700	-4.71920200	-2.02898300
H	-1.86156900	-5.37437100	-2.90053400
H	-2.35437100	-5.35796100	-1.20171100
C	3.21388700	-0.07683700	-2.07732500
H	2.62803500	-0.58152100	-2.86140300
H	3.15863300	0.99987600	-2.28689400
C	-4.12321100	2.19027100	-1.60099200
H	-3.33420500	2.46090500	-2.32157500
H	-5.06669600	2.14158800	-2.16381400
C	-3.07515900	-3.65345300	-2.32650300
H	-4.03662100	-4.13476500	-2.55812300
H	-2.77924000	-3.08169400	-3.22107400
C	2.05397400	4.19071800	0.06817500
H	1.22647700	4.49137200	0.73143300
H	2.97875300	4.59412600	0.50631700
C	1.82017800	4.78026200	-1.33350400
H	2.68523000	4.54102600	-1.97350100
H	1.75360500	5.87638100	-1.28219500
C	4.80445200	-2.04796100	-1.82637800
H	5.86022900	-2.35448500	-1.82829600
H	4.30278800	-2.62048200	-2.62318500
C	2.66477200	-1.10731200	3.19440900
H	2.38373200	-1.36352800	4.22425600
H	3.70322900	-0.75259100	3.21542200
H	2.62604800	-2.01922100	2.58907400
C	-2.89840000	3.32071600	0.30346800
H	-2.96906600	4.07262600	1.10310200
H	-2.07235000	3.63017100	-0.35596100
C	-0.66987500	-4.06469100	-1.62545200
H	-0.26088700	-3.50685100	-2.48307600
H	0.06937400	-4.83511800	-1.35985600
C	-0.76548800	-2.43587800	3.37473000
H	-1.56747300	-2.53993500	2.63569700
H	-1.13638500	-2.80468000	4.34067700
H	0.08518200	-3.05586300	3.06665300
C	4.14804800	-2.39072400	-0.47651400
H	4.70809900	-1.90172300	0.33730300
H	4.19581100	-3.47385300	-0.29216500
C	0.54459300	4.19404100	-1.96323300
H	0.40045100	4.58753500	-2.97988100

H	-0.32630900	4.51715500	-1.37057500
C	1.74052500	1.24396900	3.49692800
H	1.11091700	2.02408000	3.05262500
H	2.75840800	1.63926100	3.61162700
H	1.35587800	1.01831600	4.50018500
C	-4.20200200	3.27490800	-0.51244800
H	-5.04374500	3.05080800	0.16282600
H	-4.40593000	4.25643800	-0.96397800
B	-0.15502700	-0.38685500	0.99232800

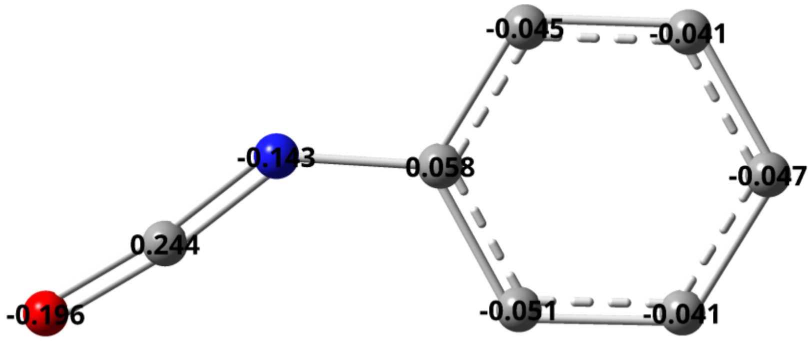

FIG. S48. OPTIMIZED STRUCTURE **PhNCO**

C	-2.53571100	-0.07590400	-0.00001100
O	-3.65752800	0.31567700	-0.00003400
N	-1.44921900	-0.62815700	0.00004100
C	-0.09179700	-0.26949800	0.00002000
C	0.31964200	1.07754800	0.00002400
C	0.86189500	-1.30157800	0.00000600
C	1.68529100	1.38091400	0.00000100
H	-0.42618900	1.87018900	0.00004300
C	2.22494600	-0.98335200	-0.00001900
H	0.52357800	-2.33462000	0.00000800
C	2.64298300	0.35529700	-0.00002000
H	1.99991100	2.42277900	0.00000100
H	2.96057800	-1.78522800	-0.00003800
H	3.70338700	0.59800000	-0.00003800

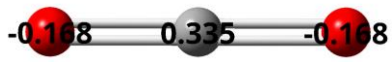

FIG. S49. OPTIMIZED STRUCTURE $\mathbf{CO_2}$

C	0.00000000	0.00000000	0.00000000
O	0.00000000	0.00000000	1.17870200
O	0.00000000	0.00000000	-1.17870200

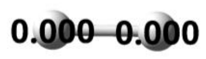

FIG. S50. OPTIMIZED STRUCTURE **H_2**

H	0.00000000	0.00000000	0.37033900
H	0.00000000	0.00000000	-0.37033900

Formation of 1a

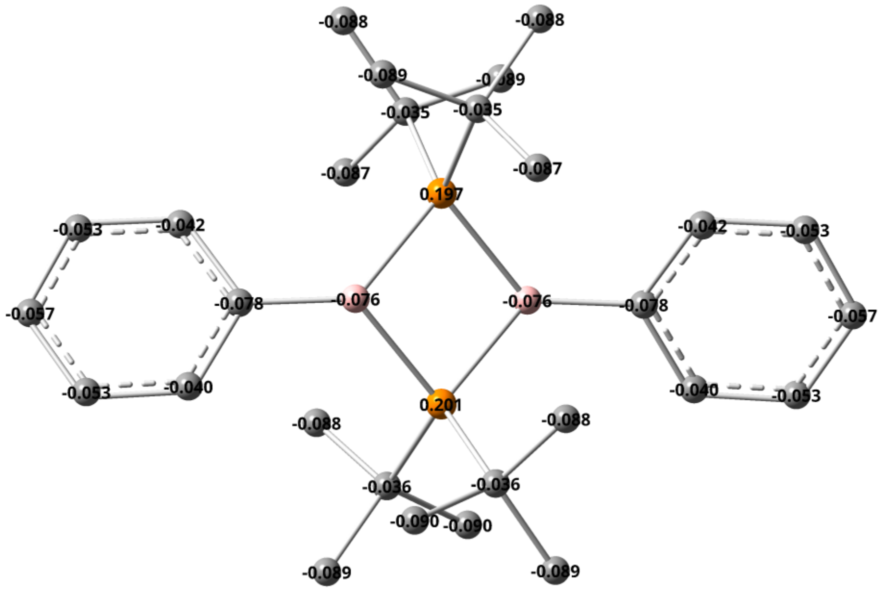

FIG. S51. OPTIMIZED STRUCTURE **1A**

P	-0.00000700	1.47264400	-0.00000200
C	0.35817300	2.60942200	1.50953600
C	1.08001000	1.79036900	2.60150000
H	0.41914600	1.02547500	3.01841800
H	1.35828400	2.47128000	3.41927200
H	1.99065100	1.30875800	2.22994100
C	-1.00189000	3.07029500	2.08785700
H	-1.54626900	3.73094400	1.40636100
H	-0.82150800	3.62427000	3.02140300
H	-1.64153600	2.21035500	2.31943900
C	1.23941400	3.84239200	1.20769200
H	2.23267000	3.56249900	0.83990700
H	1.38039400	4.39627700	2.14734900
H	0.79059600	4.53152400	0.48787800
C	-0.35820200	2.60941600	-1.50954300
C	-1.08003500	1.79035100	-2.60149700
H	-0.41916900	1.02545400	-3.01840600
H	-1.35831100	2.47125100	-3.41927700
H	-1.99067400	1.30873900	-2.22993300
C	1.00185700	3.07029600	-2.08787000
H	1.54623200	3.73095100	-1.40637700
H	0.82146800	3.62426800	-3.02141700
H	1.64150800	2.21036200	-2.31945300
C	-1.23945000	3.84238100	-1.20770200
H	-2.23270100	3.56248200	-0.83990700
H	-1.38044400	4.39625500	-2.14736400
H	-0.79063200	4.53152500	-0.48790000
C	-2.82479700	-0.03435000	0.47055200
C	-3.55370000	-1.18345800	0.85985900
H	-3.01774300	-2.03427100	1.27837200
C	-4.94634800	-1.26245800	0.73502000
H	-5.46736000	-2.16832300	1.04390500
C	-5.67065600	-0.17744900	0.21730600
H	-6.75300900	-0.23503800	0.11411900
C	-4.98108300	0.98279800	-0.15722700
H	-5.52624000	1.84026500	-0.55081700
C	-3.58511600	1.04335100	-0.02862200
H	-3.07297200	1.95798600	-0.31764300
B	-1.24170000	0.02157900	0.66115000
H	-0.92738800	0.03616000	1.83476900
P	0.00000700	-1.40757300	0.00000100
C	-0.61603200	-2.50940600	-1.43387800
C	-1.49774600	-1.63632700	-2.35093500
H	-0.91041500	-0.84372300	-2.81918500
H	-1.90316800	-2.27149400	-3.15243100
H	-2.33670300	-1.18666300	-1.80898100
C	0.57273900	-3.02712500	-2.27316100
H	1.23310700	-3.69167900	-1.70679300
H	0.18161400	-3.59360100	-3.13161800
H	1.16621300	-2.19117100	-2.66305300
C	-1.48374800	-3.69092700	-0.94804300
H	-2.35801600	-3.34012500	-0.38924400
H	-1.84913400	-4.23485000	-1.83166800
H	-0.92989900	-4.40431800	-0.32990900
C	0.61606300	-2.50939500	1.43388200
C	1.49776700	-1.63630300	2.35093600
H	0.91043200	-0.84369300	2.81917000

H	1.90318100	-2.27146000	3.15244500
H	2.33672900	-1.18664600	1.80898400
C	-0.57270300	-3.02712400	2.27316700
H	-1.23307000	-3.69167700	1.70679600
H	-0.18157300	-3.59360600	3.13161800
H	-1.16617800	-2.19117600	2.66306600
C	1.48379200	-3.69090600	0.94804700
H	2.35804600	-3.34009400	0.38923200
H	1.84920000	-4.23481400	1.83167200
H	0.92994500	-4.40431200	0.32993000
C	2.82479600	-0.03432400	-0.47054900
C	3.58510900	1.04337600	0.02863300
H	3.07296300	1.95800700	0.31766200
C	4.98107600	0.98282800	0.15724000
H	5.52622900	1.84029500	0.55083700
C	5.67065500	-0.17741300	-0.21730300
H	6.75300800	-0.23499800	-0.11411600
C	4.94635200	-1.26242000	-0.73502700
H	5.46736900	-2.16828100	-1.04391800
C	3.55370400	-1.18342500	-0.85986600
H	3.01775000	-2.03423700	-1.27838600
B	1.24169800	0.02159300	-0.66115100
H	0.92739100	0.03616800	-1.83477100

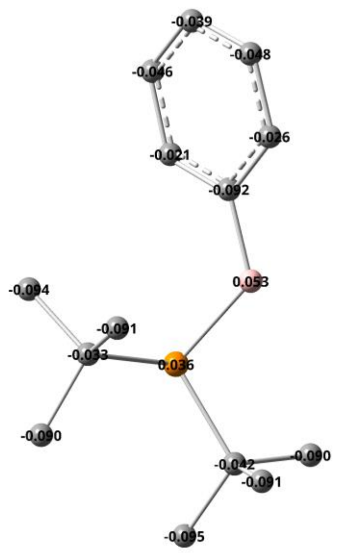

FIG. S52. OPTIMIZED STRUCTURE **1A_I1(1A')**

B	-0.40562600	1.14642600	0.09415000
C	-1.90883000	0.77209500	-0.02090900
P	0.99649600	0.09515400	-0.61829200
C	-2.45025400	0.05750000	-1.11993100
H	-1.79160200	-0.22522400	-1.94013700
C	-3.81279000	-0.25545300	-1.18876500
H	-4.20562800	-0.78944900	-2.05273200
C	-4.67122300	0.11441300	-0.14202500
H	-5.72964200	-0.13694400	-0.18897300
C	-4.16531800	0.82735900	0.95670100
H	-4.83139500	1.12891100	1.76374900

C	-2.80983500	1.17195500	1.00008900
H	-2.43159200	1.75173000	1.84197800
C	0.83691400	-1.63645400	0.22181900
C	2.18314300	-2.38542900	0.15591300
H	2.56698200	-2.44346500	-0.86999800
H	2.03022300	-3.41214000	0.52133100
H	2.94471300	-1.92065600	0.79205600
C	0.37081400	-1.54396200	1.69190700
H	1.05831000	-0.94653200	2.30078200
H	0.32183700	-2.55504700	2.12693600
H	-0.62887500	-1.09804000	1.75836800
C	-0.20262900	-2.44691900	-0.58244000
H	-1.19960700	-1.99871100	-0.52309500
H	-0.26564000	-3.46131700	-0.15943000
H	0.08308100	-2.52896100	-1.63861100
C	2.61164600	0.99198100	-0.11476600
C	2.45951700	2.43044900	-0.66640000
H	2.26281600	2.42374100	-1.74667600
H	3.39873500	2.97712900	-0.49416000
H	1.65252700	2.98072600	-0.17119100
C	3.82476900	0.35689400	-0.83422900
H	4.06442700	-0.64143700	-0.45651500
H	4.70909600	0.99291100	-0.67681300
H	3.64581400	0.28281700	-1.91435500
C	2.86156000	1.05159600	1.40474700
H	2.01622900	1.51812300	1.92564200
H	3.76401000	1.64845800	1.61048100
H	3.02269500	0.05379500	1.83060100
H	-0.14171500	2.20400100	0.59412600

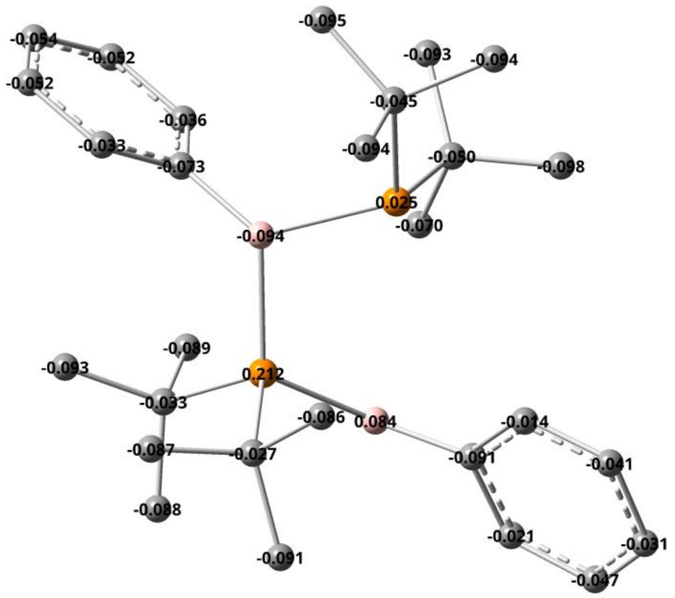

FIG. S53. OPTIMIZED STRUCTURE **1A_I2**

P	-0.23649800	1.71383400	0.29756500
C	-1.33510000	2.89024300	1.34356900
C	-1.05310300	2.50217500	2.81536200
H	-1.39011100	1.48111500	3.03157700
H	-1.59829600	3.18662200	3.48378500
H	0.01768200	2.57221800	3.04785100
C	-2.85455300	2.79315900	1.09914300
H	-3.12747800	3.05645100	0.07263100
H	-3.37842000	3.48831300	1.77587500
H	-3.22484700	1.78228200	1.29668400
C	-0.86746300	4.34889700	1.15027800
H	0.21597000	4.44414900	1.30273400
H	-1.37505700	4.99471700	1.88382500
H	-1.11182900	4.73157300	0.15232000
C	-0.25166600	2.30038100	-1.53273400
C	0.17556200	1.08458800	-2.37626200
H	1.14229900	0.68266600	-2.01009500
H	0.34817500	1.37582400	-3.42378100
H	-0.55809100	0.27429200	-2.35568400
C	0.84745900	3.37801700	-1.70418000
H	0.63211600	4.27800700	-1.11871000
H	0.91635800	3.67334000	-2.76363600
H	1.82604500	2.99454500	-1.39008800
C	-1.58377700	2.84943000	-2.08344200
H	-2.41400100	2.15503400	-1.92498300
H	-1.48856100	3.03572700	-3.16584800
H	-1.84334600	3.80363900	-1.61035200
C	-2.59400100	-0.42865800	-0.03886300
C	-3.52970100	-0.86852700	0.93292900
H	-3.19185600	-0.97671200	1.96423300
C	-4.86310100	-1.16455700	0.62190600
H	-5.54267100	-1.49680300	1.40624900
C	-5.32121600	-1.03313300	-0.69715100
H	-6.35535600	-1.26178500	-0.94991900
C	-4.42725500	-0.59673500	-1.68453500
H	-4.76460600	-0.48181200	-2.71433700
C	-3.09750800	-0.29970200	-1.35293400
H	-2.44291900	0.04554600	-2.14992500
B	-1.11617000	-0.06223900	0.48850200
H	-1.13066900	-0.18989600	1.69437100
P	0.38403800	-1.41726500	0.12729800
C	-0.23245900	-3.01242200	-0.75657500
C	-0.62569500	-2.60222800	-2.19621300
H	0.21369900	-2.18935900	-2.76464500
H	-0.97807100	-3.50200500	-2.72144600
H	-1.44795800	-1.88132300	-2.18930300
C	0.86160900	-4.09806200	-0.83055400
H	1.05255900	-4.54940800	0.14959500
H	0.51322200	-4.89922900	-1.49902800
H	1.80680500	-3.71316900	-1.23417600
C	-1.49399400	-3.61414800	-0.09659400
H	-2.31004300	-2.88929300	-0.04320300
H	-1.82843800	-4.45787500	-0.71836200
H	-1.30217800	-4.00016000	0.90772800
C	1.17579600	-1.85540900	1.82851800
C	1.41478300	-0.55253100	2.62261800
H	0.48461000	-0.01831600	2.82932000

H	1.87883400	-0.82877200	3.58140400
H	2.09670800	0.12343100	2.10145500
C	0.21209700	-2.74664100	2.63841700
H	0.17190800	-3.76821300	2.24485800
H	0.57964700	-2.80391900	3.67315600
H	-0.80255700	-2.33032800	2.66136300
C	2.54293900	-2.56146300	1.67826400
H	3.27795600	-1.91131100	1.19122400
H	2.92100000	-2.79443100	2.68472700
H	2.48200700	-3.50024100	1.12145500
C	3.17909800	-0.07248500	-0.68169900
C	3.28560400	1.05375800	0.17266600
H	2.36912200	1.47160800	0.59483400
C	4.51986800	1.66444100	0.41137000
H	4.57318800	2.53981700	1.05698300
C	5.68837500	1.15557900	-0.17880700
H	6.64973200	1.62839800	0.01664700
C	5.61186500	0.05090200	-1.04031900
H	6.51150600	-0.33552000	-1.51640600
C	4.36915700	-0.53471200	-1.30716300
H	4.31120700	-1.37475300	-1.99898100
B	1.90289900	-0.89506500	-0.99925700
H	1.94653300	-1.50762900	-2.02523900

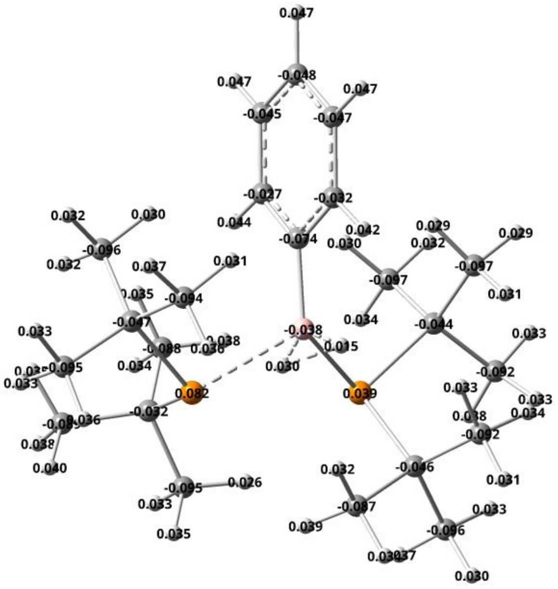

FIG. S54. OPTIMIZED STRUCTURE **1A_TS1**

B	-0.11711300	0.21649600	-0.45087100
C	0.34489900	1.72122900	-0.21128400
P	1.54921100	-1.34698800	-0.42786200
C	0.32253500	2.63328000	-1.29609200
H	-0.00163700	2.28093700	-2.27667900
P	-1.74289600	-0.66263900	0.33128700
C	0.68374700	3.97851900	-1.15276100
H	0.65364300	4.64190700	-2.01601800
C	1.07108500	4.47031300	0.10195900
H	1.34709900	5.51626200	0.22378200
C	1.09294200	3.59713500	1.19777500
H	1.39028600	3.96104900	2.18040000

C	0.74071100	2.25008700	1.03718700
H	0.78798300	1.59686500	1.90294600
C	1.55291700	-1.95199900	1.40573500
C	2.94564200	-2.56277100	1.68116700
H	3.21671100	-3.31383600	0.92884400
H	2.90594900	-3.06582500	2.65894600
H	3.73479600	-1.80486900	1.72986700
C	1.23770000	-0.89410700	2.47203500
H	1.96961200	-0.07916100	2.45669500
H	1.27147600	-1.36301700	3.46795800
H	0.23005400	-0.48701300	2.32959000
C	0.52020300	-3.10090500	1.48095800
H	-0.49873700	-2.72798200	1.33013600
H	0.57512900	-3.55777300	2.48154600
H	0.73833300	-3.87581100	0.73499800
C	3.14725200	-0.37384900	-0.85877200
C	2.83502400	0.33527700	-2.19805800
H	2.42918900	-0.36995800	-2.93803600
H	3.76673500	0.74991400	-2.61054100
H	2.13388300	1.16540500	-2.06683200
C	4.24981100	-1.42295300	-1.15627200
H	4.60186900	-1.93278400	-0.25469000
H	5.11289800	-0.90753400	-1.60481000
H	3.89478000	-2.18094700	-1.86604300
C	3.67151100	0.65317100	0.16057900
H	2.94595100	1.45013700	0.34145900
H	4.59223700	1.11232000	-0.23208100
H	3.92232300	0.18551300	1.12022600
C	-2.89913400	0.80381000	0.82368200
C	-2.37680900	1.27152000	2.20329000
H	-2.34922500	0.43938400	2.91876300
H	-3.04785700	2.04901900	2.60032100
H	-1.37538000	1.70638100	2.13071400
C	-2.97574200	2.01991000	-0.12080000
H	-2.01405400	2.53342000	-0.19626400
H	-3.70952900	2.73797100	0.27956300
H	-3.29886500	1.74219200	-1.12906700
C	-4.32488100	0.25099800	1.04548200
H	-4.82911500	0.01416900	0.10146100
H	-4.92516000	1.02078100	1.55411800
H	-4.31870200	-0.64664700	1.67743200
C	-2.53245300	-1.57510600	-1.18229500
C	-3.14059700	-0.67120700	-2.27053400
H	-4.01747200	-0.12252200	-1.90899000
H	-3.46361000	-1.28824500	-3.12398800
H	-2.40898600	0.05841400	-2.64458800
C	-3.60813000	-2.54606400	-0.63636000
H	-3.17541100	-3.22073500	0.11316000
H	-3.99892400	-3.15764500	-1.46443900
H	-4.45407300	-2.02686900	-0.17786200
C	-1.44545400	-2.45856800	-1.83699100
H	-0.70736600	-1.87824800	-2.40303800
H	-1.93078600	-3.14175700	-2.55064700
H	-0.90560200	-3.06565000	-1.10117100
H	-0.41543600	0.17980500	-1.69668700
H	0.43916200	-0.43140100	-1.43525400

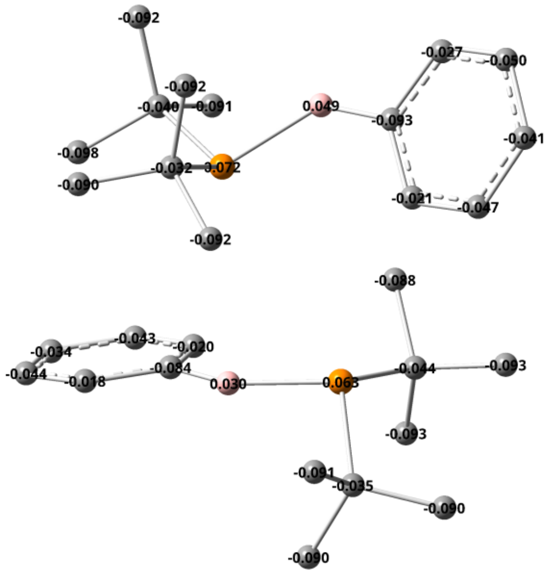

FIG. S55. OPTIMIZED STRUCTURE **1A_TS2_A**

B	1.39305700	1.33884300	0.75567600
C	2.74422500	0.79816400	0.19701500
P	-0.22019900	2.09524600	0.15530200
C	3.18137700	0.78014200	-1.15151500
H	2.51790900	1.12588600	-1.93815800
C	4.45614300	0.32810400	-1.50845400
H	4.75420200	0.32112800	-2.55586200
C	5.35198700	-0.11255700	-0.52153400
H	6.34376900	-0.46460200	-0.80088800
C	4.96178800	-0.09306900	0.82514900
H	5.64965700	-0.43012200	1.59914200
C	3.68057900	0.35265700	1.16976300
H	3.38176900	0.35841200	2.21735200
C	-0.27324800	2.56642000	-1.68699600
C	-1.65285700	3.14035100	-2.08897600
H	-2.47028500	2.52604900	-1.69060000
H	-1.72878400	3.13426400	-3.18686600
H	-1.79431400	4.17228300	-1.75532800
C	0.83784200	3.54481100	-2.11572100
H	0.69533400	4.52606700	-1.64504600
H	0.81092600	3.69158200	-3.20736000
H	1.83412400	3.18189200	-1.84017000
C	-0.15215500	1.20113500	-2.41246400
H	0.68019800	0.59045100	-2.05172700
H	-0.02091300	1.37142900	-3.49183400
H	-1.06713700	0.61383500	-2.26681500
C	-0.41975200	3.58661900	1.35306000
C	-0.88961600	2.98906300	2.69988300
H	-1.85532700	2.48006600	2.58975700
H	-1.00555700	3.80075300	3.43447200
H	-0.16704200	2.27021000	3.10206400
C	-1.50532300	4.56113900	0.85595500
H	-1.17652100	5.11761300	-0.02929600
H	-1.70993800	5.29556300	1.64964900
H	-2.44368900	4.04469800	0.61911000
C	0.89811100	4.36569300	1.56009800
H	1.66958100	3.72439100	2.00330800
H	0.72742200	5.21728600	2.23863600
H	1.28440100	4.75899000	0.61240700
H	1.33265500	1.29441600	1.95688900
B	-1.50972300	-2.05173700	-1.00613000

C	-2.90059200	-1.42068100	-0.72859800
P	0.05141100	-1.87320700	0.04632800
C	-4.05833400	-2.07385200	-1.22897000
H	-3.94015200	-2.99304300	-1.80298400
C	-5.34260600	-1.57129500	-0.99359500
H	-6.21502800	-2.10384800	-1.36963700
C	-5.50396400	-0.36327400	-0.29513600
H	-6.50128500	0.04067100	-0.12687700
C	-4.37458400	0.32877000	0.16762100
H	-4.49141600	1.27898600	0.68806300
C	-3.09459300	-0.20207600	-0.03056700
H	-2.22432200	0.36027800	0.31054700
C	-0.41738200	-2.39288800	1.84316400
C	-1.01482800	-1.15278600	2.54135400
H	-0.31931100	-0.30658000	2.52093900
H	-1.22952800	-1.40753700	3.59082600
H	-1.95423000	-0.84029800	2.07532300
C	-1.45411300	-3.53721200	1.88120100
H	-2.39530700	-3.22876800	1.41111000
H	-1.66716400	-3.80381900	2.92893900
H	-1.08978400	-4.43574200	1.37068800
C	0.84688500	-2.81685400	2.61706700
H	1.24614600	-3.77122100	2.25561300
H	0.58041500	-2.94989900	3.67651300
H	1.63581100	-2.05725800	2.55539300
C	1.29646900	-3.09082700	-0.76186800
C	0.82547600	-4.55798100	-0.75594600
H	0.75605000	-4.95376400	0.26494800
H	1.54884700	-5.18237400	-1.30424400
H	-0.15469100	-4.66446300	-1.23680800
C	2.69578800	-2.98115300	-0.11631700
H	3.02962400	-1.94013900	-0.06787600
H	3.41410700	-3.53983000	-0.73553800
H	2.73207400	-3.40393500	0.89152500
C	1.44557400	-2.59326000	-2.22044500
H	0.50490600	-2.63644600	-2.77910900
H	2.17788600	-3.23215200	-2.73682300
H	1.82181200	-1.56275300	-2.24315700
H	-1.40252900	-2.65453500	-2.03775900

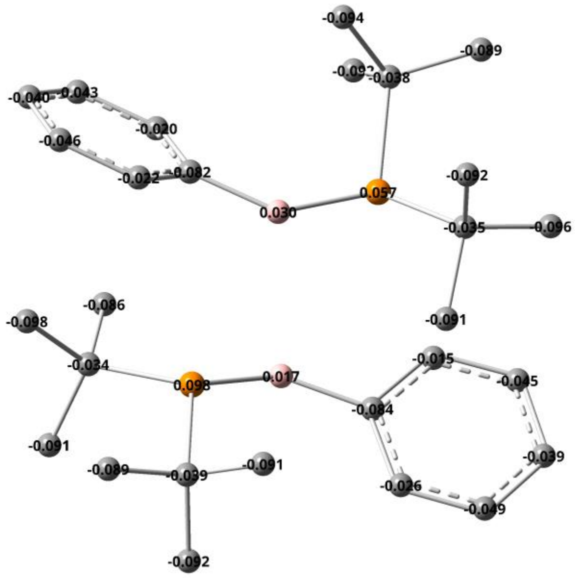

FIG. S56. OPTIMIZED STRUCTURE **1A_TS2_B**

P	1.95277800	-0.90965400	-0.01240200

C	2.74591200	-2.44344700	0.84240500
C	2.23658600	-2.43722800	2.30347600
H	2.58794600	-1.54237900	2.83228300
H	2.61766600	-3.32647200	2.82869800
H	1.14460900	-2.45362100	2.36886300
C	4.28286600	-2.33693400	0.91055100
H	4.74767000	-2.40428400	-0.07924200
H	4.65899800	-3.17989800	1.50998800
H	4.60793700	-1.40857500	1.39472400
C	2.37914500	-3.77123500	0.14568600
H	1.29859800	-3.94251800	0.13373600
H	2.85266700	-4.61044200	0.68031900
H	2.73714100	-3.78140200	-0.89104800
C	3.07287200	-0.17840200	-1.39221000
C	2.29320200	1.01081400	-2.00009000
H	1.38463600	0.69124400	-2.51682300
H	2.94297900	1.51310200	-2.73263600
H	2.01963900	1.74098000	-1.22956300
C	3.40102000	-1.20149400	-2.49813100
H	3.95291700	-2.06478600	-2.10614900
H	4.02628700	-0.72875100	-3.27254500
H	2.48283000	-1.56962700	-2.97270600
C	4.37383000	0.41488200	-0.80143100
H	4.15196500	1.08412200	0.03789600
H	4.87392200	1.00683600	-1.58294000
H	5.07985800	-0.34960600	-0.46841000
C	0.67751100	2.62543900	1.04281600
C	0.69220000	3.79148000	0.23990700
H	-0.20525300	4.08492400	-0.29751200
C	1.83141300	4.59769300	0.12638700
H	1.80520700	5.48887200	-0.49908000
C	3.00487400	4.25706900	0.81564500
H	3.89441000	4.87840500	0.72304900
C	3.02002800	3.11825300	1.63486400
H	3.92274900	2.85303700	2.18388500
C	1.87178000	2.32672300	1.75077900
H	1.89113000	1.44301400	2.38565000
B	-0.52542300	1.65437800	1.26273700
H	-0.45209100	0.97406800	2.24468800
P	-1.91418400	1.06501900	0.19442000
C	-2.47974800	2.11656200	-1.28574400
C	-1.26505400	2.17493200	-2.24228100
H	-1.03947300	1.17684400	-2.63472400
H	-1.50299200	2.83795800	-3.08787300
H	-0.36171700	2.55343000	-1.75557700
C	-3.63197300	1.42820200	-2.04973700
H	-4.56668600	1.42699600	-1.47743600
H	-3.81599100	1.98435900	-2.98114800
H	-3.37858600	0.39416900	-2.31342300
C	-2.92533700	3.54067200	-0.88465700
H	-2.14815200	4.07173000	-0.32589400
H	-3.15635800	4.12124800	-1.79176300
H	-3.82498600	3.51812900	-0.26056300
C	-3.37236100	0.38789500	1.22430200
C	-2.81007700	-0.17130900	2.54951900
H	-2.42566200	0.62369400	3.19701400
H	-3.62635500	-0.68664800	3.07719900

H	-2.00748400	-0.89602600	2.37398400
C	-4.40648700	1.48625300	1.55294500
H	-4.90631400	1.86261500	0.65272900
H	-5.18443000	1.06210800	2.20688000
H	-3.94010700	2.33178400	2.07291200
C	-4.06136900	-0.77580900	0.47362900
H	-3.34649600	-1.56575200	0.22628400
H	-4.83947300	-1.20585600	1.12243400
H	-4.54523800	-0.44685500	-0.45050100
C	-0.77701200	-2.38819900	-0.55736800
C	-1.05037000	-2.98151200	0.69860600
H	-0.48754600	-2.65937500	1.57029400
C	-2.04533600	-3.95151300	0.85988000
H	-2.23628900	-4.37416400	1.84530200
C	-2.80009000	-4.37519500	-0.24530200
H	-3.57221900	-5.13317900	-0.12378600
C	-2.56361300	-3.80139200	-1.50343600
H	-3.15430600	-4.11166000	-2.36430500
C	-1.58077800	-2.81410300	-1.64704500
H	-1.42136500	-2.35759400	-2.62376300
B	0.33709000	-1.34339100	-0.87743500
H	0.14475000	-0.70419200	-1.86998900

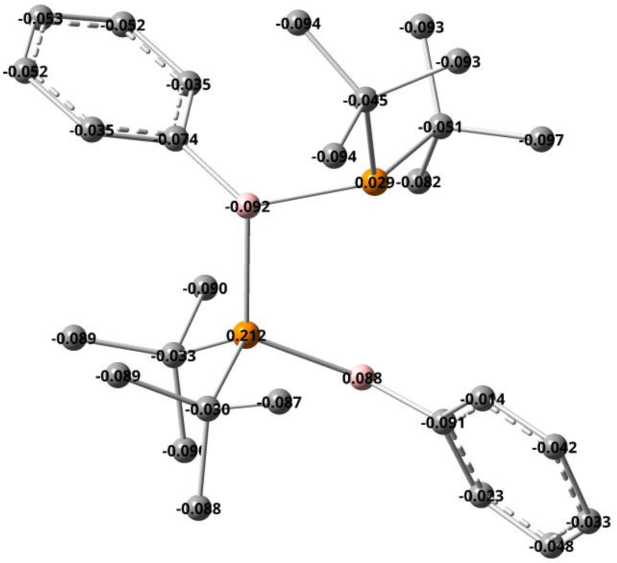

FIG. S57. OPTIMIZED STRUCTURE **1A_TS3**

P	-0.12935700	1.73828700	0.18267300
C	-1.03379000	2.91641800	1.40283700
C	-0.57945300	2.48976600	2.81957500
H	-0.89387500	1.46586900	3.05463500
H	-1.03315200	3.16106800	3.56532600
H	0.51239100	2.55093400	2.92115100
C	-2.57320400	2.84531900	1.33978600
H	-2.95914400	3.12626600	0.35454000
H	-3.00592000	3.53519000	2.08310300
H	-2.93100000	1.83474300	1.56429400
C	-0.56484300	4.37222000	1.19583500
H	0.53055900	4.44604800	1.22042100

H	-0.96707700	5.00075800	2.00564800
H	-0.91944700	4.79264200	0.24808900
C	-0.26823400	2.45034700	-1.59133000
C	0.04457800	1.26953700	-2.52785100
H	1.02599000	0.83105500	-2.28110400
H	0.10341600	1.61185600	-3.57266900
H	-0.70619600	0.47579400	-2.46546300
C	0.86314400	3.48964200	-1.78773400
H	0.72432000	4.37589500	-1.16001300
H	0.88156500	3.82155600	-2.83828500
H	1.83985100	3.05077800	-1.54690000
C	-1.61709000	3.08167600	-1.99223700
H	-2.45738500	2.40159700	-1.82316600
H	-1.59695900	3.34646300	-3.06201100
H	-1.81132100	4.00320100	-1.43155600
C	-2.61203000	-0.33675800	0.02085600
C	-3.45311600	-0.90757700	1.00894400
H	-3.03767500	-1.10282300	1.99779000
C	-4.79824900	-1.21890000	0.76887100
H	-5.40504800	-1.65429400	1.56240100
C	-5.36288900	-0.96393600	-0.48916800
H	-6.40780600	-1.19986000	-0.68468400
C	-4.56378600	-0.39227400	-1.48925800
H	-4.98652600	-0.17919600	-2.47075400
C	-3.21982100	-0.08835100	-1.23080300
H	-2.63323000	0.35715200	-2.03115100
B	-1.10007700	0.02188000	0.43742600
H	-1.02666200	-0.03444600	1.64606100
P	0.30687100	-1.43830500	0.08308500
C	-0.28647700	-2.93285200	-0.96974100
C	-0.85590400	-2.35168800	-2.28420300
H	-0.10217100	-1.79016800	-2.84711400
H	-1.18772600	-3.19067300	-2.91342400
H	-1.72001500	-1.70940800	-2.09753800
C	0.86020500	-3.90118700	-1.34130800
H	1.24750400	-4.44870000	-0.47856100
H	0.46594300	-4.64049200	-2.05430800
H	1.69385500	-3.38286900	-1.83042000
C	-1.40617800	-3.71937800	-0.25663600
H	-2.20652100	-3.06223600	0.10048600
H	-1.84371900	-4.42960200	-0.97340900
H	-1.01608200	-4.30286800	0.58513000
C	0.91730300	-2.01708400	1.80848600
C	1.61190300	-0.81190800	2.48105900
H	1.00093200	0.09554700	2.43789800
H	1.78403200	-1.06240500	3.53824400
H	2.58185600	-0.60412100	2.02171100
C	-0.26681100	-2.44835400	2.70071300
H	-0.80666300	-3.31194900	2.30312600
H	0.13478800	-2.72506400	3.68646700
H	-0.97642100	-1.62721600	2.84367500
C	1.93753500	-3.17103700	1.71833900
H	2.73784500	-2.95872100	0.99792700
H	2.40221500	-3.30158400	2.70659800
H	1.46069300	-4.12068600	1.45276100
C	3.18276200	-0.14751700	-0.59691200
C	3.39479000	0.91688900	0.31548700

H	2.52089000	1.38365300	0.76923300
C	4.67916800	1.40603500	0.56774500
H	4.81385200	2.23938800	1.25568600
C	5.79456500	0.83094800	-0.06389100
H	6.79449600	1.21085400	0.14035200
C	5.61468900	-0.21535900	-0.98028300
H	6.47249100	-0.65158700	-1.48946000
C	4.32270200	-0.67347700	-1.26442400
H	4.18763100	-1.46105900	-2.00580600
B	1.84226000	-0.83179200	-0.95888500
H	1.77819400	-1.24342100	-2.08056700

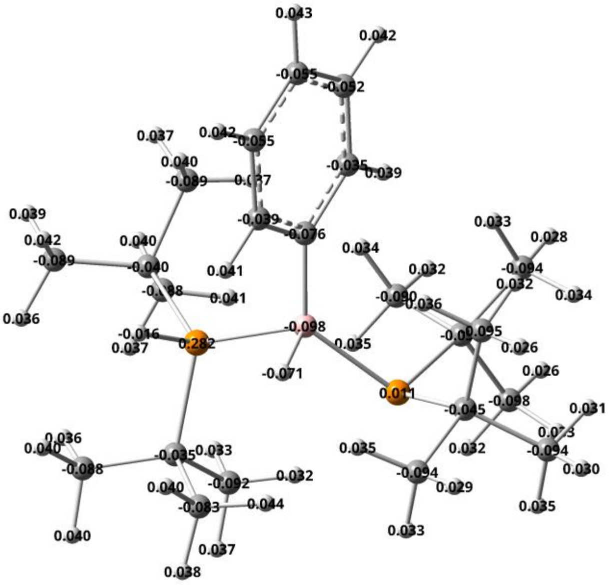

FIG. S58. OPTIMIZED STRUCTURE **1A_1_I1**

B	-0.16539600	0.27582400	-0.61940000
C	-0.24327000	1.87980700	-0.46641200
P	-1.43287900	-1.12655600	-0.01011500
C	-0.63864300	2.58301700	0.69168500
H	-0.91416800	2.02645700	1.58402300
P	1.77364400	-0.26592000	-0.47239900
C	-0.67804500	3.98277300	0.74190700
H	-0.99384800	4.48259100	1.65730300
C	-0.31010900	4.73944900	-0.38024500
H	-0.33924200	5.82745600	-0.34590700
C	0.08802700	4.07501500	-1.54945100
H	0.36889700	4.64637100	-2.43386900
C	0.11390400	2.67356500	-1.58378900
H	0.41031900	2.17272500	-2.50715800
C	-2.97151000	-0.75329700	-1.09865600
C	-4.16055700	-1.62308000	-0.63608400
H	-3.87818800	-2.68246800	-0.57534100
H	-4.98306800	-1.52939700	-1.36230700
H	-4.54890200	-1.31146600	0.34026500
C	-3.39538400	0.72827900	-1.15674500
H	-3.66581000	1.11872600	-0.17017200
H	-4.27301000	0.83615800	-1.81536600
H	-2.59068300	1.35474200	-1.55667200

C	-2.59759700	-1.21601600	-2.52781000
H	-1.76125600	-0.63613400	-2.93602400
H	-3.46318000	-1.07535500	-3.19392600
H	-2.32337000	-2.27922400	-2.54011700
C	-1.91295000	-0.95826100	1.84174300
C	-0.57946400	-0.73568500	2.58542300
H	0.14142400	-1.52462300	2.32955200
H	-0.74042300	-0.76788600	3.67422600
H	-0.13049400	0.23222100	2.33492000
C	-2.47013500	-2.32658900	2.30715700
H	-3.41834400	-2.57104800	1.81622600
H	-2.64890900	-2.30624800	3.39435100
H	-1.75911500	-3.13305300	2.08667700
C	-2.91619300	0.14298300	2.23802500
H	-2.60579300	1.13391200	1.89451900
H	-3.01213700	0.17504800	3.33582200
H	-3.91374600	-0.05488100	1.82947000
C	2.99603200	0.35811900	0.85477700
C	2.42579900	1.66738200	1.44354500
H	2.23236400	2.41891600	0.67020900
H	3.16304900	2.07810500	2.14902600
H	1.49182200	1.49636500	1.98608200
C	3.21244000	-0.65602600	1.99573100
H	2.26897700	-0.94563300	2.47036400
H	3.84405200	-0.18621800	2.76314100
H	3.72614400	-1.56097400	1.65225600
C	4.34987900	0.69493800	0.18558200
H	4.83455900	-0.17898300	-0.25939600
H	5.02632400	1.10100100	0.95176100
H	4.22713700	1.45909300	-0.59220000
C	2.11966300	-2.05632900	-0.98734400
C	1.64013300	-3.06070300	0.08425900
H	2.20005200	-2.97112600	1.02106000
H	1.79634700	-4.07787500	-0.30352500
H	0.56896300	-2.92852000	0.28194200
C	3.61437200	-2.28135100	-1.29628300
H	3.99244600	-1.56745300	-2.03961600
H	3.73347700	-3.29308900	-1.70999100
H	4.23804000	-2.21932500	-0.39730900
C	1.30006500	-2.28914400	-2.28088000
H	0.22546300	-2.24010800	-2.07806900
H	1.53745700	-3.29342600	-2.66102200
H	1.55025300	-1.55995400	-3.06284500
H	-0.16062800	0.02114000	-1.81044100
H	2.34351400	0.40840100	-1.57402000

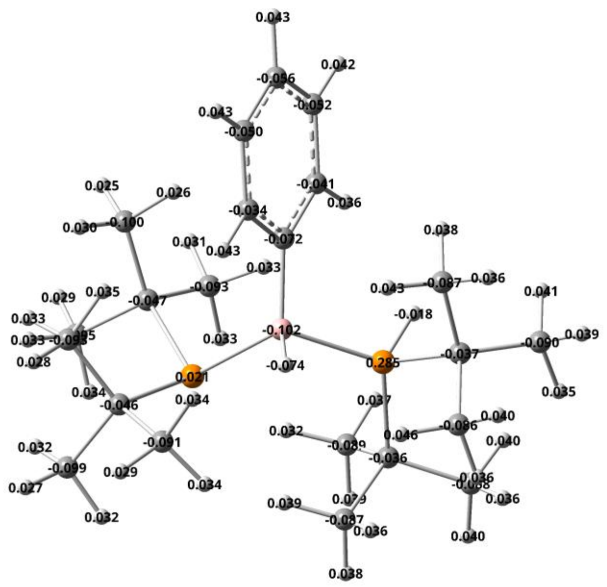

FIG. S59. OPTIMIZED STRUCTURE **1A_1_1A”**

B	0.04188600	0.23365100	0.38008100
C	-0.30677400	1.78015200	0.11061600
P	-1.31249700	-1.18562100	-0.04669000
C	-0.95596900	2.51469400	1.13120900
H	-1.15571300	2.02287400	2.08362700
P	1.85101600	-0.16501900	-0.37365900
C	-1.35299900	3.84580500	0.95611900
H	-1.85806600	4.37234800	1.76552200
C	-1.09603700	4.50473100	-0.25684000
H	-1.40218400	5.53988500	-0.39858400
C	-0.43374900	3.81285900	-1.28012300
H	-0.22101800	4.30953200	-2.22640200
C	-0.04875400	2.47725600	-1.09164500
H	0.45509000	1.96841700	-1.91446700
C	-2.47031900	-0.53635200	-1.44021200
C	-3.55994100	-1.59638800	-1.71818900
H	-3.12918000	-2.60189300	-1.81299800
H	-4.07828900	-1.35012600	-2.65843600
H	-4.31637400	-1.61529900	-0.92378700
C	-3.16029100	0.83456000	-1.26392900
H	-3.84763200	0.84539700	-0.41411500
H	-3.75084500	1.04800300	-2.17035100
H	-2.44173000	1.64594000	-1.13357400
C	-1.55681900	-0.46419900	-2.68743000
H	-0.69848700	0.19906100	-2.52268900
H	-2.12805100	-0.06371400	-3.53910100
H	-1.18213700	-1.45678000	-2.96612200
C	-2.34488400	-1.43372600	1.56673700
C	-1.38232600	-1.45237700	2.77512600
H	-0.50590100	-2.08853300	2.59097200
H	-1.91681100	-1.85748700	3.64830900
H	-1.02552500	-0.44847200	3.02920400
C	-2.98084300	-2.84337700	1.47403000
H	-3.66748700	-2.93340500	0.62585600
H	-3.55067500	-3.05314100	2.39360900
H	-2.20628300	-3.61276900	1.36296800

C	-3.44894100	-0.39535700	1.84270600
H	-3.05691200	0.62780800	1.82523500
H	-3.88601700	-0.58051300	2.83758100
H	-4.26236100	-0.46665400	1.11124400
C	3.18829600	0.72787400	0.63934900
C	2.63661200	2.09804200	1.09845300
H	2.34775600	2.73358400	0.25447800
H	3.43289600	2.60831300	1.66012300
H	1.76573600	1.98802600	1.75090500
C	3.52915700	-0.09635500	1.90037200
H	2.62704100	-0.31802100	2.48421100
H	4.20455400	0.49572900	2.53363500
H	4.03571500	-1.03836600	1.66302200
C	4.45141300	0.98777300	-0.21194900
H	4.94584100	0.06955100	-0.53931000
H	5.17029600	1.55819300	0.39368300
H	4.21085000	1.58914100	-1.09807300
C	2.29526100	-1.97601200	-0.69500600
C	1.88043300	-2.84745900	0.51130200
H	2.42577800	-2.58477100	1.42415500
H	2.10128400	-3.89807200	0.27414400
H	0.80306000	-2.75756700	0.69301800
C	3.78742100	-2.18580400	-1.02313700
H	4.10902500	-1.57084300	-1.87336400
H	3.92840200	-3.24015600	-1.30112800
H	4.44195800	-1.98087400	-0.16906300
C	1.46190200	-2.40026800	-1.92799000
H	0.39009600	-2.33840500	-1.70734200
H	1.70503900	-3.44583800	-2.16679100
H	1.69469600	-1.78705100	-2.80883900
H	0.32440900	0.04684200	1.54469900
H	2.08981300	0.37504100	-1.65824800

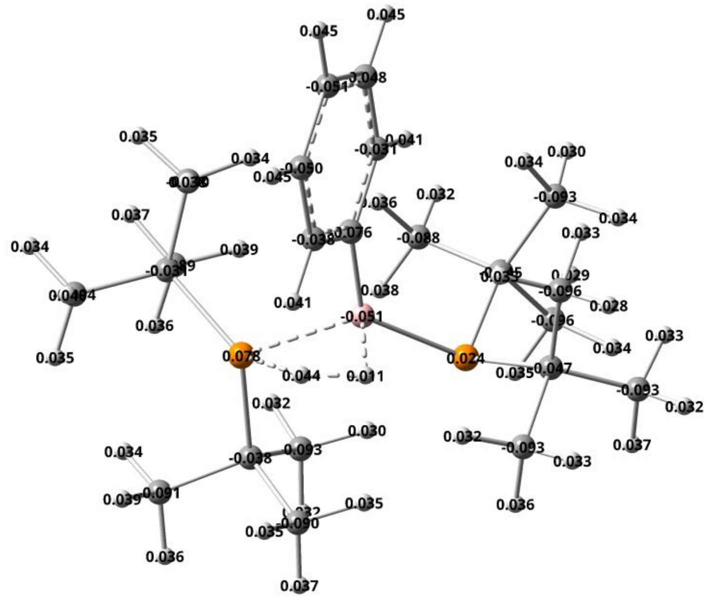

FIG. S60. OPTIMIZED STRUCTURE **1A_1_TS1_P=B**

B	-0.14326200	0.26088100	-0.54597900
C	-0.30847900	1.85214600	-0.39689400

P	-1.42213200	-1.18853100	-0.08087300
C	-0.73430600	2.48890300	0.78709400
H	-0.95438900	1.89013200	1.66629300
P	1.90141700	-0.21364800	-0.63077200
C	-0.87087200	3.87982700	0.87751600
H	-1.20639600	4.32844000	1.81176700
C	-0.57459500	4.69192700	-0.22632200
H	-0.67920700	5.77349600	-0.15988400
C	-0.14813100	4.09113600	-1.41896700
H	0.08171900	4.70430100	-2.28938500
C	-0.02602100	2.69744300	-1.49674100
H	0.29854900	2.25544900	-2.44090100
C	-3.00208900	-0.73775300	-1.07873300
C	-4.13805300	-1.70041500	-0.66662400
H	-3.81090500	-2.74710500	-0.72252600
H	-4.99195700	-1.57096900	-1.34943400
H	-4.49801800	-1.50525200	0.34997900
C	-3.48574200	0.72315600	-0.99121300
H	-3.77021400	1.00533800	0.02699600
H	-4.36970200	0.85508100	-1.63620000
H	-2.71175200	1.42062800	-1.32938600
C	-2.65060900	-1.05094200	-2.55403000
H	-1.87546600	-0.37698900	-2.94393900
H	-3.54604400	-0.91394200	-3.17940100
H	-2.30103000	-2.08468900	-2.67179700
C	-1.79872000	-1.10067300	1.80540700
C	-0.45796100	-0.81836700	2.51731500
H	0.30171800	-1.55509600	2.23309000
H	-0.59884200	-0.88018300	3.60761000
H	-0.06459500	0.17671700	2.27987400
C	-2.25919000	-2.51933000	2.22487600
H	-3.20161700	-2.80104300	1.74131800
H	-2.41504500	-2.55142000	3.31493100
H	-1.50566700	-3.27143800	1.95978500
C	-2.85639000	-0.08429300	2.27860900
H	-2.62173700	0.93952900	1.97428700
H	-2.91177800	-0.10927200	3.37886900
H	-3.85376100	-0.32881700	1.89623400
C	3.06050300	0.57879300	0.67438900
C	2.47152300	1.91240600	1.16780800
H	2.22409500	2.58889500	0.34266100
H	3.22429800	2.40308200	1.80311100
H	1.56875100	1.75850400	1.76544900
C	3.29619000	-0.34132200	1.88883800
H	2.35875700	-0.57966400	2.40155900
H	3.95173300	0.18246000	2.60139000
H	3.79370200	-1.27693800	1.61072900
C	4.40473100	0.87488200	-0.03284000
H	4.88157400	-0.03226600	-0.41805400
H	5.09326800	1.33966000	0.68963600
H	4.25825100	1.56765600	-0.86950400
C	2.16375200	-2.08517500	-0.86841300
C	1.69394100	-2.98379400	0.29271500
H	2.21947900	-2.76412500	1.22794400
H	1.89366000	-4.03536800	0.03389700
H	0.61423900	-2.87313800	0.44598200
C	3.65710600	-2.34769300	-1.16617500

H	4.01872100	-1.71904600	-1.98964500
H	3.77863700	-3.40181000	-1.45730700
H	4.29171200	-2.17482300	-0.28868700
C	1.34697200	-2.44707100	-2.13350400
H	0.27067900	-2.33513100	-1.95338300
H	1.53477700	-3.50141800	-2.38561800
H	1.64436500	-1.83134100	-2.99279500
H	-0.31141000	0.13041400	-1.84591300
H	0.72252500	0.18704300	-1.73972700

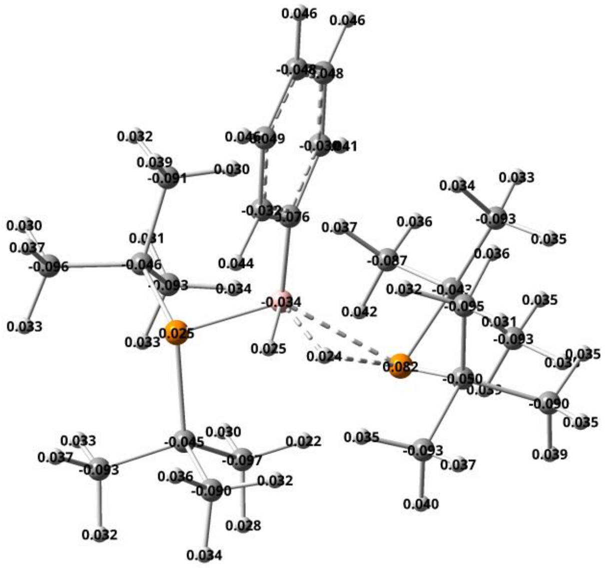

FIG. S61. OPTIMIZED STRUCTURE **1A_1_TS1_P-B**

B	0.11514100	0.35530700	-0.59133200
C	-0.49686500	1.83324900	-0.51642700
P	-1.24848900	-1.43703700	0.13343600
C	-0.86436300	2.52444900	0.65672200
H	-0.74378900	2.04776100	1.62506000
P	2.08334400	0.12055200	-0.71895900
C	-1.37258700	3.83020700	0.62607300
H	-1.64625100	4.32240600	1.55842700
C	-1.52491900	4.50060800	-0.59474700
H	-1.91987100	5.51481000	-0.62162800
C	-1.14945700	3.85212600	-1.77939700
H	-1.24597200	4.36088100	-2.73751100
C	-0.64288000	2.54709800	-1.73197200
H	-0.34150400	2.06839000	-2.66488400
C	-2.82557700	-1.33301600	-0.98047500
C	-3.91808400	-2.25723600	-0.40108900
H	-3.52804600	-3.26172700	-0.19139900
H	-4.73161900	-2.35466500	-1.13634900
H	-4.35351600	-1.85110100	0.51879600
C	-3.40331400	0.07569400	-1.22302500
H	-3.74157300	0.55065100	-0.29789000
H	-4.26858700	-0.00133300	-1.90043700
H	-2.67055800	0.74325100	-1.68963500
C	-2.38067900	-1.93018600	-2.34025800
H	-1.62553200	-1.31002200	-2.84454800
H	-3.24903800	-1.98669200	-3.01353000
H	-1.96872300	-2.93990600	-2.21967300

C	-1.72047900	-0.96367300	1.93292700
C	-0.40161900	-0.57135300	2.62740800
H	0.33989000	-1.37361800	2.52647700
H	-0.58233300	-0.40583700	3.70004900
H	0.03597700	0.33749000	2.20463500
C	-2.19027200	-2.29394800	2.58086800
H	-3.12910400	-2.65831800	2.15155100
H	-2.35637600	-2.12289400	3.65567200
H	-1.43256800	-3.07949600	2.47002800
C	-2.80501100	0.10619000	2.14401800
H	-2.58349500	1.03661100	1.61586400
H	-2.88324900	0.33085700	3.21942900
H	-3.78677300	-0.25180400	1.81280200
C	3.05224100	0.78948500	0.80438400
C	2.33554800	2.05592800	1.31224700
H	2.06472400	2.73158500	0.49183900
H	3.00639500	2.59798100	1.99638900
H	1.42424900	1.80929000	1.86537900
C	3.29003700	-0.16545300	1.98812900
H	2.35463400	-0.57084900	2.38526000
H	3.79302800	0.38585000	2.79870900
H	3.94244400	-1.00130000	1.70963200
C	4.42605200	1.23860600	0.24183600
H	5.00186000	0.40143500	-0.16763100
H	5.01822500	1.69101500	1.05279500
H	4.29801300	1.98275800	-0.55335500
C	2.46212900	-1.74215400	-0.97489900
C	2.11427000	-2.71935300	0.16702400
H	2.72199200	-2.54494700	1.05927000
H	2.31141500	-3.74928500	-0.17296000
H	1.05481000	-2.65354500	0.43574800
C	3.96514600	-1.87437400	-1.31173600
H	4.26096900	-1.18775300	-2.11514300
H	4.17347700	-2.90437200	-1.63970900
H	4.59456900	-1.67609000	-0.43554700
C	1.65003500	-2.15063100	-2.22806800
H	0.58008700	-2.23249800	-1.99994400
H	1.98391300	-3.14185800	-2.57037800
H	1.79033400	-1.43799900	-3.05253200
H	-0.64587500	-0.45444800	-1.23498200
H	-0.01128300	0.09651400	-1.85190000

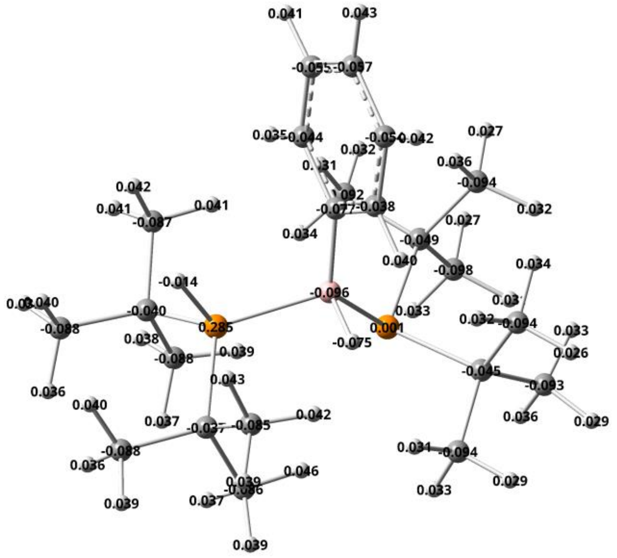

FIG. S62. OPTIMIZED STRUCTURE **1A_1_TS2**

B	-0.08574800	0.27339900	-0.44630100
C	-0.54874000	1.82122100	-0.51053700
P	-1.09669000	-1.28777000	0.32542500
C	-0.57769800	2.73835500	0.56574100
H	-0.26186700	2.41982900	1.55586600
P	1.90732400	0.23366500	-0.05051900
C	-0.99146700	4.06957600	0.41332400
H	-1.00004200	4.73313500	1.27772100
C	-1.39167700	4.54553300	-0.84246600
H	-1.71854000	5.57694400	-0.96525200
C	-1.35094200	3.67396600	-1.93987600
H	-1.64054400	4.02661600	-2.92948000
C	-0.92901600	2.34843800	-1.76980600
H	-0.88852600	1.69892400	-2.64362600
C	-2.12607100	-1.84141600	-1.22010600
C	-3.23629000	-2.83177400	-0.80859700
H	-2.84780900	-3.63181900	-0.16524100
H	-3.65487100	-3.29612100	-1.71521600
H	-4.06151500	-2.33160800	-0.28940000
C	-2.75142900	-0.70757100	-2.05676000
H	-3.42876700	-0.07761500	-1.47307000
H	-3.32419300	-1.14208900	-2.89259800
H	-1.97611800	-0.06386700	-2.48257600
C	-1.14733900	-2.61866600	-2.13003600
H	-0.34125000	-1.97322600	-2.49988800
H	-1.69189900	-3.00279300	-3.00694300
H	-0.70083500	-3.47085900	-1.60105900
C	-2.35791500	-0.71544200	1.66507400
C	-1.54692300	0.01599400	2.75183300
H	-0.70489400	-0.59404600	3.10486600
H	-2.19581800	0.22481200	3.61646800
H	-1.17184000	0.97417800	2.38328300
C	-2.97246300	-1.95758800	2.35638500
H	-3.63489900	-2.52794000	1.70063800
H	-3.56652800	-1.62900000	3.22413000
H	-2.18440800	-2.63256500	2.71496700
C	-3.47432400	0.22814300	1.17677500

H	-3.06082500	1.10096600	0.65799100
H	-4.05888500	0.58662200	2.04004500
H	-4.17094100	-0.28233000	0.50181500
C	2.58642600	-0.02251900	1.70023300
C	1.88548900	1.05541700	2.55780100
H	2.13329400	2.06851100	2.21555100
H	2.22771400	0.95301700	3.59742300
H	0.80140800	0.93506700	2.54908600
C	2.21169300	-1.43028700	2.21149700
H	1.15387200	-1.66121500	2.03350100
H	2.41209200	-1.48427100	3.29126700
H	2.81324700	-2.20452400	1.72085600
C	4.10779000	0.20975500	1.82622600
H	4.69274800	-0.61747700	1.41669000
H	4.35399000	0.28823100	2.89503500
H	4.42556200	1.14153200	1.34093300
C	2.84140000	-0.75792200	-1.37193000
C	2.45472900	-2.24801100	-1.26258100
H	2.92033500	-2.71680000	-0.38803600
H	2.80613100	-2.78017800	-2.15787800
H	1.37157500	-2.38259400	-1.18252700
C	4.37493500	-0.60121100	-1.28261300
H	4.68174200	0.45087400	-1.22277100
H	4.81864600	-1.02819500	-2.19326900
H	4.79751000	-1.14083700	-0.43104800
C	2.40251700	-0.18993200	-2.74636500
H	1.33756000	-0.34281500	-2.93873000
H	2.97458500	-0.70943600	-3.52793700
H	2.61640700	0.88425100	-2.82570000
H	0.02852000	-0.12555200	-1.58136000
H	2.41198100	1.52650300	-0.31590800

Formation of 1b

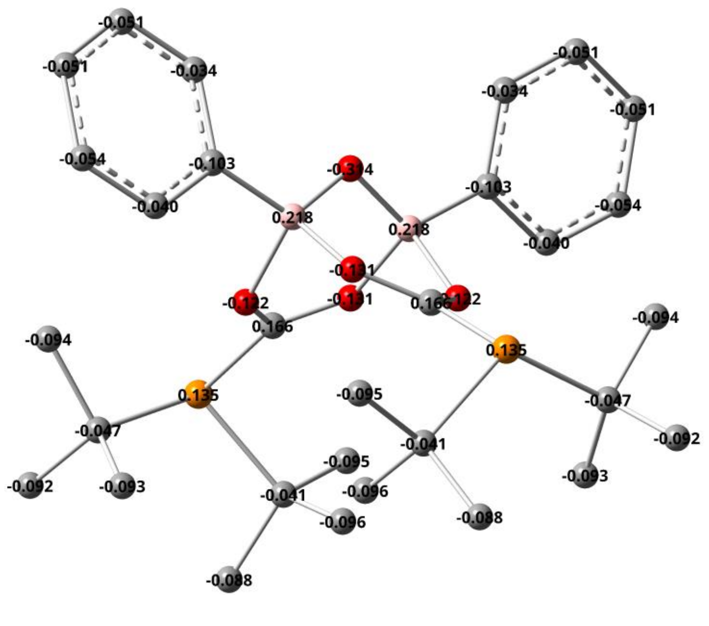

FIG. S63. OPTIMIZED STRUCTURE **1B**

P 2.55140300 -2.24146900 -0.71990300

P	-2.55140700	2.24148600	-0.71985800
O	0.24726900	-1.74601800	0.56514800
O	1.53767300	0.09781200	0.32474000
O	0.00000300	-0.00001900	2.24550000
O	-0.24727300	1.74601400	0.56518400
O	-1.53767800	-0.09781100	0.32474700
C	-1.77181700	-1.87470500	2.16053500
C	-2.48122800	-2.85593400	1.43687000
H	-2.29437700	-2.97510300	0.36874300
C	-3.42535000	-3.68108800	2.06208200
H	-3.96177200	-4.43324800	1.48459400
C	-3.67894800	-3.53681200	3.43500600
H	-4.41193000	-4.17606300	3.92528300
C	-2.98416300	-2.56684900	4.17219500
H	-3.17710900	-2.45206300	5.23817100
C	-2.04152900	-1.74520600	3.53695100
H	-1.50190600	-0.99070800	4.10802300
C	1.77182400	1.87466800	2.16056100
C	2.04155500	1.74512900	3.53697000
H	1.50194000	0.99061500	4.10802700
C	2.98419600	2.56675500	4.17222400
H	3.17715800	2.45193800	5.23819500
C	3.67897000	3.53674000	3.43505400
H	4.41195900	4.17597800	3.92533900
C	3.42535400	3.68105600	2.06213800
H	3.96176600	4.43323300	1.48466500
C	2.48122400	2.85591800	1.43691500
H	2.29435800	2.97511800	0.36879400
C	1.31323900	-1.15335700	0.12392800
C	3.86506800	-1.00863300	-1.34818000
C	3.37284200	0.09257900	-2.30429400
H	3.00835100	-0.32582800	-3.25055600
H	4.21116300	0.76507900	-2.54112900
H	2.57775500	0.69090800	-1.84874000
C	4.49301000	-0.37717700	-0.07717500
H	3.80702000	0.30042000	0.43595700
H	5.38054200	0.19708800	-0.38114500
H	4.82033700	-1.15184200	0.62903000
C	4.97648500	-1.84624500	-2.02971300
H	5.31799200	-2.66186200	-1.37897700
H	5.83573500	-1.18909600	-2.22652100
H	4.66021000	-2.27442900	-2.98558700
C	1.40974300	-2.88556100	-2.13450900
C	0.41049700	-1.84428800	-2.67617000
H	-0.28661400	-1.51403700	-1.89889500
H	-0.18297600	-2.30138500	-3.48260000
H	0.91745800	-0.96530900	-3.08707000
C	0.63853000	-4.08039600	-1.52408600
H	1.32754800	-4.86683700	-1.19204100
H	-0.02529700	-4.50315500	-2.29336600
H	0.02718800	-3.77307700	-0.66983200
C	2.28616500	-3.40596200	-3.29258500
H	2.76030000	-2.58789500	-3.84699500
H	1.64202900	-3.95416600	-3.99549800
H	3.06478600	-4.09562500	-2.94271900
C	-1.31324400	1.15336000	0.12395500
C	-1.40974800	2.88559500	-2.13445800

C	-0.63853200	4.08042000	-1.52402000
H	-1.32754700	4.86685800	-1.19196300
H	0.02529500	4.50318800	-2.29329500
H	-0.02718800	3.77308800	-0.66977200
C	-2.28617000	3.40601400	-3.29252500
H	-2.76030900	2.58795500	-3.84694600
H	-1.64203500	3.95422500	-3.99543200
H	-3.06478900	4.09567400	-2.94264800
C	-0.41050500	1.84432700	-2.67613400
H	0.28660700	1.51406500	-1.89886500
H	0.18296700	2.30143300	-3.48256000
H	-0.91746800	0.96535400	-3.08704500
C	-3.86507700	1.00866200	-1.34815100
C	-4.97649300	1.84628500	-2.02966900
H	-5.31799700	2.66189500	-1.37892200
H	-5.83574500	1.18914100	-2.22648300
H	-4.66022000	2.27448200	-2.98553900
C	-4.49301600	0.37718900	-0.07715200
H	-3.80702700	-0.30041600	0.43596900
H	-5.38055100	-0.19707000	-0.38112800
H	-4.82034100	1.15184500	0.62906300
C	-3.37285500	-0.09253700	-2.30428100
H	-3.00836600	0.32588100	-3.25053800
H	-4.21117900	-0.76503300	-2.54112300
H	-2.57776900	-0.69087600	-1.84873700
B	-0.73063600	-0.91143600	1.45629000
B	0.73063700	0.91141400	1.45630400

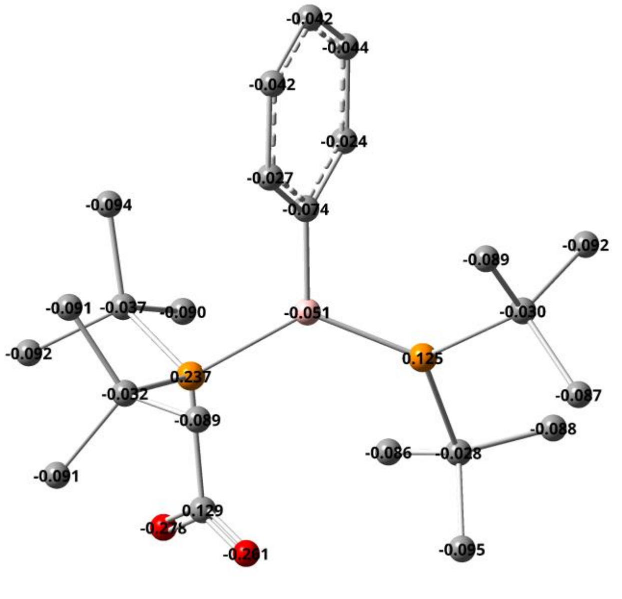

FIG. S64. OPTIMIZED STRUCTURE **1B_I1**

B	0.19588600	0.34417500	0.00891400
C	0.43297500	1.90361400	-0.12812400
P	-1.63436200	-0.37420600	0.04325100
C	0.08641600	2.89652800	0.81671100
H	-0.34672400	2.60695500	1.77014800
P	1.72033000	-0.65015300	-0.01809400
C	0.34183600	4.25334200	0.58692300
H	0.07712100	4.98684500	1.34709100

C	0.94514000	4.66877500	-0.60990400
H	1.14063700	5.72442700	-0.78926700
C	1.29984100	3.70883700	-1.56720500
H	1.77454500	4.01312000	-2.49885500
C	1.05302200	2.35106000	-1.32346800
H	1.33572700	1.61181500	-2.07186300
C	-2.55106700	0.17448600	-1.52624600
C	-4.01732300	-0.30864600	-1.47625600
H	-4.07532000	-1.37972400	-1.25640100
H	-4.47360100	-0.12685300	-2.46045500
H	-4.60082200	0.25145400	-0.73528800
C	-2.51144900	1.69460000	-1.79163600
H	-3.03078800	2.26992200	-1.02025700
H	-3.02219900	1.88120900	-2.74840500
H	-1.48878700	2.07298400	-1.87552200
C	-1.81039900	-0.54887900	-2.67680700
H	-0.75643200	-0.24190100	-2.72327400
H	-2.28650900	-0.27337300	-3.62901500
H	-1.86560100	-1.63598000	-2.55800000
C	-2.48106300	0.22145900	1.65256700
C	-1.41718400	0.13969400	2.77255400
H	-1.02147700	-0.87897800	2.84589400
H	-1.89116600	0.41113500	3.72744700
H	-0.57623500	0.82238200	2.60137400
C	-3.63676200	-0.74325800	2.01617400
H	-4.38843700	-0.80865400	1.22270400
H	-4.13037300	-0.35134100	2.91772100
H	-3.26673800	-1.74858400	2.23692900
C	-3.06368000	1.64604700	1.53891900
H	-2.34424000	2.37821200	1.16377200
H	-3.38820000	1.96549000	2.54046500
H	-3.94489300	1.66152100	0.88706800
C	3.32865800	0.14356700	0.64300900
C	3.01268600	1.41028400	1.46990200
H	2.71212400	2.25126700	0.84174700
H	3.93210800	1.69600800	2.00207500
H	2.22800800	1.22712200	2.21323600
C	3.99008300	-0.86437300	1.61666300
H	3.32043000	-1.09955100	2.45266400
H	4.89983300	-0.39998400	2.02442000
H	4.28496900	-1.80124400	1.13660400
C	4.30263500	0.53550200	-0.49104400
H	4.62533400	-0.32495300	-1.08423900
H	5.19850500	0.99654400	-0.04851600
H	3.83686800	1.26787000	-1.16053000
C	1.93493800	-2.46090400	-0.56778300
C	1.83535100	-3.45630800	0.60861800
H	2.62942100	-3.29149200	1.34551600
H	1.94825400	-4.47678700	0.21248300
H	0.86275100	-3.37990800	1.10361000
C	3.29161700	-2.62003800	-1.29707500
H	3.37567600	-1.93179800	-2.14724100
H	3.33463300	-3.64675800	-1.68798100
H	4.15861400	-2.48353400	-0.64401700
C	0.84705300	-2.75761000	-1.62240600
H	-0.16642000	-2.68065500	-1.23158900
H	0.98024900	-3.79315400	-1.96755000

H	0.94462700	-2.08972000	-2.48660500
C	-1.79273800	-2.38202800	0.29740700
O	-2.47118300	-2.95003200	-0.56799800
O	-1.19481000	-2.70513700	1.33812500

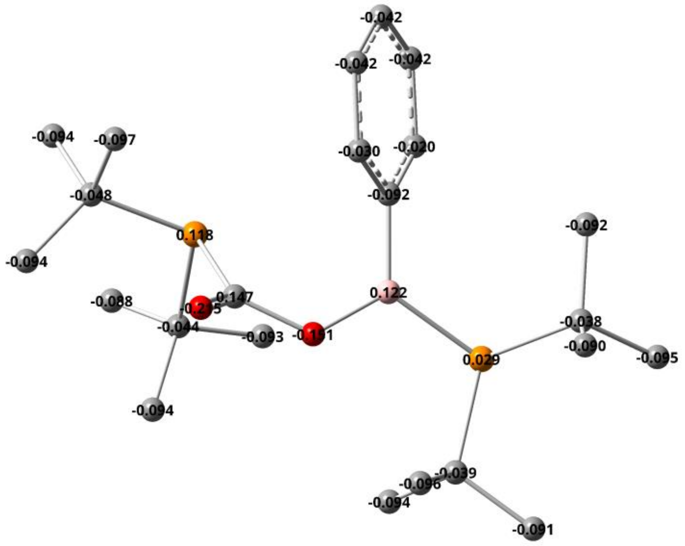

FIG. S65. OPTIMIZED STRUCTURE **1B_I2A**

B	0.93277800	0.25572500	0.14589100
C	0.40199200	1.71029500	-0.09374000
P	-2.40383200	-0.08247300	-0.33347600
C	-0.10705600	2.47590300	0.98060800
H	-0.09183000	2.06642700	1.99037100
P	2.59898900	-0.40995900	-0.51585300
C	-0.63153800	3.75764800	0.77043600
H	-1.01166300	4.33289500	1.61336400
C	-0.67621700	4.29377400	-0.52480700
H	-1.09367500	5.28543300	-0.69172900
C	-0.18200800	3.54640000	-1.60492600
H	-0.22166000	3.95514000	-2.61354000
C	0.35892500	2.27451100	-1.38791500
H	0.73373200	1.69662300	-2.23188300
C	-2.32393800	-1.80570800	-1.19371200
C	-2.14950900	-3.00341400	-0.23921300
H	-1.21401800	-2.93794600	0.32751700
H	-2.11900300	-3.93371600	-0.82699300
H	-2.97611300	-3.07974600	0.47473100
C	-3.60138700	-2.00300800	-2.03724300
H	-4.48461000	-2.17553700	-1.41219900
H	-3.46522300	-2.89008700	-2.67353600
H	-3.79435200	-1.14488800	-2.69383700
C	-1.12257800	-1.71722700	-2.16431100
H	-1.26987600	-0.91455500	-2.89755200
H	-1.02517400	-2.67022300	-2.70592600
H	-0.17796700	-1.53301300	-1.64337600
C	-4.06237600	0.16242000	0.57710300
C	-3.83680800	1.38491400	1.50480900
H	-3.17712800	1.13998400	2.34172900

H	-4.81009700	1.70048800	1.90931500
H	-3.40812000	2.23055500	0.95077200
C	-4.59261200	-1.02863100	1.39549400
H	-4.85474700	-1.87808100	0.75275700
H	-5.50797000	-0.72267000	1.92518100
H	-3.85957100	-1.35501000	2.14039200
C	-5.10342100	0.58794400	-0.48816800
H	-4.73291900	1.42552300	-1.09389000
H	-6.01793100	0.91842900	0.02599800
H	-5.37854100	-0.23001700	-1.16148100
C	3.99524300	0.85006000	-0.10963000
C	3.53230900	2.26948500	-0.50146200
H	3.15466300	2.30797000	-1.53023700
H	4.40001500	2.94302400	-0.43412300
H	2.75246200	2.65051500	0.16408900
C	4.40635700	0.86488700	1.37602000
H	3.55010900	1.09059200	2.02334700
H	5.16964900	1.64325500	1.53194000
H	4.84059800	-0.08929500	1.69433900
C	5.21731300	0.50968900	-0.99707200
H	5.63879600	-0.47553500	-0.77764600
H	6.00528200	1.25805800	-0.82255100
H	4.94592500	0.53760400	-2.05954500
C	2.91112300	-2.08472800	0.37098400
C	2.64962500	-2.07740900	1.89322200
H	3.32929800	-1.40143400	2.42181200
H	2.81308700	-3.09257100	2.28853300
H	1.61998000	-1.78538800	2.11970000
C	4.34827600	-2.57826400	0.10036800
H	4.58332200	-2.57679900	-0.97172700
H	4.43949000	-3.61155800	0.46763000
H	5.09915100	-1.97707300	0.62605700
C	1.93912800	-3.08363800	-0.30061200
H	0.89672900	-2.80569900	-0.12087700
H	2.10036400	-4.08488600	0.12732200
H	2.10647200	-3.13824400	-1.38377900
C	-1.25909700	-0.45333200	1.10831900
O	-1.54647400	-0.53158700	2.28757100
O	0.07789200	-0.67917700	0.74151600

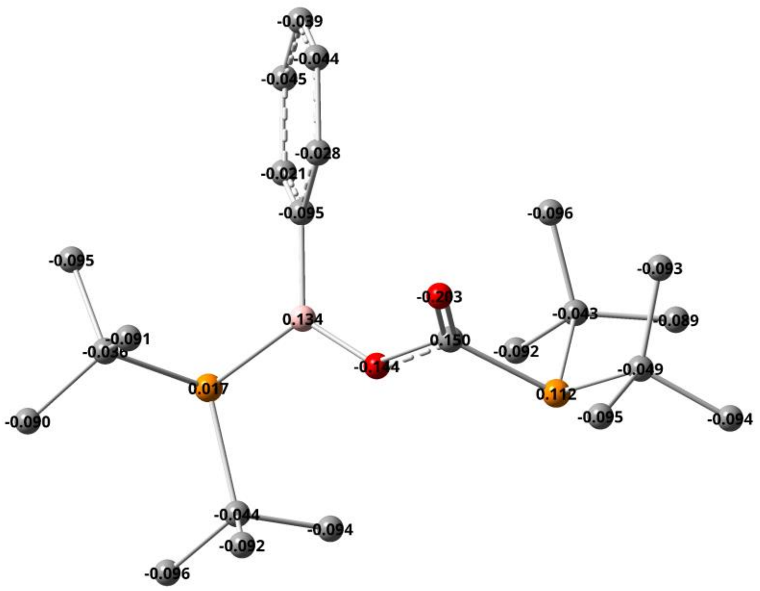

FIG. S66. OPTIMIZED STRUCTURE **1B_I2B**

B	1.06877300	0.24853200	-0.00417900
C	1.03256900	1.81471100	-0.00561000
P	-2.65608200	-1.02240600	0.12703900
C	1.48913100	2.49112800	1.15346700
H	1.87203000	1.90666700	1.98923900
P	2.63575900	-0.65654500	0.66103500
C	1.45024900	3.88792600	1.24468700
H	1.79270100	4.38261300	2.15223200
C	0.98295700	4.64664400	0.16193500
H	0.96109900	5.73352000	0.22480600
C	0.54760200	3.99983900	-1.00479000
H	0.19062300	4.58501700	-1.85090900
C	0.56022500	2.60202900	-1.08414400
H	0.20211100	2.11177000	-1.98568100
C	-2.71993500	0.25447600	1.57112100
C	-2.47357400	1.71972100	1.16071700
H	-1.47101900	1.86341900	0.74392100
H	-2.55370800	2.35930300	2.05311700
H	-3.20293800	2.07217100	0.42537400
C	-4.09363000	0.13164700	2.26502600
H	-4.90167000	0.53879500	1.64687600
H	-4.06638900	0.71108600	3.19962100
H	-4.33343800	-0.90885400	2.51999600
C	-1.63120900	-0.18543000	2.57812000
H	-1.78716200	-1.21904400	2.91060300
H	-1.67504700	0.47411500	3.45763800
H	-0.62438800	-0.11094100	2.15523300
C	-4.14209600	-0.80261200	-1.04845100
C	-3.75360400	-1.57083700	-2.33852500
H	-2.96682400	-1.05428800	-2.89575400
H	-4.64227800	-1.64625400	-2.98245900
H	-3.41765700	-2.59148000	-2.10916200
C	-4.54847900	0.63897700	-1.40507700
H	-4.93826800	1.17797700	-0.53292200
H	-5.35055300	0.60988600	-2.15852500
H	-3.70495200	1.19715900	-1.82461300
C	-5.33832300	-1.55276400	-0.41181900

H	-5.06729700	-2.58338500	-0.14757700
H	-6.15914000	-1.59375800	-1.14286600
H	-5.71797800	-1.05559400	0.48662200
C	2.26308500	-2.53443300	0.59146100
C	1.04579100	-2.75117000	1.52568200
H	1.21681100	-2.29703600	2.51143400
H	0.90314900	-3.83234200	1.67372700
H	0.12404600	-2.34232900	1.10406500
C	1.94798900	-3.09851600	-0.80716500
H	1.11280000	-2.56436700	-1.27370400
H	1.66942400	-4.16060800	-0.72106900
H	2.81782700	-3.04017900	-1.47300600
C	3.44996900	-3.30412500	1.21945200
H	4.35336100	-3.26558400	0.60374600
H	3.16863300	-4.36219700	1.33306300
H	3.69383900	-2.90698000	2.21324500
C	3.90941100	-0.14062400	-0.70235700
C	3.30011500	-0.06899000	-2.11979100
H	2.86152900	-1.02440700	-2.42677800
H	4.08477700	0.19453400	-2.84707000
H	2.52204700	0.70259300	-2.17853700
C	5.10362200	-1.11610300	-0.71058300
H	5.54094300	-1.23218800	0.28888600
H	5.88091600	-0.71369500	-1.37794000
H	4.82415000	-2.10537200	-1.09043200
C	4.43772500	1.25704900	-0.30666900
H	3.64445900	2.01155800	-0.30869700
H	5.20191700	1.56797900	-1.03579100
H	4.89614300	1.23981900	0.68995800
C	-1.28119100	-0.22492000	-0.86748400
O	-1.34126800	0.34063400	-1.94582700
O	-0.06173900	-0.52178700	-0.27609100

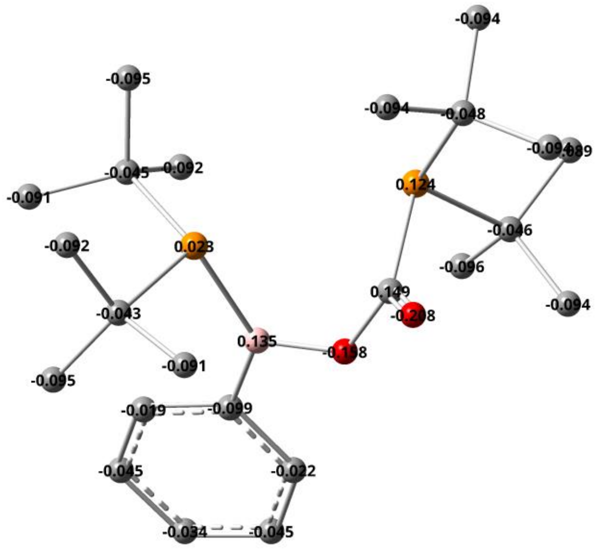

FIG. S67. OPTIMIZED STRUCTURE **1B_I2C**

B	-1.05635900	0.52615900	-0.44272400
C	-2.13244000	1.63955600	-0.24147800

P	2.16905500	0.14230800	0.39032400
C	-3.36560700	1.40134900	0.40394500
H	-3.58170400	0.40217100	0.77187300
P	-0.94865700	-1.38923800	0.00545200
C	-4.31430200	2.41590800	0.57461000
H	-5.25606800	2.20370600	1.07802300
C	-4.04874700	3.70562800	0.09143600
H	-4.78373500	4.49869900	0.22015600
C	-2.83519600	3.97069500	-0.56356100
H	-2.62948200	4.96955800	-0.94520600
C	-1.89284800	2.95025400	-0.72565600
H	-0.95649900	3.15734000	-1.24094200
C	3.59258300	-1.04849800	-0.04454500
C	4.58171300	-0.58607100	-1.13025800
H	4.06115200	-0.35355400	-2.06487200
H	5.29962300	-1.39703900	-1.32819800
H	5.15804100	0.29101300	-0.81166200
C	4.34610900	-1.34520000	1.27536600
H	4.89725500	-0.47624500	1.65029600
H	5.07363700	-2.15022900	1.09498700
H	3.65574800	-1.68111200	2.06042900
C	2.90490900	-2.36396800	-0.49341300
H	2.14580900	-2.68810100	0.22943000
H	3.67155100	-3.14984900	-0.56924400
H	2.42282300	-2.25310900	-1.46899200
C	2.78589400	1.97225100	0.43500900
C	1.63539300	2.76806500	1.09621900
H	0.71991200	2.73578300	0.49644100
H	1.93766700	3.82163700	1.19651200
H	1.40517900	2.37884300	2.09603500
C	3.11322900	2.59360500	-0.93724100
H	3.88335200	2.03091800	-1.47391500
H	3.48183200	3.62042100	-0.78864900
H	2.22301900	2.64821500	-1.57527400
C	4.01967900	2.05456500	1.35858200
H	3.83211000	1.57925000	2.33035300
H	4.25244200	3.11418500	1.54194600
H	4.90504000	1.59342900	0.90683600
C	-1.52566200	-1.68070400	1.81486100
C	-1.09846600	-0.43731100	2.62809900
H	-0.04626500	-0.18742100	2.43900400
H	-1.21402700	-0.64625000	3.70302900
H	-1.71178900	0.43854000	2.38486900
C	-3.01064900	-1.99043800	2.07963200
H	-3.67073800	-1.16150100	1.80243800
H	-3.14826800	-2.17263700	3.15695000
H	-3.34590700	-2.88888700	1.54862300
C	-0.68261700	-2.88858600	2.29795700
H	-0.91380100	-3.79698600	1.72702600
H	-0.89362000	-3.08946200	3.35992700
H	0.38909700	-2.68182300	2.18896400
C	-2.13000800	-2.19110400	-1.27472900
C	-3.58382800	-1.68319900	-1.29520300
H	-4.10361900	-1.87013700	-0.34997100
H	-4.13938200	-2.20610600	-2.08999900
H	-3.62718500	-0.60876000	-1.50787800
C	-2.09956200	-3.71813500	-1.04687100

H	-1.06921900	-4.09589900	-1.05106600
H	-2.65520700	-4.22032100	-1.85350900
H	-2.56639300	-4.00203000	-0.09563000
C	-1.47796500	-1.88391000	-2.64420600
H	-1.49292900	-0.80983200	-2.87086400
H	-2.03984700	-2.40116400	-3.43666100
H	-0.43542600	-2.22107200	-2.68027400
C	1.26586500	0.23261200	-1.25630900
O	1.62510800	-0.15118600	-2.35048900
O	0.06949200	0.96053300	-1.13924100

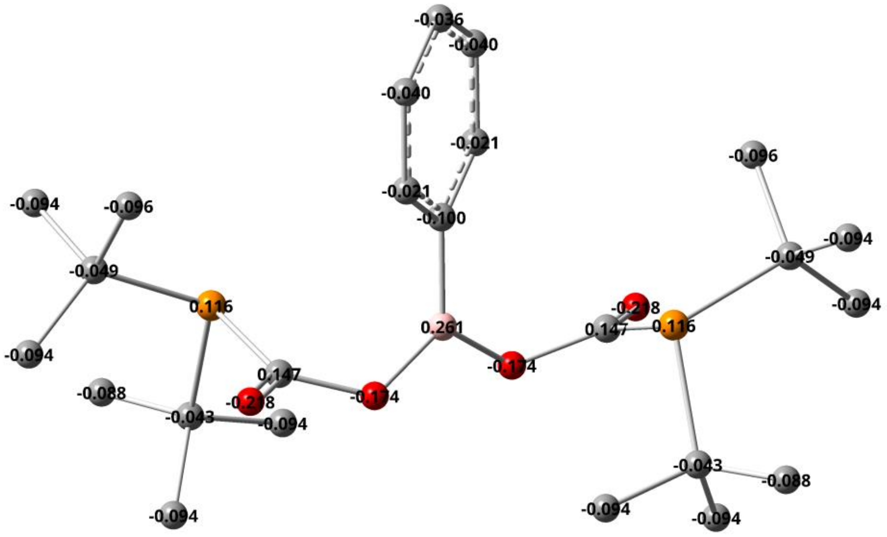

FIG. S68. OPTIMIZED STRUCTURE **1B_I3**

B	-0.00000100	-0.19327900	0.00000600
C	-0.00000100	1.36385100	0.00000100
P	3.18293500	-0.03546000	0.39268800
C	0.40212900	2.08971600	-1.14396100
H	0.70431700	1.55556900	-2.04329000
P	-3.18293400	-0.03546600	-0.39268900
C	0.39587400	3.48904100	-1.14816300
H	0.69786700	4.03082600	-2.04292400
C	0.00000000	4.19175400	-0.00001100
H	0.00000000	5.28058800	-0.00001500
C	-0.39587400	3.48905000	1.14814700
H	-0.69786600	4.03084300	2.04290300
C	-0.40212900	2.08972600	1.14395600
H	-0.70431700	1.55558500	2.04329000
C	3.52981400	-1.81408700	1.05970500
C	3.70511400	-2.89668700	-0.02370600
H	2.78591400	-3.03360500	-0.60570400
H	3.93681500	-3.85576100	0.46420700
H	4.51922800	-2.66366900	-0.71707900
C	4.78815500	-1.75302700	1.95206100
H	5.70157400	-1.59986900	1.36647800
H	4.88856000	-2.71192900	2.48149900
H	4.71476400	-0.95880000	2.70633400
C	2.31825300	-2.17392600	1.95094800

H	2.17061500	-1.43148700	2.74507600
H	2.50339000	-3.15095000	2.42224900
H	1.39142300	-2.25172200	1.37340600
C	4.72833300	0.71170500	-0.43382400
C	4.19130700	1.88839200	-1.28923700
H	3.64351000	1.53018900	-2.16608500
H	5.04518500	2.48844500	-1.63710300
H	3.53247000	2.54291900	-0.70310100
C	5.57545600	-0.22614400	-1.31290600
H	6.04788700	-1.02079900	-0.72270300
H	6.38164000	0.35643200	-1.78472000
H	4.97066300	-0.67904700	-2.10531900
C	5.59575000	1.31832500	0.69638200
H	5.00659200	1.99200600	1.33236000
H	6.40990300	1.90274400	0.24317500
H	6.05052200	0.55294700	1.33387900
C	-4.72833500	0.71170800	0.43381000
C	-4.19131200	1.88840700	1.28920900
H	-3.64351600	1.53021700	2.16606300
H	-5.04519200	2.48846300	1.63706600
H	-3.53247500	2.54292800	0.70306500
C	-5.59575100	1.31831200	-0.69640400
H	-5.00659400	1.99198500	-1.33239100
H	-6.40990500	1.90273600	-0.24320700
H	-6.05052100	0.55292500	-1.33389300
C	-5.57545800	-0.22613100	1.31290300
H	-6.04789000	-1.02079200	0.72270900
H	-6.38164200	0.35645100	1.78471000
H	-4.97066500	-0.67902500	2.10532100
C	-3.52980900	-1.81409800	-1.05969100
C	-4.78815800	-1.75305000	-1.95203700
H	-5.70157300	-1.59989300	-1.36644700
H	-4.88856300	-2.71195700	-2.48146600
H	-4.71477800	-0.95882800	-2.70631600
C	-3.70509400	-2.89669200	0.02372900
H	-2.78588800	-3.03359800	0.60572200
H	-3.93678900	-3.85577200	-0.46417600
H	-4.51920500	-2.66367600	0.71710500
C	-2.31825300	-2.17393700	-1.95094200
H	-2.17062700	-1.43150400	-2.74507800
H	-2.50338900	-3.15096500	-2.42223300
H	-1.39141800	-2.25172300	-1.37340700
C	2.17333400	-0.54344500	-1.10661700
O	2.50030700	-0.58914400	-2.27607800
O	0.89523900	-0.96406300	-0.73429300
C	-2.17333800	-0.54343800	1.10662400
O	-2.50031500	-0.58912200	2.27608400
O	-0.89524200	-0.96405700	0.73431000

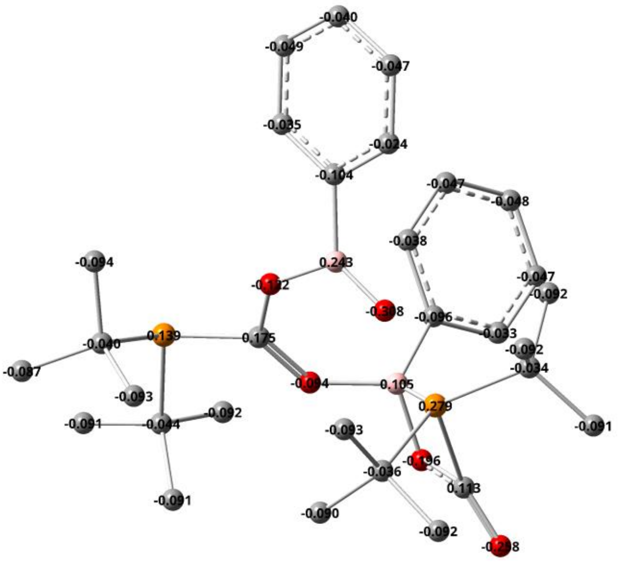

FIG. S69. OPTIMIZED STRUCTURE **1B_I4**

B	-0.61821100	-1.13832000	1.00638300
C	-1.13690400	-0.24419600	2.21670700
P	-1.72152000	-1.28104500	-0.67268300
C	-1.97816000	-0.85466700	3.17607800
H	-2.19394000	-1.91790800	3.08466200
P	3.32566700	0.28581100	0.58462800
C	-2.53138300	-0.12673000	4.23742900
H	-3.17420000	-0.62736600	4.96038300
C	-2.25860300	1.24294700	4.36887300
H	-2.69331900	1.81446500	5.18743400
C	-1.41289600	1.86731800	3.44172800
H	-1.18540500	2.92843100	3.53381100
C	-0.85712600	1.12895700	2.38831600
H	-0.19961900	1.65327200	1.69920800
C	-1.02555200	-2.02372800	-2.29394200
C	-2.00424800	-3.10687000	-2.81060700
H	-2.22549600	-3.86050300	-2.04818500
H	-1.53181900	-3.60956000	-3.66704500
H	-2.94547700	-2.67210900	-3.16371700
C	-0.77880500	-0.98916500	-3.40961300
H	-1.68174800	-0.42840100	-3.66462100
H	-0.44540000	-1.53662700	-4.30402300
H	-0.00371300	-0.26962900	-3.13386200
C	0.31985900	-2.70670400	-1.95337900
H	1.06201800	-1.98131400	-1.60859900
H	0.70864000	-3.17671800	-2.86802900
H	0.21369300	-3.48887200	-1.19330800
C	-3.55925500	-0.77324900	-0.76587900
C	-3.82020600	0.29784000	0.31805700
H	-3.66476200	-0.09391400	1.32779200
H	-4.86959000	0.61615300	0.23311600
H	-3.17730000	1.17240000	0.18149100
C	-4.46322100	-1.99436500	-0.48949200
H	-4.29373800	-2.81281200	-1.19623700
H	-5.50978500	-1.66973300	-0.58120500
H	-4.31669700	-2.38833200	0.52155000

C	-3.91330700	-0.16835600	-2.14165200
H	-3.26758100	0.68314900	-2.37380500
H	-4.95570000	0.17919100	-2.09811200
H	-3.84615300	-0.90276200	-2.95192200
C	3.96489300	0.61277600	-1.20438200
C	3.78046300	2.13014000	-1.44553700
H	2.72635200	2.42153500	-1.41569800
H	4.17656200	2.37933700	-2.44108900
H	4.32735100	2.71982700	-0.69930600
C	5.47263800	0.28976200	-1.26226700
H	6.03160100	0.77414900	-0.45180800
H	5.86834600	0.66683900	-2.21622500
H	5.66070600	-0.78914400	-1.22913400
C	3.23121700	-0.18075900	-2.30226200
H	3.31241300	-1.26225200	-2.15112800
H	3.67908000	0.05928300	-3.27816400
H	2.17082300	0.08797700	-2.35272000
C	3.83807700	-1.41847600	1.27144400
C	5.34112300	-1.32242400	1.63853300
H	5.99378800	-1.29133100	0.76176700
H	5.60961700	-2.21482900	2.22114800
H	5.54726200	-0.44076200	2.25959400
C	3.58980200	-2.62104000	0.34403600
H	2.53116300	-2.71278400	0.08236400
H	3.88835800	-3.54334500	0.86382600
H	4.18330100	-2.55279800	-0.57596900
C	3.06546400	-1.59694300	2.60598800
H	3.17566500	-0.71581100	3.25189400
H	3.49673000	-2.45890800	3.13501100
H	2.00284500	-1.79094600	2.45128000
C	-1.70879700	-2.98769200	0.30829400
O	-2.19840800	-4.10146100	0.28881500
O	-0.76415500	-2.59422600	1.23752000
C	1.52026100	0.11842100	0.25066200
O	0.98729800	1.15237100	-0.34220000
O	0.86632300	-0.88100900	0.70440600
O	-1.25357000	0.61940700	-1.23568100
B	-0.39965500	1.50721500	-0.79701200
C	-0.61202600	3.05656100	-0.74531200
C	-1.82312500	3.59320200	-1.23720200
C	0.33035200	3.95107100	-0.19019300
C	-2.08347300	4.96707500	-1.18275100
H	-2.56071100	2.91401700	-1.66193700
C	0.07533100	5.32713900	-0.12860900
H	1.26818900	3.56243800	0.20477900
C	-1.13273000	5.83763300	-0.62680800
H	-3.02284800	5.36086900	-1.56794000
H	0.81299700	5.99967200	0.30678100
H	-1.33327400	6.90697400	-0.58058000

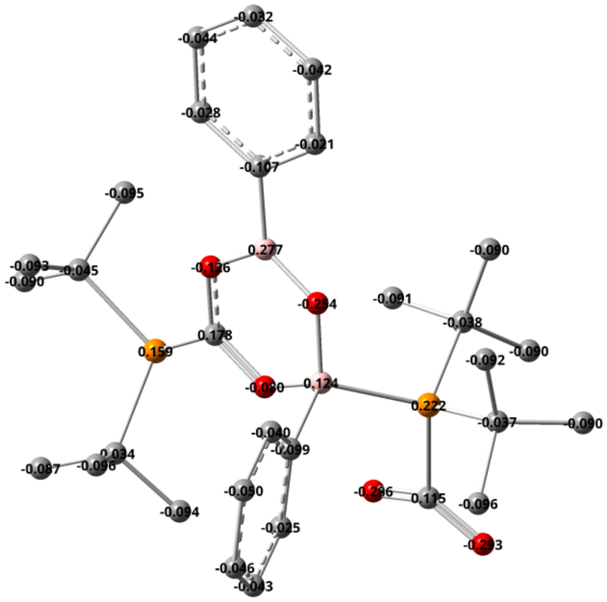

FIG. S70. OPTIMIZED STRUCTURE **1B_I5**

B	0.91492600	-0.00069900	0.46509900
C	1.24439100	-0.70038300	1.85964200
P	-2.70098300	-1.55263800	-1.00556000
C	1.50502300	-2.08776700	1.92725000
H	1.49558900	-2.67899300	1.01215900
P	2.41402600	-0.16961900	-0.86853000
C	1.78868000	-2.71187000	3.14757900
H	1.99414800	-3.78096200	3.17533500
C	1.81892000	-1.95816700	4.33177100
H	2.04655600	-2.44032500	5.28124300
C	1.55663400	-0.58139200	4.28646000
H	1.57743200	0.00833800	5.20207700
C	1.26953900	0.03690200	3.06015600
H	1.06891600	1.10792400	3.03096800
C	-2.43527200	-3.06001100	0.16839400
C	-2.15326100	-2.68085500	1.63627400
H	-1.21129300	-2.13583000	1.74887000
H	-2.06222300	-3.60686300	2.22283200
H	-2.96594600	-2.08717600	2.07055200
C	-3.68704100	-3.96036200	0.10773900
H	-4.53387900	-3.52683100	0.65261000
H	-3.43639700	-4.91547300	0.59059800
H	-3.99534300	-4.17800200	-0.92255200
C	-1.23834800	-3.83013300	-0.43743900
H	-1.48804400	-4.22001600	-1.43200000
H	-1.00920700	-4.68213300	0.21974500
H	-0.33766800	-3.21744200	-0.53796700
C	-4.36761500	-0.65744800	-0.75548100
C	-4.31529900	0.58805400	-1.67854100
H	-3.56153900	1.31340200	-1.36452800
H	-5.29889700	1.07958200	-1.65280200
H	-4.10935400	0.29803800	-2.71683500
C	-4.71022000	-0.25993600	0.69194700
H	-4.82307700	-1.14314500	1.33169200
H	-5.66825900	0.28139800	0.70125900
H	-3.94636000	0.39319700	1.12687800
C	-5.47559700	-1.57868100	-1.32641300

H	-5.21646400	-1.94942100	-2.32676200
H	-6.40020000	-0.99071300	-1.41438500
H	-5.68710500	-2.43513600	-0.68108700
C	1.97081700	0.73741700	-2.48468600
C	0.49657300	0.40616600	-2.81756300
H	0.30360700	-0.67123000	-2.76961200
H	0.28482100	0.75843600	-3.83752200
H	-0.19041800	0.93497100	-2.14590500
C	2.11614400	2.26915500	-2.36303200
H	1.53558700	2.66473200	-1.52169600
H	1.73677100	2.73077500	-3.28711300
H	3.16050300	2.57746200	-2.24405300
C	2.84483600	0.20447700	-3.64431700
H	3.91324200	0.36189000	-3.47366600
H	2.56335900	0.73969300	-4.56348500
H	2.67593300	-0.86577400	-3.80618800
C	3.99972600	0.50621500	-0.07287600
C	3.74444200	1.84596800	0.65278700
H	3.41960800	2.63893600	-0.02889500
H	4.68372500	2.16861500	1.12682600
H	2.98982000	1.73493900	1.43921600
C	5.11139400	0.66251100	-1.13282900
H	5.27736000	-0.28268900	-1.66205200
H	6.04247700	0.93830800	-0.61630600
H	4.89029000	1.45422000	-1.85790900
C	4.48055200	-0.53827000	0.96694500
H	3.77999000	-0.63718600	1.80082600
H	5.44226000	-0.18838100	1.37119700
H	4.63046700	-1.51652500	0.49911000
C	-1.46829300	-0.41092500	-0.27385100
O	-1.79081500	0.85309700	-0.00536500
O	-0.27737000	-0.83891300	-0.13933100
O	0.48568800	1.39655400	0.58615700
B	-0.77181800	1.81118800	0.40014900
C	-1.23551000	3.27718600	0.57386800
C	-2.58753900	3.66941700	0.45191500
C	-0.27238000	4.27085300	0.86636400
C	-2.96604500	5.00726300	0.61478700
H	-3.34608900	2.91985300	0.23333700
C	-0.64545300	5.60939500	1.02542500
H	0.77199400	3.97862000	0.96630500
C	-1.99433700	5.97860400	0.89952100
H	-4.01192600	5.29415600	0.52034200
H	0.10724700	6.36382200	1.24734100
H	-2.28699100	7.01984200	1.02420400
C	2.65939400	-2.07217000	-1.48068200
O	3.84018500	-2.44290500	-1.51232700
O	1.54914400	-2.55743000	-1.77335400

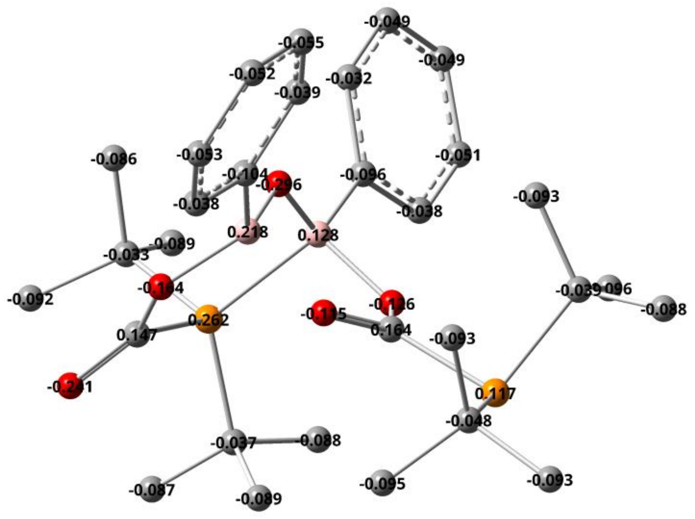

FIG. S71. OPTIMIZED STRUCTURE **1B_I6**

B	-1.02990000	0.39336100	0.58226500
C	-1.77104100	1.47369300	1.49105900
P	2.36867400	1.64387500	-1.42841800
C	-2.21668700	2.72263200	1.00886900
H	-2.06169500	2.98144700	-0.03704900
P	-2.24856900	-0.61341800	-0.80038500
C	-2.85119000	3.64849200	1.84779400
H	-3.18520000	4.60523700	1.44809300
C	-3.05184600	3.34447000	3.20299000
H	-3.54780900	4.05982300	3.85728600
C	-2.59788700	2.11855800	3.71051600
H	-2.73375100	1.88102100	4.76489600
C	-1.96252200	1.20069200	2.86205200
H	-1.59100700	0.25801100	3.26261500
C	2.72476900	2.67249200	0.17028600
C	2.65960700	1.86545500	1.48384600
H	1.64706900	1.49682800	1.68294800
H	2.93692300	2.52646100	2.31881600
H	3.34379600	1.01163600	1.48555400
C	4.11498500	3.32574300	0.02495300
H	4.92445600	2.59237700	0.11094300
H	4.24075700	4.05676200	0.83691300
H	4.21999500	3.86027500	-0.92814600
C	1.65416300	3.78800000	0.19671200
H	1.70410900	4.40850600	-0.70711300
H	1.83740100	4.43148400	1.07020700
H	0.64405700	3.37600400	0.28628000
C	3.78952300	0.43052100	-1.81273600
C	3.20538500	-0.57943300	-2.83527500
H	2.48776600	-1.26133100	-2.37201400
H	4.03266400	-1.17735200	-3.24469100
H	2.71888500	-0.06232100	-3.67408300
C	4.41389400	-0.32246600	-0.62264600
H	4.92752700	0.36160900	0.06362000
H	5.16629400	-1.02832600	-1.00564100
H	3.66858800	-0.89585100	-0.06321500
C	4.87620000	1.25037700	-2.55334300

H	4.45228700	1.80170700	-3.40317300
H	5.63438300	0.55592700	-2.94315600
H	5.38554200	1.96420600	-1.89816900
C	-3.87813500	-1.15169400	0.01223300
C	-3.48247300	-1.72666200	1.39468700
H	-2.85099100	-2.61777700	1.29674500
H	-4.40588400	-2.01787300	1.91488200
H	-2.95586100	-0.99566400	2.01220600
C	-4.76456500	0.10067400	0.19708300
H	-4.25111400	0.88801300	0.75936000
H	-5.66304800	-0.19203600	0.75858900
H	-5.09399000	0.50745800	-0.76727400
C	-4.65333000	-2.24206600	-0.75847000
H	-5.08998000	-1.86842000	-1.68775600
H	-5.47846300	-2.57338400	-0.11149300
H	-4.02650800	-3.10783300	-0.99174700
C	-2.42114500	0.08713600	-2.55626100
C	-2.63634100	1.61266000	-2.43231600
H	-3.52660300	1.86108300	-1.84274900
H	-2.76931100	2.02978500	-3.44061600
H	-1.76469900	2.09507600	-1.97690200
C	-3.57152700	-0.54840000	-3.36043800
H	-3.47768600	-1.63830100	-3.39906500
H	-3.52539900	-0.15748400	-4.38740900
H	-4.55132000	-0.27991200	-2.94926100
C	-1.09033200	-0.16900200	-3.30449900
H	-0.23324600	0.26992400	-2.78446300
H	-1.15701200	0.31470000	-4.28929500
H	-0.91121900	-1.23778200	-3.45882100
C	1.09912900	0.53514300	-0.65448100
O	1.28262300	-0.71628000	-0.48879300
O	-0.00544100	1.13901300	-0.30821500
O	-0.43284700	-0.67940100	1.28612300
B	0.39235600	-1.59612700	0.58708900
C	1.48967900	-2.35066700	1.45457000
C	2.15531300	-3.50180700	0.98479700
C	1.88205300	-1.82574600	2.70279200
C	3.17764300	-4.10526600	1.73042600
H	1.86322200	-3.92995500	0.02626000
C	2.90323500	-2.42194000	3.45609600
H	1.37264600	-0.94057400	3.08285200
C	3.55658500	-3.56373900	2.96840000
H	3.67717200	-4.99579400	1.35026200
H	3.18832700	-2.00099600	4.41990400
H	4.35100300	-4.03028500	3.54962500
C	-1.37346100	-2.28108100	-1.07300000
O	-1.83683700	-3.05216100	-1.90855100
O	-0.31562500	-2.55988900	-0.33437300

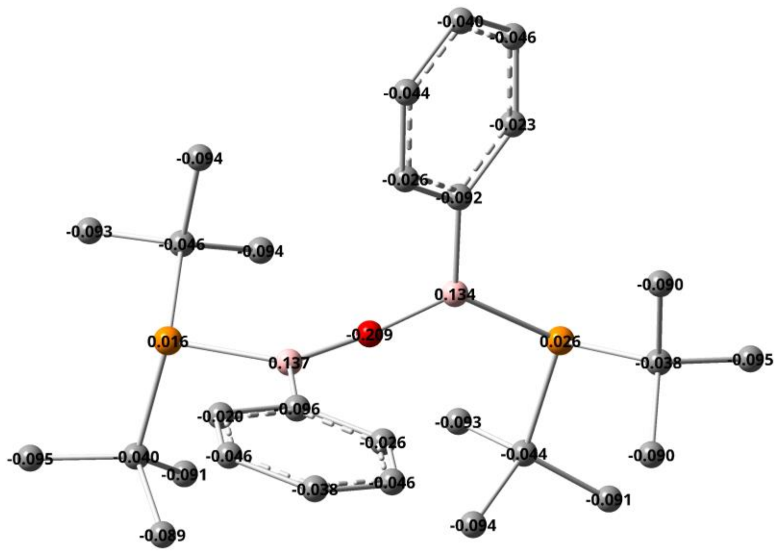

FIG. S72. OPTIMIZED STRUCTURE **1B_I1_PhBO**

B	1.20028800	0.38540600	-0.45193800
C	1.54934800	1.91568100	-0.48586300
P	2.47218200	-0.92996300	-1.06504400
C	2.39362300	2.45788300	-1.48215700
H	2.82628300	1.79086900	-2.22739500
P	-3.01603400	0.43055000	-0.18746200
C	2.65833900	3.83223300	-1.53985600
H	3.29896100	4.23046700	-2.32534500
C	2.09531800	4.69488900	-0.58780800
H	2.30375100	5.76292200	-0.62757900
C	1.25468500	4.17886800	0.41088300
H	0.81251200	4.84493700	1.15023600
C	0.97575600	2.80785700	0.45036900
H	0.31436800	2.42168300	1.22536600
C	1.61419700	-2.63918300	-1.03050200
C	2.64044900	-3.74315300	-1.36555300
H	3.20258200	-3.50327000	-2.27734000
H	2.10595800	-4.69063600	-1.53251300
H	3.35476500	-3.90820600	-0.55025700
C	0.85919700	-3.00940200	0.26333900
H	1.52548700	-3.08709300	1.12786300
H	0.37113600	-3.98767600	0.12871500
H	0.08047200	-2.27639800	0.49310800
C	0.59641900	-2.56735200	-2.19379500
H	-0.15361900	-1.78967900	-2.01976100
H	0.07279500	-3.53189200	-2.27914800
H	1.09562100	-2.35837300	-3.14822100
C	3.99213700	-0.89884300	0.12190700
C	4.31535500	0.55839900	0.51189300
H	4.45090600	1.20047600	-0.36536900
H	5.25568700	0.56321000	1.08385300
H	3.53137000	1.00082500	1.13690800
C	5.20024700	-1.46115500	-0.66483900
H	5.04765200	-2.50214000	-0.96958700
H	6.09886300	-1.42325900	-0.02994000
H	5.38805000	-0.86625800	-1.56676700
C	3.80019300	-1.71007200	1.41838500

H	2.95593600	-1.33460400	2.00807300
H	4.70888600	-1.61860200	2.03377700
H	3.64142700	-2.77596000	1.22328800
C	-3.81571700	-1.32022100	-0.12293800
C	-3.47442900	-1.94441200	1.24811600
H	-2.40685300	-2.18247100	1.33479400
H	-4.04233100	-2.88010100	1.36398900
H	-3.74167800	-1.28132900	2.07964900
C	-5.34947400	-1.14532900	-0.20390900
H	-5.71297800	-0.50207900	0.60675300
H	-5.83679000	-2.12864500	-0.11322500
H	-5.66226100	-0.70184500	-1.15575600
C	-3.34285700	-2.28021700	-1.22986000
H	-3.64558200	-1.93595300	-2.22549700
H	-3.79964300	-3.27003600	-1.06996700
H	-2.25393600	-2.40248900	-1.21856700
C	-2.78524100	1.04606100	-1.98405000
C	-4.17359700	1.12558400	-2.65541900
H	-4.56614200	0.12843900	-2.89226000
H	-4.08988800	1.68153000	-3.60173400
H	-4.90128300	1.64259500	-2.01628900
C	-1.81809500	0.28102200	-2.90897100
H	-0.80209600	0.27560600	-2.50646500
H	-1.78867100	0.78445800	-3.88836300
H	-2.13106900	-0.75362900	-3.07815200
C	-2.24137400	2.48485200	-1.79662700
H	-2.92159500	3.09360600	-1.18748300
H	-2.13274800	2.96458400	-2.78132700
H	-1.25412100	2.48460200	-1.31718100
O	-0.10957000	0.05874900	-0.21122800
B	-1.23691400	0.18081200	0.55353100
C	-1.03230700	0.21814900	2.11152500
C	0.18047600	-0.23005200	2.68533100
C	-2.02218400	0.72343400	2.98702500
C	0.39599400	-0.19366200	4.06636300
H	0.96226500	-0.61755200	2.03247300
C	-1.81271700	0.77030000	4.37103200
H	-2.95263100	1.10068900	2.56374400
C	-0.60448100	0.30819000	4.91435200
H	1.33714900	-0.55004100	4.48285000
H	-2.58519900	1.17191700	5.02512200
H	-0.43988100	0.34482300	5.99014900

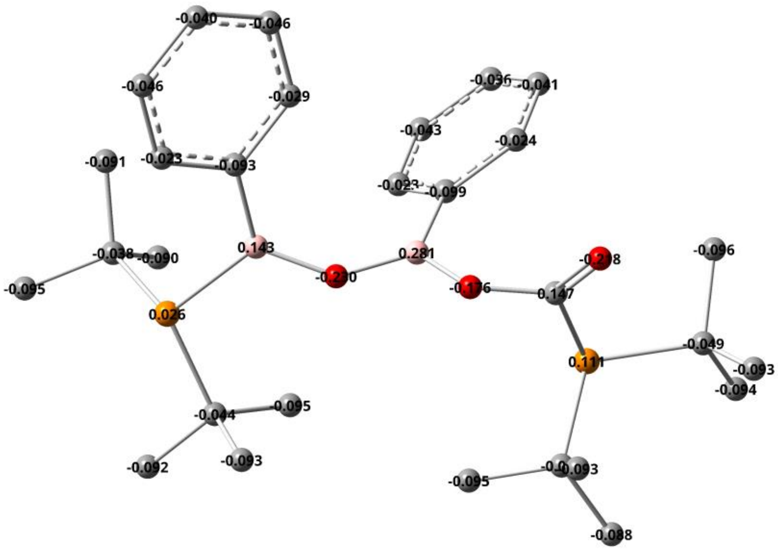

FIG. S73. OPTIMIZED STRUCTURE **1B_I2A_PhBO**

B	-2.17863100	-0.05231500	-0.70328700
C	-2.83013600	-1.35284100	-1.29306800
P	-3.29411400	1.47159400	-0.30854300
C	-3.96586600	-1.27075700	-2.13414800
H	-4.37969500	-0.28977200	-2.36558500
P	3.43092200	0.40836600	0.16935700
C	-4.54832400	-2.41839200	-2.68660700
H	-5.41293000	-2.32729300	-3.34243000
C	-4.01689800	-3.68318200	-2.39577800
H	-4.47109100	-4.57790200	-2.81874900
C	-2.89108600	-3.79069000	-1.56472200
H	-2.47261100	-4.76975700	-1.33640600
C	-2.29988900	-2.63880000	-1.03299800
H	-1.42800000	-2.74372500	-0.38953700
C	-2.14258300	2.91705200	0.17298100
C	-2.98356500	4.12714000	0.63441700
H	-3.78183900	4.35791400	-0.08277900
H	-2.33030000	5.00930500	0.71513200
H	-3.43743500	3.96318100	1.61940900
C	-1.05740000	2.61627500	1.22813900
H	-1.48717600	2.36280400	2.20216700
H	-0.42999600	3.51129400	1.36488600
H	-0.40679100	1.79720100	0.91098000
C	-1.46018400	3.29415600	-1.16614900
H	-0.89023500	2.45492400	-1.57996400
H	-0.76225700	4.12822300	-0.99727100
H	-2.20284400	3.60827100	-1.91009100
C	-4.47092600	1.00019000	1.14340300
C	-4.97568000	-0.44539200	0.95421100
H	-5.43606700	-0.59665800	-0.02829600
H	-5.73825900	-0.64830300	1.72149900
H	-4.17112900	-1.18088600	1.06671400
C	-5.69384000	1.94550400	1.07147100
H	-5.41347800	2.99572100	1.20681900
H	-6.40735200	1.68103100	1.86713100
H	-6.20230100	1.85049800	0.10449900

C	-3.82163700	1.10383700	2.53742900
H	-2.94663500	0.44882000	2.62260000
H	-4.55450100	0.79088100	3.29764500
H	-3.51570000	2.12776900	2.77799100
C	5.15783900	-0.35373900	0.44276200
C	4.89290900	-1.80141700	0.92890800
H	4.16689000	-1.82202800	1.75249000
H	5.83785100	-2.22701200	1.29801000
H	4.52586700	-2.43588400	0.11630500
C	6.11366100	-0.38247600	-0.76387500
H	5.66273500	-0.90655000	-1.61284000
H	7.03358600	-0.91562000	-0.47843100
H	6.40480700	0.62710200	-1.07791000
C	5.80316700	0.41806500	1.62035800
H	6.05931700	1.44956400	1.35690200
H	6.73245900	-0.09292900	1.91225000
H	5.13807300	0.44006500	2.49365700
C	3.54470400	1.97663600	-0.95193100
C	3.87760700	1.70171300	-2.43195200
H	4.82321000	1.16521100	-2.55407000
H	3.95921000	2.66239200	-2.96301600
H	3.08660300	1.11919200	-2.91903500
C	4.59002100	2.93580500	-0.34410500
H	4.40629100	3.11506500	0.72328400
H	4.52221100	3.90241700	-0.86487200
H	5.61303100	2.56350800	-0.46786600
C	2.15659500	2.64664000	-0.86111600
H	1.37109600	2.00026400	-1.26473600
H	2.16548800	3.57485600	-1.45206400
H	1.90087500	2.89907300	0.17480500
O	-0.80291800	0.05390600	-0.69458400
B	0.34279000	-0.63951200	-0.42697400
C	0.45964300	-1.51858100	0.86217000
C	-0.36094400	-1.22827500	1.97492500
C	1.32720000	-2.63022000	0.95237800
C	-0.30517300	-1.99821900	3.14138300
H	-1.04179800	-0.37965100	1.92358600
C	1.37673100	-3.41464600	2.11155800
H	1.95274100	-2.89991300	0.10397100
C	0.56592100	-3.09639700	3.21089800
H	-0.93855700	-1.74834000	3.99106100
H	2.04483500	-4.27314300	2.15653500
H	0.60815400	-3.70320400	4.11389000
C	2.71688400	-0.74492600	-1.13233400
O	3.28863300	-1.59798800	-1.78382300
O	1.36656300	-0.49249500	-1.36169500

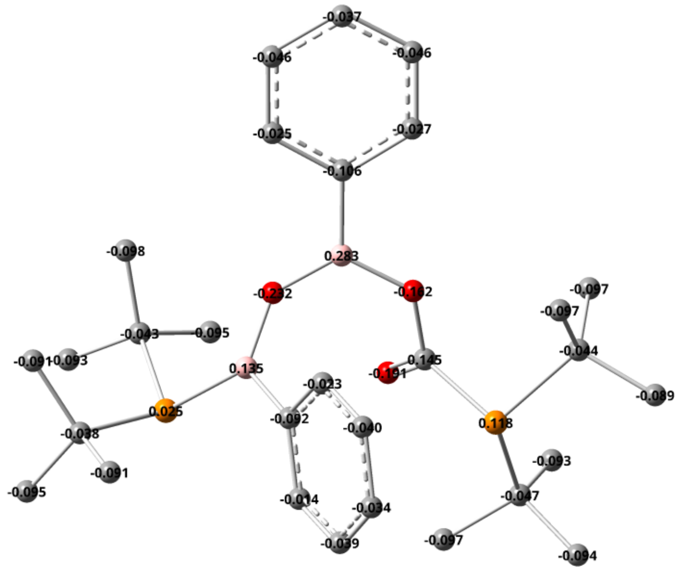

FIG. S74. OPTIMIZED STRUCTURE **1B_I2B_PhBO**

B	-1.50117400	-0.24640600	0.32019800
C	-0.61256200	-0.86991800	1.45213000
P	-2.92962600	-1.24135700	-0.49657000
C	-0.36857300	-2.26103500	1.52168900
H	-0.81755900	-2.90980300	0.77064600
P	2.84757900	-0.97958700	0.01910000
C	0.45766400	-2.80538100	2.51164200
H	0.64238200	-3.87850700	2.53680900
C	1.05564800	-1.96847300	3.46430800
H	1.70101300	-2.38984800	4.23347000
C	0.83080300	-0.58438300	3.41661500
H	1.29637200	0.07048000	4.15159100
C	0.01313200	-0.04592000	2.41759600
H	-0.15372700	1.03222100	2.39459500
C	-3.57742900	-0.17946500	-1.95125100
C	-4.84242000	-0.83264100	-2.54843300
H	-4.68982200	-1.90178600	-2.74546100
H	-5.08393900	-0.34004500	-3.50261100
H	-5.71328700	-0.71954500	-1.89071300
C	-3.86390400	1.30942400	-1.65882400
H	-4.68021900	1.44161600	-0.94167200
H	-4.16119300	1.80439400	-2.59705800
H	-2.97498900	1.81580900	-1.27389200
C	-2.44235800	-0.27261300	-3.00126800
H	-1.49786600	0.13215800	-2.62243700
H	-2.73166800	0.30214200	-3.89467500
H	-2.26599500	-1.31323100	-3.30127600
C	-4.28884600	-1.48046200	0.85000700
C	-3.62402500	-1.89010900	2.18077300
H	-2.97050300	-2.76208400	2.06250200
H	-4.41620900	-2.15549500	2.89739500
H	-3.02939000	-1.07791600	2.61346900
C	-5.19854600	-2.64792200	0.40013000
H	-5.71636400	-2.43225100	-0.54025800
H	-5.96235400	-2.83287100	1.17131300
H	-4.61420800	-3.56620300	0.26470300
C	-5.14188600	-0.22150100	1.10002800

H	-4.51915700	0.63508900	1.38636400
H	-5.84991200	-0.42156500	1.91990400
H	-5.73008900	0.05579100	0.21825000
C	3.22906100	-2.44070500	-1.15014100
C	1.94292900	-3.30782800	-1.16881200
H	1.65385300	-3.59842700	-0.15119300
H	2.15479100	-4.22504300	-1.73865100
H	1.10445800	-2.78875700	-1.63946600
C	3.62887800	-2.05673300	-2.58603900
H	2.85999000	-1.43713000	-3.06070300
H	3.74904400	-2.97095700	-3.18733800
H	4.58531700	-1.51988400	-2.60806600
C	4.33519000	-3.30441300	-0.49572300
H	5.32147500	-2.83265800	-0.52981500
H	4.40306000	-4.25662300	-1.04195400
H	4.09446100	-3.53224400	0.55124800
C	4.21958600	0.37248000	-0.05395600
C	4.23957700	1.22848500	-1.33708300
H	4.40121300	0.61823700	-2.23211300
H	5.06448000	1.95483800	-1.27033300
H	3.30735200	1.78850200	-1.46261600
C	5.59869200	-0.29757500	0.12512200
H	5.61662300	-0.98195900	0.98305700
H	6.34700700	0.48840100	0.30512800
H	5.90596300	-0.84674600	-0.77213100
C	3.96333800	1.27419400	1.17813400
H	2.98027800	1.75211000	1.13135900
H	4.73190200	2.06151800	1.21212900
H	4.02038700	0.69349500	2.10738200
O	-1.14631000	1.01154800	-0.14511900
B	0.01306800	1.74295000	-0.17568500
C	-0.02529700	3.27989500	0.06624400
C	-1.23934400	3.91882000	0.40575400
C	1.13962300	4.07337800	-0.03491800
C	-1.29023600	5.29865200	0.63536900
H	-2.14633100	3.32135400	0.48672100
C	1.09523800	5.45378000	0.19252600
H	2.08432300	3.59882600	-0.29517700
C	-0.12126000	6.06801800	0.52853400
H	-2.23422900	5.77504500	0.89498800
H	2.00193000	6.05077400	0.10846700
H	-0.15811700	7.14181700	0.70568600
C	1.48309300	-0.11921000	-0.90446800
O	0.77926500	-0.56282200	-1.79818500
O	1.26531000	1.15669500	-0.39447100

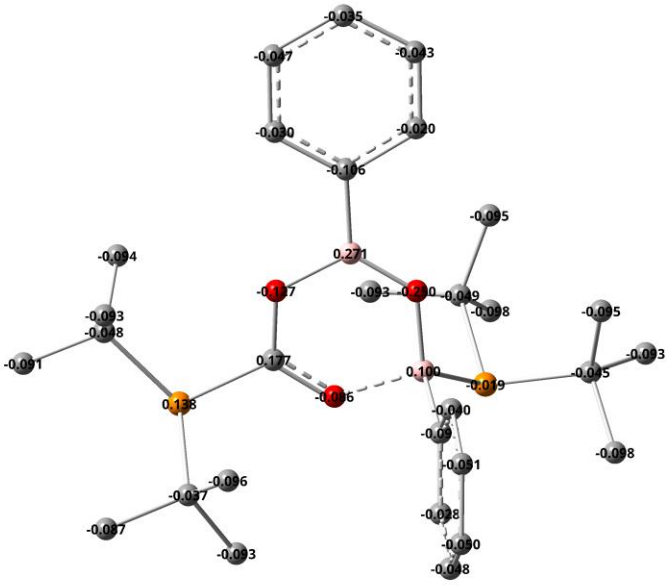

FIG. S75. OPTIMIZED STRUCTURE **1B_I3_PhBO**

B	1.16878000	-0.52584400	0.10116800
C	1.17607100	-1.57269300	1.31389000
P	-2.84813700	-0.92756000	-1.30649000
C	1.16619700	-2.96500100	1.07108400
H	1.23470700	-3.32003100	0.04266900
P	2.53406200	-0.84456000	-1.31631400
C	1.08706500	-3.89031100	2.11860000
H	1.08203500	-4.95827300	1.90308400
C	1.02278100	-3.44141500	3.44758500
H	0.96483900	-4.15766400	4.26597400
C	1.04034200	-2.06507900	3.71429600
H	0.99667500	-1.70851900	4.74290900
C	1.11200200	-1.14593200	2.65613900
H	1.12231300	-0.07791800	2.87406700
C	-2.99281300	-2.47226600	-0.15379200
C	-2.58237400	-2.22077200	1.31183400
H	-1.51957800	-1.97106300	1.40313400
H	-2.74514000	-3.14399400	1.88716700
H	-3.17637000	-1.42589400	1.77556400
C	-4.44474100	-2.99061600	-0.20774000
H	-5.13692100	-2.33349100	0.33116200
H	-4.47459000	-3.97505100	0.28102300
H	-4.80142400	-3.11660700	-1.23804200
C	-2.06313300	-3.53362800	-0.78590000
H	-2.38469900	-3.78546600	-1.80417700
H	-2.10155100	-4.44463000	-0.17079400
H	-1.02474100	-3.19202700	-0.82014800
C	-4.18882200	0.37738200	-0.93911000
C	-3.74605600	1.65483300	-1.69936300
H	-2.85442600	2.11210800	-1.26339200
H	-4.56482900	2.38786900	-1.65540000
H	-3.54622300	1.43660600	-2.75671300
C	-4.46805400	0.69490700	0.54139500
H	-4.87800600	-0.17478000	1.06840700
H	-5.21598100	1.50001600	0.60173400
H	-3.56537700	1.02763300	1.06350200

C	-5.48703900	-0.12061100	-1.62473300
H	-5.30873100	-0.38637500	-2.67484400
H	-6.22678700	0.69243900	-1.60223800
H	-5.92730700	-0.98447700	-1.11818800
C	2.39111800	0.67878200	-2.49176000
C	0.90073800	0.80527200	-2.88551000
H	0.46568400	-0.16232700	-3.16738400
H	0.80926900	1.48583900	-3.74590400
H	0.30634200	1.23591900	-2.07094600
C	2.85414600	2.04020200	-1.93450400
H	2.32739100	2.29218900	-1.00816300
H	2.64416900	2.82897200	-2.67547200
H	3.93154100	2.05864600	-1.73496400
C	3.17230000	0.35251800	-3.78738500
H	4.24736500	0.24862600	-3.60924400
H	3.02895600	1.16459900	-4.51847200
H	2.81132100	-0.58294700	-4.23333800
C	4.18354900	-0.69279800	-0.32782900
C	4.19071200	0.37678000	0.78509400
H	4.00842000	1.38267700	0.39361900
H	5.17335100	0.37796500	1.28496400
H	3.42885700	0.16305500	1.54308000
C	5.35984000	-0.44060700	-1.29581100
H	5.37676300	-1.18238000	-2.10501700
H	6.30583300	-0.52456500	-0.73825900
H	5.32958400	0.55995300	-1.74177500
C	4.42419000	-2.07659800	0.32396600
H	3.66519700	-2.31232900	1.07615200
H	5.40577900	-2.07140600	0.82403900
H	4.42427000	-2.87493400	-0.42926500
C	-1.31979600	-0.22990100	-0.53543400
O	-1.34234200	0.96475100	0.04650200
O	-0.25765600	-0.91079800	-0.64325100
O	1.01546100	0.86166700	0.56212700
B	-0.11420000	1.56593500	0.56181500
C	-0.23420100	3.02417900	1.07100200
C	-1.45948500	3.72721600	1.09774400
C	0.92847000	3.68680300	1.52848500
C	-1.52338400	5.04602400	1.56359600
H	-2.36763900	3.23540000	0.75324300
C	0.87008100	5.00514000	1.99219200
H	1.87760700	3.15295600	1.51376700
C	-0.35757400	5.68656500	2.01005000
H	-2.47530800	5.57426900	1.57890200
H	1.77423600	5.50274700	2.33870600
H	-0.40513300	6.71278300	2.37089200

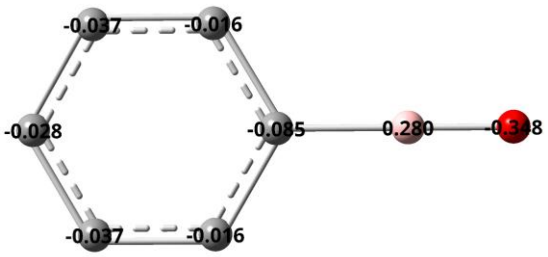

FIG. S76. OPTIMIZED STRUCTURE **1B_PhBO**

O	3.31890700	-0.00000400	0.00000300
B	2.09637000	0.00000800	-0.00000400
C	0.57294800	0.00000800	-0.00000200
C	-0.14545600	1.21867100	-0.00000100
C	-0.14543900	-1.21865800	0.00000000
C	-1.54468200	1.21583200	0.00000100
H	0.39296400	2.16429900	-0.00000100
C	-1.54467100	-1.21584100	0.00000000
H	0.39298600	-2.16428400	0.00000000
C	-2.24442400	-0.00001100	0.00000100
H	-2.08794800	2.15861300	0.00000200
H	-2.08791500	-2.15863400	0.00000100
H	-3.33285100	-0.00001200	0.00000200

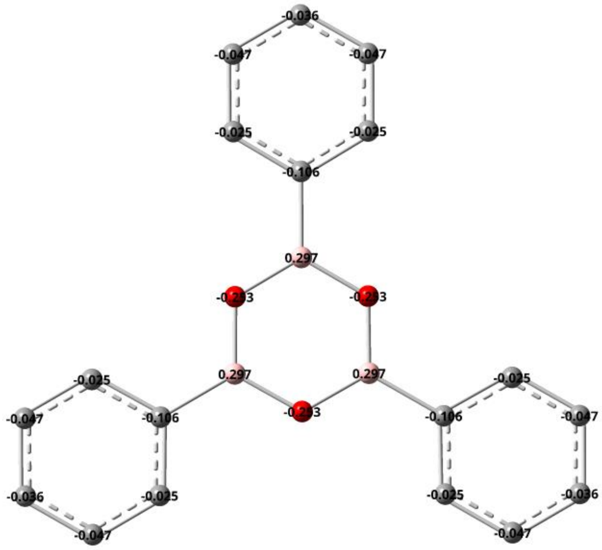

FIG. S77. OPTIMIZED STRUCTURE **1B_[PhBO]$_3$**

O	-1.20500500	0.68379700	0.00019000

O	0.01039400	-1.38524500	0.00016200
O	1.19456000	0.70181500	0.00005400
C	-2.54404900	-1.49428500	0.00028800
C	-0.02215700	2.95053500	0.00005800
C	2.56618000	-1.45590700	0.00004600
C	-2.55470600	-2.90750600	0.00030700
H	-1.60633800	-3.44270200	0.00026700
C	1.17813600	5.08419400	-0.00007300
H	2.11649800	5.63644800	-0.00014300
C	-3.78359500	-0.81541200	0.00033900
H	-3.78925800	0.27353800	0.00032500
C	2.59794500	-2.86880700	0.00008200
H	1.65768000	-3.41811200	0.00014000
C	3.79544900	-0.75860100	-0.00002800
H	3.78483500	0.33031200	-0.00005600
C	3.81384700	-3.56222000	0.00004400
H	3.82291600	-4.65099500	0.00007200
C	-4.99202300	-1.52176000	0.00040900
H	-5.93949300	-0.98528200	0.00044900
C	-0.04319000	5.77605900	-0.00002300
H	-0.05130000	6.86501500	-0.00005400
C	-1.24069000	3.66644100	0.00010700
H	-2.17840400	3.11279000	0.00017600
C	1.18557700	3.68449500	-0.00003300
H	2.13142800	3.14486800	-0.00007100
C	-1.25408400	5.06609800	0.00006700
H	-2.20057100	5.60431000	0.00010600
C	5.02369500	-2.85048000	-0.00003000
H	5.97080900	-3.38793800	-0.00005900
C	-4.98046700	-2.92538100	0.00042700
H	-5.91943900	-3.47693900	0.00048100
C	5.01429000	-1.44684000	-0.00006600
H	5.95363900	-0.89626500	-0.00012300
C	-3.76012400	-3.61898700	0.00037600
H	-3.75293300	-4.70777600	0.00039000
B	1.21596100	-0.68978600	0.00008800
B	-1.20548400	-0.70797800	0.00021000
B	-0.01052400	1.39814700	0.00010100

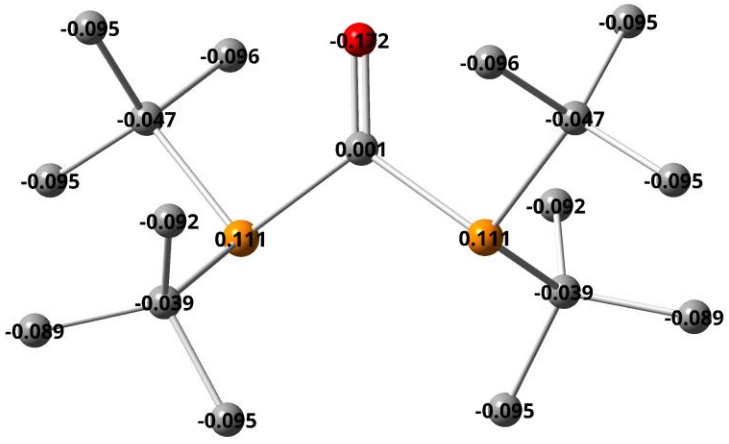

FIG. S78. OPTIMIZED STRUCTURE **1B_*t*Bu$_2$PC(O)P*t*Bu$_2$**

P	-1.55430400	0.39213300	-0.43392700
P	1.55429900	-0.39214400	-0.43392000
O	0.00001000	0.00008100	1.81483700
C	-0.00000500	0.00002900	0.58120700
C	1.96267200	1.37377900	-1.10346200
C	1.20132200	1.51747500	-2.44080300
C	1.55725800	2.51743300	-0.15142100
C	3.47324900	1.46151600	-1.40737600
H	1.51288200	0.74694200	-3.15743600
H	0.11657000	1.44491200	-2.29761400
H	1.42363900	2.50514800	-2.87320300
H	2.01310500	2.41194200	0.83871300
H	1.88453700	3.47783800	-0.57904400
H	0.46883100	2.56161800	-0.02797600
H	3.66431900	2.40323800	-1.94352000
H	4.08042700	1.47105200	-0.49565600
H	3.80865200	0.63609500	-2.04808300
C	2.79456600	-0.91514300	0.92330000
C	3.13660100	0.14314400	1.98935300
C	2.15585900	-2.15550500	1.60095300
C	4.08618400	-1.40266500	0.22180900
H	3.66459600	1.00113000	1.55590100
H	2.23407100	0.49688900	2.49710200
H	3.80363700	-0.30720200	2.74102200
H	1.88611800	-2.91813900	0.85735400
H	2.89496900	-2.60159900	2.28321200
H	1.26753700	-1.89013500	2.18043900
H	4.73374300	-1.88253300	0.97111000
H	3.86104300	-2.14324300	-0.55693700
H	4.65478900	-0.58623300	-0.23354700
C	-2.79459200	0.91515800	0.92326100
C	-4.08622600	1.40261300	0.22175000
C	-3.13659000	-0.14309200	1.98936200
C	-2.15592000	2.15557000	1.60085600
H	-3.86110400	2.14311700	-0.55707200
H	-4.65484100	0.58613600	-0.23351300
H	-4.73377000	1.88255100	0.97101800
H	-2.23404600	-0.49676700	2.49713700
H	-3.80365700	0.30725900	2.74099900
H	-3.66453400	-1.00112700	1.55594700
H	-2.89504800	2.60168700	2.28308100
H	-1.26759800	1.89025000	2.18036400
H	-1.88618300	2.91817100	0.85722100
C	-1.96264900	-1.37381200	-1.10343300
C	-1.20130300	-1.51753000	-2.44077400
C	-1.55720600	-2.51742800	-0.15136000
C	-3.47322500	-1.46159000	-1.40733100
H	-1.51288600	-0.74702700	-3.15742900
H	-0.11655200	-1.44493500	-2.29759200
H	-1.42360100	-2.50522100	-2.87314000
H	-2.01304700	-2.41191000	0.83877400
H	-1.88447100	-3.47785400	-0.57894400
H	-0.46877800	-2.56159100	-0.02792000
H	-3.66427500	-2.40332500	-1.94346200
H	-4.08039700	-1.47113600	-0.49560700
H	-3.80866000	-0.63618800	-2.04804500

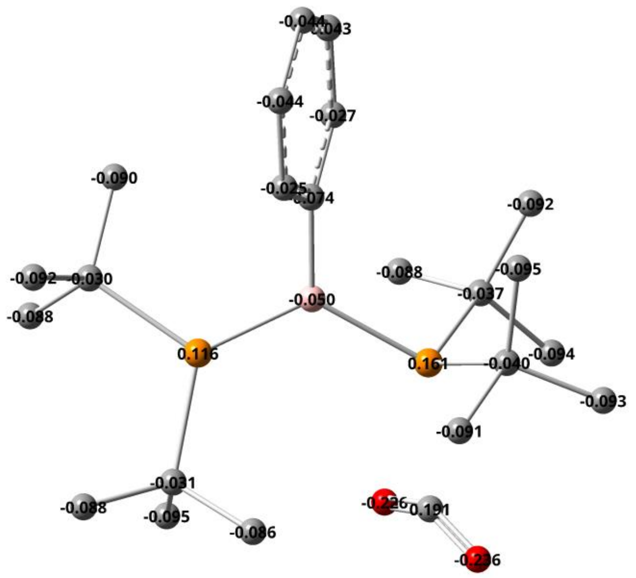

FIG. S79. OPTIMIZED STRUCTURE **1B_TS1**

B	0.25356900	0.34970600	0.00753300
C	0.69997400	1.86643000	-0.09789100
P	-1.65004700	-0.19228500	0.02396400
C	0.54827600	2.85489100	0.90082800
H	0.11472500	2.59021300	1.86175000
P	1.65647100	-0.81399200	-0.07822800
C	1.00001600	4.16684400	0.71378700
H	0.88196000	4.89546700	1.51456300
C	1.61195500	4.54146400	-0.49176700
H	1.96071000	5.56225100	-0.63741400
C	1.77655200	3.58409200	-1.50211800
H	2.25639900	3.85530600	-2.44138700
C	1.33425500	2.26977600	-1.30133700
H	1.47623500	1.53073000	-2.08898900
C	-2.47612200	0.53986400	-1.53298400
C	-4.00055700	0.29802700	-1.47202700
H	-4.23626500	-0.74897800	-1.25531500
H	-4.43317700	0.55508100	-2.45048500
H	-4.47934500	0.94089300	-0.72331500
C	-2.20718400	2.03650000	-1.79303700
H	-2.59998300	2.67822700	-0.99939900
H	-2.71309100	2.31540400	-2.73043500
H	-1.14111700	2.24856500	-1.90903800
C	-1.87852700	-0.27980100	-2.70170100
H	-0.78835100	-0.15409100	-2.75727400
H	-2.30977100	0.08095500	-3.64723100
H	-2.10978500	-1.34567300	-2.59903100
C	-2.39025600	0.54076000	1.63362300
C	-1.35561300	0.24942100	2.74617800
H	-1.14170800	-0.82418900	2.80154600
H	-1.76773400	0.57464600	3.71319400
H	-0.40732400	0.77617500	2.58324400
C	-3.69899200	-0.20401800	1.99645000
H	-4.44893400	-0.13614000	1.20145900
H	-4.12130300	0.26726200	2.89677800
H	-3.51652100	-1.25871800	2.22132800
C	-2.71488600	2.04701700	1.55269300

H	-1.88495800	2.64663900	1.17206400
H	-2.96594100	2.40645700	2.56257300
H	-3.58857300	2.22484700	0.91454400
C	3.37918100	-0.27325100	0.55014500
C	3.30209300	1.06830700	1.31134500
H	3.11140600	1.91432400	0.64757000
H	4.27706700	1.22879000	1.79526600
H	2.53102900	1.05095900	2.08984500
C	3.88046500	-1.32897800	1.56677600
H	3.19624000	-1.40760600	2.42018100
H	4.86226700	-1.00504100	1.94220200
H	4.00681300	-2.32387200	1.13162300
C	4.38111800	-0.11313300	-0.61601400
H	4.53517700	-1.04538600	-1.16755300
H	5.35368600	0.20568100	-0.21168200
H	4.03365600	0.65443400	-1.31745800
C	1.55948600	-2.66169600	-0.51313600
C	1.29261200	-3.54220600	0.72728400
H	2.11201400	-3.47715500	1.45153700
H	1.20744400	-4.59134800	0.40596700
H	0.35832700	-3.25934200	1.21961800
C	2.85035000	-3.12246200	-1.23124500
H	3.05536400	-2.51336700	-2.12017600
H	2.69250300	-4.15925500	-1.56194600
H	3.73511700	-3.11684000	-0.58738700
C	0.41802300	-2.81046100	-1.54212700
H	-0.53457000	-2.43175700	-1.17279600
H	0.28573700	-3.87756900	-1.77103900
H	0.65877800	-2.27990600	-2.47112500
C	-2.31540700	-2.40717400	0.29608700
O	-3.02158100	-2.70399200	-0.64687000
O	-1.83582800	-2.77384900	1.35325000

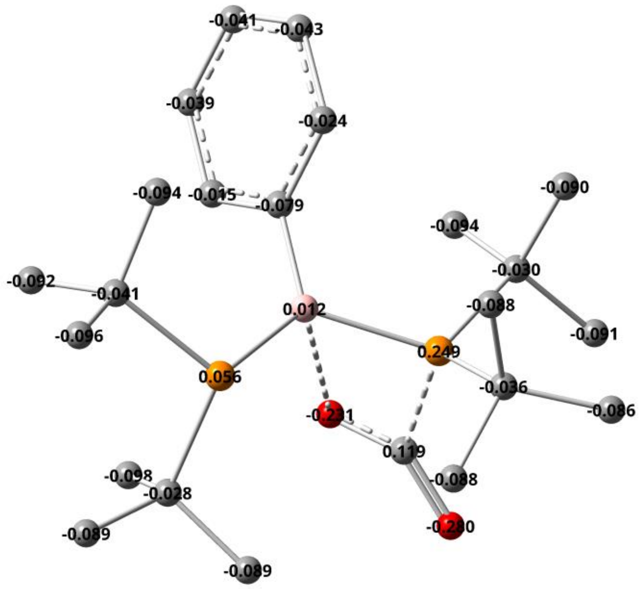

FIG. S80. OPTIMIZED STRUCTURE **1B_TS2**

B	-0.17281700	0.24928200	-0.01119900
C	-0.24011900	1.80617100	-0.13006900
P	1.64123900	-0.52884400	0.05351500
C	-0.79236400	2.37827500	-1.29753400
H	-1.15956200	1.72141900	-2.08380300
P	-1.72214300	-0.77733000	0.49907900
C	-0.82928000	3.76791200	-1.47250700
H	-1.23847500	4.18663000	-2.39050700
C	-0.34736400	4.61814300	-0.46637200
H	-0.38593400	5.69806600	-0.59725700
C	0.18375200	4.06944700	0.71178300
H	0.55081300	4.72269200	1.50191700
C	0.25172300	2.68056900	0.86568000
H	0.65802300	2.26576700	1.78773100
C	1.96570400	-1.56433500	1.61051700
C	3.42379000	-2.05362700	1.72237100
H	3.71558300	-2.63639100	0.84119200
H	3.49082100	-2.71196000	2.60045300
H	4.13891500	-1.23727600	1.86705900
C	1.56246300	-0.73538100	2.85193700
H	2.17489600	0.16621400	2.96771000
H	1.69747700	-1.35430100	3.75120200
H	0.50558800	-0.44397700	2.80013400
C	1.05506600	-2.80735700	1.51003100
H	-0.00332400	-2.52312700	1.47168400
H	1.20724200	-3.41980500	2.41093300
H	1.30371500	-3.41173000	0.63083500
C	3.08401700	0.67864400	-0.33317000
C	2.66644400	1.69957900	-1.41961400
H	2.15711400	1.21752100	-2.25985000
H	3.58722700	2.17212800	-1.79132400
H	2.02050200	2.48378700	-1.01951100
C	4.25178000	-0.16009700	-0.91496900
H	4.61844200	-0.92562100	-0.22704600
H	5.08208900	0.52912100	-1.12507800
H	3.96695400	-0.65045400	-1.85122800
C	3.56476800	1.44580900	0.91647100
H	2.76816800	2.06091000	1.34635000
H	4.37665400	2.12086000	0.60963900
H	3.96226600	0.78337300	1.69290000
C	-3.18276700	0.39271200	0.97000100
C	-2.64238900	1.53808700	1.85907700
H	-1.88329600	1.18316300	2.57011900
H	-3.48047200	1.94787200	2.44203100
H	-2.21473100	2.35030200	1.26958900
C	-3.98368200	1.00984400	-0.19220300
H	-3.33552900	1.60221100	-0.84735900
H	-4.75511200	1.68001500	0.21833500
H	-4.49479300	0.25203000	-0.79611000
C	-4.11951700	-0.44188100	1.88467000
H	-4.58881700	-1.28510700	1.37359200
H	-4.92236300	0.21488900	2.25245100
H	-3.56978700	-0.82894300	2.75201000
C	-2.28680200	-1.98416300	-0.90726300
C	-2.35059400	-1.28548700	-2.28127400
H	-3.04780700	-0.44077800	-2.27242500
H	-2.71038200	-2.01083100	-3.02839000

H	-1.36113500	-0.93709100	-2.58995100
C	-3.67231400	-2.57708900	-0.57109500
H	-3.68546800	-3.05860700	0.41441700
H	-3.89603100	-3.34559500	-1.32573000
H	-4.47372200	-1.83264200	-0.61796900
C	-1.30226900	-3.16946100	-0.96156100
H	-0.27471800	-2.86978500	-1.16976400
H	-1.61264700	-3.83621700	-1.77958600
H	-1.32314500	-3.74356600	-0.02737000
C	1.47247800	-1.42168800	-1.65613300
O	2.05343800	-2.47337100	-1.93053800
O	0.71121900	-0.62872000	-2.29415800

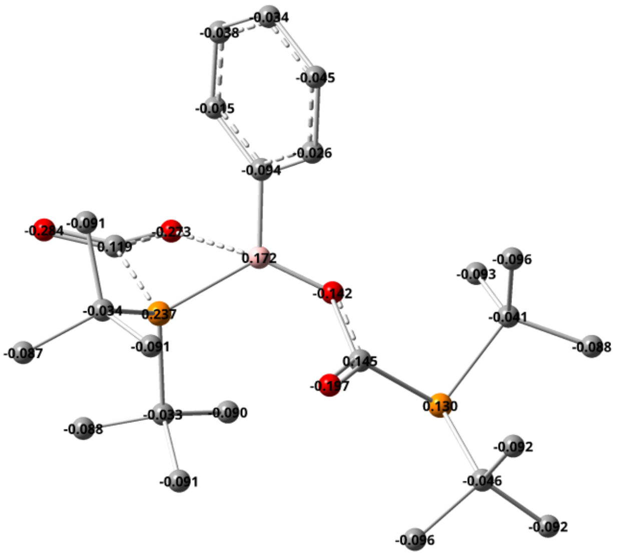

FIG. S81. OPTIMIZED STRUCTURE **1B_TS3**

B	0.87544700	0.64843200	0.20071600
C	1.49505200	2.05735600	-0.02750900
P	-3.00242500	-0.36935900	0.53342600
C	2.63830700	2.59831400	0.60533900
H	3.11334000	2.04925800	1.41261700
P	2.00514400	-0.95380700	0.18556300
C	3.13511800	3.85182500	0.23302800
H	4.00570300	4.25806800	0.74473000
C	2.51973700	4.58381500	-0.79434700
H	2.91777500	5.55385100	-1.08766500
C	1.38484100	4.06704500	-1.43574500
H	0.89929400	4.63040300	-2.23089400
C	0.86993600	2.82610200	-1.04306500
H	-0.01140400	2.43171900	-1.54784400
C	-3.41185000	1.45223700	1.00493400
C	-3.01502500	2.50792200	-0.04657300
H	-1.93013700	2.54561000	-0.18324300
H	-3.33972700	3.49873400	0.30542800
H	-3.49001500	2.32288100	-1.01619500
C	-4.92536400	1.56093500	1.28986100
H	-5.51933500	1.52084900	0.36967500
H	-5.11725700	2.53331300	1.76601200
H	-5.27535300	0.77741500	1.97399900
C	-2.64946400	1.71698800	2.32595500

H	-2.96374800	1.01759700	3.11060400
H	-2.87366700	2.73970100	2.66375700
H	-1.56673600	1.63052700	2.19549500
C	-4.15221400	-1.04884000	-0.82871800
C	-3.53632600	-2.40638600	-1.25843600
H	-2.59985700	-2.27177400	-1.80487400
H	-4.25445700	-2.91969300	-1.91471500
H	-3.35321200	-3.05200200	-0.38888400
C	-4.33778400	-0.13961100	-2.05697400
H	-4.85804900	0.78981800	-1.79530800
H	-4.95105500	-0.66228200	-2.80655800
H	-3.37530000	0.10753900	-2.51877900
C	-5.52250700	-1.36459200	-0.17994800
H	-5.40480300	-1.97228000	0.72684100
H	-6.12233000	-1.94201800	-0.89814800
H	-6.08913500	-0.46520900	0.07685700
C	1.19133600	-2.56281100	0.79319000
C	0.17156900	-2.19567200	1.89724700
H	0.61902400	-1.54578400	2.65713000
H	-0.15166000	-3.12999000	2.37835200
H	-0.72236700	-1.70816900	1.49816500
C	0.48847600	-3.33914700	-0.33928400
H	-0.24974200	-2.72495300	-0.86298400
H	-0.03142800	-4.19921600	0.10709900
H	1.20420000	-3.72967700	-1.07201600
C	2.28740100	-3.44806100	1.43749200
H	3.07582300	-3.72777500	0.73311600
H	1.80635100	-4.37055900	1.79270400
H	2.75296900	-2.95238200	2.29577800
C	2.95117100	-1.10531200	-1.44713800
C	1.98856400	-1.15847200	-2.65137400
H	1.33737400	-2.03766600	-2.62753700
H	2.58721800	-1.19783500	-3.57353200
H	1.35012300	-0.26872100	-2.69291200
C	3.86029800	-2.35208200	-1.40213500
H	4.51661000	-2.33005000	-0.52421400
H	4.49035000	-2.34809400	-2.30298300
H	3.28539200	-3.28494500	-1.40466700
C	3.85133100	0.14919200	-1.56148300
H	3.26398100	1.06568200	-1.67469500
H	4.47572100	0.03315500	-2.45910600
H	4.50892900	0.24979600	-0.69093700
C	-1.41217900	-0.13974600	-0.40169800
O	-1.08843800	-0.60803200	-1.48606200
O	-0.51382700	0.62026500	0.32163600
C	3.13016600	-0.42471700	1.72446100
O	2.34357800	0.11858400	2.53959900
O	4.33606600	-0.68421500	1.68225800

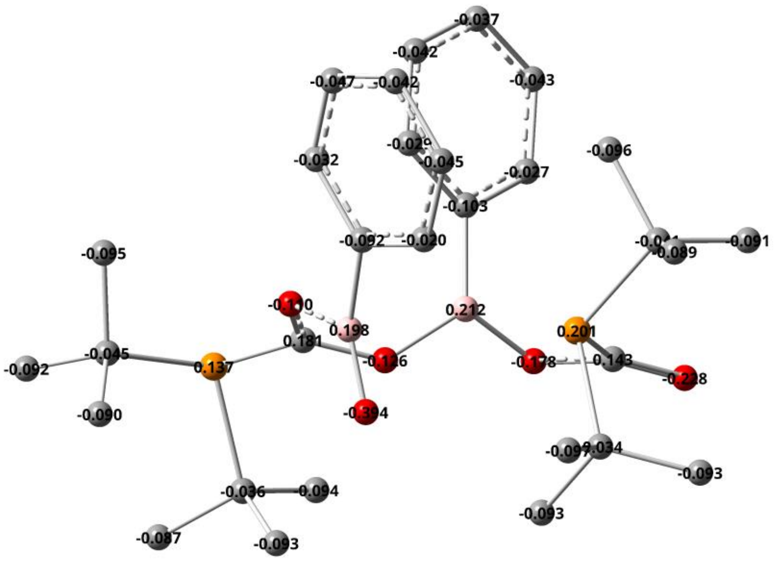

FIG. S82. OPTIMIZED STRUCTURE **1B_TS4**

B	-0.19955600	-0.93036000	1.13384700
C	-0.55969300	0.12433200	2.23743000
P	-2.20737800	-1.06010200	-0.28528400
C	-1.18359500	-0.34535300	3.41923300
H	-1.36504500	-1.41183600	3.53933800
P	3.61701000	-0.23937600	0.69064800
C	-1.56712600	0.53548900	4.43860400
H	-2.04390500	0.14893200	5.33787400
C	-1.34142700	1.91213900	4.29708400
H	-1.64966900	2.60104200	5.08184800
C	-0.71765500	2.39702500	3.13738300
H	-0.54717500	3.46547400	3.01445700
C	-0.32803000	1.51277800	2.12660200
H	0.12013400	1.91205800	1.22393800
C	-2.27257600	-2.16553000	-1.85479600
C	-3.44423200	-3.16690400	-1.80376900
H	-3.42819900	-3.77804300	-0.89508200
H	-3.35765500	-3.83621700	-2.67276300
H	-4.41575100	-2.66352900	-1.87196800
C	-2.34744300	-1.30257600	-3.13298200
H	-3.28314000	-0.74062400	-3.20745900
H	-2.30208400	-1.98467700	-3.99521900
H	-1.48814200	-0.62482900	-3.19219400
C	-0.92023900	-2.91792400	-1.87543600
H	-0.08883500	-2.20441700	-1.90252500
H	-0.88059800	-3.52105900	-2.79394500
H	-0.80339400	-3.59806100	-1.02319500
C	-3.89804300	-0.25855900	0.12007600
C	-3.58362400	0.98967700	0.97929500
H	-3.13903500	0.72258800	1.94299500
H	-4.52569300	1.52262000	1.17537500
H	-2.90287300	1.67481900	0.46034100
C	-4.82742400	-1.20796500	0.90396800
H	-5.04468900	-2.12825900	0.35246100
H	-5.77632000	-0.68233300	1.08917500
H	-4.39677000	-1.48770000	1.87076300
C	-4.60418900	0.21926600	-1.16634400

H	-3.96876100	0.89271900	-1.75188100
H	-5.50182700	0.78113600	-0.86978300
H	-4.92683400	-0.61294900	-1.80066700
C	4.60581900	1.10543500	-0.23291300
C	4.06110800	2.46444600	0.28040100
H	3.05721500	2.67030700	-0.09695200
H	4.73316900	3.26008400	-0.07256400
H	4.04322200	2.49908600	1.37807200
C	6.07554600	1.00763600	0.24778900
H	6.13967600	1.01698100	1.34385600
H	6.62270300	1.88298600	-0.13043600
H	6.58534400	0.11360000	-0.12180000
C	4.53752000	1.04743500	-1.77008800
H	5.03468100	0.15116900	-2.15977200
H	5.05966200	1.92213700	-2.18616600
H	3.50454200	1.06144600	-2.13340400
C	3.89852000	-2.00545000	-0.04369600
C	5.41983200	-2.24919400	-0.13960900
H	5.87828100	-1.66518400	-0.94542100
H	5.58010500	-3.31232700	-0.36984900
H	5.93740000	-2.02772000	0.80261500
C	3.25276500	-2.23369800	-1.42530300
H	2.16522800	-2.12564400	-1.39443600
H	3.48159700	-3.25939200	-1.75227000
H	3.64042300	-1.54337900	-2.18060300
C	3.31162900	-2.99647900	0.98905300
H	3.77877900	-2.86890100	1.97366000
H	3.51408000	-4.02012000	0.64055500
H	2.22926000	-2.88436900	1.09624900
C	-1.93409100	-2.45814700	0.97217600
O	-2.62711600	-3.38054500	1.34924300
O	-0.63304700	-2.29210400	1.36534700
C	1.89339200	0.04584500	0.11181200
O	1.50573500	1.07282100	-0.50936500
O	1.05069500	-0.92611700	0.44523300
O	0.44385100	0.08051900	-2.54079600
B	0.34090600	1.07001100	-1.76616300
C	-0.49996400	2.37635000	-1.60839400
C	-1.69743200	2.48237700	-2.35209200
C	-0.14897300	3.45932400	-0.77423900
C	-2.52818400	3.60378600	-2.23878700
H	-1.96981000	1.67043100	-3.02380700
C	-0.96946500	4.58930200	-0.66244200
H	0.78268400	3.42111300	-0.21131300
C	-2.16837600	4.65920500	-1.38701900
H	-3.45215700	3.65780200	-2.81302500
H	-0.67608300	5.41321500	-0.01332800
H	-2.81202400	5.53273100	-1.29589000

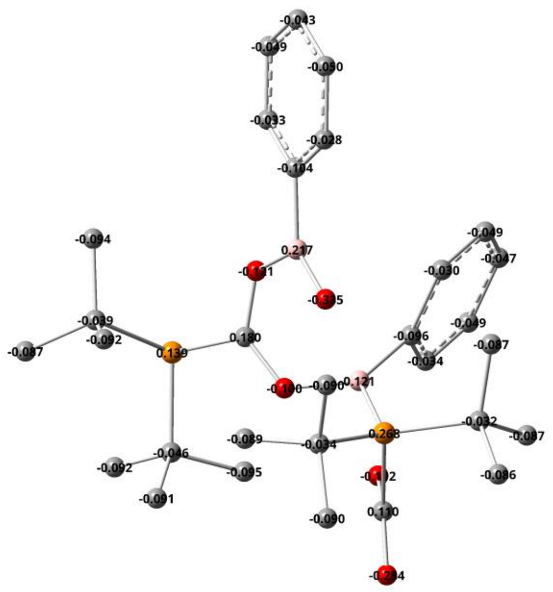

FIG. S83. OPTIMIZED STRUCTURE **1B_TS5**

B	-0.94120900	-0.36926700	0.58420500
C	-1.04743200	0.53745900	1.89163100
P	-2.41208800	-0.90989500	-0.61688700
C	-0.79926800	-0.06855500	3.14651800
H	-0.60354200	-1.13686900	3.18805000
P	3.15580700	-1.12005800	0.66687900
C	-0.82394100	0.67017000	4.33643700
H	-0.62481200	0.17154800	5.28417000
C	-1.10959700	2.04285600	4.31003800
H	-1.13248600	2.61851700	5.23412200
C	-1.36265800	2.66561100	3.08097800
H	-1.57911000	3.73221200	3.04035400
C	-1.32557800	1.92244700	1.89309900
H	-1.50117100	2.43253700	0.95002900
C	-2.12252400	-1.17301300	-2.46140700
C	-2.97871500	-2.36354000	-2.95597000
H	-2.85896700	-3.24449400	-2.31490700
H	-2.64354000	-2.62037900	-3.97118700
H	-4.04290600	-2.11665600	-3.00967000
C	-2.40562000	0.10278000	-3.27879600
H	-3.47362100	0.35137700	-3.28666300
H	-2.10006100	-0.07816300	-4.31979900
H	-1.83437100	0.94964600	-2.88274400
C	-0.62944100	-1.54406700	-2.59679100
H	0.00211400	-0.71246600	-2.27549100
H	-0.42115500	-1.76566900	-3.65320800
H	-0.38217600	-2.43728700	-2.01019600
C	-4.07985400	-0.13862100	-0.18104900
C	-4.31954200	-0.38463400	1.32834200
H	-4.40523600	-1.45378400	1.55431000
H	-5.26758700	0.10036700	1.60094600
H	-3.52507400	0.04267000	1.94739300
C	-5.21076000	-0.82022400	-0.98064700
H	-5.16209000	-0.57730900	-2.04805500
H	-6.17100600	-0.44595000	-0.59817200
H	-5.19668800	-1.90967000	-0.85789000

C	-4.05840000	1.37868500	-0.46391500
H	-3.28534600	1.88022300	0.12307300
H	-5.03524500	1.79552500	-0.17898000
H	-3.88676200	1.60045300	-1.52167000
C	4.00251400	-0.71200600	-1.02020200
C	4.58494800	0.71251700	-0.87223700
H	3.79931000	1.45730200	-0.71337300
H	5.11973800	0.97264200	-1.79784800
H	5.29307000	0.76731500	-0.03613900
C	5.16891600	-1.69919500	-1.23413200
H	5.83825100	-1.73845400	-0.36544400
H	5.75727400	-1.35557400	-2.09722100
H	4.81556400	-2.71235100	-1.45577000
C	3.05294000	-0.75959000	-2.23380600
H	2.56445700	-1.73360100	-2.33870600
H	3.63188900	-0.56784500	-3.14966800
H	2.27844900	0.01349700	-2.16941800
C	2.71871900	-2.96582400	0.84045500
C	4.03189600	-3.68652300	1.23908800
H	4.76310300	-3.72199300	0.42561200
H	3.78751600	-4.72247700	1.51356000
H	4.50138300	-3.20850300	2.10879400
C	2.08908300	-3.64534900	-0.38757100
H	1.16642100	-3.14045300	-0.68763100
H	1.83599000	-4.68467700	-0.13213300
H	2.77853500	-3.67067200	-1.24031300
C	1.75545200	-3.06317900	2.05259000
H	2.14056500	-2.50152200	2.91484000
H	1.68282800	-4.12039100	2.34645200
H	0.74861700	-2.71318500	1.81258700
C	-2.27576400	-2.51994400	0.36862000
O	-2.80159200	-3.61555900	0.19877500
O	-1.46450300	-2.09776700	1.30115300
C	1.54167100	-0.28836400	0.34561800
O	1.60977500	1.00131100	0.21433400
O	0.43291800	-0.94846300	0.32427800
O	-0.57316000	1.16103500	-0.84410200
B	0.49930800	1.79547900	-0.47127300
C	0.90194600	3.29660200	-0.63472800
C	0.04821900	4.14475200	-1.37556900
C	2.05731500	3.86320800	-0.05319600
C	0.33938600	5.50400500	-1.53914200
H	-0.85052400	3.71906300	-1.82096700
C	2.35201800	5.22440700	-0.20694600
H	2.72268300	3.23291300	0.53507000
C	1.49455600	6.04641800	-0.95352300
H	-0.32893900	6.14157600	-2.11635400
H	3.24481700	5.64491000	0.25374800
H	1.72315800	7.10412600	-1.07626500

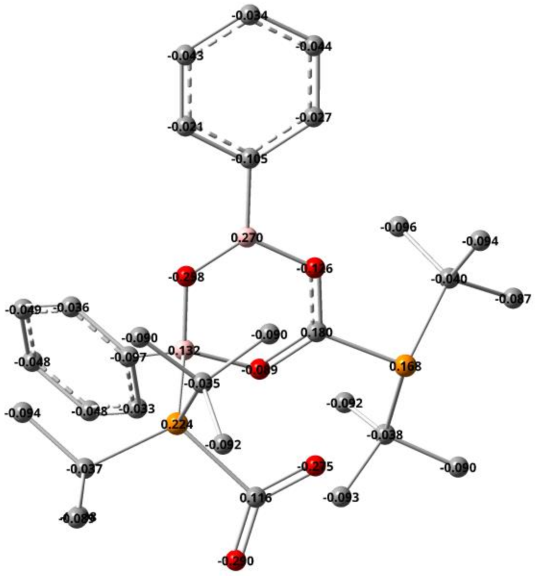

FIG. S84. OPTIMIZED STRUCTURE **1B_TS6**

B	0.94158100	0.12818000	0.64212600
C	1.42115600	-0.36955600	2.08312500
P	-2.55902400	-1.65557600	-0.90343900
C	1.40561900	-1.73052400	2.45782900
H	1.11593100	-2.48438000	1.72731000
P	2.22468300	-0.11162000	-0.94581000
C	1.76580000	-2.13885100	3.74868000
H	1.74762200	-3.19655300	4.00803400
C	2.15313500	-1.18671100	4.70337100
H	2.43745400	-1.50045900	5.70655800
C	2.16451300	0.17234300	4.35815900
H	2.45371600	0.92006000	5.09559900
C	1.79550900	0.57082800	3.06549000
H	1.78845000	1.63047900	2.81205900
C	-2.11247900	-3.32688800	-0.08328100
C	-2.20155400	-3.32156500	1.45369500
H	-1.54338800	-2.56365600	1.89420200
H	-1.88267600	-4.30409300	1.83246400
H	-3.22690700	-3.14945500	1.80293900
C	-3.06729600	-4.39196100	-0.68012900
H	-4.09991300	-4.28728200	-0.33762200
H	-2.71032400	-5.38117700	-0.36142400
H	-3.05402900	-4.36903000	-1.77715500
C	-0.68436100	-3.71109400	-0.54250400
H	-0.56472700	-3.63129100	-1.62778000
H	-0.50932200	-4.75448600	-0.24231800
H	0.08114300	-3.08780300	-0.08016700
C	-4.21891600	-0.91674300	-0.25375600
C	-4.59126800	0.17860900	-1.28325700
H	-3.81600800	0.94665600	-1.36073600
H	-5.52837400	0.66094500	-0.96745100
H	-4.74380500	-0.25510600	-2.27915600
C	-4.18200100	-0.32512100	1.17087400
H	-3.86998200	-1.06773100	1.91288900
H	-5.19487100	0.01249800	1.43725900
H	-3.51692800	0.54051000	1.23921600

C	-5.31081800	-2.00694000	-0.30945700
H	-5.33232100	-2.52552800	-1.27604200
H	-6.28619300	-1.51956200	-0.16764300
H	-5.19217100	-2.74652900	0.49017400
C	2.06378300	1.29925100	-2.23524800
C	0.57219300	1.63629600	-2.47426100
H	-0.02473000	0.72965800	-2.61325700
H	0.50613900	2.23931800	-3.39149400
H	0.16189500	2.25121100	-1.66679900
C	2.78982900	2.57977300	-1.77330900
H	2.45078400	2.89787500	-0.78000400
H	2.56142200	3.38663900	-2.48559100
H	3.87804400	2.45425500	-1.75430000
C	2.62765300	0.81845800	-3.59592900
H	3.67570000	0.51569200	-3.54571900
H	2.55083700	1.65335600	-4.30824800
H	2.03800600	-0.01820700	-3.98526700
C	4.02460200	-0.31910100	-0.35924200
C	4.44679900	0.79056800	0.62886600
H	4.44835300	1.78500100	0.17144700
H	5.47087100	0.57567500	0.96991300
H	3.79968500	0.80881000	1.51139700
C	4.99796700	-0.35640100	-1.55671300
H	4.69655800	-1.11293200	-2.29015600
H	5.99357600	-0.62701200	-1.17574300
H	5.08989700	0.61847900	-2.04916000
C	4.11185200	-1.68825100	0.36069400
H	3.51818400	-1.70112200	1.27822900
H	5.16335300	-1.85268900	0.63984000
H	3.78736400	-2.49875200	-0.29968200
C	-1.36812100	-0.47739500	-0.17845200
O	-1.70010300	0.81377100	-0.21710100
O	-0.22958600	-0.84789200	0.25488000
O	0.43370100	1.49626000	0.69241900
B	-0.80392600	1.83227500	0.30086900
C	-1.36953200	3.27228100	0.38887900
C	-2.69498000	3.59512800	0.02263700
C	-0.53164900	4.31198500	0.85408900
C	-3.16860500	4.90920000	0.11596500
H	-3.35752500	2.81051300	-0.33695400
C	-0.99919700	5.62684800	0.94747900
H	0.49184300	4.07453000	1.14036900
C	-2.32014400	5.92671300	0.57773800
H	-4.19293400	5.14162500	-0.17023600
H	-0.34141800	6.41667400	1.30617300
H	-2.68641600	6.94953100	0.64967600
C	1.62084000	-1.69448500	-2.02482300
O	2.42609300	-2.63142500	-2.10926900
O	0.47652200	-1.46067200	-2.47031900

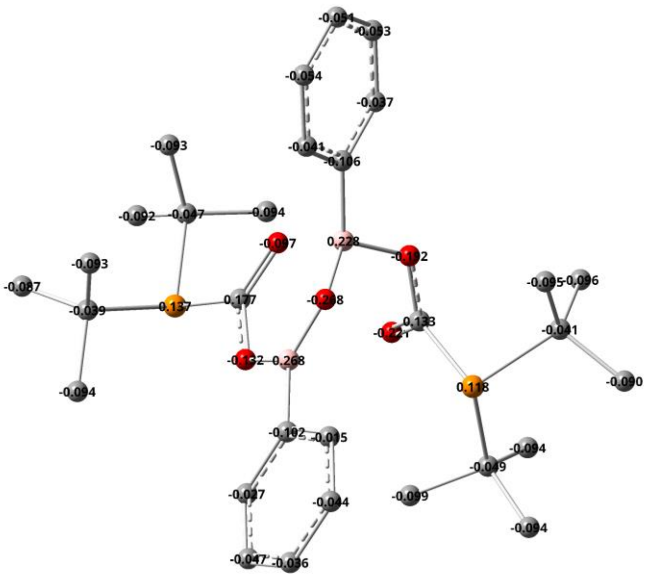

FIG. S85. OPTIMIZED STRUCTURE **1B_TS7**

B	0.17298300	-1.03064900	1.24228000
C	0.99380100	-2.17974300	1.87700500
P	-2.97449600	-1.23292700	-1.34219000
C	0.79442900	-3.53397100	1.52915400
H	0.02520500	-3.79281100	0.80317300
P	2.94718200	0.16558200	-0.60295800
C	1.58462000	-4.54464300	2.09024300
H	1.42330700	-5.58396000	1.80878100
C	2.58883300	-4.21390500	3.01268900
H	3.20569500	-4.99805900	3.44914700
C	2.80305700	-2.87249400	3.36941600
H	3.58752400	-2.61694300	4.07990200
C	2.01395200	-1.86637800	2.80320600
H	2.18789500	-0.82244800	3.05776800
C	-3.97886800	-2.05812400	0.09043800
C	-3.98547500	-1.26663800	1.41445600
H	-2.98247000	-1.20208200	1.85338400
H	-4.62615100	-1.78968600	2.14006800
H	-4.37307600	-0.25076800	1.29018300
C	-5.42814300	-2.26980500	-0.39545100
H	-5.97919300	-1.32557700	-0.46643600
H	-5.94927900	-2.90548300	0.33509300
H	-5.46476700	-2.77594200	-1.36855500
C	-3.32762800	-3.44186400	0.31602100
H	-3.34725900	-4.04510300	-0.59996600
H	-3.88965200	-3.97582800	1.09667300
H	-2.28891800	-3.34922900	0.64882700
C	-3.83804400	0.33033300	-2.00819600
C	-2.76233100	1.06886400	-2.84788000
H	-1.97469100	1.50433300	-2.22963600
H	-3.25991100	1.87838400	-3.40195500
H	-2.29253900	0.39672000	-3.57725800
C	-4.45691000	1.28685000	-0.97192900
H	-5.28680200	0.81694000	-0.42982000
H	-4.86381200	2.16275100	-1.49903200
H	-3.71349400	1.64248600	-0.25203500
C	-4.92760800	-0.16075000	-2.99519700

H	-4.51357200	-0.86077300	-3.73276300
H	-5.32075400	0.70986400	-3.53925600
H	-5.76994100	-0.64526400	-2.49129900
C	3.86423000	1.85999400	-0.62565000
C	3.57757000	2.51019300	0.74860900
H	2.50975800	2.70497900	0.88559600
H	4.11521200	3.46900900	0.80733000
H	3.92400600	1.86861800	1.56890800
C	5.38269000	1.60943700	-0.73793000
H	5.73561000	0.88533400	0.00775300
H	5.90697200	2.56003000	-0.55950600
H	5.66786300	1.25642800	-1.73573600
C	3.41579200	2.81340600	-1.75232300
H	3.56100300	2.37230500	-2.74462300
H	4.01357000	3.73673100	-1.70069600
H	2.36159300	3.09001600	-1.64152100
C	3.52681100	-0.99129100	-2.00107400
C	4.89460900	-1.59199600	-1.59565400
H	5.70984200	-0.86385800	-1.64852500
H	5.13666200	-2.41435700	-2.28526200
H	4.86100900	-2.00272000	-0.57765700
C	3.60739800	-0.36410500	-3.40425500
H	2.64633300	0.07354000	-3.69572700
H	3.86738300	-1.14253500	-4.13841100
H	4.38342600	0.40980000	-3.45607200
C	2.50350900	-2.15807000	-1.99582400
H	2.42063100	-2.60522100	-0.99607800
H	2.85931000	-2.93753700	-2.68656300
H	1.51340100	-1.82841600	-2.32247000
C	-1.64272000	-0.52179900	-0.27822000
O	-1.48265900	0.71798300	-0.09412500
O	-0.83484900	-1.42809800	0.27020600
O	0.30936700	0.26884100	1.54195200
B	-0.17790900	1.35150200	0.69330500
C	-0.71331900	2.62788700	1.46563400
C	-0.90685300	3.85458000	0.79707700
C	-1.04226200	2.56478200	2.83455100
C	-1.41283800	4.97658300	1.46719800
H	-0.65002600	3.92741200	-0.25968900
C	-1.54806300	3.68197000	3.51494900
H	-0.88911200	1.62914200	3.37265900
C	-1.73647900	4.89147700	2.83020500
H	-1.55233900	5.91533200	0.93218900
H	-1.79127100	3.61217700	4.57465800
H	-2.12827700	5.76193900	3.35479400
C	1.27422800	0.72322500	-1.20645800
O	0.64031500	0.27670200	-2.15749800
O	0.74414000	1.68609700	-0.38552600

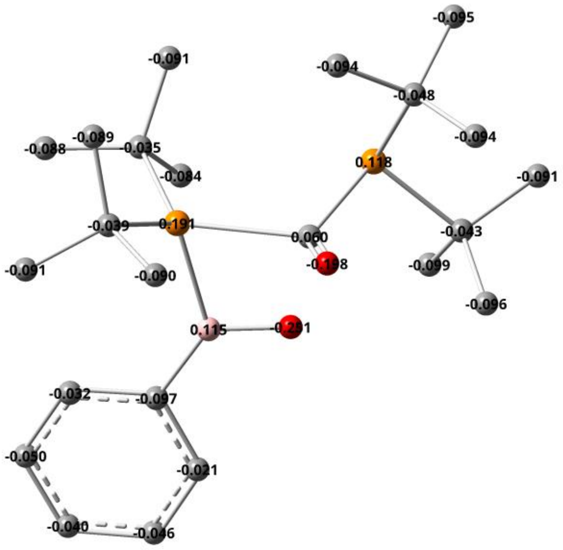

FIG. S86. OPTIMIZED STRUCTURE **1B_TS_PhBO**

B	-1.47073200	0.70133500	-0.34577400
C	-2.92819300	1.25497800	-0.24901200
P	2.22939100	0.49526300	0.51873100
C	-4.02837800	0.48452200	0.18395400
H	-3.87470200	-0.55931400	0.45389900
P	-0.70735500	-1.09302900	-0.01471600
C	-5.31503000	1.03154000	0.27519800
H	-6.14820600	0.41761800	0.61431800
C	-5.52653700	2.37309500	-0.07467800
H	-6.52510800	2.80261700	-0.00914800
C	-4.44858000	3.16117000	-0.51065400
H	-4.61245200	4.20286100	-0.78296700
C	-3.16643500	2.60716400	-0.59303600
H	-2.32660000	3.21466000	-0.92745000
C	3.68648000	-0.55081400	-0.17907700
C	4.30848500	-0.07681700	-1.50593700
H	3.55182100	-0.01877600	-2.29501700
H	5.08077600	-0.79875000	-1.81585000
H	4.79381800	0.90046600	-1.40751500
C	4.76439100	-0.60642200	0.93078000
H	5.22849800	0.36821400	1.11314000
H	5.55895800	-1.30336900	0.62357400
H	4.34095800	-0.96729600	1.87746200
C	3.15244800	-1.99199900	-0.37575400
H	2.66867800	-2.37561600	0.53073200
H	4.00117400	-2.65311500	-0.60697700
H	2.44325400	-2.04772800	-1.20787300
C	2.56878500	2.35913900	0.20698900
C	1.54259600	3.10613400	1.09608200
H	0.51769200	2.89094900	0.77984700
H	1.71763100	4.18932800	1.00555100
H	1.65919100	2.82919100	2.15257700
C	2.41901300	2.82790700	-1.25332200
H	3.11545100	2.31671400	-1.92506500
H	2.63233600	3.90772100	-1.29828600
H	1.40046000	2.66027900	-1.61410600

C	3.97869700	2.70283100	0.73819300
H	4.12363700	2.35111500	1.76873500
H	4.08982600	3.79751500	0.74093100
H	4.77663900	2.29118100	0.11059300
C	-0.78190700	-1.48615800	1.85711000
C	-0.82480400	-0.11660200	2.57607900
H	0.04582000	0.49684200	2.31638400
H	-0.80887100	-0.29073900	3.66216200
H	-1.73931700	0.43893700	2.33290800
C	-2.04534100	-2.28549700	2.23837400
H	-2.96409400	-1.77027400	1.93579600
H	-2.06731400	-2.38831800	3.33368800
H	-2.04618600	-3.29482100	1.81182700
C	0.47526000	-2.25389300	2.31653600
H	0.59190200	-3.21191700	1.79744200
H	0.38891300	-2.46022200	3.39388300
H	1.37285600	-1.64616000	2.15716300
C	-1.25601500	-2.54609500	-1.11098100
C	-2.78658200	-2.74880500	-1.05930500
H	-3.14956700	-3.00469000	-0.05921900
H	-3.04949200	-3.57744200	-1.73447700
H	-3.31344100	-1.85257800	-1.40610700
C	-0.52237600	-3.84138100	-0.70505400
H	0.56574800	-3.70542500	-0.71299700
H	-0.77067800	-4.63213300	-1.42799900
H	-0.82286800	-4.19340500	0.28855800
C	-0.87389900	-2.18401900	-2.56644400
H	-1.31495500	-1.22936000	-2.87768200
H	-1.26349600	-2.97355400	-3.22613300
H	0.20796400	-2.10976400	-2.70271400
C	0.95554300	0.07260100	-0.83004500
O	1.17923300	-0.07830600	-2.01494000
O	-0.38414100	1.41491800	-0.61392500

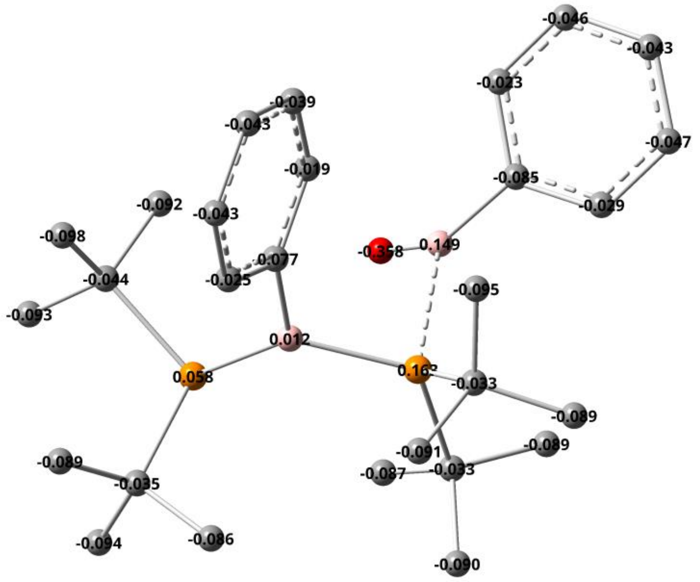

FIG. S87. OPTIMIZED STRUCTURE **1B_TS1_PhBO**

B	0.79305100	-0.03725600	0.21023900

C	0.42512400	-1.37044100	0.95091800
P	2.37408700	0.09233800	-0.88421000
C	1.10306300	-1.65926300	2.16020800
H	1.87731400	-0.97952100	2.51525400
P	-0.69180600	1.21108900	0.17560200
C	0.79666700	-2.79707000	2.91545100
H	1.32287000	-2.98397000	3.85031900
C	-0.17165200	-3.70390500	2.45708600
H	-0.40221800	-4.59734400	3.03449800
C	-0.83370000	-3.45322300	1.24671200
H	-1.58391600	-4.15107800	0.87831100
C	-0.54834600	-2.29462400	0.51394000
H	-1.08601300	-2.10763100	-0.41179600
C	2.92697000	-1.67309800	-1.39620900
C	4.21385100	-1.50145100	-2.23764900
H	4.08902500	-0.72720700	-3.00585300
H	4.44067200	-2.45217600	-2.74333500
H	5.08284300	-1.24203900	-1.61989100
C	3.15281600	-2.76790000	-0.33323200
H	3.91903100	-2.50006400	0.40018200
H	3.48942100	-3.68299700	-0.84631300
H	2.23079900	-3.00762100	0.20234700
C	1.79542900	-2.13911500	-2.34477500
H	0.85612500	-2.29258300	-1.80289900
H	2.08603100	-3.09804100	-2.80120800
H	1.60009100	-1.40894000	-3.13784100
C	3.73447000	0.94433900	0.19512900
C	3.05957600	1.96138400	1.13206700
H	2.44233600	2.66766000	0.56949000
H	3.83124000	2.53934700	1.66401400
H	2.42397900	1.46694300	1.87712000
C	4.67615100	1.71127300	-0.76159000
H	5.17729500	1.03813400	-1.46571200
H	5.45136000	2.23228800	-0.17805700
H	4.12060200	2.45648000	-1.34398500
C	4.55789100	-0.03229500	1.05471700
H	3.92211400	-0.60807500	1.73634800
H	5.27370500	0.54732400	1.65944500
H	5.13476600	-0.73465600	0.44440000
C	-1.45409900	1.36222700	1.94203300
C	-2.11162900	0.01962100	2.34431400
H	-2.80967000	-0.33636400	1.57894700
H	-2.67696200	0.18712600	3.27360400
H	-1.37516000	-0.76267500	2.53717700
C	-0.34997900	1.71215900	2.96381200
H	0.40955700	0.92282800	3.00004000
H	-0.79744100	1.79485900	3.96634900
H	0.14300300	2.66273900	2.73231900
C	-2.56376300	2.43586700	2.00259400
H	-2.21500100	3.43930500	1.74614700
H	-2.93895500	2.47085000	3.03572600
H	-3.40995000	2.18131100	1.35653900
C	-0.45586100	2.94006800	-0.62446500
C	0.02830400	4.05559200	0.32663200
H	-0.70241600	4.30745500	1.10147800
H	0.19803000	4.96290000	-0.27206000
H	0.97147600	3.80143900	0.81840300

C	-1.82435100	3.34529400	-1.22926400
H	-2.14362900	2.62711800	-1.99440800
H	-1.71812600	4.32742000	-1.71297600
H	-2.61290600	3.42889300	-0.47375500
C	0.54645500	2.81161300	-1.79342300
H	1.57273200	2.67611400	-1.43673500
H	0.50501600	3.74422900	-2.37709100
H	0.30447300	1.96610900	-2.44544100
O	-0.80363400	-0.04944900	-2.51033500
B	-1.67997100	0.03809100	-1.61254300
C	-3.13602700	-0.44461400	-1.31764600
C	-4.11812000	0.34647900	-0.69122500
C	-3.50516000	-1.74106100	-1.74579200
C	-5.41795300	-0.13185200	-0.48327700
H	-3.86199600	1.35623900	-0.37764400
C	-4.79642900	-2.23504700	-1.52206100
H	-2.76977500	-2.35914300	-2.25956400
C	-5.75640300	-1.43138700	-0.88793000
H	-6.16281900	0.50316900	-0.00576700
H	-5.05735800	-3.24094300	-1.84800200
H	-6.76191300	-1.81261100	-0.71726800

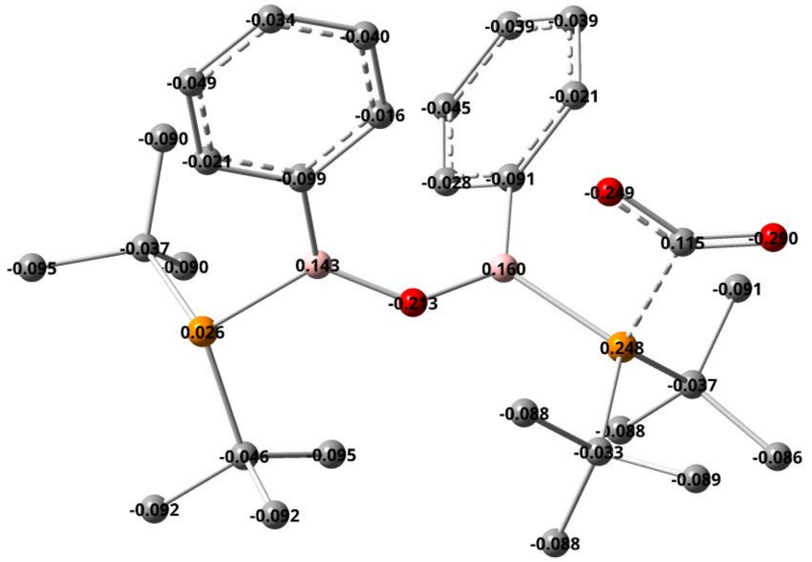

FIG. S88. OPTIMIZED STRUCTURE **1B_TS2_PhBO**

B	1.51097900	0.14776600	-0.73908400
C	1.86729800	1.52206600	-1.38490100
P	2.90504500	-1.18995800	-0.56489600
C	3.09208400	1.71050600	-2.07497800
H	3.76869000	0.86331000	-2.18636700
P	-2.70785200	-0.23363700	-0.27987300
C	3.42841400	2.94480200	-2.64001700
H	4.37213300	3.06276500	-3.17040900
C	2.53926900	4.02597700	-2.53516900
H	2.79565200	4.98759900	-2.97756200
C	1.30978600	3.85880800	-1.87978800
H	0.60562000	4.68628100	-1.81491600
C	0.97567500	2.62086900	-1.32037800
H	0.00400500	2.51068900	-0.84632600
C	2.07777400	-2.88487400	-0.28935700

C	3.16977600	-3.96094500	-0.10070200
H	3.91949100	-3.90704500	-0.90056600
H	2.70620000	-4.95878600	-0.13110100
H	3.68620300	-3.86620200	0.86163400
C	1.03860900	-2.98143900	0.84520800
H	1.48739000	-2.82565600	1.83133700
H	0.58222400	-3.98432000	0.84209000
H	0.23916300	-2.24730300	0.70752200
C	1.37676700	-3.15761400	-1.64349400
H	0.62953300	-2.38909800	-1.87298600
H	0.86278900	-4.13026400	-1.60318900
H	2.10309900	-3.18287600	-2.46503400
C	4.04299800	-0.69835800	0.91723500
C	4.07561800	0.83801000	1.05881200
H	4.38485400	1.33334400	0.13251300
H	4.80296800	1.10162800	1.84185000
H	3.09770400	1.24109600	1.34947100
C	5.46358100	-1.19800200	0.56359900
H	5.49305400	-2.28858700	0.45079900
H	6.16455600	-0.91949000	1.36564800
H	5.81327400	-0.75066900	-0.37450000
C	3.62691300	-1.29504200	2.27639700
H	2.63559900	-0.95121800	2.58698600
H	4.34926400	-0.96401800	3.03874100
H	3.63212100	-2.39018300	2.27387600
C	-3.57590200	-1.03554800	1.19754800
C	-3.83518300	0.09364800	2.22499200
H	-2.90274200	0.53309900	2.59240000
H	-4.36704300	-0.34230400	3.08300300
H	-4.45649600	0.88691700	1.79455100
C	-4.93368700	-1.64544300	0.79418800
H	-5.57663300	-0.89999000	0.31215200
H	-5.43883800	-1.99094900	1.70742900
H	-4.82282700	-2.50995100	0.13015400
C	-2.65507100	-2.10252100	1.82897000
H	-2.43024000	-2.92274400	1.13830100
H	-3.15689800	-2.52799600	2.71034100
H	-1.70941900	-1.65939500	2.16582200
C	-2.82556100	-1.21092200	-1.89669700
C	-4.25688000	-1.09945900	-2.47328900
H	-5.00515400	-1.58689800	-1.84210000
H	-4.26698200	-1.59727600	-3.45340100
H	-4.55273500	-0.05421700	-2.61364200
C	-2.44228500	-2.68881500	-1.67482600
H	-1.45702100	-2.78677700	-1.20693800
H	-2.40426900	-3.19398200	-2.65067700
H	-3.17862700	-3.21492800	-1.05627000
C	-1.84689300	-0.55900700	-2.90441700
H	-2.03773100	0.51357000	-3.01903700
H	-1.99252300	-1.04741000	-3.87834000
H	-0.80484900	-0.69060200	-2.60291900
O	0.16622900	-0.16312400	-0.52530400
B	-0.88784400	0.35926900	0.16062500
C	-0.61918900	1.19570800	1.46312800
C	0.32817800	0.64740300	2.35796200
C	-1.23181300	2.41807400	1.81834400
C	0.64629100	1.27801000	3.56700600

H	0.81476700	-0.29213800	2.09933800
C	-0.90251200	3.06187100	3.01719700
H	-1.94210800	2.87612400	1.13526900
C	0.03001200	2.49266600	3.89835400
H	1.37617800	0.82981500	4.23964500
H	-1.37218900	4.01287800	3.26269400
H	0.27897300	2.99573900	4.83122200
C	-3.31330200	1.56188100	-0.72522800
O	-4.50349700	1.87361800	-0.64351100
O	-2.25127400	2.15741900	-1.07729500

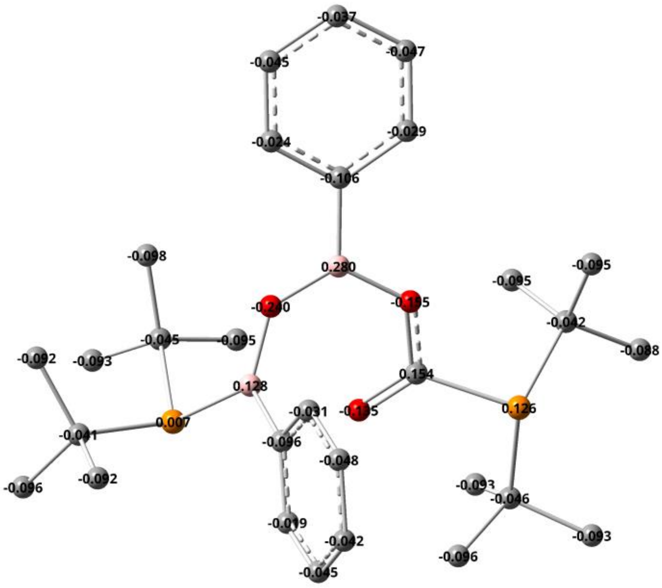

FIG. S89. OPTIMIZED STRUCTURE **1B_TS3_PhBO**

B	-1.48233500	-0.31829900	0.42591200
C	-1.17958900	-1.17623700	1.70702100
P	-2.60671900	-1.02125800	-1.00321200
C	-1.11814000	-2.58755600	1.65124600
H	-1.30854800	-3.08219300	0.69917800
P	3.20925500	-0.94559100	0.25640000
C	-0.79638400	-3.34662700	2.78133800
H	-0.74333300	-4.43231500	2.71166500
C	-0.53698400	-2.70796200	4.00436400
H	-0.28819200	-3.29648400	4.88622300
C	-0.59494400	-1.30873900	4.08613200
H	-0.39406900	-0.80882700	5.03262800
C	-0.90653900	-0.55549800	2.94676800
H	-0.94884900	0.53143700	3.02098400
C	-2.54903900	0.27237600	-2.42533400
C	-3.55235600	-0.15009400	-3.52113400
H	-3.43919400	-1.20941000	-3.78623200
H	-3.37571800	0.45242100	-4.42560800
H	-4.59012200	0.02215000	-3.21019200
C	-2.78721800	1.75811300	-2.07704400
H	-3.80848000	1.94524600	-1.73269000
H	-2.62764700	2.36370200	-2.98379600
H	-2.09607900	2.10978500	-1.30751500
C	-1.12104500	0.13731700	-3.00907900
H	-0.36204400	0.47471400	-2.29542100
H	-1.04107300	0.75973000	-3.91377300

H	-0.89300300	-0.90181200	-3.27786900
C	-4.38495100	-1.08415800	-0.24813800
C	-4.31276800	-1.71256800	1.16034600
H	-3.77420000	-2.66743400	1.15711700
H	-5.33917300	-1.90347300	1.50962700
H	-3.82352100	-1.05188000	1.88456300
C	-5.22994900	-2.03324100	-1.13150600
H	-5.34029600	-1.66207700	-2.15566500
H	-6.23778300	-2.13362900	-0.69882300
H	-4.77288700	-3.02929700	-1.17856500
C	-5.09203100	0.27984800	-0.13102500
H	-4.48985800	0.99973600	0.43704300
H	-6.05209700	0.14635000	0.39280400
H	-5.31325400	0.71015400	-1.11420800
C	3.16368700	-2.58884200	-0.71215200
C	2.11094000	-3.46994800	0.00931000
H	2.30602100	-3.52347600	1.08854100
H	2.17660000	-4.48945500	-0.39913300
H	1.09649500	-3.09751000	-0.14408900
C	2.82000500	-2.47390200	-2.20882300
H	1.85188400	-1.98391400	-2.35759500
H	2.76142400	-3.48297600	-2.64427300
H	3.59067600	-1.92140500	-2.76042200
C	4.53516300	-3.28368200	-0.52526100
H	5.34087700	-2.79389400	-1.07953800
H	4.45487400	-4.31520200	-0.89767200
H	4.81872900	-3.32928500	0.53458000
C	4.23049600	0.40514000	-0.66509600
C	3.51453800	1.04354100	-1.87381300
H	3.23328400	0.29582300	-2.62349700
H	4.19427600	1.76475000	-2.35263900
H	2.61456300	1.58789100	-1.56913500
C	5.57696000	-0.18751700	-1.13073800
H	6.09910200	-0.71136500	-0.32019600
H	6.22045100	0.63926500	-1.46586400
H	5.45363300	-0.87401600	-1.97578100
C	4.52588400	1.47996100	0.40764700
H	3.60424400	1.86784600	0.85228900
H	5.06966100	2.31620200	-0.05735800
H	5.14610900	1.06865000	1.21377700
O	-1.08850500	1.02754300	0.46122400
B	0.10280900	1.66842500	0.53416500
C	0.14253300	3.21284200	0.72045500
C	-1.06548400	3.91628300	0.93434500
C	1.34238800	3.95590800	0.66585700
C	-1.07488400	5.30632900	1.09370400
H	-1.99981400	3.35803900	0.97288400
C	1.33969600	5.34734200	0.82101000
H	2.28312500	3.43720900	0.49518700
C	0.12992100	6.02449900	1.03686900
H	-2.01457100	5.83085900	1.25890200
H	2.27467100	5.90336000	0.77363900
H	0.12554900	7.10657000	1.15867800
C	1.49314300	-0.29553200	-0.03470500
O	0.55018700	-0.93011100	-0.50755100
O	1.34817400	0.98971200	0.42803400

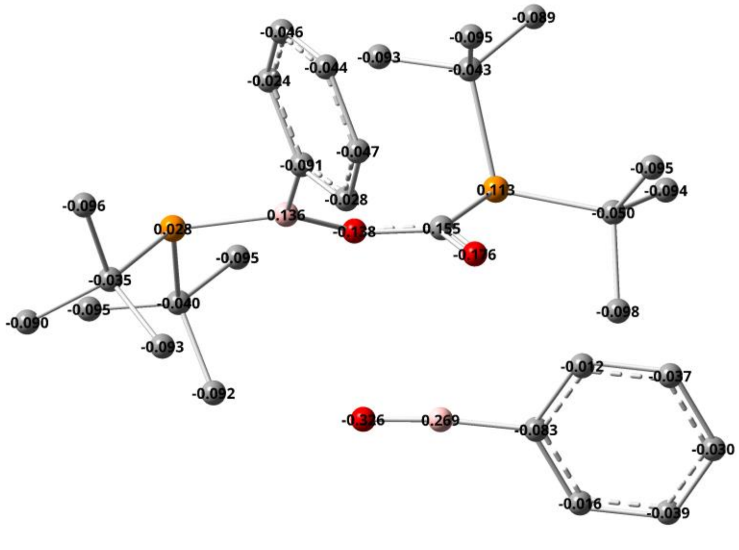

FIG. S90. OPTIMIZED STRUCTURE **1B_TS4_PhBO**

B	-1.62067600	0.13072700	0.26560900
C	-1.49233700	-0.13755800	1.80833500
P	1.60092200	2.15530100	-0.88921100
C	-2.15765200	0.73737600	2.70064000
H	-2.75227700	1.55616900	2.29631900
P	-3.34023400	0.26727700	-0.55659200
C	-2.06540300	0.57075400	4.08903400
H	-2.57817300	1.26437500	4.75371900
C	-1.32522500	-0.49568300	4.61803500
H	-1.25972700	-0.63527100	5.69595000
C	-0.67462800	-1.38657700	3.75064500
H	-0.10694800	-2.22356300	4.15530500
C	-0.74870700	-1.20278900	2.36475200
H	-0.23968200	-1.89559600	1.70031200
C	0.96935100	3.39542500	0.44627200
C	0.91163400	2.83220500	1.88007000
H	0.22228800	1.98453900	1.96075400
H	0.54688000	3.61824300	2.55919000
H	1.89385100	2.50700600	2.23628700
C	1.87032800	4.64810600	0.41520400
H	2.86353800	4.44686100	0.83274700
H	1.40367100	5.43150700	1.03055800
H	1.98809600	5.04335400	-0.60215700
C	-0.45262000	3.80682700	-0.00274300
H	-0.44311100	4.23930000	-1.01084900
H	-0.84075000	4.56254100	0.69664800
H	-1.14578500	2.95958600	-0.00264500
C	3.45641500	1.77696000	-0.65501500
C	3.71131200	0.50078400	-1.49719600
H	3.25228600	-0.38105300	-1.04378700
H	4.79596000	0.32709800	-1.55690500
H	3.33091600	0.61560100	-2.52157200
C	3.95627200	1.55547500	0.78424600
H	3.89639000	2.47481900	1.37955700
H	5.01387100	1.25062700	0.75370900
H	3.38623100	0.76559000	1.28389200

C	4.24348300	2.93232700	-1.32071300
H	3.89820600	3.10971200	-2.34781000
H	5.30780600	2.65722700	-1.36245000
H	4.16440400	3.87187800	-0.76422100
C	-3.09359000	0.39793500	-2.45320700
C	-2.25627600	1.67912800	-2.69689700
H	-2.71452000	2.54930800	-2.20763100
H	-2.23156200	1.87560100	-3.77951500
H	-1.22778900	1.58092200	-2.34425300
C	-2.38600100	-0.82038400	-3.07683800
H	-1.42427800	-1.02098000	-2.59273200
H	-2.20743700	-0.63290800	-4.14776700
H	-3.00095900	-1.72534600	-2.99804500
C	-4.45899200	0.63730100	-3.14245800
H	-5.10755900	-0.24222500	-3.12295200
H	-4.27800200	0.89247300	-4.19766600
H	-4.99554700	1.47455500	-2.67745700
C	-4.23060300	-1.36706500	-0.04329900
C	-3.30424700	-2.60048200	-0.04004300
H	-2.82979100	-2.76643800	-1.01136100
H	-3.89427000	-3.49563000	0.21543200
H	-2.50883700	-2.49943100	0.70530100
C	-5.42710600	-1.64197100	-0.97703800
H	-6.09142100	-0.77315600	-1.06430000
H	-6.00979000	-2.47505700	-0.55576700
H	-5.10167600	-1.94378600	-1.97904900
C	-4.78647200	-1.13828600	1.38143800
H	-3.98829200	-0.97039100	2.11098700
H	-5.34325600	-2.03620100	1.69122100
H	-5.46930200	-0.27960600	1.40974100
C	0.81664000	0.61473300	-0.16697300
O	1.36689200	-0.31901400	0.39821800
O	-0.52355600	0.59985400	-0.47663300
O	-0.05266700	-2.78334500	-0.98132100
B	1.14143600	-2.82881200	-0.71273800
C	2.63494300	-3.00430600	-0.46082600
C	3.21979500	-2.65508600	0.77779800
C	3.46086200	-3.51201300	-1.49048900
C	4.59530400	-2.80788500	0.97719900
H	2.59824200	-2.23953300	1.56617700
C	4.83822700	-3.65716800	-1.28787500
H	3.02449300	-3.78384100	-2.44987200
C	5.40590100	-3.30459500	-0.05487700
H	5.03671100	-2.53078100	1.93260100
H	5.46616700	-4.04184700	-2.08915300
H	6.47761400	-3.41538500	0.10101500

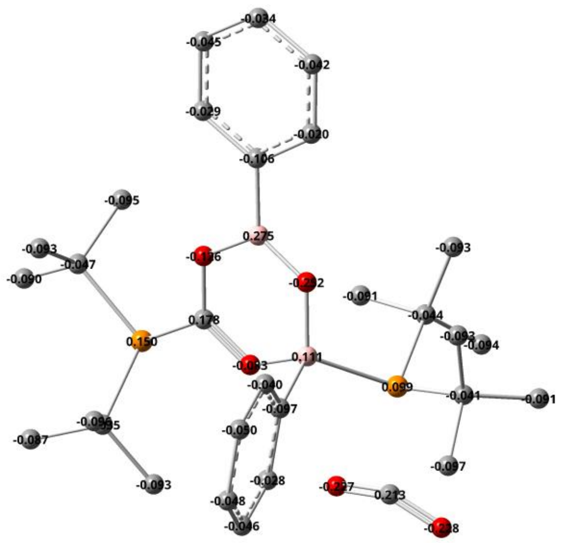

FIG. S91. OPTIMIZED STRUCTURE **1B_TS5_PhBO**

B	0.94987500	0.07785200	0.44973300
C	1.20754800	-0.60578000	1.87158500
P	-2.59830600	-1.68248500	-1.04544400
C	1.53230000	-1.97778800	1.96864300
H	1.64009800	-2.56505500	1.05748400
P	2.47891800	-0.10570300	-0.82765900
C	1.72869300	-2.59438700	3.20977800
H	1.98216000	-3.65263800	3.25718400
C	1.60793000	-1.84594400	4.39159200
H	1.76621300	-2.32059000	5.35881000
C	1.28516200	-0.48343200	4.31924200
H	1.19062400	0.10370900	5.23197700
C	1.08405900	0.12457200	3.07055500
H	0.83308100	1.18423400	3.02324800
C	-2.35596200	-3.08408200	0.26058100
C	-2.14764300	-2.58152400	1.70368800
H	-1.22057000	-2.00925800	1.81046900
H	-2.06611800	-3.45307300	2.36980500
H	-2.98798500	-1.97040700	2.05148800
C	-3.58266000	-4.01864500	0.21415400
H	-4.46828200	-3.55891200	0.66807200
H	-3.34289700	-4.92026800	0.79563600
H	-3.82635600	-4.33480200	-0.80798700
C	-1.11452800	-3.87035900	-0.22059600
H	-1.29068900	-4.32247000	-1.20423200
H	-0.91186200	-4.67529500	0.50128100
H	-0.22396200	-3.24134000	-0.29347900
C	-4.29455900	-0.81992200	-0.91476700
C	-4.21720800	0.39860700	-1.87166900
H	-3.51520900	1.16061800	-1.52544400
H	-5.21628900	0.85414800	-1.93475200
H	-3.92425300	0.08678400	-2.88256500
C	-4.73676500	-0.38575000	0.49449100
H	-4.87855800	-1.25090300	1.15295100
H	-5.70169500	0.13870200	0.42445300
H	-4.01295300	0.29278700	0.95761600

C	-5.34159500	-1.78879100	-1.52162900
H	-5.01954900	-2.16709400	-2.50063600
H	-6.28094700	-1.23624800	-1.66589200
H	-5.55484600	-2.64189100	-0.87195800
C	2.04621500	0.88868000	-2.40587600
C	0.60502800	0.49757800	-2.81129000
H	0.45729800	-0.58874700	-2.81205200
H	0.40867100	0.87335400	-3.82649400
H	-0.13541500	0.96124000	-2.14883400
C	2.10681500	2.42138500	-2.23914800
H	1.47625200	2.75920200	-1.40940600
H	1.74376000	2.89775100	-3.16358500
H	3.12893500	2.77619800	-2.06618400
C	2.98011700	0.44458100	-3.55676400
H	4.03377400	0.64835600	-3.34596900
H	2.70512000	0.99176000	-4.47174500
H	2.87408900	-0.62796300	-3.75872900
C	3.99113200	0.65753100	0.05536700
C	3.65953900	1.95402500	0.82543800
H	3.29298100	2.74750000	0.16606000
H	4.57245900	2.31799500	1.32288100
H	2.90384000	1.77252800	1.59753100
C	5.12847100	0.91483100	-0.95673200
H	5.36738600	0.00705900	-1.52353600
H	6.02915500	1.21708800	-0.40132200
H	4.88953700	1.72191300	-1.65879800
C	4.49970400	-0.40269500	1.06398200
H	3.77220400	-0.59248200	1.85851200
H	5.42045500	-0.02186800	1.53215800
H	4.73222300	-1.34823300	0.56180800
C	-1.42086900	-0.46579000	-0.32512600
O	-1.81509500	0.77278300	-0.04381200
O	-0.21043100	-0.82983600	-0.19907100
O	0.42563200	1.44607400	0.55425200
B	-0.84889000	1.78925400	0.36042200
C	-1.39739100	3.22811300	0.52015500
C	-2.76671500	3.54498500	0.37492100
C	-0.49558000	4.27429000	0.82449400
C	-3.22125600	4.86037300	0.52690200
H	-3.47897200	2.75420600	0.14573000
C	-0.94462100	5.59050200	0.97334600
H	0.56154000	4.04004900	0.94150100
C	-2.30957500	5.88460100	0.82451700
H	-4.27969100	5.08883400	0.41398400
H	-0.23828500	6.38585900	1.20475400
H	-2.66122600	6.90843500	0.94096500
C	2.91362000	-2.41090700	-1.63140800
O	4.11173200	-2.53150300	-1.59359700
O	1.80868900	-2.83209500	-1.87496700

Formation of 1d

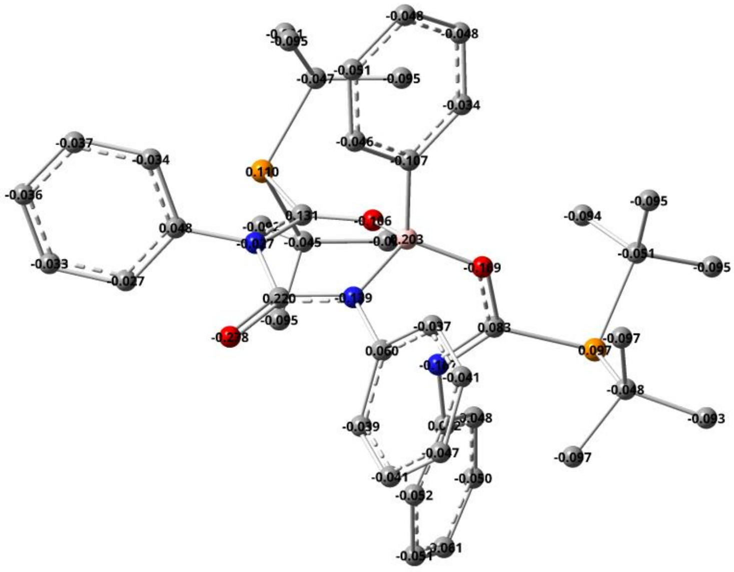

FIG. S92. OPTIMIZED STRUCTURE **1D**

P	3.65317800	0.71837000	0.94533900
P	-3.25271100	1.48422400	0.76185200
O	1.36159500	-0.81731600	0.64706900
O	-0.88399800	0.06825500	0.60427400
O	-1.61914500	-1.26561500	-3.11877900
N	-2.33232300	-0.41572500	-1.08509300
N	-0.16445500	-1.37237400	-1.29756700
N	1.42401400	0.98855300	-0.75805700
C	-2.02523400	0.25073100	0.04712200
C	-0.58592300	-2.37721400	1.12003200
C	-5.32576700	0.45064000	-3.16150500
H	-5.59818100	1.10961600	-3.98323300
C	2.31675900	-2.03357900	-4.02310900
H	2.60685700	-1.61927500	-4.98708400
C	3.32888100	0.40284200	2.80505700
C	-4.01206500	0.45180800	-2.68049400
H	-3.25091100	1.09141900	-3.11734300
C	2.98975400	-3.15115000	-3.50569400
H	3.80257100	-3.60895200	-4.06621000
C	2.82701200	4.61337000	-2.42554000
H	3.16338800	5.54785600	-2.87051800
C	-3.68129700	-0.39492900	-1.61836100
C	1.56300700	-3.08430800	-1.53878200
H	1.25925900	-3.49064200	-0.57701000
C	-4.61814100	-1.26982800	-1.05996800
H	-4.31368400	-1.93869100	-0.25848200
C	1.27379900	-1.43847600	-3.30613100
H	0.74457700	-0.57982700	-3.70615100
C	-1.44138000	-3.35207000	0.56483000
H	-1.61582500	-3.36035000	-0.51155900
C	-5.93204200	-1.26189300	-1.54556800
H	-6.67197500	-1.93079900	-1.11082400
C	-0.36729700	-2.42429700	2.51384500
H	0.28859100	-1.68604900	2.97222700
C	-6.28701600	-0.39837200	-2.59112900

H	-7.30772900	-0.39283000	-2.96831400
C	-1.31144000	-1.07462000	-1.94452200
C	-0.93372500	3.19624800	1.45705100
H	-1.21487100	3.36395700	2.50205900
H	-0.35758300	4.07089800	1.12183000
H	-0.28487600	2.31786400	1.39657300
C	6.18217600	-0.38648000	0.59231700
H	6.48020800	0.64655000	0.37135700
H	6.85524100	-1.06455300	0.04605200
H	6.32807200	-0.56808700	1.66509400
C	1.95595600	2.18728900	-1.26914400
C	0.89361800	-1.96714700	-2.06108700
C	2.20254100	2.27275500	-2.65451100
H	2.05690600	1.38496400	-3.26521500
C	2.64787200	3.47200000	-3.22221100
H	2.84890100	3.51601700	-4.29166200
C	2.13490500	3.33735700	-0.47268100
H	1.93750000	3.27644800	0.59387900
C	4.49078000	1.09247800	3.56012500
H	5.45217200	0.60269900	3.36140600
H	4.30561400	1.03898400	4.64396300
H	4.58118800	2.14859000	3.27568000
C	-2.02620400	0.81113600	3.41389800
H	-1.21931500	0.28542300	2.89972600
H	-2.24424800	0.26888400	4.34657300
H	-1.67893400	1.81205100	3.68576500
C	1.98222900	0.29793900	0.17654500
C	-4.36431900	1.74152000	3.27694900
H	-3.99804700	2.76644900	3.41704700
H	-4.57624200	1.32475800	4.27247900
H	-5.30599700	1.78271600	2.71421700
B	0.00171300	-1.17230000	0.23562200
C	-1.83181400	-4.34714900	2.74566900
H	-2.30933800	-5.10166400	3.36905400
C	-3.31342300	0.85416000	2.57022300
C	-0.97591600	-3.39445400	3.32023100
H	-0.78711600	-3.40799400	4.39314800
C	2.56395400	4.53755000	-1.04920500
H	2.69985500	5.41510600	-0.41839800
C	-1.71882400	3.05150100	-0.92764900
H	-0.95845100	2.28435900	-1.11816100
H	-1.26667700	4.02433600	-1.16635400
H	-2.56871500	2.89573000	-1.60551800
C	-2.06083800	-4.32633100	1.36265100
H	-2.71352700	-5.06947300	0.90590800
C	3.20275700	-1.05318700	3.28808800
H	2.50397500	-1.62574000	2.67252400
H	2.84058200	-1.05712800	4.32877500
H	4.17532000	-1.55843100	3.27971200
C	-3.87334700	-0.58451500	2.43936200
H	-4.78962300	-0.60644800	1.83534400
H	-4.11316300	-0.96863000	3.44131800
H	-3.13622000	-1.26721100	1.99603200
C	2.02364700	1.16893300	3.12514400
H	2.07020200	2.20838400	2.77190800
H	1.87148600	1.19131000	4.21486300
H	1.15320500	0.68591300	2.66841300

C	4.72372400	-0.66048400	0.16233700
C	4.36030600	-2.12599700	0.46352000
H	4.65605600	-2.41885600	1.47559000
H	4.89841700	-2.77586700	-0.24348800
H	3.28938200	-2.30823600	0.34327300
C	2.60980500	-3.67096300	-2.26201300
H	3.12520600	-4.53547100	-1.84782500
C	4.59073300	-0.40984800	-1.35988300
H	3.59428700	-0.68393900	-1.72525200
H	5.32463800	-1.03239200	-1.89237800
H	4.77660500	0.64165500	-1.61586200
C	-2.16858300	3.05506800	0.55376200
C	-3.12756900	4.24619900	0.78452300
H	-4.00037100	4.19353600	0.12162300
H	-2.59074600	5.18324100	0.57597300
H	-3.48402800	4.28924300	1.82142100

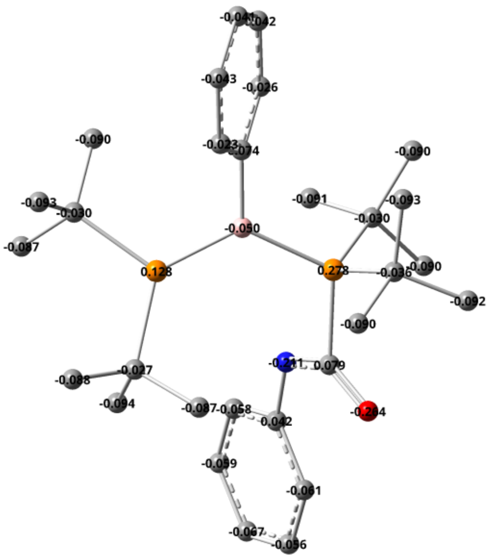

FIG. S93. OPTIMIZED STRUCTURE **1D_I1_A**

B	1.21252000	0.04587000	0.00221500
C	2.75181500	-0.15659200	-0.31150500
P	0.06122100	-1.53737800	0.19849200
C	3.31608200	-0.71355000	-1.48181500
H	2.67200600	-0.99951800	-2.30850700
P	0.70159800	1.77530800	0.23508000
C	4.69995100	-0.85615000	-1.63671000
H	5.09761900	-1.27369600	-2.56050100
C	5.57303600	-0.45508300	-0.61426600
H	6.64887900	-0.56882200	-0.73419100
C	5.04489000	0.10052800	0.55882700
H	5.70839900	0.42232600	1.36009700
C	3.65902600	0.25149900	0.70025900
H	3.25738200	0.68439900	1.61561700
C	0.74413900	-2.62790300	1.59201700
C	-0.08631500	-3.92483900	1.72257000
H	-1.15457000	-3.70513600	1.81255600
H	0.24382900	-4.44967000	2.63106100
H	0.08470300	-4.59842100	0.87399500
C	2.23650500	-2.98967000	1.42912400

H	2.42957100	-3.60376900	0.54553800
H	2.53170400	-3.57457000	2.31283900
H	2.87634400	-2.10448800	1.38211000
C	0.58616200	-1.77582200	2.87508900
H	1.17165000	-0.84873800	2.80491700
H	0.97046300	-2.35547000	3.72682500
H	-0.46471800	-1.53177500	3.05963100
C	-0.04373500	-2.45736500	-1.47860300
C	-0.13267300	-1.37826900	-2.58278800
H	-0.98400100	-0.71817000	-2.38522200
H	-0.27136200	-1.88022800	-3.55159100
H	0.77266400	-0.76282200	-2.64162800
C	-1.34530100	-3.29435400	-1.51832400
H	-1.39661200	-4.02464400	-0.70420300
H	-1.35868000	-3.84512700	-2.47022200
H	-2.23156200	-2.65510600	-1.47661700
C	1.15019700	-3.40480200	-1.72207200
H	2.12065300	-2.91945900	-1.58962900
H	1.08900100	-3.76948500	-2.75792100
H	1.10546100	-4.27871800	-1.06215700
C	1.75547100	3.13272700	-0.60451300
C	2.52978700	2.54128600	-1.80529900
H	3.38103900	1.93517900	-1.48967900
H	2.91532400	3.38217100	-2.40049400
H	1.88269800	1.93259800	-2.44810500
C	0.78438400	4.19107800	-1.18675100
H	0.08799800	3.73647600	-1.90167200
H	1.38123800	4.94496600	-1.72026800
H	0.20316700	4.71293200	-0.42171300
C	2.77190500	3.79407200	0.35310300
H	2.29012200	4.28219300	1.20515200
H	3.33691700	4.55911700	-0.20069900
H	3.48388700	3.05179100	0.73059800
C	-0.68648600	2.47780400	1.33673700
C	-1.93238400	2.87287400	0.51769500
H	-1.72514200	3.69776200	-0.17218800
H	-2.72265100	3.19931700	1.20945800
H	-2.30595800	2.01978700	-0.05227900
C	-0.14367000	3.69441700	2.12551600
H	0.73196900	3.42554000	2.72933900
H	-0.93944000	4.02226600	2.80986000
H	0.11268700	4.54800500	1.49126000
C	-1.06042900	1.41195400	2.38606900
H	-1.50154700	0.52084000	1.94201600
H	-1.81287000	1.84947400	3.05818100
H	-0.19026300	1.12245100	2.98610900
C	-1.79619800	-1.20055600	0.59430000
O	-2.30059000	-1.76450700	1.59498000
N	-2.24160700	-0.42430300	-0.37837600
C	-3.60352400	-0.09026500	-0.48691600
C	-4.61988600	-0.39262800	0.45566300
C	-3.96422500	0.63776700	-1.64813600
C	-5.93659400	0.02979300	0.22877300
H	-4.36045300	-0.95431000	1.34724400
C	-5.28106000	1.05100300	-1.86524200
H	-3.18043100	0.86971100	-2.36825500
C	-6.28012700	0.75112800	-0.92450400

H	-6.70366600	-0.21078100	0.96463700
H	-5.52961400	1.60839600	-2.76786500
H	-7.30721000	1.07261500	-1.08911200

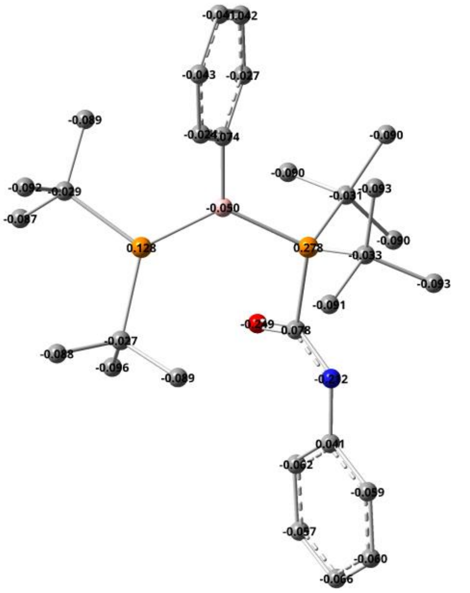

FIG. S94. OPTIMIZED STRUCTURE **1D_I1_B**

B	-1.33529400	-0.08729200	0.01172000
C	-2.77911400	-0.69110700	-0.22619500
P	0.20226200	-1.30240000	0.16658500
C	-3.50631100	-1.51752400	0.66121400
H	-3.09569500	-1.75303000	1.63890300
P	-1.26726100	1.73088500	-0.04356400
C	-4.78058300	-2.00020200	0.34218800
H	-5.31650200	-2.61948400	1.05995400
C	-5.37193400	-1.68119900	-0.88972500
H	-6.36184400	-2.05934100	-1.13843000
C	-4.67645600	-0.86568000	-1.79231900
H	-5.12237500	-0.60444300	-2.75082200
C	-3.40590100	-0.37666100	-1.46023100
H	-2.87212900	0.25570900	-2.16823200
C	0.32250200	-2.36766800	-1.40350300
C	1.48730800	-3.37395500	-1.28630400
H	2.41492900	-2.86026600	-1.01316700
H	1.62011700	-3.85884400	-2.26467100
H	1.26826500	-4.16178500	-0.55516700
C	-0.98603400	-3.11938800	-1.73385200
H	-1.24334000	-3.87201100	-0.98378800
H	-0.83220300	-3.64124600	-2.68999200
H	-1.83597300	-2.44181000	-1.85317500
C	0.61336400	-1.36432300	-2.54516600
H	-0.19522500	-0.62631900	-2.63928000
H	0.67545200	-1.92287700	-3.49045400
H	1.56163700	-0.84606200	-2.37053700
C	0.02535500	-2.33676300	1.76789400
C	-0.55307800	-1.40533500	2.85950600
H	0.09058200	-0.52920700	2.98912600
H	-0.60050900	-1.96578500	3.80495400

H	-1.56388900	-1.05563600	2.61867300
C	1.43314100	-2.79718400	2.22003800
H	1.94531900	-3.38452100	1.45097600
H	1.30784100	-3.43351200	3.10837800
H	2.06395000	-1.94579600	2.49161300
C	-0.85889000	-3.58766400	1.58359000
H	-1.83576000	-3.36551300	1.14618600
H	-1.02212600	-4.03680100	2.57417100
H	-0.36113600	-4.33848400	0.95928500
C	-2.81454900	2.69229100	0.53740100
C	-3.77057200	1.76811100	1.32477300
H	-4.30113300	1.06905200	0.67508000
H	-4.51844200	2.40738300	1.81692900
H	-3.24188800	1.19886400	2.09827200
C	-2.34387500	3.78668600	1.52856000
H	-1.82815000	3.34285300	2.38852900
H	-3.23277100	4.31938500	1.89695200
H	-1.68132200	4.52723700	1.07277100
C	-3.59960500	3.31593200	-0.63837100
H	-3.00131800	4.03100000	-1.21037400
H	-4.47537100	3.84966200	-0.23945700
H	-3.95671000	2.53445000	-1.31891000
C	0.19666000	2.85453500	-0.51633500
C	1.02816000	3.30382000	0.70419300
H	0.44002300	3.92794300	1.38630500
H	1.87887300	3.90210600	0.34477600
H	1.41282700	2.43701900	1.24966100
C	-0.33456700	4.08501700	-1.29183900
H	-0.91772400	3.78648100	-2.17187300
H	0.53982000	4.65187300	-1.64241700
H	-0.93803300	4.76196300	-0.67967600
C	1.07563700	2.06660700	-1.51122400
H	1.50170100	1.15808200	-1.08467700
H	1.91525000	2.71129000	-1.80852300
H	0.50744000	1.80128600	-2.41105800
C	1.81551900	-0.36572100	0.57431100
O	1.70467200	0.34371700	1.61252300
N	2.78013900	-0.64798500	-0.27575100
C	4.06300700	-0.08560300	-0.15002300
C	4.50749900	0.77122400	0.89002400
C	4.97821500	-0.41853600	-1.17845800
C	5.81789200	1.26733800	0.88078300
H	3.81954300	1.03238600	1.68747200
C	6.28257400	0.08238700	-1.17802900
H	4.63305000	-1.07777800	-1.97358200
C	6.71332100	0.93245600	-0.14597400
H	6.14171700	1.92364100	1.68845000
H	6.96515100	-0.18960700	-1.98249000
H	7.72906900	1.32449200	-0.14209400

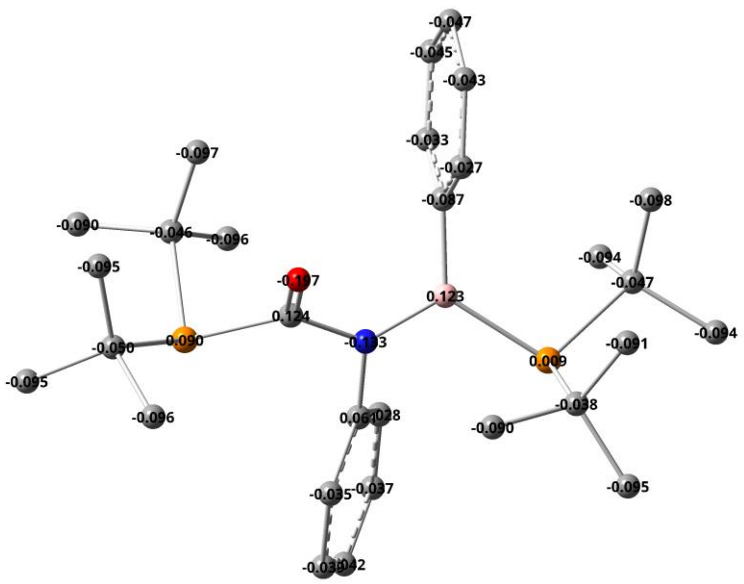

FIG. S95. OPTIMIZED STRUCTURE **1D_I2_A**

B	1.06670200	0.23905000	0.04906600
C	1.06053300	1.80713100	0.12252000
P	-3.04971900	-0.41324100	-0.16968300
C	1.34453100	2.54560500	1.29140100
H	1.47939000	2.02213100	2.23574300
P	2.66476700	-0.87659600	-0.14325500
C	1.42519100	3.94139700	1.26784500
H	1.63300100	4.48573000	2.18789300
C	1.23664700	4.64216200	0.06532500
H	1.30897800	5.72816400	0.04594800
C	0.94527300	3.93227700	-1.10578400
H	0.78375400	4.46227400	-2.04339900
C	0.85102600	2.53299800	-1.07159100
H	0.61606000	2.00067000	-1.99326400
C	-4.19857600	-0.20198600	1.34280200
C	-4.40342600	1.21529300	1.90764400
H	-3.44779800	1.68185600	2.16499200
H	-5.01951200	1.15102100	2.81833900
H	-4.93852300	1.85736900	1.19717900
C	-5.56440500	-0.80044600	0.92351400
H	-6.05893300	-0.20270600	0.14955300
H	-6.22866300	-0.82813100	1.80034800
H	-5.45180700	-1.82468600	0.54494000
C	-3.60547000	-1.11797200	2.44234000
H	-3.42341600	-2.13209100	2.05977100
H	-4.32694800	-1.19744900	3.26900900
H	-2.67055300	-0.71315900	2.84378500
C	-3.21133900	1.09056000	-1.35931000
C	-2.31428700	0.72000600	-2.56418200
H	-1.26041300	0.65725800	-2.26898500
H	-2.40235100	1.50005900	-3.33548100
H	-2.60576000	-0.24214900	-3.00311200
C	-2.78165800	2.46490100	-0.81307000
H	-3.39378600	2.78259100	0.03521800
H	-2.89653700	3.21314200	-1.61309200
H	-1.73398300	2.47373800	-0.49790000
C	-4.67787800	1.15132100	-1.83839000

H	-5.03234400	0.17358400	-2.19066500
H	-4.75033400	1.86218700	-2.67526300
H	-5.34902300	1.50316800	-1.04598600
C	3.07465100	-1.21536100	1.71829900
C	1.73925400	-1.40962300	2.46944600
H	1.16367800	-0.47666900	2.54180400
H	1.94823200	-1.75706700	3.49300500
H	1.11251300	-2.16384900	1.97948500
C	3.85804700	-2.54708600	1.76650300
H	3.27448400	-3.36022600	1.31819400
H	4.07765100	-2.80867500	2.81384100
H	4.81102300	-2.47717900	1.22883500
C	3.88830600	-0.11678600	2.42508100
H	4.89291000	-0.01643900	1.99859800
H	4.00615800	-0.38669900	3.48681200
H	3.39225600	0.85822100	2.37338400
C	4.03135700	0.19152700	-0.95867900
C	5.32755700	-0.65060400	-0.95029200
H	5.75598500	-0.72460400	0.05725300
H	6.08020700	-0.17227700	-1.59560700
H	5.14769100	-1.66713900	-1.32432400
C	4.34094700	1.60592700	-0.42650500
H	3.46873200	2.26280500	-0.47438600
H	5.13206800	2.04920600	-1.05254900
H	4.70535700	1.59312800	0.60431000
C	3.54340600	0.31685400	-2.42368200
H	3.34854000	-0.66876100	-2.86651600
H	4.31206100	0.82394200	-3.02660300
H	2.62560300	0.91622000	-2.48637800
C	-1.38023200	0.08992000	0.59640200
O	-1.26188400	0.84861400	1.55474700
N	-0.20454400	-0.47674300	-0.00460500
C	-0.33429500	-1.80092500	-0.56848700
C	0.01012300	-2.02840700	-1.90792100
C	-0.80880800	-2.85592800	0.22495700
C	-0.11924200	-3.31024400	-2.45231100
H	0.37970400	-1.20251000	-2.51110100
C	-0.94140400	-4.13670500	-0.32369400
H	-1.07285300	-2.66759100	1.26342300
C	-0.59685400	-4.36736700	-1.66315300
H	0.15317400	-3.48340900	-3.49163700
H	-1.31291300	-4.95160200	0.29470800
H	-0.69894700	-5.36344500	-2.08927400

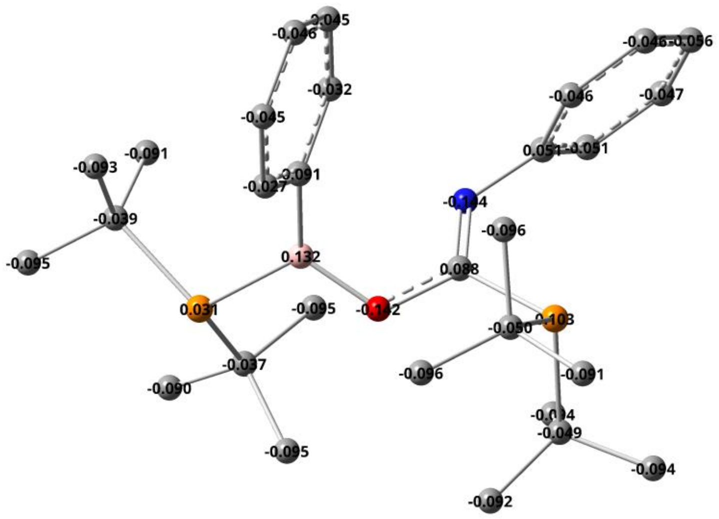

FIG. S96. OPTIMIZED STRUCTURE **1D_I2_B**

B	-1.34496700	0.11162500	0.37213900
C	-1.15648900	1.63307500	0.73367600
P	2.62570900	-0.72196400	0.58391800
C	-0.82518400	2.64183600	-0.19776100
H	-0.69976500	2.37749900	-1.24494100
P	-3.01464800	-0.81037900	0.36230800
C	-0.64591900	3.97130900	0.20760000
H	-0.38622200	4.72832500	-0.53099200
C	-0.79237800	4.32556200	1.55692800
H	-0.64880200	5.35791800	1.87161900
C	-1.12799500	3.34118100	2.49724400
H	-1.24809900	3.60505900	3.54699100
C	-1.31107000	2.01401200	2.08763200
H	-1.58389100	1.26336500	2.82896600
C	2.43717900	-0.09090700	2.37926900
C	1.21378000	-0.51832500	3.21325000
H	0.27467200	-0.31000700	2.69693800
H	1.21900100	0.05124800	4.15553200
H	1.23823000	-1.58134100	3.46777900
C	3.72890500	-0.50201000	3.12209400
H	3.75613600	-1.58199200	3.31539600
H	3.76652600	0.01067400	4.09482900
H	4.62821200	-0.22671500	2.55532800
C	2.41979500	1.44973400	2.21935300
H	3.29656000	1.80298600	1.66149000
H	2.43121900	1.91804700	3.21468200
H	1.51259600	1.79070300	1.70437400
C	2.52423800	-2.63429300	0.49953400
C	2.02476100	-2.96816400	-0.92606700
H	0.96059300	-2.73695600	-1.04825500
H	2.16187600	-4.04351700	-1.11277900
H	2.58951100	-2.41547100	-1.68915600
C	1.64263200	-3.34204300	1.54249500
H	2.08016600	-3.27304000	2.54575100
H	1.56870500	-4.41077500	1.28795900
H	0.63106000	-2.92615000	1.57340800
C	3.98288400	-3.13603400	0.62996800

H	4.62622800	-2.68987000	-0.13848800
H	4.00254100	-4.22948600	0.50749900
H	4.41092300	-2.90164900	1.61218200
C	-4.38460500	0.39417800	-0.23207700
C	-4.36411100	1.64124300	0.68254500
H	-4.33260500	1.36574800	1.74468300
H	-5.28875300	2.21155900	0.50736700
H	-3.51680400	2.29718300	0.47055200
C	-4.23106600	0.84366100	-1.69839200
H	-3.25981600	1.32542200	-1.86419900
H	-5.02190300	1.56941900	-1.94486800
H	-4.32547100	0.00331800	-2.39607100
C	-5.75813200	-0.28880400	-0.02184100
H	-5.89881400	-1.16788800	-0.65627400
H	-6.55286300	0.43141100	-0.26834500
H	-5.88596800	-0.59451500	1.02413500
C	-2.74727500	-2.24983900	-0.88678900
C	-1.94239200	-1.84286200	-2.14062700
H	-2.47519400	-1.09378900	-2.73656000
H	-1.78591700	-2.73017900	-2.77509700
H	-0.96264300	-1.42893000	-1.88141000
C	-4.08703300	-2.87213600	-1.32980500
H	-4.71442400	-3.14662500	-0.47261600
H	-3.87238200	-3.78851800	-1.90038800
H	-4.65658800	-2.20426000	-1.98660900
C	-1.96589800	-3.32461600	-0.09630900
H	-1.00941900	-2.93654500	0.26369700
H	-1.76202500	-4.18224900	-0.75596600
H	-2.54390000	-3.67910800	0.76647800
C	0.97274700	-0.26772700	-0.18292800
O	-0.19496600	-0.72068000	0.39102800
N	0.83093500	0.36037500	-1.29592000
C	1.91480300	0.93429300	-1.99355700
C	2.24934700	0.43384400	-3.26639800
C	2.58411500	2.06795200	-1.49297300
C	3.27876100	1.03068400	-4.00316400
H	1.70667600	-0.42528200	-3.65556600
C	3.60058100	2.66805300	-2.24508600
H	2.29193500	2.46917100	-0.52599900
C	3.95945600	2.14949800	-3.49796800
H	3.54516100	0.62464600	-4.97769800
H	4.11562700	3.54120400	-1.84747700
H	4.75358400	2.61566600	-4.07782500

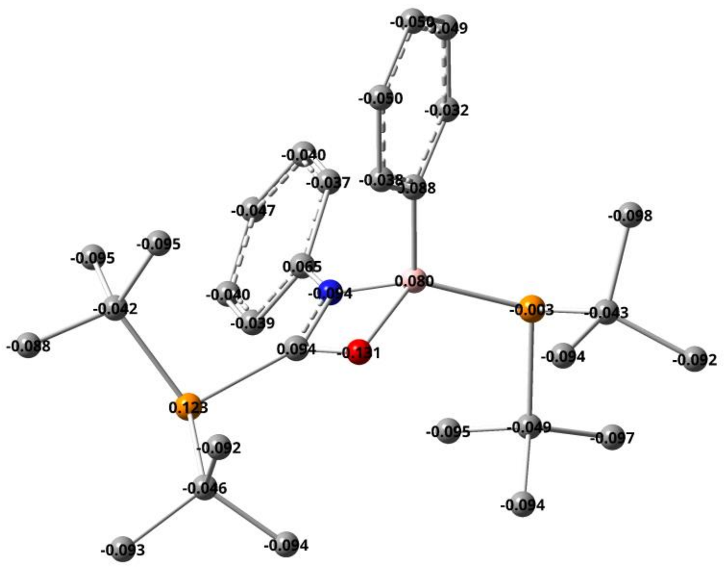

FIG. S97. OPTIMIZED STRUCTURE **1D_I3**

B	-0.94564500	0.19195300	-0.29970000
C	-1.19675400	1.02557100	-1.64068000
P	2.76395400	-0.84913400	0.35883400
C	-0.73008400	0.57409400	-2.89406700
H	-0.19389800	-0.37167300	-2.94906300
P	-2.61094200	-0.11083300	0.72317400
C	-0.95040500	1.30826400	-4.06868600
H	-0.58393700	0.92822700	-5.02172100
C	-1.64177100	2.52658100	-4.01770800
H	-1.81276500	3.10031000	-4.92727500
C	-2.12345700	2.99336800	-2.78521100
H	-2.67946000	3.92876800	-2.73545400
C	-1.90963700	2.24638400	-1.62006600
H	-2.34336900	2.59090300	-0.68105200
C	2.75990700	-2.63696100	-0.31266300
C	2.44662500	-2.82687600	-1.80831400
H	1.47499800	-2.39465100	-2.06805100
H	2.41476000	-3.90437300	-2.03064000
H	3.21936700	-2.38304300	-2.44690600
C	4.15097000	-3.23030300	0.02276300
H	4.95830000	-2.76931800	-0.55526500
H	4.14064500	-4.30390500	-0.21515500
H	4.38338900	-3.12033500	1.08988900
C	1.71282800	-3.39829100	0.53550400
H	1.84941000	-3.21492700	1.61009700
H	1.83176800	-4.47739300	0.35993000
H	0.69564200	-3.11507800	0.25446200
C	3.57172700	0.35912400	-0.91254000
C	3.75134200	1.70209900	-0.16707200
H	2.79019800	2.17028500	0.06841700
H	4.30891900	2.39285100	-0.81707000
H	4.31217200	1.57681900	0.76726200
C	2.74943400	0.60668600	-2.18997900
H	2.55785800	-0.31332000	-2.75017300
H	3.30737200	1.29294900	-2.84512400
H	1.78599100	1.07616400	-1.96321000
C	4.96899400	-0.18855700	-1.27198800

H	5.56158000	-0.41941300	-0.37698200
H	5.50787500	0.58140900	-1.84306500
H	4.91165700	-1.08562600	-1.89844300
C	-2.24156700	-1.25880900	2.22580700
C	-1.03590500	-0.65451400	2.97923300
H	-0.09270400	-0.85353800	2.46045200
H	-0.96896000	-1.11375200	3.97748900
H	-1.13793000	0.42981100	3.11305400
C	-3.45050200	-1.17923200	3.19049600
H	-3.64680500	-0.13895700	3.47922300
H	-3.22491300	-1.75441500	4.10260400
H	-4.36697300	-1.58882300	2.75563500
C	-1.91792400	-2.73025500	1.90574700
H	-2.77592600	-3.26581100	1.48497000
H	-1.62571200	-3.25198700	2.83158800
H	-1.08733900	-2.80306900	1.19676900
C	-3.69905800	-1.09888600	-0.53382400
C	-4.87527400	-1.77662300	0.20289300
H	-4.55070300	-2.58457400	0.86692500
H	-5.55223700	-2.21716700	-0.54518500
H	-5.45021300	-1.04970700	0.79113000
C	-2.92417000	-2.15195100	-1.35418100
H	-2.14501600	-1.67941600	-1.96264300
H	-3.61737600	-2.66886700	-2.03782100
H	-2.45011900	-2.90632900	-0.71828600
C	-4.32667200	-0.07736800	-1.51418800
H	-4.84135300	0.72772800	-0.97447100
H	-5.06535500	-0.60033500	-2.14190800
H	-3.58281000	0.37208600	-2.17691700
C	0.99576900	-0.34960400	0.10731500
O	0.08217300	-1.00646700	-0.59688600
N	0.33021200	0.72325400	0.54021600
C	0.66067500	1.76103000	1.43737800
C	0.03332900	3.00787600	1.27523500
C	1.55381800	1.55762700	2.50723000
C	0.31149300	4.04932600	2.16807500
H	-0.65190900	3.15325500	0.44667800
C	1.82879800	2.60720700	3.38850700
H	2.02504200	0.58730000	2.64152900
C	1.21175500	3.85737400	3.22531700
H	-0.17665500	5.01253200	2.03257500
H	2.51954100	2.44207700	4.21341800
H	1.42641700	4.66874700	3.91775200

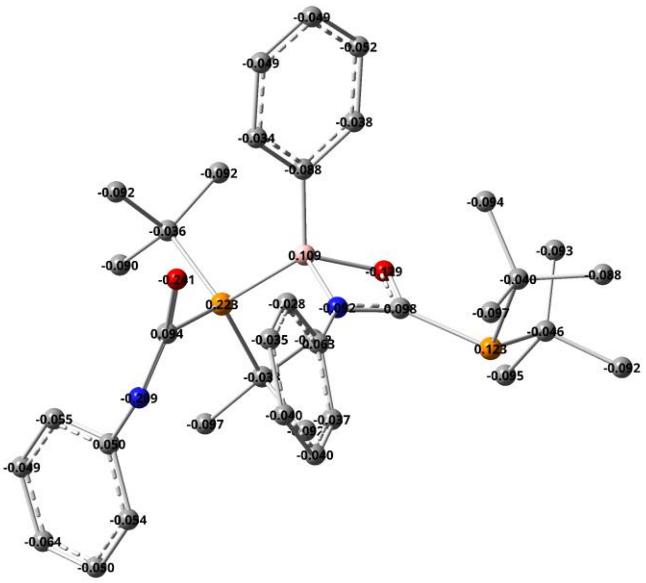

FIG. S98. OPTIMIZED STRUCTURE **1D_I4**

B	0.27472300	1.00863600	0.05231500
C	0.88309500	2.34115500	0.70123400
P	3.01333000	-1.60787500	-0.85715400
C	1.77496600	3.12548300	-0.06920200
H	1.98360900	2.83688800	-1.09737600
P	-1.69391400	0.84266800	-0.42001100
C	2.39877800	4.26583600	0.45388100
H	3.07608200	4.84616900	-0.17176900
C	2.15484800	4.65463700	1.77822800
H	2.63962300	5.53783600	2.19160000
C	1.27737100	3.89315200	2.56240200
H	1.07527700	4.18332100	3.59284200
C	0.64987700	2.75753500	2.03075800
H	-0.04421800	2.18928200	2.64257400
C	3.45981200	-1.04016400	-2.62849400
C	3.93748900	0.41483200	-2.79620100
H	3.18658000	1.12344100	-2.43428000
H	4.11277400	0.61192200	-3.86485700
H	4.88016500	0.59976600	-2.26861800
C	4.53311500	-2.02239200	-3.16049900
H	5.50637600	-1.88978400	-2.67951100
H	4.66633600	-1.84218900	-4.23709800
H	4.21921900	-3.06607800	-3.02814300
C	2.18019300	-1.27272400	-3.46877100
H	1.80056000	-2.29592700	-3.34565400
H	2.42624100	-1.12918400	-4.53118100
H	1.38825700	-0.56832700	-3.20545800
C	4.33222800	-0.98075900	0.41527300
C	4.14716700	-1.84450500	1.68514000
H	3.17319100	-1.68921200	2.15643000
H	4.92281500	-1.56546300	2.41395300
H	4.25386800	-2.91239400	1.45855600
C	4.20506000	0.51248300	0.77217300
H	4.27742000	1.15756900	-0.10948700
H	5.01764800	0.78506300	1.46216500
H	3.25661600	0.73263800	1.27465200
C	5.74124400	-1.27056400	-0.14385300

H	5.85617700	-2.32028400	-0.44292900
H	6.47065300	-1.06942900	0.65411600
H	5.99559700	-0.62843600	-0.99366200
C	-1.88116100	-0.17103200	-2.03957400
C	-1.19839700	-1.53056400	-1.75560700
H	-0.12092400	-1.43736700	-1.61158300
H	-1.36294100	-2.18999100	-2.61992800
H	-1.63557800	-2.01163700	-0.87261500
C	-3.33055200	-0.49041600	-2.46611600
H	-3.85969500	-1.08377800	-1.71974800
H	-3.26716500	-1.08733300	-3.38865900
H	-3.92046800	0.39960700	-2.69038400
C	-1.18752300	0.56774500	-3.20707100
H	-1.78284700	1.42981700	-3.53154300
H	-1.11265000	-0.12066600	-4.06253600
H	-0.18028400	0.90944300	-2.95146700
C	-2.44880700	2.59256100	-0.59392300
C	-3.79249500	2.55685700	-1.35162800
H	-3.64786500	2.38839600	-2.42474200
H	-4.27279600	3.53895500	-1.23381400
H	-4.47364500	1.79308400	-0.96211600
C	-1.49300800	3.55177800	-1.33909800
H	-0.57276900	3.72527700	-0.77665500
H	-2.00417100	4.51860700	-1.46011400
H	-1.23179200	3.18474700	-2.33780500
C	-2.70252100	3.13766500	0.82970500
H	-3.43905800	2.53147300	1.36946500
H	-3.09549500	4.16060300	0.73470300
H	-1.78713600	3.16875900	1.42582900
C	1.64957700	-0.42204000	-0.43948900
O	1.23549000	0.61375100	-1.16757100
N	0.87108500	-0.36139500	0.63744300
C	0.76284200	-1.26661600	1.72623600
C	0.84737900	-0.78891900	3.04203600
C	0.57068500	-2.63311500	1.46899300
C	0.76179400	-1.69390900	4.10451300
H	0.98291100	0.27308400	3.21589300
C	0.49598300	-3.53104800	2.53967400
H	0.48425300	-2.97984000	0.44248900
C	0.59199200	-3.06471800	3.85821900
H	0.82348800	-1.32594800	5.12656300
H	0.34784900	-4.59091900	2.34206600
H	0.52315200	-3.76332100	4.68966100
C	-2.17958800	0.05045300	1.39049600
N	-2.93334300	-0.98749600	1.56606900
O	-1.44741200	0.68450000	2.17875500
C	-4.01081000	-1.34066700	0.77456300
C	-4.16176000	-2.67870400	0.33476600
C	-5.04576200	-0.41742200	0.47911900
C	-5.27604700	-3.05760600	-0.41919900
H	-3.38662400	-3.39867100	0.59085600
C	-6.16050800	-0.80681900	-0.27302200
H	-4.97089000	0.59093700	0.88171100
C	-6.27956200	-2.12495900	-0.73805600
H	-5.36549100	-4.08775600	-0.76203600
H	-6.94179200	-0.07936500	-0.49134900
H	-7.14606100	-2.42728600	-1.32281100

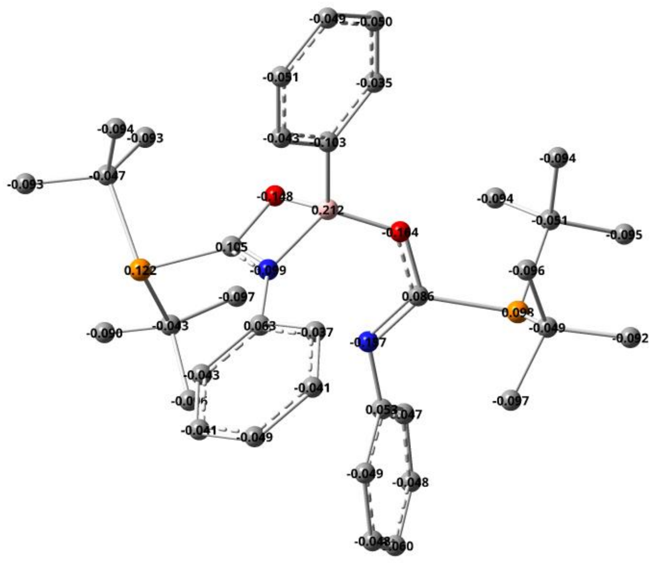

FIG. S99. OPTIMIZED STRUCTURE **1D_I5**

P	3.37664200	-0.31413400	-0.82388000
P	-3.57987400	-0.72536100	-0.12491200
O	1.01944600	1.07487400	-0.42124900
O	-1.43856100	0.85673400	-1.09902200
N	-1.15230300	0.48944400	0.93392900
N	0.80172900	-1.18188400	-0.09554400
C	-1.98084100	0.21234000	-0.07238800
C	-0.52295300	2.98310000	0.16382800
C	0.32357400	0.23380600	4.29391400
H	1.17575600	0.65126000	4.82675500
C	3.54480900	0.87835300	-2.30919800
C	0.08283500	0.62218000	2.97293700
H	0.73316000	1.33880900	2.47760900
C	1.93287100	-5.24795300	0.04802700
H	2.19557900	-6.30315100	0.09175000
C	-1.01063500	0.08356400	2.26686700
C	-1.86940600	-0.83658400	2.89810900
H	-2.71852100	-1.24616000	2.35760700
C	-1.29013700	3.49844300	1.22936800
H	-1.75402400	2.81324600	1.93859200
C	-1.61969100	-1.21295200	4.22161700
H	-2.28474400	-1.92735800	4.70350500
C	0.05927300	3.90835600	-0.72933500
H	0.64997700	3.54063000	-1.56702400
C	-0.52389400	-0.68841400	4.92513700
H	-0.33440100	-0.99489900	5.95175700
C	-1.92683800	-2.06299900	-2.16667200
H	-2.33351900	-1.45197100	-2.97764400
H	-1.64512600	-3.04252600	-2.58181400
H	-1.01916100	-1.58181700	-1.78932500
C	5.63682400	0.50348500	0.58752500
H	6.03517500	-0.46721800	0.26438200
H	6.11277200	0.76893100	1.54353900
H	5.92824900	1.26231900	-0.14984600
C	1.25837600	-2.51358300	-0.06588000
C	1.26005300	-3.19520600	1.16767500

H	0.98641600	-2.64989400	2.06918100
C	1.60977900	-4.54904700	1.22167300
H	1.62096500	-5.06117300	2.18271000
C	1.57093300	-3.21849300	-1.24488500
H	1.54455200	-2.69364600	-2.19735500
C	4.88336900	0.50573800	-2.99279600
H	5.74484800	0.71794300	-2.34751400
H	4.99974900	1.09732200	-3.91359400
H	4.91192600	-0.55835800	-3.25933600
C	-4.13745600	0.50218800	-2.75670400
H	-3.09533300	0.82371200	-2.84400000
H	-4.77272600	1.23705500	-3.27472800
H	-4.26122800	-0.45878900	-3.27033800
C	1.54295000	-0.18153700	-0.41470000
C	-6.04150100	-0.08416000	-1.20198700
H	-6.16185700	-1.08111400	-1.64160800
H	-6.68855300	0.60851900	-1.75980800
H	-6.39390300	-0.12053800	-0.16317600
B	-0.33931900	1.42275300	-0.07055400
C	-0.87699600	5.77894600	0.50378800
H	-1.01062600	6.85169300	0.63534000
C	-4.57936100	0.41879700	-1.28474900
C	-0.11394700	5.28877800	-0.56720400
H	0.34374100	5.98120900	-1.27268200
C	1.90561300	-4.57591000	-1.18358900
H	2.14980400	-5.10884100	-2.10138300
C	-2.26646500	-3.11441500	0.08788500
H	-1.42547000	-2.56796300	0.52960700
H	-1.86266000	-4.04456600	-0.33709000
H	-2.97776600	-3.37553400	0.88220800
C	-1.46568800	4.87912000	1.40298200
H	-2.05775300	5.25081200	2.23833500
C	3.52205400	2.38910800	-2.02208300
H	2.64398500	2.67368800	-1.43661200
H	3.50153100	2.93802500	-2.97689200
H	4.42348300	2.70519300	-1.48288700
C	-4.51154700	1.82349800	-0.63593700
H	-4.76948700	1.78703300	0.43156100
H	-5.23876500	2.48066400	-1.13482900
H	-3.51911300	2.27477300	-0.74235300
C	2.39126000	0.51127600	-3.27353500
H	2.35480900	-0.57123000	-3.46030300
H	2.55469400	1.01206500	-4.23950700
H	1.41957300	0.83099500	-2.88117500
C	4.10428500	0.45412700	0.77564300
C	3.59479900	1.83545600	1.23194400
H	3.94922100	2.64161500	0.58373600
H	3.97415600	2.03257200	2.24700900
H	2.50250100	1.88242000	1.25490900
C	3.76440100	-0.58837600	1.86919400
H	2.68661600	-0.61743800	2.07184600
H	4.27615700	-0.31214600	2.80357100
H	4.08462300	-1.59783700	1.58230300
C	-2.94502300	-2.29230600	-1.03389100
C	-4.17359700	-3.06213100	-1.56108300
H	-4.93638100	-3.19815400	-0.78252300
H	-3.84902000	-4.05878300	-1.89405900

H	-4.63437700	-2.55809000	-2.41926900

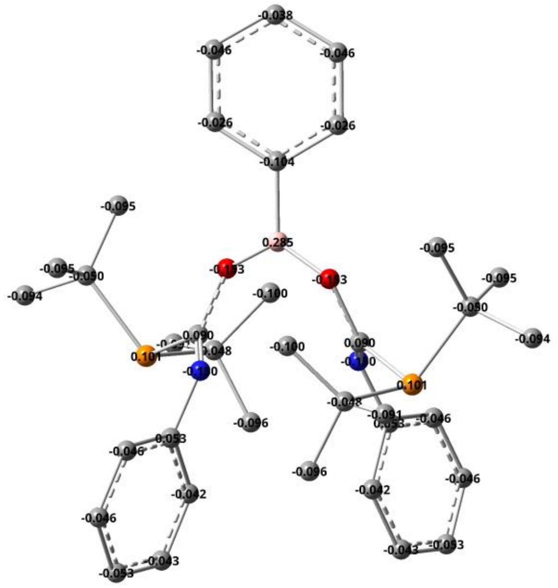

FIG. S100. OPTIMIZED STRUCTURE **1D_I6**

P	-3.29821100	-0.55878200	-0.03095300
P	3.29821600	-0.55874000	0.03095200
O	-1.24438300	1.30644400	0.02303600
O	1.24435600	1.30645400	-0.02304300
N	0.83644700	-0.47125800	-1.37543500
N	-0.83644500	-0.47125400	1.37543900
C	1.59506100	0.05688600	-0.48642800
C	-0.00002600	3.48683200	-0.00000500
C	1.22993300	-3.02310800	-4.02239500
H	1.20909900	-3.06610900	-5.11011800
C	-4.34072000	0.97334700	0.47590000
C	1.05049000	-1.79538400	-3.37705200
H	0.87895200	-0.88235100	-3.94354400
C	-1.41850000	-4.19697800	3.27532900
H	-1.54178000	-5.15355900	3.77935500
C	1.09490600	-1.72155300	-1.97009600
C	1.27696900	-2.90090300	-1.22160800
H	1.27115900	-2.85060900	-0.13725800
C	1.09735000	4.21638900	-0.51052900
H	1.95180500	3.67709900	-0.91398400
C	1.43374900	-4.12884200	-1.87475700
H	1.56884400	-5.03341300	-1.28390200
C	-1.09741200	4.21637300	0.51052200
H	-1.95186000	3.67707000	0.91397300
C	1.41857000	-4.19697400	-3.27532100
H	1.54186900	-5.15355300	-3.77934700
C	2.36256400	0.42595500	2.66479300
H	2.75304800	1.43057100	2.47770100
H	2.41573800	0.23650400	3.74880200
H	1.30976300	0.39596200	2.36543700
C	-4.61254600	-0.72484300	-2.49523200
H	-5.21880400	-1.47790200	-1.97361800
H	-4.57018700	-1.00354900	-3.55856100

H	-5.12218200	0.24296600	-2.42642400
C	-1.09488200	-1.72155300	1.97010300
C	-1.27692600	-2.90090500	1.22161600
H	-1.27111700	-2.85061100	0.13726500
C	-1.43368300	-4.12884700	1.87476500
H	-1.56876400	-5.03342100	1.28391000
C	-1.05046100	-1.79538100	3.37705900
H	-0.87893800	-0.88234600	3.94355000
C	-5.80596900	0.47583900	0.54746700
H	-6.19574600	0.19399800	-0.43717800
H	-6.44141900	1.28371700	0.94071600
H	-5.89829200	-0.39104200	1.21372800
C	4.26353800	2.22422800	0.41735600
H	3.23362900	2.56891900	0.54225900
H	4.84747000	3.03440100	-0.04761400
H	4.69151400	2.04116000	1.40985200
C	-1.59506600	0.05687300	0.48642900
C	5.80596000	0.47591500	-0.54746900
H	6.19574000	0.19408100	0.43717700
H	6.44139900	1.28380100	-0.94072000
H	5.89829600	-0.39096700	-1.21372800
B	-0.00001600	1.92641000	-0.00000300
C	-0.00004500	6.31892400	0.00000000
H	-0.00005100	7.40783900	0.00000200
C	4.34070300	0.97340300	-0.47590400
C	-1.09821500	5.61613500	0.51872000
H	-1.95014500	6.15864900	0.92553400
C	-1.22988000	-3.02310900	4.02240200
H	-1.20904300	-3.06611100	5.11012500
C	2.50295900	-2.03582300	2.20562400
H	1.47078800	-2.05549100	1.84032200
H	2.46543300	-2.21746400	3.28994400
H	3.06259300	-2.85489100	1.73576600
C	1.09813400	5.61615100	-0.51872400
H	1.95005700	6.15867800	-0.92553500
C	-4.26357100	2.22417100	-0.41736400
H	-3.23366600	2.56887500	-0.54226800
H	-4.84751200	3.03433800	0.04760500
H	-4.69154500	2.04109500	-1.40985900
C	3.89527100	1.32856300	-1.91503300
H	3.88396700	0.44120900	-2.56265500
H	4.60462000	2.05241400	-2.34298700
H	2.89644800	1.77941400	-1.93373700
C	-3.89529100	1.32851500	1.91502800
H	-3.88397800	0.44116300	2.56265200
H	-4.60464900	2.05235900	2.34298000
H	-2.89647300	1.77937700	1.93373200
C	-3.17182000	-0.66646900	-1.93983500
C	-2.36256900	0.42592800	-2.66479100
H	-2.75307000	1.43053700	-2.47769900
H	-2.41574000	0.23647800	-3.74880100
H	-1.30976800	0.39595000	-2.36543400
C	-2.50292800	-2.03585400	-2.20562200
H	-1.47075700	-2.05550600	-1.84031800
H	-2.46539800	-2.21749400	-3.28994100
H	-3.06255000	-2.85492900	-1.73576300
C	3.17183000	-0.66642800	1.93983500

C	4.61255800	-0.72478000	2.49523000
H	5.21882700	-1.47782800	1.97361300
H	4.57020600	-1.00348900	3.55855800
H	5.12217800	0.24303700	2.42642200

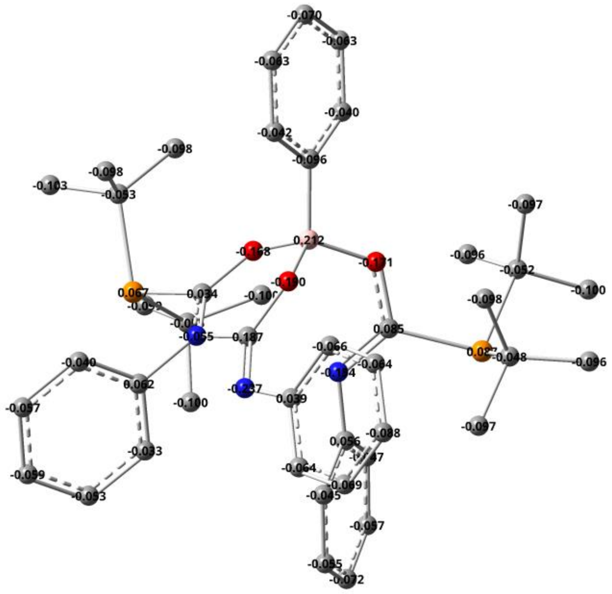

FIG. S101. OPTIMIZED STRUCTURE **1D_I7**

P	-3.18817500	-0.74397700	-1.69369700
P	3.65791000	-0.21070000	-0.68286400
O	-1.17366700	-1.50260200	0.02256700
O	1.24867200	-1.19344600	0.17163900
O	-0.22883100	-0.10516900	1.79479500
N	1.59583200	0.98492700	0.80367000
N	-0.05743100	2.23999500	1.82347600
N	-0.75484100	0.49966800	-0.98517500
C	1.98419000	-0.13812600	0.16650900
C	0.09017100	-2.63327700	1.89160100
C	3.07751300	4.26502300	-0.21383500
H	2.95748100	5.01142400	-0.99654000
C	-3.20626800	1.99992700	3.87333100
H	-3.75212600	1.29410300	4.49775900
C	-3.26750300	-2.63871100	-1.94654900
C	2.25803400	3.13021400	-0.21290200
H	1.48926100	2.98837100	-0.96689200
C	-3.72202000	3.28577100	3.65479500
H	-4.66753100	3.58178200	4.10556500
C	-1.18237900	3.60361900	-3.82225600
H	-1.26604100	4.41106800	-4.54712800
C	2.42844900	2.17815800	0.79422700
C	-1.78616900	3.80749200	2.28713000
H	-1.20683100	4.50108300	1.68071200
C	3.36889600	2.34661900	1.81446100
H	3.45981100	1.59335100	2.59371700
C	-1.99878200	1.60009400	3.28921300
H	-1.61798200	0.59903100	3.45806200
C	-0.04338400	-3.92397500	1.33800100
H	-0.32010700	-4.02695300	0.28880700
C	4.18352900	3.48466200	1.80564400
H	4.92613200	3.62268000	2.58876100

C	0.44482800	-2.53799800	3.25204500
H	0.54848400	-1.55163100	3.70480700
C	4.04189500	4.44139300	0.78932900
H	4.67757900	5.32463100	0.78318600
C	0.32904300	1.06009500	1.49861500
C	1.87630700	-1.42000000	-2.73613500
H	2.05830800	-2.43506600	-2.37326500
H	1.69836600	-1.47212400	-3.82147700
H	0.96580800	-1.04210800	-2.26247400
C	-5.75439100	-0.54566700	-0.60278700
H	-6.01227800	-0.18377500	-1.60718000
H	-6.43672700	-0.06922700	0.11696600
H	-5.93389800	-1.62730800	-0.56467800
C	-0.97169000	1.50841100	-1.94364300
C	-1.27640900	2.50240700	2.47329700
C	-1.04824600	2.84834500	-1.51225000
H	-1.02035600	3.05622200	-0.44520400
C	-1.16502900	3.88376800	-2.44737000
H	-1.23902500	4.91291700	-2.09829000
C	-0.98024400	1.23004600	-3.32682500
H	-0.89801300	0.19746400	-3.65855800
C	-4.42261400	-2.87814800	-2.95006600
H	-5.39840100	-2.62007100	-2.52150800
H	-4.44872700	-3.94315800	-3.22668600
H	-4.28496000	-2.28697300	-3.86456500
C	3.62537500	-3.17285400	-0.36651100
H	2.54982500	-3.17927500	-0.17010500
H	4.07860500	-4.00255600	0.19725900
H	3.79866200	-3.36563700	-1.43195600
C	-1.52567500	-0.52822200	-0.84273000
C	5.79670100	-1.89017500	-0.22803500
H	5.99412000	-1.99620200	-1.30106000
H	6.24780500	-2.75363600	0.28256900
H	6.29868200	-0.98033400	0.12512000
B	-0.06905700	-1.33602300	0.97824300
C	0.52081200	-4.95622800	3.46230100
H	0.68446500	-5.84810900	4.06554200
C	4.28045600	-1.85642800	0.08683900
C	0.65666000	-3.68312500	4.03422300
H	0.92471600	-3.58368300	5.08551900
C	-1.08514200	2.27164700	-4.25436100
H	-1.09260100	2.04158000	-5.31882300
C	2.68136000	0.94077200	-2.97567300
H	1.78373700	1.30301600	-2.46251700
H	2.44783600	0.90273000	-4.04969100
H	3.49368600	1.66144800	-2.81949100
C	0.16825800	-5.07468000	2.10898700
H	0.05698500	-6.06070800	1.65920800
C	-3.47070500	-3.53236000	-0.71000200
H	-2.70469300	-3.35049300	0.04859400
H	-3.41466600	-4.58850000	-1.01865100
H	-4.45958000	-3.37595500	-0.26170700
C	4.11102700	-1.69577000	1.61764600
H	4.51218900	-0.73427100	1.96780700
H	4.66895600	-2.49627900	2.12446100
H	3.06232600	-1.78228000	1.92443600
C	-1.93980200	-3.01418500	-2.64898600

H	-1.74775500	-2.36537800	-3.51559000
H	-2.00316800	-4.05040500	-3.01399800
H	-1.08922400	-2.94231000	-1.96269000
C	-4.29875800	-0.19451800	-0.22681900
C	-3.96369300	-0.75330400	1.16863300
H	-4.04258100	-1.84269700	1.21565500
H	-4.67302600	-0.32570600	1.89422700
H	-2.95634300	-0.46699500	1.48268900
C	-3.00265600	4.19028700	2.85826700
H	-3.38583900	5.19522900	2.68876800
C	-4.14323900	1.34496200	-0.18588600
H	-3.12746100	1.63418300	0.10704500
H	-4.83127600	1.75738500	0.56675500
H	-4.36765500	1.80326000	-1.15725400
C	3.06803800	-0.47611200	-2.49032500
C	4.30349400	-0.95856500	-3.28260500
H	5.18030100	-0.32322700	-3.09820900
H	4.07358200	-0.91415000	-4.35706500
H	4.56467900	-1.99560300	-3.04060500

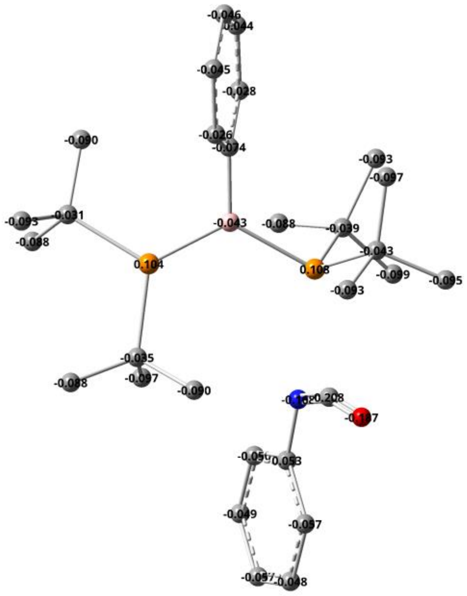

FIG. S102. OPTIMIZED STRUCTURE **1D_TS1_A**

B	1.43211900	0.03283900	0.01766600
C	2.97580600	-0.24289400	-0.19309900
P	0.09897400	-1.44051200	0.14447800
C	3.56688100	-0.66811300	-1.40427000
H	2.94756600	-0.79829000	-2.28884800
P	1.08683000	1.78785600	0.43733100
C	4.94691000	-0.87336100	-1.51813300
H	5.36802900	-1.18479500	-2.47308900
C	5.78700500	-0.67094700	-0.41268700
H	6.85941400	-0.83614100	-0.49972600
C	5.23054400	-0.24505900	0.80126500
H	5.86941700	-0.07394700	1.66654800
C	3.84996100	-0.02671700	0.90287700
H	3.42964900	0.31368800	1.84824700
C	0.83299100	-2.72217200	1.36356500
C	-0.09404100	-3.95815800	1.40256300
H	-1.14260300	-3.68361100	1.55442300

H	0.21499200	-4.60517100	2.23757900
H	-0.00766500	-4.54836700	0.48168100
C	2.27896900	-3.20464900	1.12604500
H	2.38638600	-3.75276700	0.18599700
H	2.54577600	-3.89254400	1.94404800
H	2.99874000	-2.38331200	1.13044300
C	0.77225400	-1.99066800	2.72720300
H	1.40526000	-1.09218200	2.72272600
H	1.14322700	-2.66229900	3.51592500
H	-0.25284000	-1.69586100	2.97887300
C	0.00457700	-2.15305300	-1.63915300
C	-0.07250100	-0.92249100	-2.57194900
H	-0.89771700	-0.26405800	-2.27316900
H	-0.25213900	-1.25761100	-3.60491400
H	0.85353900	-0.33255300	-2.55840200
C	-1.30617400	-2.95959500	-1.80126300
H	-1.36313400	-3.79358900	-1.09247000
H	-1.33043600	-3.38210900	-2.81778500
H	-2.18578800	-2.32475600	-1.66714200
C	1.17239300	-3.07696000	-2.03933100
H	2.15428900	-2.65102500	-1.82233800
H	1.11126200	-3.27506700	-3.12117800
H	1.09729400	-4.04275400	-1.52538200
C	2.11343900	3.17816300	-0.38996200
C	3.27738200	2.61636500	-1.23090400
H	4.02020100	2.09106400	-0.62522500
H	3.77325700	3.47058200	-1.71616200
H	2.92182300	1.93745400	-2.01275300
C	1.20502900	3.97173400	-1.35992500
H	0.80816200	3.31952100	-2.14706600
H	1.81049100	4.75737400	-1.83637200
H	0.36526400	4.46156200	-0.85950700
C	2.70716700	4.11136500	0.69067500
H	1.93626100	4.59028000	1.30145100
H	3.29008600	4.90479200	0.19901700
H	3.37824100	3.55312400	1.35427500
C	-0.62195000	2.38333600	1.01043600
C	-1.64918900	2.37340200	-0.14076800
H	-1.38880700	3.10026600	-0.91799300
H	-2.64254100	2.63612000	0.25295700
H	-1.72134500	1.37877800	-0.58950400
C	-0.56116100	3.78590200	1.65722400
H	0.16424700	3.82003900	2.47937600
H	-1.55467000	4.00239200	2.07621600
H	-0.32649100	4.58418600	0.94559600
C	-1.03803600	1.40104200	2.12943200
H	-0.97852200	0.36100900	1.80387600
H	-2.07689100	1.60902500	2.42386000
H	-0.39258800	1.51876600	3.00870700
C	-2.43399400	-1.21906200	0.80654200
O	-2.49087500	-1.76978800	1.87479300
N	-2.84665600	-0.60897700	-0.21126400
C	-4.18421200	-0.14571100	-0.32648500
C	-5.14271400	-0.24819300	0.70271700
C	-4.53432200	0.47024600	-1.54307500
C	-6.43178800	0.26168000	0.50543200
H	-4.87678800	-0.72415600	1.64442800

C	-5.82588900	0.97516100	-1.72987100
H	-3.78214800	0.54351800	-2.32575800
C	-6.78146700	0.87528700	-0.70723900
H	-7.16615500	0.17772700	1.30513000
H	-6.08542500	1.44839200	-2.67555500
H	-7.78546200	1.26884100	-0.85316800

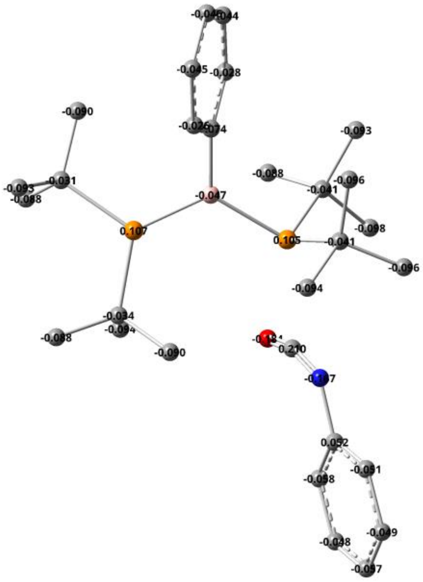

FIG. S103. OPTIMIZED STRUCTURE **1D_TS1_B**

B	-1.50668600	-0.14874500	0.00439100
C	-2.86175700	-0.88603400	-0.35491000
P	0.21016400	-1.09426600	0.32874800
C	-3.64489700	-1.65429200	0.53616400
H	-3.33789700	-1.75640100	1.57425600
P	-1.63189500	1.67046000	-0.17199000
C	-4.84868100	-2.24735900	0.13654400
H	-5.43304300	-2.81697000	0.85788800
C	-5.30785600	-2.10227200	-1.18107700
H	-6.24200200	-2.56648200	-1.49228500
C	-4.55363100	-1.34597100	-2.08880000
H	-4.89888700	-1.21597600	-3.11355300
C	-3.35753400	-0.74347800	-1.67679400
H	-2.78529600	-0.14838700	-2.38751200
C	0.48420400	-2.23909100	-1.18323900
C	1.69888500	-3.15289500	-0.90832200
H	2.56064700	-2.57943900	-0.55052500
H	1.98072700	-3.65697400	-1.84561300
H	1.45454600	-3.93374900	-0.17740500
C	-0.70672900	-3.10970200	-1.63120500
H	-1.01550600	-3.82527500	-0.86405500
H	-0.39584900	-3.68413600	-2.51839500
H	-1.57629600	-2.50846300	-1.90787000
C	0.83720100	-1.25792700	-2.32667000
H	0.00308000	-0.57242400	-2.53241800
H	1.04012300	-1.83050600	-3.24456000
H	1.72629500	-0.66772100	-2.07686200
C	-0.10399100	-2.12570200	1.91937800
C	-0.78500100	-1.15460000	2.91221200
H	-0.17570900	-0.25344000	3.05470900

H	-0.89997000	-1.65065000	3.88796200
H	-1.77811600	-0.83719200	2.56959900
C	1.25315600	-2.55281600	2.53070000
H	1.84673500	-3.15504400	1.83461900
H	1.05370100	-3.16939300	3.42080900
H	1.84750400	-1.69006100	2.84381100
C	-0.94851800	-3.40102700	1.72283300
H	-1.87277200	-3.22278300	1.16891100
H	-1.21042200	-3.81001400	2.71155500
H	-0.37391000	-4.16971800	1.19218400
C	-3.27956600	2.56155100	0.21939700
C	-4.36693200	1.56938400	0.68266900
H	-4.68663500	0.89354500	-0.11406800
H	-5.23901400	2.16177800	0.99808900
H	-4.03206200	0.97036300	1.53638300
C	-3.05081500	3.54614400	1.39237600
H	-2.71766000	3.01374100	2.29138200
H	-4.00724300	4.04004400	1.61990800
H	-2.32290200	4.32923800	1.16343500
C	-3.80475500	3.30804000	-1.02859800
H	-3.10774200	4.07332100	-1.38338800
H	-4.75392700	3.80524600	-0.77754200
H	-3.99257800	2.60281400	-1.84693500
C	-0.13935500	2.84429900	-0.18888700
C	0.41240700	3.10690400	1.22897300
H	-0.31511600	3.63698800	1.85338600
H	1.31462100	3.73317100	1.15377400
H	0.68998800	2.17417700	1.72668600
C	-0.48489500	4.18232300	-0.88342000
H	-0.87174100	4.02002900	-1.89696000
H	0.44445600	4.76438000	-0.96711800
H	-1.20295300	4.79175600	-0.32566100
C	0.92952200	2.15914000	-1.06904200
H	1.18434400	1.15976600	-0.71154200
H	1.84560000	2.76777700	-1.05539000
H	0.58337100	2.07250900	-2.10626100
C	2.50607600	0.01905000	0.97648900
O	2.27025000	0.49940600	2.05502000
N	3.16818400	-0.30521600	-0.03722200
C	4.52557700	0.06795500	-0.22488400
C	5.25764300	0.84365200	0.69745700
C	5.14387200	-0.37134800	-1.41060300
C	6.59181000	1.17037700	0.42583100
H	4.78288500	1.18359900	1.61575200
C	6.47818100	-0.03973800	-1.67104300
H	4.56417000	-0.96847900	-2.11114800
C	7.20939200	0.73263900	-0.75574600
H	7.15063400	1.76965100	1.14307200
H	6.94696700	-0.38528300	-2.59100900
H	8.24689400	0.98990400	-0.95972600

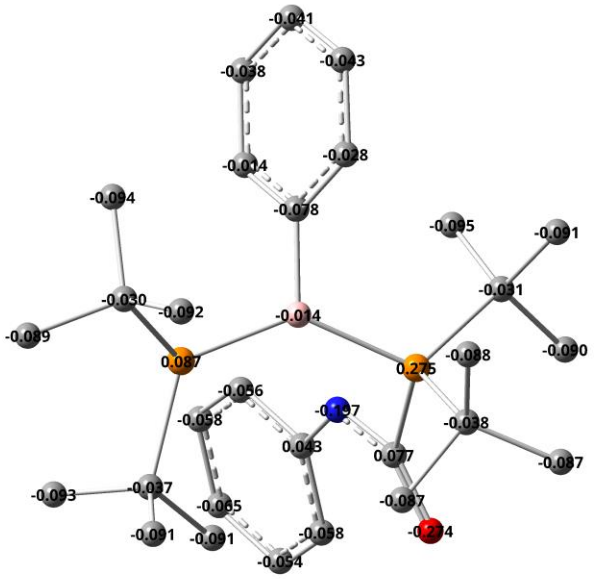

FIG. S104. OPTIMIZED STRUCTURE **1D_TS2_A**

B	-0.90789300	-0.03070600	0.08698600
C	-1.79870000	-0.50212900	1.29071500
P	0.09132400	-1.47637300	-0.78713900
C	-1.48817600	-0.07493600	2.60394900
H	-0.63036800	0.57850700	2.75005500
P	-0.88506300	1.84814800	-0.02967800
C	-2.24763700	-0.49590500	3.70230100
H	-1.98108500	-0.16297300	4.70414900
C	-3.35317900	-1.33774200	3.51297000
H	-3.94924100	-1.66023700	4.36476900
C	-3.68644700	-1.76548300	2.21780000
H	-4.54789100	-2.41306900	2.06148900
C	-2.90829200	-1.36400800	1.12544700
H	-3.18390600	-1.70008000	0.12690700
C	-0.56554300	-1.78786300	-2.53280700
C	0.16997900	-2.95002300	-3.23123900
H	1.25315600	-2.78318600	-3.22178900
H	-0.16497400	-2.98585600	-4.27821600
H	-0.05309800	-3.92212900	-2.77898900
C	-2.09155100	-2.01969400	-2.50443100
H	-2.37558500	-2.91427000	-1.94154200
H	-2.44957600	-2.14150000	-3.53714900
H	-2.60830200	-1.15187800	-2.07319500
C	-0.28787700	-0.49332000	-3.32264500
H	-0.76314400	0.36364400	-2.83279700
H	-0.71871400	-0.59409300	-4.32938000
H	0.78788200	-0.31151600	-3.41760600
C	0.23031600	-3.11161000	0.21343100
C	0.35166700	-2.80736600	1.72496700
H	1.05144200	-1.98502400	1.90669700
H	0.73605800	-3.71469100	2.21336200
H	-0.61613400	-2.56564800	2.17056800
C	1.54658400	-3.80091700	-0.22953500
H	1.59668200	-3.99010400	-1.30520500
H	1.60775200	-4.76731300	0.29090100
H	2.41877100	-3.20360700	0.05549700
C	-0.95882700	-4.06881300	-0.00672200

H	-1.90874800	-3.61134600	0.28844500
H	-0.80352400	-4.95015300	0.63218600
H	-1.03354800	-4.42163200	-1.04103200
C	-2.71416600	2.49008400	0.01279400
C	-3.41563600	2.15419400	1.34981300
H	-3.63353800	1.09085400	1.45910300
H	-4.37321200	2.69652100	1.36851100
H	-2.82011600	2.47898800	2.21035100
C	-2.74526900	4.03234300	-0.09300800
H	-2.22020800	4.49911100	0.74880900
H	-3.79736200	4.34956200	-0.05006400
H	-2.32772800	4.41712800	-1.02649300
C	-3.51944800	1.86527200	-1.14856400
H	-3.06132700	2.06742800	-2.12327300
H	-4.54057200	2.27832000	-1.15680200
H	-3.59180700	0.77844900	-1.01930400
C	0.14527800	2.86870500	-1.28953800
C	0.51345400	4.19984500	-0.57906600
H	-0.34839600	4.84346000	-0.38618100
H	1.20774000	4.75469000	-1.22745600
H	1.02102900	4.00184900	0.37248900
C	-0.52839200	3.16711800	-2.64630900
H	-0.74848500	2.25216900	-3.20449000
H	0.16831700	3.76597600	-3.25201300
H	-1.45568000	3.74032100	-2.55100500
C	1.47796000	2.14050100	-1.54455900
H	2.01808500	1.94887700	-0.61358100
H	2.10378800	2.78991400	-2.17444600
H	1.35047000	1.19485100	-2.07238400
C	1.89104900	-0.87844600	-0.82686000
O	2.62702900	-1.04068000	-1.82486400
N	2.01634800	-0.36894100	0.39526000
C	3.21255000	0.18641200	0.87312900
C	4.43221500	0.26995300	0.15336500
C	3.16360100	0.72457000	2.18209900
C	5.55117500	0.87471600	0.74011200
H	4.47861000	-0.13852300	-0.85156700
C	4.28655200	1.32612700	2.75573900
H	2.22156100	0.66109500	2.72457700
C	5.49129200	1.40570400	2.03756400
H	6.48097800	0.93056500	0.17460800
H	4.22377600	1.73434100	3.76388100
H	6.36788800	1.87340200	2.48256000

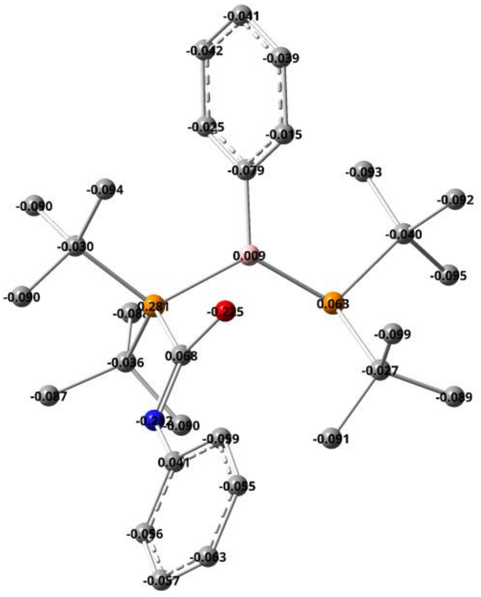

FIG. S105. OPTIMIZED STRUCTURE **1D_TS2_B**

B	1.15708100	-0.08654600	0.00709400
C	2.32075200	-0.70234100	-0.83499000
P	-0.24921100	-1.37101000	0.58199200
C	2.49202700	-0.25731800	-2.16512200
H	1.81528300	0.49904400	-2.55897500
P	1.36544000	1.63115300	0.82603600
C	3.48604700	-0.80365900	-2.98717200
H	3.58735400	-0.46021100	-4.01540100
C	4.35436800	-1.78441200	-2.48505100
H	5.13477200	-2.20101800	-3.11924100
C	4.21345800	-2.22505100	-1.15954000
H	4.89041900	-2.97849600	-0.75979600
C	3.19679400	-1.70105100	-0.35362800
H	3.10596400	-2.04120900	0.67741700
C	-0.39573600	-1.53392600	2.46304900
C	-1.40653700	-2.61584300	2.89289700
H	-2.39169800	-2.41988900	2.45420000
H	-1.50275000	-2.57295600	3.98738700
H	-1.08562400	-3.62893700	2.62843800
C	1.01007400	-1.80850100	3.04445600
H	1.42365700	-2.76633300	2.70875500
H	0.93812600	-1.83748000	4.14129000
H	1.70808200	-1.00437900	2.77635500
C	-0.90601300	-0.17293600	2.98345000
H	-0.20923200	0.63654300	2.73446000
H	-0.97860300	-0.23131200	4.07943500
H	-1.89569000	0.05427200	2.57280100
C	-0.21225900	-3.07662800	-0.29883600
C	0.22315100	-2.91034600	-1.77521400
H	-0.29714200	-2.07769600	-2.25961200
H	-0.03811400	-3.84167500	-2.29847400
H	1.30049000	-2.75807700	-1.87020500
C	-1.66093400	-3.62811900	-0.30413500
H	-2.08133900	-3.74982400	0.69687500
H	-1.63559100	-4.61624200	-0.78550300
H	-2.33185200	-2.98030800	-0.87652900
C	0.72745800	-4.08425900	0.39449000

H	1.76227000	-3.72888200	0.41540200
H	0.70981500	-5.01769900	-0.18601500
H	0.41008200	-4.32379300	1.41511200
C	3.12778200	2.35231100	0.51750700
C	4.18276100	1.23319900	0.68655900
H	3.94230000	0.56495800	1.52468400
H	5.15221300	1.70609600	0.90278000
H	4.29638000	0.63212500	-0.21690700
C	3.35859100	3.04236100	-0.84064400
H	3.18505300	2.34814600	-1.67021100
H	4.40368400	3.38470100	-0.89683600
H	2.71702400	3.91939800	-0.97788200
C	3.38711000	3.35020300	1.67861600
H	2.71504800	4.21061700	1.67167500
H	4.41631100	3.72916900	1.58836600
H	3.29130500	2.84748600	2.64932900
C	0.02702600	2.91516400	0.27178800
C	-0.12594100	2.93866300	-1.26366100
H	0.80970200	3.22188000	-1.75800900
H	-0.88893400	3.68777800	-1.52902100
H	-0.45382100	1.96376900	-1.63460200
C	0.38721900	4.33055400	0.77174200
H	0.53247300	4.35816500	1.85859500
H	-0.45882900	4.99004300	0.52819700
H	1.27353800	4.74406800	0.27953000
C	-1.31398500	2.54208200	0.93355800
H	-1.64344300	1.53066300	0.69450900
H	-2.08596600	3.23187400	0.56293700
H	-1.25745800	2.64534500	2.02404000
C	-1.61576800	-0.52121900	-0.32528600
O	-1.05327400	-0.04029700	-1.38277800
N	-2.82456300	-0.54518500	0.17062400
C	-3.90597900	0.10777700	-0.44928400
C	-3.86298300	0.78598800	-1.69411500
C	-5.12994200	0.07941400	0.25968700
C	-5.01212400	1.41430700	-2.19085300
H	-2.93101400	0.80809500	-2.24992900
C	-6.27072200	0.70896000	-0.24555700
H	-5.15461200	-0.44643600	1.21272000
C	-6.21906300	1.38411800	-1.47613200
H	-4.96251000	1.93142400	-3.14880300
H	-7.20134300	0.67436600	0.31960000
H	-7.10629300	1.87514600	-1.87241200

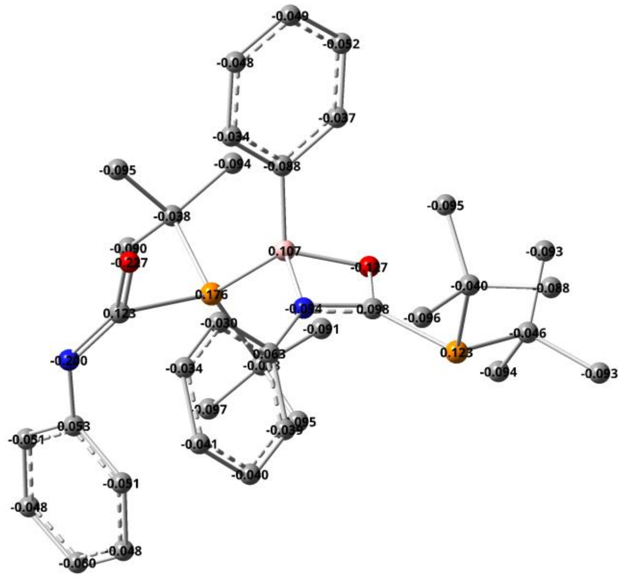

FIG. S106. OPTIMIZED STRUCTURE **1D_TS3**

B	-0.34633800	1.03688100	-0.03913700
C	-1.00672600	2.33262200	-0.71088600
P	-2.98437900	-1.73585800	0.67404100
C	-1.90781500	3.10412300	0.06190700
H	-2.08831900	2.82940200	1.09951800
P	1.59349600	0.97180000	0.50780200
C	-2.58403100	4.20902800	-0.47180600
H	-3.26640100	4.78020300	0.15673100
C	-2.38826100	4.57145700	-1.81145600
H	-2.91489100	5.42580400	-2.23410500
C	-1.50433500	3.82127500	-2.59910500
H	-1.33727000	4.09241200	-3.64083100
C	-0.82269700	2.72336900	-2.05541400
H	-0.11545300	2.17858800	-2.67179400
C	-3.57091900	-1.23228500	2.42223700
C	-4.16619100	0.17922500	2.58140200
H	-3.45807600	0.94865900	2.25894400
H	-4.40167300	0.34815800	3.64324900
H	-5.09791000	0.29528500	2.01607600
C	-4.60240800	-2.30067000	2.86380100
H	-5.53861500	-2.24437600	2.30016400
H	-4.84148500	-2.13789300	3.92474000
H	-4.19533600	-3.31478000	2.75909300
C	-2.33508100	-1.38385400	3.34197300
H	-1.85898600	-2.36593100	3.21839900
H	-2.66334700	-1.29804600	4.38820500
H	-1.59245500	-0.60494400	3.15359800
C	-4.23301100	-1.12267300	-0.67002000
C	-3.83774600	-1.85581600	-1.97301200
H	-2.85067800	-1.55205300	-2.33405600
H	-4.57159600	-1.60459700	-2.75330600
H	-3.83451900	-2.94430000	-1.83743000
C	-4.22510600	0.39842600	-0.91436200
H	-4.45459700	0.96889300	-0.00942800
H	-4.98709500	0.63954700	-1.67071600
H	-3.25866100	0.74499900	-1.29672300
C	-5.64783000	-1.58930300	-0.26734600

H	-5.67695500	-2.66253300	-0.03817400
H	-6.32319800	-1.40922500	-1.11626600
H	-6.04017500	-1.03249200	0.59062400
C	1.75256000	-0.04831800	2.13990900
C	1.11567100	-1.42727000	1.85379400
H	0.03588300	-1.36549500	1.71113700
H	1.29811900	-2.08105000	2.71924600
H	1.56961300	-1.89748600	0.97603100
C	3.21749000	-0.31589400	2.54928100
H	3.78884400	-0.78712100	1.74730700
H	3.20125900	-1.00889300	3.40390700
H	3.74109300	0.58731700	2.86853500
C	1.02295300	0.62779700	3.32151800
H	1.57975300	1.49915900	3.68494200
H	0.96136800	-0.09152200	4.15286900
H	0.00605700	0.93921300	3.06135400
C	2.20619400	2.76834100	0.79781700
C	3.58525500	2.75525500	1.49324800
H	3.50424100	2.50680900	2.55701000
H	4.01444700	3.76525800	1.42296700
H	4.28168300	2.05541000	1.01539500
C	1.21592000	3.60289200	1.64079900
H	0.26608000	3.74254600	1.11716900
H	1.65754500	4.59766400	1.80475400
H	1.01653100	3.16114600	2.62132100
C	2.37825800	3.47147300	-0.56942200
H	3.16653500	3.00616000	-1.17051800
H	2.66759300	4.51380500	-0.36817100
H	1.45445000	3.47526000	-1.15299200
C	-1.65634700	-0.48360300	0.35632300
O	-1.36703400	0.57697600	1.10413000
N	-0.81489000	-0.37132700	-0.67099100
C	-0.45945100	-1.31723100	-1.66683600
C	-0.28462200	-0.90150000	-2.99553400
C	-0.25583600	-2.66251800	-1.30960800
C	0.09328700	-1.83633400	-3.96508200
H	-0.43534500	0.14020900	-3.25360500
C	0.11041700	-3.59105800	-2.29023900
H	-0.38435400	-2.97049500	-0.27542900
C	0.28978000	-3.18128500	-3.61953100
H	0.24012300	-1.50842900	-4.99190700
H	0.26958800	-4.63074500	-2.01016600
H	0.58909600	-3.90218800	-4.37751700
C	2.47274100	0.54247200	-1.46953900
N	3.62405500	0.00045200	-1.59120600
O	1.66951500	1.10790900	-2.21293700
C	4.25040900	-0.89861900	-0.74248400
C	3.67859400	-2.16670500	-0.48082900
C	5.53037800	-0.61007600	-0.21705300
C	4.34981200	-3.09587500	0.32213400
H	2.71939600	-2.40774100	-0.93535400
C	6.19409400	-1.54586000	0.58394200
H	5.98140600	0.35402900	-0.44319400
C	5.60724800	-2.79084800	0.86591400
H	3.89054500	-4.06377400	0.52080700
H	7.17366700	-1.30227500	0.99304400
H	6.12752800	-3.51499500	1.48980300

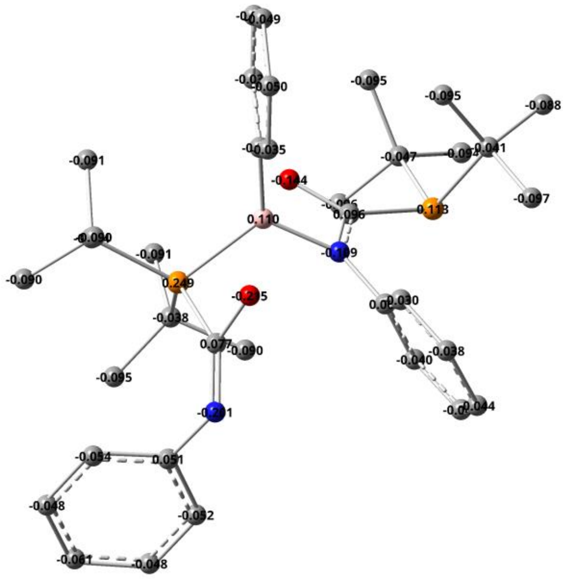

FIG. S107. OPTIMIZED STRUCTURE **1D_TS4**

B	0.17061300	0.96610100	0.28152900
C	0.80875100	2.31092600	0.86706100
P	3.08552700	-1.53352400	-0.83361700
C	1.64504700	3.12825400	0.06841900
H	1.80999900	2.85990900	-0.97193000
P	-1.69348300	0.81620700	-0.48420300
C	2.27546400	4.26922400	0.58250500
H	2.90990300	4.87239900	-0.06611800
C	2.09545800	4.62952200	1.92477500
H	2.58656900	5.51319100	2.32966500
C	1.27171300	3.83882600	2.73742300
H	1.11637400	4.10636100	3.78198800
C	0.63729800	2.70305100	2.21479000
H	-0.01736300	2.11726000	2.85361200
C	3.52917600	-0.98332300	-2.61042900
C	3.97700300	0.47799200	-2.80386300
H	3.21276200	1.17418500	-2.44562200
H	4.14236100	0.66259700	-3.87663200
H	4.91954900	0.68849600	-2.28530700
C	4.62310300	-1.95191500	-3.12447100
H	5.59046900	-1.79565500	-2.63813600
H	4.76110400	-1.78345800	-4.20263900
H	4.32773100	-2.99943900	-2.97961300
C	2.25581200	-1.25443500	-3.44830800
H	1.89547000	-2.28271000	-3.30760900
H	2.49761000	-1.12405800	-4.51357400
H	1.45266300	-0.55962300	-3.19283000
C	4.37609300	-0.83960100	0.42840500
C	4.19261500	-1.66433600	1.72390000
H	3.21048000	-1.50919600	2.17866000
H	4.95513500	-1.34930500	2.45217900
H	4.31713500	-2.73752200	1.53424300
C	4.21376600	0.66024600	0.74002300
H	4.27927000	1.28126400	-0.15913700
H	5.01307100	0.97156000	1.42953500
H	3.25536600	0.87226900	1.22701300

C	5.79737900	-1.11749900	-0.10497100
H	5.93531500	-2.17313900	-0.37229800
H	6.51487100	-0.87837300	0.69357400
H	6.04790100	-0.49634900	-0.97149900
C	-1.87808600	-0.22540100	-2.07047200
C	-1.18364900	-1.57177500	-1.75733600
H	-0.11061300	-1.45459800	-1.59649100
H	-1.32447300	-2.24329200	-2.61622400
H	-1.62598500	-2.04990500	-0.87503300
C	-3.33237000	-0.54652900	-2.47835600
H	-3.87481800	-1.07692200	-1.69331900
H	-3.28170300	-1.20511200	-3.35833200
H	-3.90205600	0.34000900	-2.76281600
C	-1.17264400	0.49710800	-3.23997400
H	-1.76003500	1.35777400	-3.58235400
H	-1.08807100	-0.20251200	-4.08465200
H	-0.16758900	0.83575600	-2.96886200
C	-2.46757100	2.55111500	-0.64960500
C	-3.80856400	2.51775800	-1.41129700
H	-3.65916600	2.34588600	-2.48326000
H	-4.28518800	3.50205900	-1.29824100
H	-4.49407500	1.75762000	-1.02318100
C	-1.50032800	3.49973300	-1.39428400
H	-0.57320200	3.65109300	-0.83656700
H	-1.99620200	4.47558100	-1.50177300
H	-1.25408800	3.13708100	-2.39828800
C	-2.71077100	3.09193300	0.77763500
H	-3.44191800	2.48261800	1.32184100
H	-3.10755900	4.11351200	0.68869100
H	-1.78890300	3.12387600	1.36456500
C	1.67864800	-0.36735200	-0.44773800
O	1.30153300	0.65346500	-1.15840800
N	0.87684000	-0.39684200	0.62781200
C	0.77796100	-1.38387700	1.64788000
C	0.78991700	-0.99901900	2.99763000
C	0.66703900	-2.73852000	1.29612700
C	0.70528300	-1.97723300	3.99304900
H	0.86712500	0.05290000	3.24989600
C	0.59773100	-3.71170700	2.30030900
H	0.64352600	-3.02188000	0.24734300
C	0.61486200	-3.33495200	3.65019000
H	0.70945300	-1.67716600	5.03902900
H	0.51697300	-4.76159300	2.02466400
H	0.54973800	-4.09178600	4.42942400
C	-2.15716100	0.07037600	1.23674500
N	-3.02333200	-0.81485100	1.59743900
O	-1.24863400	0.62998700	1.94611100
C	-4.11352100	-1.20826800	0.82967900
C	-4.24798600	-2.56190600	0.44026800
C	-5.15559500	-0.30735400	0.50745400
C	-5.35699200	-2.97920500	-0.30208400
H	-3.46400900	-3.26256900	0.72125700
C	-6.26595900	-0.73457100	-0.23211000
H	-5.08984800	0.71596800	0.87140400
C	-6.36926800	-2.06853900	-0.65245500
H	-5.43430500	-4.02110700	-0.61013800
H	-7.05468700	-0.02364400	-0.47586300

H	-7.23145800	-2.39931700	-1.22806900

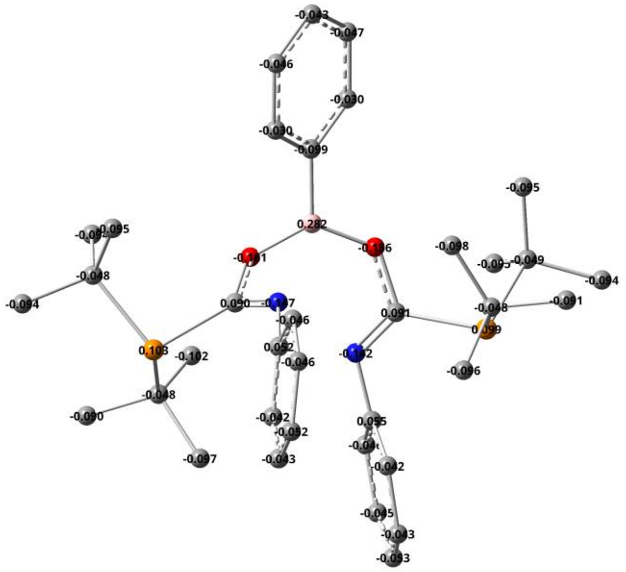

FIG. S108. OPTIMIZED STRUCTURE **1D_TS5**

P	3.29533000	-0.68689800	-0.02022000
P	-3.38517300	-0.50445900	0.03009000
O	1.25027600	0.93046800	-0.92749300
O	-1.23491200	0.88897200	-1.12102500
N	-0.94016300	0.58100200	1.04726900
N	0.76204400	-1.29816000	-1.13637500
C	-1.69997600	0.32830300	0.02613900
C	0.07478500	3.12099400	-0.82754100
C	-0.96667000	0.47548100	4.74164800
H	-0.81843200	1.16697900	5.56941700
C	4.31880800	0.27913700	-1.32901800
C	-0.90020000	0.94817900	3.42710900
H	-0.69062100	1.99513900	3.21727300
C	1.22764700	-5.46451400	-0.63951900
H	1.31891900	-6.54251200	-0.52219100
C	-1.10135900	0.06752700	2.34486200
C	-1.33015700	-1.30027000	2.60113300
H	-1.44068600	-1.98866400	1.76849500
C	-0.69624200	3.86821600	0.08855400
H	-1.34026100	3.34483300	0.79225100
C	-1.38509600	-1.76666700	3.91922800
H	-1.55957700	-2.82545800	4.10385400
C	0.93957900	3.82664500	-1.69309200
H	1.55121300	3.27478400	-2.40573500
C	-1.20884600	-0.88389800	4.99508700
H	-1.24759100	-1.25231600	6.01831200
C	-2.38366900	-1.50902700	-2.57398100
H	-2.81199000	-0.66986200	-3.13028500
H	-2.37913200	-2.38923600	-3.23652200
H	-1.34865200	-1.26491900	-2.31701900
C	4.72137000	0.43682000	2.09369300
H	5.26313300	-0.51796200	2.05249200
H	4.73311300	0.78804800	3.13612300

H	5.26489000	1.17273700	1.48969200
C	0.99061400	-2.67540900	-0.94452400
C	1.12532800	-3.24026600	0.33889500
H	1.11550700	-2.59060100	1.20925300
C	1.24085700	-4.62719700	0.48543200
H	1.34199900	-5.05218500	1.48279800
C	0.94604800	-3.51841000	-2.07210700
H	0.80928200	-3.07364100	-3.05553100
C	5.77117900	-0.23888500	-1.18625300
H	6.21435900	0.03619600	-0.22221000
H	6.39196100	0.20418200	-1.97963800
H	5.81392000	-1.33093800	-1.28335800
C	-4.27878900	1.33661600	-2.11526800
H	-3.23556100	1.56819800	-2.35235400
H	-4.88819300	2.23010400	-2.32286200
H	-4.62529500	0.53990700	-2.78376800
C	1.57430200	-0.39856100	-0.72742100
C	-5.91820100	0.57517000	-0.34352400
H	-6.25580400	-0.28046700	-0.93804000
H	-6.56135900	1.43194600	-0.59458600
H	-6.06354200	0.33556300	0.71751100
B	0.01422500	1.55747900	-0.87076800
C	0.24004800	5.94809100	-0.74342900
H	0.30310800	7.03460300	-0.71078100
C	-4.44493600	0.95226400	-0.63438200
C	1.01412200	5.22532700	-1.66282400
H	1.67809600	5.74921100	-2.34876400
C	1.08307800	-4.90206900	-1.91777000
H	1.06425200	-5.54408700	-2.79693100
C	-2.52365000	-3.03407200	-0.58205800
H	-1.51126400	-2.77275200	-0.25724000
H	-2.43013000	-3.88846500	-1.26825600
H	-3.10598400	-3.35234400	0.29255000
C	-0.61105000	5.26514500	0.13801000
H	-1.20427800	5.81985600	0.86347700
C	4.30738900	1.81595200	-1.25885200
H	3.28960600	2.21238700	-1.30590600
H	4.87785500	2.21740900	-2.11141900
H	4.78340200	2.18555600	-0.34306500
C	-4.08450700	2.16737100	0.25040700
H	-4.07596500	1.90658200	1.31780000
H	-4.83263200	2.95928500	0.09846400
H	-3.10666100	2.57657900	-0.02128700
C	3.78294300	-0.17865800	-2.70695200
H	3.70129700	-1.27277600	-2.76530400
H	4.47974400	0.15179500	-3.49147500
H	2.79912100	0.25441500	-2.92312400
C	3.25323000	0.28147700	1.63543300
C	2.54020700	1.64706800	1.67326200
H	2.93798700	2.35648100	0.94302100
H	2.67443800	2.07872200	2.67770300
H	1.46624600	1.53877400	1.49664500
C	2.53669400	-0.66855000	2.62447200
H	1.48058200	-0.79738700	2.36110000
H	2.56587200	-0.23100200	3.63325600
H	3.01784500	-1.65452200	2.65908000
C	-3.20495100	-1.85626000	-1.31771900

C	-4.62748200	-2.30247200	-1.72239000
H	-5.25437600	-2.51825100	-0.84645500
H	-4.55052900	-3.22462500	-2.31716400
H	-5.13346600	-1.55135100	-2.34007500

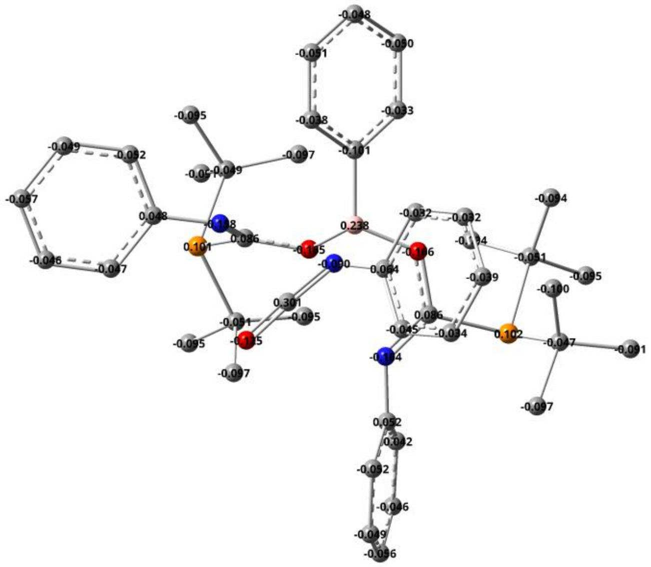

FIG. S109. OPTIMIZED STRUCTURE **1D_TS6**

P	3.47287500	0.89542500	1.09194300
P	-3.26980100	1.67576000	0.62284000
O	1.25041700	-0.70693900	0.62167600
O	-0.92441000	0.20675900	0.33506200
O	-0.98711900	-0.09192900	-3.21368600
N	-2.64889100	-0.62716600	-0.87523000
N	0.03478000	-1.52653800	-1.55012300
N	1.49332900	0.95825800	-0.92646100
C	-2.22266900	0.26874400	-0.05309000
C	-0.69597000	-2.29503000	1.01554000
C	-5.58579500	0.28897100	-2.98268600
H	-5.86782700	1.04233900	-3.71651000
C	3.15538900	-2.69036600	-3.27620700
H	3.85201700	-2.33987800	-4.03500000
C	3.12745600	0.33707500	2.88553800
C	-4.32134100	0.34940700	-2.38688300
H	-3.61594200	1.13317100	-2.65215200
C	3.35297900	-3.92880400	-2.65001200
H	4.20217900	-4.55052000	-2.92567400
C	2.83532400	4.59062000	-2.63143100
H	3.15852400	5.52464000	-3.08673400
C	-3.94695100	-0.61400500	-1.42976500
C	1.36100100	-3.56649100	-1.29615500
H	0.66216100	-3.89067600	-0.53185000
C	-4.84194300	-1.64985900	-1.10051100
H	-4.53845600	-2.39666900	-0.36981900
C	2.06866800	-1.88463800	-2.91678200
H	1.92401500	-0.91048600	-3.37734500
C	-1.62504500	-3.17959900	0.42519100
H	-1.93399600	-3.00643200	-0.60286200
C	-6.10994600	-1.69456700	-1.69213500
H	-6.80200300	-2.48888800	-1.41666400

C	-0.31126100	-2.55323000	2.34916300
H	0.40105500	-1.89073900	2.83559600
C	-6.48859400	-0.72750200	-2.63554700
H	-7.47301900	-0.76798400	-3.09771800
C	-0.52245700	-0.78444500	-2.38942800
C	-0.87769400	3.17623600	1.51315000
H	-1.22434100	3.27498400	2.54908100
H	-0.24761300	4.05128700	1.29152400
H	-0.26445200	2.27529100	1.42591600
C	6.06164500	-0.16851800	1.01163200
H	6.36466000	0.87813800	1.14673000
H	6.84900900	-0.68077400	0.43863300
H	6.00590000	-0.64667500	1.99722900
C	1.99896000	2.16580100	-1.44535200
C	1.17231100	-2.33480400	-1.93609600
C	2.24688900	2.24102600	-2.83089600
H	2.09950800	1.35228600	-3.44075300
C	2.67819500	3.43730600	-3.41433100
H	2.87749000	3.47103200	-4.48419400
C	2.13882900	3.33407300	-0.66633100
H	1.91331300	3.29363800	0.39475600
C	4.19078600	1.05073700	3.75784400
H	5.20585900	0.69477200	3.54694300
H	3.97888700	0.85105300	4.81918000
H	4.17014900	2.13667300	3.60080400
C	-2.23800100	0.53792800	3.16476600
H	-1.81671200	-0.34001200	2.66722900
H	-2.53365100	0.23422000	4.18155100
H	-1.45444600	1.29479300	3.25334500
C	1.92103900	0.38770100	0.14778000
C	-4.12692500	2.21321300	3.22284000
H	-3.41136100	3.02417900	3.40720800
H	-4.46156200	1.83502400	4.20040200
H	-4.99917800	2.62989700	2.70168800
B	-0.12346200	-0.99402800	0.29938700
C	-1.75602400	-4.50100300	2.46151600
H	-2.16325700	-5.34612100	3.01450700
C	-3.48337500	1.05875200	2.42485000
C	-0.83269400	-3.63840600	3.06781200
H	-0.51818100	-3.80843800	4.09672000
C	2.55229000	4.53152700	-1.25798400
H	2.65215000	5.42377400	-0.64157500
C	-1.51043300	3.12382700	-0.92697300
H	-0.83307800	2.27949500	-1.09150800
H	-0.94553700	4.04746700	-1.11823000
H	-2.32997200	3.07156700	-1.65723800
C	-2.14792000	-4.26900400	1.13423200
H	-2.86001400	-4.93719300	0.65114200
C	3.15275400	-1.17192900	3.18513500
H	2.50476900	-1.72979100	2.50395600
H	2.80795500	-1.34232400	4.21743600
H	4.17039700	-1.57449800	3.11097200
C	-4.49866700	-0.10365200	2.30154600
H	-5.41373800	0.20831200	1.78303100
H	-4.76860100	-0.45685400	3.30826900
H	-4.06436300	-0.95068500	1.75535500
C	1.74804200	0.93231300	3.25383200

H	1.70593600	2.00685500	3.02866100
H	1.57827900	0.80954500	4.33403400
H	0.93227900	0.43524600	2.72112100
C	4.72667400	-0.28271000	0.24477100
C	4.31767400	-1.75985200	0.11302000
H	4.25668500	-2.26399800	1.08112700
H	5.07091900	-2.28490700	-0.49389100
H	3.35189800	-1.86594200	-0.38820300
C	2.45835200	-4.35758000	-1.65940700
H	2.60943300	-5.31382800	-1.16269300
C	4.91232300	0.30640000	-1.17370900
H	4.00057500	0.19396600	-1.77119100
H	5.71920200	-0.24006400	-1.68524900
H	5.17460700	1.37111100	-1.14369100
C	-2.05230400	3.14526200	0.52248200
C	-2.91350200	4.41948800	0.69996300
H	-3.74431400	4.44095500	-0.01672000
H	-2.28511200	5.30610000	0.52682800
H	-3.33194800	4.49744100	1.71072300

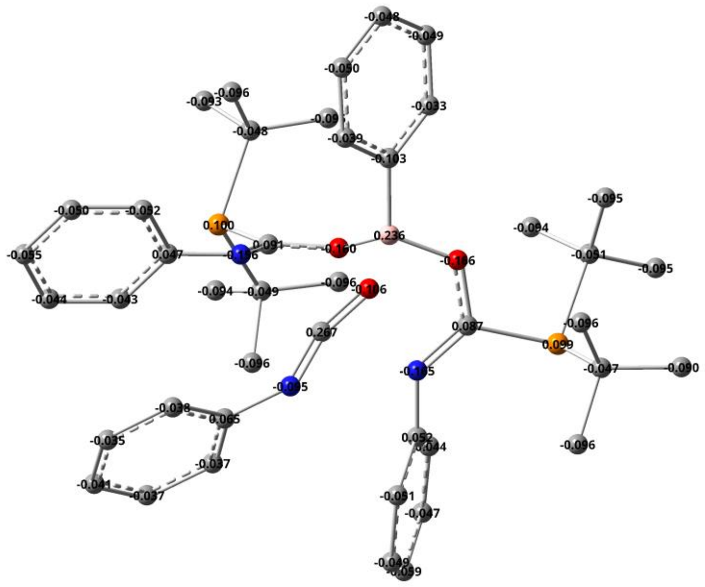

FIG. S110. OPTIMIZED STRUCTURE **1D_TS7**

P	4.20229300	0.58719000	0.13498400
P	-2.19199500	-1.18345500	2.08920700
O	1.99034400	-0.79551100	-0.75995200
O	-0.14701700	-0.86438200	0.26904600
O	0.08992900	-0.03246000	-2.21605500
N	-2.17986300	-0.53341200	-0.67477300
N	-1.22619300	1.94351700	-1.97075300
N	1.49165200	1.34647700	-0.14133400
C	-1.48703100	-0.81878500	0.38121700
C	0.36519600	-2.57940600	-1.58027300
C	-5.67685500	0.60968700	0.01552700
H	-6.20499800	1.31407600	0.65576200
C	-4.67283000	3.28396100	-2.05927700
H	-5.57353900	3.19949500	-2.66316900
C	4.73010900	-1.19758000	0.57223600
C	-4.29016500	0.48197100	0.13696700
H	-3.74105800	1.09412100	0.84560800

C	-4.66625000	4.08801600	-0.90939300
H	-5.56102400	4.63825100	-0.62551500
C	1.87776900	5.09321900	1.77297300
H	1.94584900	6.06099300	2.26598100
C	-3.58819600	-0.41664800	-0.68933000
C	-2.35226900	3.47302900	-0.48161900
H	-1.44321300	3.53330800	0.11074200
C	-4.29482600	-1.16726500	-1.64887800
H	-3.74204300	-1.85309400	-2.28747700
C	-3.53085900	2.56689400	-2.42681000
H	-3.52763300	1.92029700	-3.30022600
C	-0.65691100	-2.85000300	-2.51354800
H	-1.26089200	-2.02781700	-2.89023800
C	-5.68474200	-1.04084900	-1.75909200
H	-6.22145300	-1.63651600	-2.49579400
C	1.13166900	-3.67042100	-1.11672300
H	1.92904300	-3.49543000	-0.39597200
C	-6.38290600	-0.15155700	-0.92807500
H	-7.46261100	-0.04851500	-1.01912700
C	-0.71161100	0.85493500	-1.96713400
C	0.41101300	-0.35885200	3.29734700
H	0.58523300	-1.27988600	3.86258300
H	0.89253100	0.46341500	3.84898900
H	0.88911900	-0.44607800	2.31890400
C	6.31472000	0.89545800	-1.69474100
H	6.80228800	1.41394400	-0.85842700
H	6.68439900	1.33994300	-2.63083300
H	6.62454500	-0.15615700	-1.68238800
C	1.70374000	2.58009700	0.49600900
C	-2.38256400	2.65500700	-1.62262400
C	1.30790100	3.75540700	-0.17676000
H	0.93015100	3.67304500	-1.19404800
C	1.40876100	5.00097700	0.45357600
H	1.11182900	5.89946600	-0.08547500
C	2.16610100	2.67625800	1.82592000
H	2.44602700	1.76979900	2.35538700
C	6.13895600	-1.07384200	1.20490000
H	6.89077000	-0.74512400	0.47815500
H	6.45015200	-2.05770200	1.58739900
H	6.13863500	-0.36345000	2.04156800
C	-0.21885900	-3.40730200	2.29469900
H	0.36399700	-2.89949800	1.52256200
H	-0.11825400	-4.49223200	2.13826700
H	0.21210500	-3.17178900	3.27355800
C	2.36997700	0.40957800	-0.24619500
C	-2.43892200	-3.57318200	3.47721300
H	-2.03398200	-3.13952100	4.40065900
H	-2.30408100	-4.66386900	3.53352500
H	-3.51476900	-3.36002600	3.43854700
B	0.60821700	-1.14820200	-0.94055800
C	-0.14167700	-5.22442200	-2.47882900
H	-0.33842800	-6.23894100	-2.82222800
C	-1.71059400	-3.03313800	2.22518800
C	0.88583300	-4.97774600	-1.55705000
H	1.49278100	-5.80066200	-1.18157500
C	2.24911800	3.92313900	2.45390000
H	2.60635700	3.98050900	3.48115500

C	-1.25073800	1.36240300	2.53956500
H	-0.76473700	1.41956100	1.55874200
H	-0.77239300	2.10823400	3.19089500
H	-2.30842500	1.63778600	2.43036800
C	-0.91039900	-4.15458300	-2.95933000
H	-1.70675300	-4.33678900	-3.68008800
C	4.76638200	-2.24405100	-0.55618600
H	3.80270900	-2.31974300	-1.06612500
H	5.01243000	-3.22746300	-0.12508300
H	5.54066500	-2.01074700	-1.29703600
C	-2.33802900	-3.67672200	0.96308400
H	-3.38234300	-3.36461300	0.82519400
H	-2.31801100	-4.77115200	1.07054800
H	-1.77575700	-3.42059600	0.05725600
C	3.75492100	-1.65602800	1.68360200
H	3.68245400	-0.90904300	2.48651900
H	4.12721700	-2.59121600	2.12809200
H	2.75068100	-1.84016500	1.28967700
C	4.77940100	1.04687900	-1.63933200
C	4.12493900	0.27069100	-2.79845400
H	4.31284700	-0.80506600	-2.74156400
H	4.53910900	0.64010100	-3.74968900
H	3.04037800	0.42439600	-2.82128200
C	-3.50656200	4.18057000	-0.12616500
H	-3.49551500	4.80488500	0.76521100
C	4.42577900	2.54592500	-1.78593900
H	3.34162500	2.70555700	-1.76531900
H	4.80223300	2.91143500	-2.75348400
H	4.87440600	3.14863700	-0.98667000
C	-1.08993800	-0.04358400	3.16685300
C	-1.73564900	-0.03940600	4.57222700
H	-2.80315300	0.21110300	4.52377000
H	-1.23203200	0.70942800	5.20201600
H	-1.63366600	-1.01353100	5.06732000

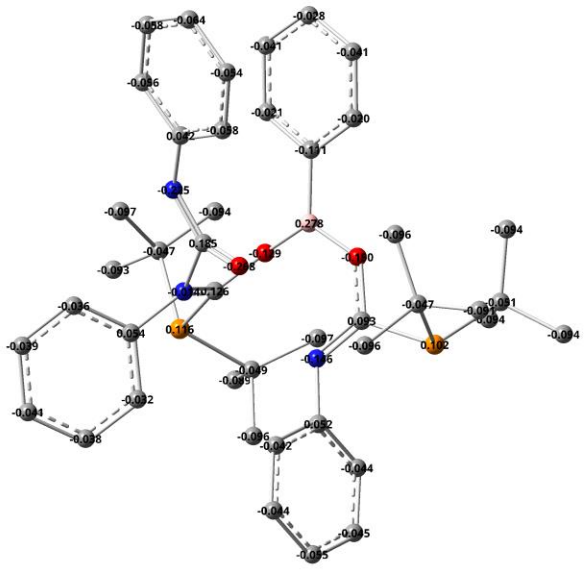

FIG. S111. OPTIMIZED STRUCTURE **1D_TS8**

P	3.80715600	0.88899500	-0.07121800
P	-1.76884800	-2.89276100	-1.08494900

O	1.15409500	1.11019000	-0.86061400
O	-0.84118000	-0.28085800	-1.20270700
O	-0.18440000	0.84913800	1.87287000
N	-1.37634800	-0.97167700	0.89960800
N	-2.46843900	1.05524000	1.17169400
N	1.65237100	-0.90911100	0.11332900
C	-1.19421400	-1.25138600	-0.36973900
C	-1.00712200	2.25476100	-1.45048000
C	-1.17690700	-3.99632500	3.07734900
H	-0.53117100	-4.85953000	3.22590400
C	-2.19974600	4.67473100	2.17474900
H	-1.46179600	5.39655300	2.52435700
C	4.12369400	1.66537600	-1.79234300
C	-0.86189300	-3.06654800	2.08084800
H	0.02518400	-3.18322900	1.46579400
C	-3.52482800	5.08038500	1.95773400
H	-3.82220100	6.11206100	2.13873800
C	4.11774500	-3.72332300	2.07861000
H	4.73337500	-4.46207900	2.58793400
C	-1.69518900	-1.95502200	1.89475900
C	-4.07750600	2.81714100	1.27115600
H	-4.79177700	2.07927200	0.90929300
C	-2.81901900	-1.74276900	2.70677200
H	-3.42958800	-0.85873600	2.53940500
C	-1.80364200	3.35147700	1.94644600
H	-0.77769700	3.04236200	2.11905800
C	-2.36822100	2.18524200	-1.82379700
H	-2.85523300	1.21629800	-1.88340000
C	-3.12251300	-2.68072600	3.70036500
H	-3.99697900	-2.52901100	4.32997200
C	-0.41822000	3.53387500	-1.33049100
H	0.61819300	3.61176900	-1.01582600
C	-2.30747800	-3.80818900	3.88594200
H	-2.54733600	-4.53143200	4.66296100
C	-1.28953800	0.53768800	1.39766100
C	0.41024000	-2.26392400	-3.00472700
H	-0.24955300	-1.63170700	-3.60672500
H	1.14619700	-2.72925900	-3.67866800
H	0.95306300	-1.63053200	-2.29683500
C	4.86046400	3.01393500	1.38641800
H	5.72698100	2.34512200	1.47629800
H	4.82396800	3.64670000	2.28546300
H	5.01603500	3.67310700	0.52287200
C	2.53281100	-1.80910300	0.76346800
C	-2.74352600	2.39856500	1.48368000
C	2.36288800	-2.03806100	2.14117200
H	1.61428200	-1.46247300	2.68018700
C	3.16257500	-2.98454000	2.79305200
H	3.03482000	-3.14459100	3.86246400
C	3.48614000	-2.55228300	0.04281300
H	3.60005900	-2.37513900	-1.02449400
C	5.65911500	1.84685400	-1.88470100
H	6.02633100	2.59714300	-1.17470600
H	5.92405900	2.18505000	-2.89779900
H	6.18403800	0.90385900	-1.68635700
C	-2.96086800	-1.34788300	-3.31412700
H	-2.30543500	-0.50066100	-3.09465900

H	-3.90522500	-0.95348000	-3.71978300
H	-2.49276900	-1.95349400	-4.09912700
C	2.05352800	0.24428800	-0.26752500
C	-4.12930900	-3.41032400	-2.45082300
H	-3.63660400	-4.04877100	-3.19218300
H	-5.07194100	-3.05248900	-2.89076800
H	-4.37130300	-4.02173900	-1.57247500
B	-0.20721200	0.98648900	-1.08171000
C	-2.50495100	4.60212300	-1.94328500
H	-3.08810500	5.50559500	-2.11294700
C	-3.26702000	-2.18479600	-2.06011500
C	-1.15685600	4.69617000	-1.57139800
H	-0.69072000	5.67238200	-1.45029100
C	4.27272200	-3.50376200	0.70134800
H	5.01197300	-4.07031600	0.13742600
C	0.63849300	-4.14124300	-1.35099000
H	1.05416300	-3.48398200	-0.58011800
H	1.47976300	-4.51677800	-1.95231500
H	0.15532100	-4.99523900	-0.85968000
C	-3.10923600	3.34236500	-2.07315900
H	-4.16131700	3.26746100	-2.34169700
C	3.42979500	2.99389900	-2.13507700
H	2.34212000	2.90644000	-2.06652300
H	3.68681700	3.27841800	-3.16744700
H	3.76473300	3.80493900	-1.47755000
C	-4.07178500	-1.35334400	-1.03035200
H	-4.29480800	-1.94814100	-0.13415900
H	-5.02861200	-1.05842200	-1.48613000
H	-3.55661700	-0.44261400	-0.70014400
C	3.70693000	0.58438100	-2.81852600
H	4.15238400	-0.39065200	-2.57714300
H	4.06055600	0.87787900	-3.81799200
H	2.61770500	0.46979200	-2.86651500
C	3.53744900	2.22518000	1.27334900
C	2.35484500	3.19589400	1.10885600
H	2.44498500	3.81037500	0.20786900
H	2.33832800	3.87538200	1.97546300
H	1.40139500	2.66038800	1.08739600
C	-4.46438900	4.13814700	1.50839300
H	-5.49812900	4.43654700	1.33688000
C	3.31526500	1.40646100	2.56856700
H	2.35358000	0.88186900	2.53759600
H	3.29222300	2.09532900	3.42637000
H	4.11666200	0.67472400	2.73092400
C	-0.35032200	-3.38284900	-2.26823600
C	-0.93035000	-4.38795100	-3.28859700
H	-1.51431600	-5.17761000	-2.79676900
H	-0.09660200	-4.86923000	-3.82019600
H	-1.56353900	-3.89789800	-4.03708800

Formation of 2b

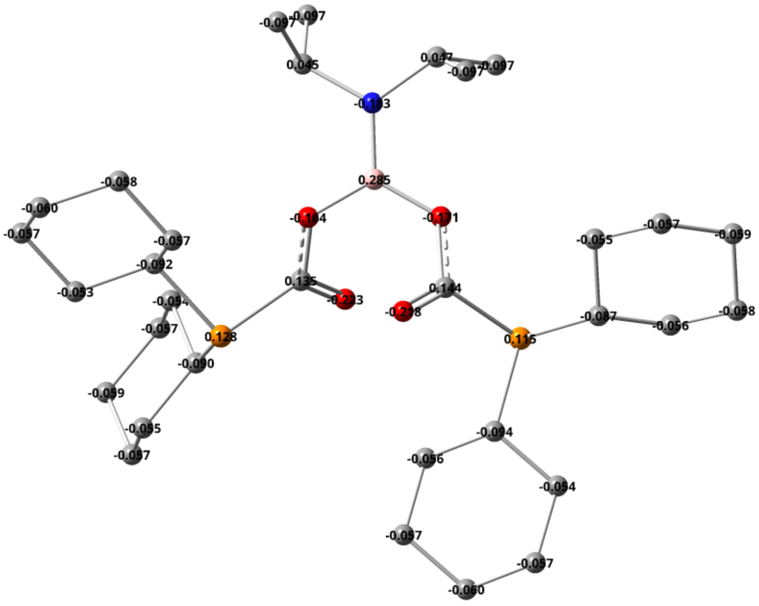

FIG. S112. OPTIMIZED STRUCTURE **2B**

P	-2.90562800	-1.02116100	0.54445300
P	2.48737500	-1.01179000	-0.59513000
O	0.99858400	1.16433500	0.07619200
O	-1.43854000	1.24399500	0.37795600
N	-0.07214000	3.25256500	0.66984400
C	1.21406400	-0.13903800	0.44392600
C	-1.69143600	0.10205900	-0.35620700
C	1.26872500	3.88094900	0.79276200
H	1.98630700	3.07086500	0.62844600
C	1.49915700	4.94018500	-0.30008200
H	1.36547100	4.49912300	-1.29497300
H	2.52069900	5.33577000	-0.22923700
H	0.80757500	5.78747000	-0.19947500
C	1.50415300	4.44041200	2.20733700
H	0.81733500	5.26524000	2.44005300
H	2.52775500	4.82816500	2.29076500
H	1.36643800	3.65332700	2.95830300
C	-1.24555600	4.14921600	0.85609000
H	-0.83104900	5.14475900	1.06077800
C	-2.08729700	3.74167300	2.07828200
H	-2.52746000	2.74939900	1.93642900
H	-2.89761400	4.46644900	2.23413700
H	-1.46420900	3.71673200	2.98037200
C	-2.08101700	4.25408200	-0.43109400
H	-1.45654500	4.59042500	-1.26747000
H	-2.89971900	4.97328100	-0.29455900
H	-2.51497300	3.28347500	-0.69060700
C	-2.67569200	-2.51791200	-0.57367100
H	-2.67697800	-2.15721500	-1.61486400
C	-1.31057500	-3.18192900	-0.28299400
H	-0.50000200	-2.46569700	-0.45571200
H	-1.26903000	-3.46846000	0.77985300
C	-1.10063000	-4.42920500	-1.16165400
H	-1.05082900	-4.11988100	-2.21780300
H	-0.13338600	-4.89309200	-0.92001000

C	-2.24402600	-5.44247000	-0.97941100
H	-2.09752000	-6.31192100	-1.63633900
H	-2.23352900	-5.81808200	0.05686300
C	-3.60599900	-4.78453400	-1.26340200
H	-4.42299300	-5.50132400	-1.09584700
H	-3.65061500	-4.48889100	-2.32400500
C	-3.82291100	-3.53465600	-0.38671200
H	-4.79036400	-3.07751000	-0.63744700
H	-3.87382700	-3.83742400	0.67205300
C	-4.53820300	-0.31705800	-0.14369000
H	-4.71925900	-0.82510400	-1.10580600
C	-5.67457000	-0.67638400	0.84162200
H	-5.70154900	-1.76025500	1.01894000
H	-5.45573200	-0.20554000	1.81286800
C	-7.04155000	-0.18502700	0.32924000
H	-7.28925700	-0.71034300	-0.60749700
H	-7.82583500	-0.44060600	1.05597000
C	-7.01909300	1.33187100	0.07108200
H	-6.86000500	1.85766200	1.02652300
H	-7.98822900	1.66931100	-0.32284200
C	-5.88834400	1.70179400	-0.90470800
H	-6.09724700	1.25039600	-1.88801600
H	-5.85034600	2.79036000	-1.05532600
C	-4.51830400	1.20208400	-0.40545800
H	-4.24947100	1.73148600	0.52102800
H	-3.74801000	1.44443700	-1.14924400
C	3.27989800	-1.92911200	0.84883100
H	2.38835700	-2.36205400	1.33236200
C	3.99303000	-1.04821900	1.89553900
H	3.31349400	-0.25894900	2.24496200
H	4.86234700	-0.55134700	1.43834900
C	4.47268400	-1.89836400	3.08924300
H	5.00763500	-1.26288600	3.80973500
H	3.59358900	-2.30707400	3.61195500
C	5.37204700	-3.05789200	2.62374000
H	6.28980400	-2.64600100	2.17245600
H	5.68511900	-3.66599300	3.48422700
C	4.64846600	-3.93493600	1.58544400
H	3.77447500	-4.40982900	2.05957200
H	5.30694100	-4.74553000	1.24186600
C	4.17357400	-3.09863900	0.38084700
H	3.61984000	-3.72813900	-0.32946000
H	5.05376800	-2.71257700	-0.15480100
C	3.64091700	0.41130200	-1.05392900
H	3.73484600	1.09078000	-0.19202900
C	5.04637800	-0.10781800	-1.43248800
H	5.50219300	-0.64769000	-0.59347600
H	4.95458800	-0.82506300	-2.26415500
C	5.96875700	1.05414000	-1.85368000
H	6.12323800	1.71726500	-0.98690900
H	6.95624700	0.65978800	-2.13326000
C	5.36622600	1.86748200	-3.01256300
H	6.02089200	2.71401700	-3.26428500
H	5.30783800	1.22950000	-3.90910200
C	3.95361300	2.36264600	-2.65644500
H	3.50987700	2.90064500	-3.50639600
H	4.01732900	3.08054000	-1.82233500

C	3.03996200	1.19217200	-2.24733800
H	2.92768000	0.50089700	-3.09764900
H	2.03924400	1.55776700	-1.99589700
B	-0.18402600	1.88204000	0.37758100
O	-1.13793800	-0.11728800	-1.42156400
O	0.57975300	-0.68254000	1.33998600

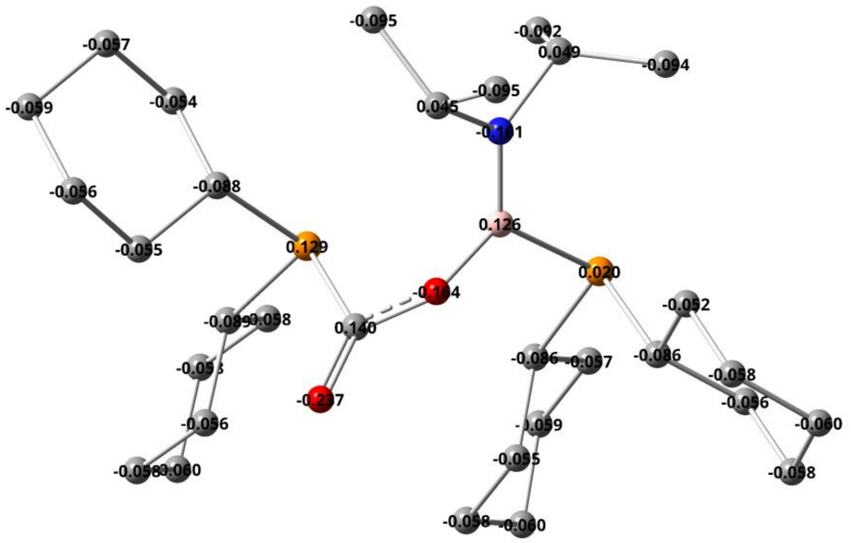

FIG. S113. OPTIMIZED STRUCTURE **2B_I1A**

P	-1.77136000	-0.01177600	0.27103800
P	2.31946600	0.13492500	0.94486600
N	0.90972400	-2.47897500	0.68464600
C	3.52555100	0.13106100	-0.52782100
H	2.99357200	0.52833600	-1.40529800
C	4.73654500	1.03813800	-0.21733900
H	4.40508100	2.05216000	0.04479900
H	5.25601200	0.63953000	0.66870700
C	-2.96388900	-1.38711800	-0.26322500
H	-2.31419600	-2.25447200	-0.45047600
C	1.44301800	1.81843300	0.75011000
H	0.38256800	1.60501100	0.96837500
C	-2.87033400	1.52031000	0.09337600
H	-3.88859300	1.12118200	0.24804400
C	1.96562200	2.76495000	1.85899900
H	1.84759500	2.28300900	2.83903100
H	3.04765300	2.91799500	1.71640800
C	0.18839700	-3.51514800	-0.10667000
H	-0.25416000	-2.98051600	-0.95305100
C	-3.89297800	-1.73724700	0.92136300
H	-3.29693900	-1.94672600	1.82090100
H	-4.52598400	-0.86569600	1.15565000
C	1.52732200	2.50215900	-0.63091100
H	2.58265100	2.67210500	-0.89043400
H	1.10844800	1.85776200	-1.41300600
C	-2.57063900	2.47341100	1.27518300
H	-2.61223200	1.92022800	2.22418600
H	-1.54234800	2.85118900	1.17843400
C	1.44833300	-2.96857900	1.99173700

H	1.08999000	-4.00072000	2.07924300
C	-3.78181400	-1.12384200	-1.54279200
H	-4.43266200	-0.24976700	-1.38720300
H	-3.11524700	-0.88656300	-2.38056300
C	-3.84777100	3.46818400	-1.20306500
H	-3.81268000	4.00930200	-2.15938200
H	-4.87491300	3.08216800	-1.09274400
C	4.02114000	-1.28425800	-0.89059000
H	4.54202400	-1.72341600	-0.02658800
H	3.17068800	-1.94093100	-1.11923500
C	2.98356700	-3.02756700	2.04594200
H	3.38413700	-3.57311800	1.18372500
H	3.29425200	-3.54758500	2.96253700
H	3.41190000	-2.01894500	2.06027600
C	1.36146200	4.79822600	0.45877700
H	0.82258400	5.75675100	0.45217500
H	2.41910800	5.02365900	0.24491100
C	-2.86204300	2.28381600	-1.24749500
H	-1.84966200	2.65801600	-1.44794500
H	-3.10512600	1.61185700	-2.07612900
C	5.71127600	1.09659200	-1.40988900
H	5.20234700	1.56608100	-2.26728900
H	6.57222900	1.73374000	-1.16056800
C	0.81043000	3.86724700	-0.63423600
H	0.90930800	4.33264600	-1.62562400
H	-0.26543900	3.71422900	-0.46349400
C	-4.79607400	-2.93956900	0.58142100
H	-4.16472600	-3.83270500	0.44964700
H	-5.47186400	-3.14704800	1.42330800
C	-5.59945200	-2.69339500	-0.70795200
H	-6.30028900	-1.85867400	-0.54387500
H	-6.20652400	-3.57665300	-0.95167600
C	-3.53846200	4.42152200	-0.03557800
H	-4.26314300	5.24777400	-0.01159500
H	-2.54448000	4.87139700	-0.19023700
C	-0.94659500	-4.19270900	0.68788300
H	-0.56661700	-4.93399700	1.40256500
H	-1.61138000	-4.72182800	-0.00695800
H	-1.53833300	-3.45563000	1.24137500
C	4.98551700	-1.23622500	-2.09204300
H	5.33381300	-2.25146800	-2.33254000
H	4.43968600	-0.86676700	-2.97484700
C	1.25590600	4.13163800	1.84143800
H	0.19282400	3.99052300	2.09462500
H	1.68245900	4.78380500	2.61743000
C	0.85382400	-2.17496200	3.16745900
H	1.22945300	-1.14516000	3.16413800
H	1.13912400	-2.64520200	4.11811300
H	-0.24025600	-2.14776000	3.09976800
C	-3.54680400	3.66335900	1.30338400
H	-4.56496200	3.29011600	1.50220000
H	-3.28699700	4.33826200	2.13160200
C	-4.66536600	-2.34233400	-1.87951400
H	-5.24885900	-2.13730200	-2.78817100
H	-4.01934300	-3.20688300	-2.10316100
C	1.17116400	-4.55737700	-0.67009800
H	1.93664500	-4.07270300	-1.28680200

H	0.63239500	-5.28630900	-1.28976600
H	1.67157800	-5.10819300	0.13769500
C	6.18432900	-0.31253100	-1.80999700
H	6.78133800	-0.73860500	-0.98716700
H	6.84258300	-0.26074000	-2.68906400
B	1.04171200	-1.15909200	0.20076900
C	-0.68264200	-0.10430000	-1.28547000
O	-0.91659600	0.34745600	-2.39461100
O	0.43465200	-0.85583000	-1.07091800

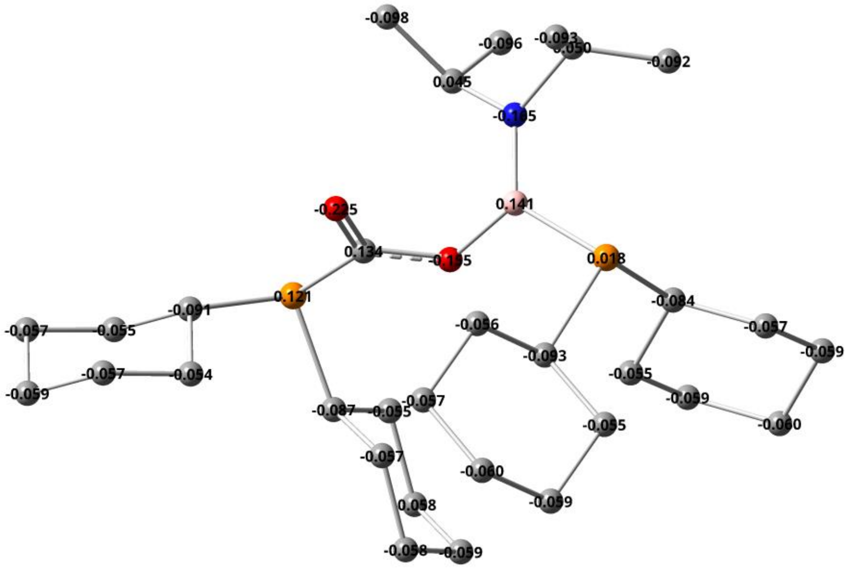

FIG. S114. OPTIMIZED STRUCTURE **2B_I1B**

P	-2.42880900	0.66591300	-1.26672400
P	2.55474800	0.33761100	1.19883400
N	1.49363100	2.76632100	-0.17567600
C	1.38413800	-0.97153900	1.87973000
H	0.72627800	-1.31620300	1.06973500
C	2.12216800	-2.19408400	2.46077400
H	2.72961900	-2.68157900	1.68641100
H	2.81725500	-1.85773900	3.24671700
C	-3.97997400	0.74389100	-0.21480200
H	-3.92798600	1.76110200	0.20653600
C	3.36958200	-0.47659400	-0.32489900
H	3.59363600	0.37976700	-0.98331200
C	-2.15197200	-1.16571900	-1.62931100
H	-3.18088400	-1.53619500	-1.78893900
C	4.72009300	-1.13218300	0.04204700
H	5.35075700	-0.40712300	0.57459100
H	4.54693400	-1.96899800	0.73581700
C	0.59497400	3.46503700	-1.14092500
H	-0.13138500	2.72012600	-1.47758500
C	-5.22972200	0.65480400	-1.12064000
H	-5.15633200	1.38403000	-1.93952500
H	-5.27446800	-0.34483900	-1.58450100
C	2.48060000	-1.45112900	-1.12235000
H	2.21279300	-2.30920600	-0.48614900
H	1.54116500	-0.95913800	-1.40318800

C	-1.37605300	-1.32856900	-2.95722000
H	-1.85942500	-0.74214100	-3.75042200
H	-0.36381600	-0.91607600	-2.82578400
C	2.58450700	3.62804600	0.37638800
H	2.43307400	4.61231600	-0.08268600
C	-4.08666700	-0.25160000	0.95766200
H	-4.11696600	-1.27853700	0.56065700
H	-3.20452500	-0.16773500	1.60450100
C	-1.41701500	-3.49642700	-0.92865400
H	-0.92928100	-4.07244200	-0.12888400
H	-2.43804300	-3.89791900	-1.03543000
C	0.50234100	-0.31524900	2.96690100
H	1.15365700	0.06403100	3.77119000
H	-0.03586800	0.54924600	2.55624600
C	2.44754700	3.80860800	1.89755800
H	1.44154300	4.16263300	2.15115800
H	3.18062200	4.54584900	2.25211500
H	2.62972100	2.86136100	2.41753700
C	4.55214500	-2.62812500	-2.00992200
H	5.07227100	-2.96924900	-2.91643200
H	4.35709100	-3.52322800	-1.39673200
C	-1.48986700	-2.01190300	-0.52395400
H	-0.47203100	-1.63288300	-0.36955000
H	-2.02125300	-1.90116400	0.42764300
C	1.12336000	-3.21097500	3.04907400
H	0.49163800	-3.60234500	2.23459400
H	1.66578600	-4.06879900	3.47253100
C	3.21102100	-1.96878000	-2.37753700
H	2.57042300	-2.67944500	-2.92030400
H	3.39709800	-1.12274800	-3.05927400
C	-6.51910900	0.88851500	-0.30894500
H	-6.51782600	1.92308500	0.06990900
H	-7.39636500	0.78848000	-0.96398200
C	-6.62146300	-0.08535200	0.87906300
H	-6.72027800	-1.11412000	0.49533500
H	-7.52692500	0.12447700	1.46581400
C	-0.65517300	-3.67018100	-2.25424800
H	-0.63977900	-4.72800900	-2.55268400
H	0.39125800	-3.36278200	-2.10612100
C	1.36888600	3.92416100	-2.39037600
H	2.11192800	4.69806600	-2.15688700
H	0.66702700	4.34728400	-3.12031500
H	1.88513000	3.07583600	-2.85611100
C	-0.49904700	-1.32689800	3.55536500
H	-1.10252000	-0.84507900	4.33805700
H	-1.19658400	-1.64328500	2.76320900
C	5.44253900	-1.65556700	-1.21507100
H	5.70592700	-0.80037800	-1.85905100
H	6.38603500	-2.14383500	-0.93116200
C	3.98729500	3.15117100	-0.03477100
H	4.25162900	2.22048200	0.48217000
H	4.73065100	3.91206900	0.23893300
H	4.04076200	2.98579600	-1.11778100
C	-1.28059900	-2.81090100	-3.36805500
H	-2.29334200	-3.18597400	-3.58918000
H	-0.69736000	-2.90329000	-4.29541600
C	-5.37090500	0.00043200	1.77244900

H	-5.43771600	-0.72463200	2.59624100
H	-5.31451400	1.00094600	2.22969600
C	-0.18189000	4.61318600	-0.47371500
H	-0.72906200	4.24116700	0.39885300
H	-0.90219000	5.03270600	-1.18793200
H	0.48430000	5.42700800	-0.15712500
C	0.22409300	-2.56392500	4.11805000
H	0.84679800	-2.25792900	4.97435400
H	-0.50402400	-3.29538900	4.49754100
B	1.27373700	1.42785100	0.19754700
C	-1.16832700	1.13354700	0.01663700
O	-1.41983700	1.83998300	0.98942900
O	0.09980300	0.76152000	-0.32357000

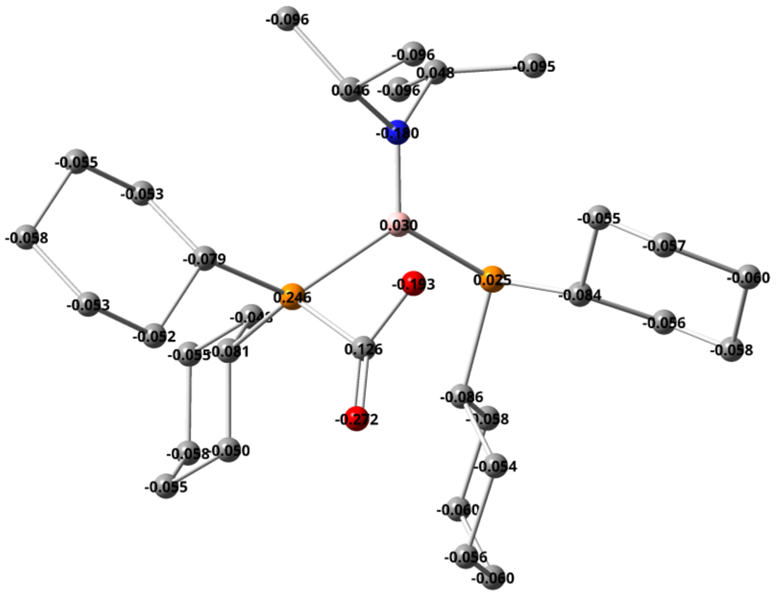

FIG. S115. OPTIMIZED STRUCTURE **2B_TS1**

P	-1.34099700	0.02187800	-0.32664300
P	1.85526800	0.06057600	0.86449300
N	0.05558000	-2.23648700	1.17051400
C	3.08936800	-0.59501800	-0.42443000
H	2.75785900	-0.18977600	-1.38974300
C	4.50081200	-0.05147300	-0.10452700
H	4.48084200	1.03129200	0.08088000
H	4.85614800	-0.52034900	0.82705300
C	-2.87290900	-0.88140200	-0.94206500
H	-2.48240300	-1.79053500	-1.42295800
C	1.59600800	1.84466200	0.22445500
H	0.50787200	1.99518200	0.20455800
C	-2.10401100	1.56294400	0.43132800
H	-3.17795700	1.31829000	0.45988800
C	2.17539700	2.82331900	1.27428500
H	1.75807900	2.59334700	2.26529600
H	3.26154300	2.65571600	1.35392700
C	-0.84768100	-3.26653600	0.60064100
H	-1.39156100	-2.76205600	-0.20250800
C	-3.86581200	-1.28200200	0.16671500
H	-3.36237800	-1.85480000	0.95124400
H	-4.27211100	-0.37461800	0.64072700
C	2.11937500	2.19374500	-1.18673100

H	3.20480000	2.02668600	-1.23139800
H	1.65982000	1.54964700	-1.94365500
C	-1.65422800	1.78040300	1.89378000
H	-1.80075000	0.85989100	2.47501700
H	-0.58024700	2.00808400	1.92853600
C	0.65137900	-2.60170300	2.49376300
H	0.16746300	-3.54768300	2.76068700
C	-3.58922600	-0.04642300	-2.03158800
H	-3.98754000	0.87708900	-1.58211000
H	-2.88191300	0.24633100	-2.81642000
C	-2.75252500	3.99393700	0.22350400
H	-2.61731900	4.91007700	-0.36798600
H	-3.82771000	3.75205300	0.19728700
C	3.17245700	-2.12050900	-0.60515700
H	3.58250300	-2.58235200	0.30210300
H	2.17614200	-2.53777700	-0.77606500
C	2.16169500	-2.88190300	2.51086700
H	2.42774500	-3.63976400	1.76681700
H	2.44181200	-3.25903200	3.50480000
H	2.73668800	-1.96899400	2.31855600
C	2.44402000	4.62572100	-0.49758600
H	2.21892200	5.67006100	-0.75786800
H	3.54160500	4.52443200	-0.49802400
C	-1.95802800	2.84189700	-0.42114900
H	-0.90007400	3.12423600	-0.48889800
H	-2.29951100	2.65781200	-1.44740800
C	5.47264000	-0.37063600	-1.25819200
H	5.13731200	0.16420500	-2.16159300
H	6.47914100	0.00319300	-1.01921100
C	1.84716300	3.66933600	-1.54336300
H	2.25845100	3.88240700	-2.54028900
H	0.76081100	3.83560900	-1.61263800
C	-5.03395000	-2.09319500	-0.42900000
H	-4.64046800	-3.03484700	-0.84414200
H	-5.74034700	-2.36505000	0.36782000
C	-5.74999800	-1.30316300	-1.53913400
H	-6.22688200	-0.41371100	-1.09607900
H	-6.55347300	-1.90941000	-1.97988400
C	-2.31917600	4.22902200	1.68254500
H	-2.91790500	5.03389700	2.13100000
H	-1.27075600	4.56463600	1.69793600
C	-1.88091100	-3.83546600	1.59991200
H	-1.45245700	-4.62376500	2.23231500
H	-2.71370200	-4.28628400	1.04451800
H	-2.28416400	-3.05483200	2.25647800
C	4.10552200	-2.44900800	-1.78757300
H	4.15432900	-3.53819000	-1.93429900
H	3.68173200	-2.01916200	-2.70855800
C	1.90784200	4.29407900	0.90524300
H	0.82076500	4.47673800	0.92390900
H	2.35425400	4.95856600	1.65931000
C	0.25963000	-1.57509100	3.57369000
H	0.73932000	-0.60936400	3.37482100
H	0.58256200	-1.92391300	4.56413600
H	-0.82874300	-1.43387900	3.59177900
C	-2.44764600	2.94360300	2.52041200
H	-3.51019800	2.65838100	2.58808600

H	-2.09751400	3.11833200	3.54722100
C	-4.75959700	-0.85438800	-2.62860500
H	-5.27305600	-0.24892000	-3.38824300
H	-4.35535700	-1.73926700	-3.14482400
C	-0.04429600	-4.40834000	-0.04970200
H	0.59737100	-4.01651900	-0.84509200
H	-0.72418300	-5.15629300	-0.48111900
H	0.58399300	-4.91330300	0.69647000
C	5.51818400	-1.88223500	-1.55058100
H	5.97006600	-2.39921500	-0.68802400
H	6.16452500	-2.08283600	-2.41730100
B	0.22237200	-0.98118300	0.49352400
C	-0.30520200	-0.13467900	-1.93732600
O	-0.36081800	0.52809900	-2.96828600
O	0.34499800	-1.20212100	-1.61201300

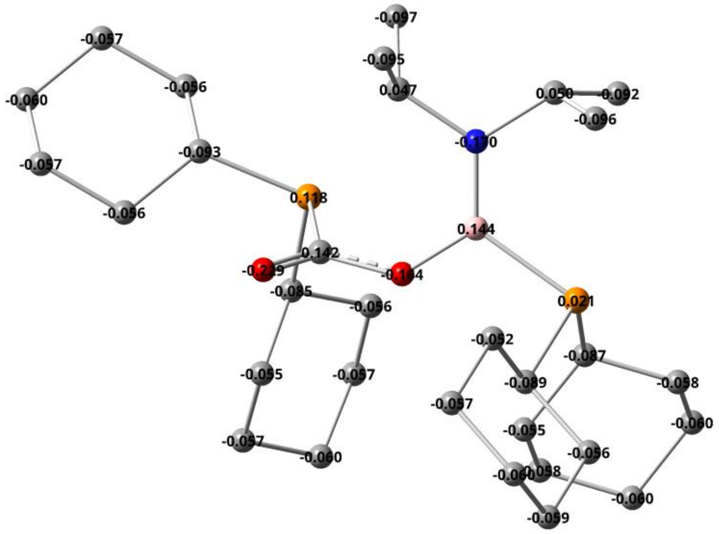

FIG. S116. OPTIMIZED STRUCTURE **2B_TS2**

P	2.33067900	0.08462600	-0.51204100
P	-2.80356500	-0.59516000	-0.65453100
N	-0.49301900	-2.42142700	-1.13024700
C	-3.15580600	-0.52359200	1.20453600
H	-2.43553300	0.16033800	1.67927000
C	-4.59144600	-0.02909700	1.48177400
H	-4.74377000	0.97373900	1.05913600
H	-5.30103400	-0.69757100	0.96828900
C	3.95327900	-0.56530800	0.19289800
H	3.74936300	-1.63728600	0.36009600
C	-2.59482700	1.22901200	-1.16969300
H	-1.77664600	1.22603500	-1.91039700
C	2.38544800	1.93766200	-0.12071000
H	3.45818900	2.17582900	-0.23285300
C	-3.88637000	1.67417600	-1.89817300
H	-4.10323400	0.97722900	-2.71913100
H	-4.73456400	1.60322400	-1.19820600
C	0.81234400	-3.06917000	-0.82743300
H	1.30461900	-2.44453100	-0.07805100
C	5.02980800	-0.45282600	-0.91392700
H	4.66452000	-0.91047900	-1.84402900

H	5.21161700	0.61288200	-1.13331300
C	-2.22340900	2.22635300	-0.05420400
H	-2.98680900	2.19251700	0.73698000
H	-1.27336700	1.93290800	0.41084000
C	1.61101600	2.72824400	-1.19642500
H	1.94536700	2.42811800	-2.19897700
H	0.54393600	2.47021300	-1.12699800
C	-1.30228000	-3.18568100	-2.13605300
H	-0.63485700	-3.98248100	-2.48499900
C	4.46804000	0.03372500	1.51683300
H	4.64845100	1.11208800	1.38166900
H	3.70916900	-0.08648000	2.29709100
C	2.16009800	3.87931100	1.48811100
H	1.83211400	4.17569000	2.49455600
H	3.23952900	4.09464100	1.42722900
C	-2.95409800	-1.92541900	1.82383400
H	-3.62148100	-2.64325700	1.32027700
H	-1.92434100	-2.26985400	1.65471900
C	-2.53649300	-3.87410400	-1.52662600
H	-2.25790100	-4.45477300	-0.63947000
H	-2.97657700	-4.55755900	-2.26605600
H	-3.29393700	-3.13550300	-1.24285800
C	-3.44608600	4.09403700	-1.28601300
H	-3.35646800	5.12030300	-1.67018800
H	-4.26952300	4.09626000	-0.55324500
C	1.93762700	2.36727400	1.29111800
H	0.86671900	2.13962800	1.40862500
H	2.46792900	1.79715900	2.06125400
C	-4.89639100	-0.01275300	2.99258300
H	-4.23212600	0.71504400	3.48640900
H	-5.92797600	0.32792500	3.16378300
C	-2.14354700	3.67272600	-0.58494500
H	-1.91923400	4.35875600	0.24478600
H	-1.31183300	3.75612800	-1.30239700
C	6.35445800	-1.10882600	-0.47768000
H	6.19138100	-2.19188300	-0.35484900
H	7.10944000	-0.98673400	-1.26747900
C	6.86178000	-0.51998900	0.85048200
H	7.11224800	0.54320600	0.70182400
H	7.78692500	-1.02511500	1.16240800
C	1.41053900	4.69434400	0.41981200
H	1.61188900	5.76769400	0.54527600
H	0.32901700	4.55327700	0.56059300
C	1.74244700	-3.12401500	-2.05309100
H	1.36352000	-3.80453500	-2.82668300
H	2.73088300	-3.49120200	-1.74634800
H	1.86162900	-2.12398000	-2.48364900
C	-3.25609100	-1.91667800	3.33450900
H	-3.12487500	-2.92733000	3.74782700
H	-2.52452200	-1.26665000	3.84006200
C	-3.78562000	3.11733700	-2.42443300
H	-2.99627000	3.16892300	-3.19253100
H	-4.72711400	3.40624200	-2.91368600
C	-1.64731400	-2.32980800	-3.36595200
H	-2.37128800	-1.54810600	-3.10687100
H	-2.09404500	-2.96243200	-4.14496900
H	-0.74299500	-1.85974100	-3.77128300

C	1.79596700	4.24624600	-1.00209700
H	2.85208600	4.50099400	-1.18863100
H	1.20080000	4.79265700	-1.74789600
C	5.78862400	-0.63663400	1.94682000
H	6.14566300	-0.18424500	2.88303100
H	5.59826700	-1.70095100	2.15947100
C	0.62402300	-4.45096500	-0.17421700
H	-0.00417900	-4.36959600	0.72042700
H	1.60143600	-4.85328600	0.12170300
H	0.16198200	-5.17005500	-0.86300900
C	-4.67896000	-1.40234100	3.61821200
H	-5.40993200	-2.10760900	3.18998800
H	-4.86452100	-1.36791000	4.70140300
B	-0.95035700	-1.23666700	-0.50099700
C	1.13926800	-0.52713900	0.82375300
O	1.43553500	-0.89358300	1.94933000
O	-0.17524800	-0.49439200	0.42488500

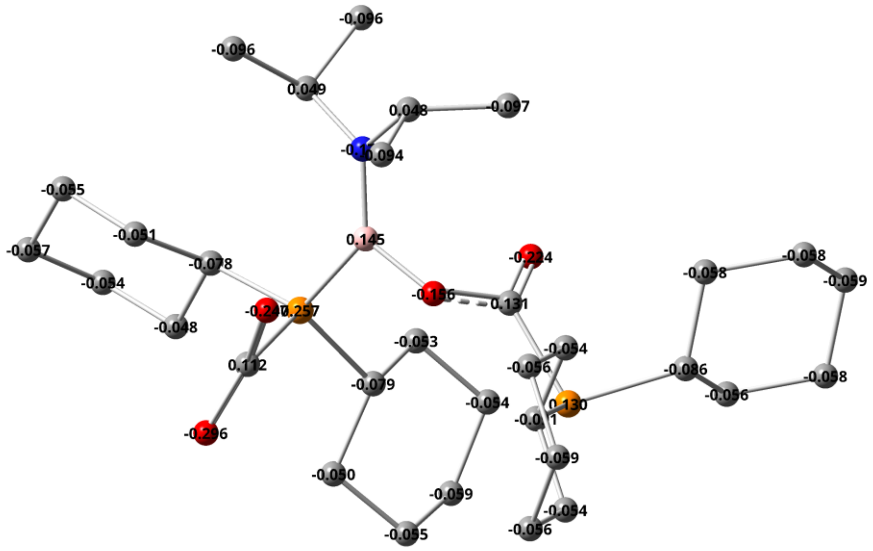

FIG. S117. OPTIMIZED STRUCTURE **2B_TS3**

P	-2.48235900	0.84530100	0.57750600
P	2.14115700	0.31177600	0.36456700
N	1.14126500	-2.08751000	-1.29630900
C	3.94343900	0.38038500	-0.12287300
H	3.97525100	0.38827500	-1.22480700
C	4.67124100	1.64434700	0.38958300
H	4.18505500	2.55093300	0.01157900
H	4.61651400	1.67070300	1.48743700
C	-2.86537000	-0.55938700	1.76063600
H	-1.87291900	-0.79853500	2.17455400
C	1.32849300	1.97959800	0.37718300
H	0.27333000	1.70748000	0.53581500
C	-3.94855600	1.00219500	-0.59599200
H	-4.81773300	0.85520500	0.07016900
C	1.71525200	2.87124100	1.58065200
H	1.65699800	2.29542200	2.51206800
H	2.75033600	3.22294300	1.47851200
C	2.41725300	-2.22324300	-2.03060100
H	3.03404100	-1.37904800	-1.70745600

C	-3.75714800	-0.03803300	2.91174000
H	-3.31582000	0.86738600	3.35060400
H	-4.74451200	0.24592000	2.51034600
C	1.40964600	2.73913900	-0.96496200
H	2.44614100	3.06222400	-1.14854600
H	1.10734500	2.08159100	-1.78918500
C	-4.00513700	2.44779100	-1.14685500
H	-3.96345300	3.16742500	-0.31756400
H	-3.11260100	2.61942600	-1.76812500
C	0.14764200	-3.17893500	-1.53774600
H	0.65641700	-3.89106600	-2.19945000
C	-3.45959700	-1.84607800	1.15733000
H	-4.44665200	-1.62286600	0.72261700
H	-2.81838200	-2.21544200	0.34750300
C	-5.31868200	0.21131800	-2.58421200
H	-5.35685100	-0.51518900	-3.40866200
H	-6.20212000	0.02392600	-1.95175100
C	4.66602500	-0.88366200	0.40619700
H	4.63344100	-0.86395700	1.50480100
H	4.14604500	-1.79521200	0.09488700
C	-0.19210100	-3.91947800	-0.23362300
H	0.71521900	-4.31803100	0.23307900
H	-0.87965800	-4.74985500	-0.44408600
H	-0.66657600	-3.24504800	0.48661100
C	0.81834900	4.87481100	0.29423900
H	0.12199200	5.72434600	0.33171900
H	1.82879900	5.29579300	0.16193600
C	-4.03828500	-0.01425500	-1.75563900
H	-3.15922100	0.11237300	-2.40131900
H	-4.01410800	-1.04097900	-1.37253400
C	6.14576600	1.62776500	-0.06336200
H	6.18117400	1.68673000	-1.16339600
H	6.65299200	2.52395400	0.32015700
C	0.48458700	3.97148500	-0.90765700
H	0.56122200	4.53649000	-1.84734400
H	-0.55539900	3.62013700	-0.82573300
C	-3.94316500	-1.12039000	3.99376700
H	-2.96674500	-1.32653800	4.45947400
H	-4.60477200	-0.74468400	4.78725400
C	-4.50601300	-2.42350700	3.39762900
H	-5.52491200	-2.23844300	3.01924900
H	-4.59021000	-3.19247400	4.17832300
C	-5.38113800	1.64797700	-3.13248500
H	-6.31086500	1.80222600	-3.69838300
H	-4.54682200	1.80066300	-3.83565700
C	2.20707600	-2.07785900	-3.54905700
H	1.59203100	-2.89544100	-3.94760400
H	3.17436700	-2.10570500	-4.06800300
H	1.70833900	-1.12848200	-3.77635100
C	6.13073700	-0.90662700	-0.06525400
H	6.62463200	-1.80937000	0.31959500
H	6.16099400	-0.96786400	-1.16550400
C	0.76939700	4.08812800	1.61803100
H	-0.25902500	3.73662600	1.79574300
H	1.03975400	4.73931800	2.46100300
C	-1.10783700	-2.69690200	-2.28451700
H	-1.76177700	-2.10871800	-1.63456900

H	-1.67833900	-3.56692200	-2.63736400
H	-0.83956600	-2.07501400	-3.14532000
C	-5.27362700	2.67500500	-1.99112400
H	-6.15866800	2.58636300	-1.33976700
H	-5.27517700	3.69813700	-2.39347700
C	-3.62400100	-2.92904800	2.24176900
H	-4.05581500	-3.83801100	1.79872300
H	-2.62982200	-3.20070200	2.62957000
C	3.17026400	-3.52070600	-1.67572500
H	3.24129200	-3.64269700	-0.58869300
H	4.18394400	-3.49121100	-2.09627300
H	2.66888400	-4.40613000	-2.08754500
C	6.87584100	0.35592100	0.40203700
H	6.93101900	0.35258000	1.50183700
H	7.90904500	0.35363600	0.02802200
B	0.92748100	-1.08675900	-0.33523800
C	-1.16650100	0.12964900	-0.51032800
O	-0.89493600	0.55062100	-1.63397300
O	-0.39623700	-0.78007700	0.15650300
C	2.01164400	-0.65063900	2.05285700
O	2.37324200	-0.17522900	3.12945200
O	1.52927100	-1.77186800	1.68560700

Formation of 2c and 2d

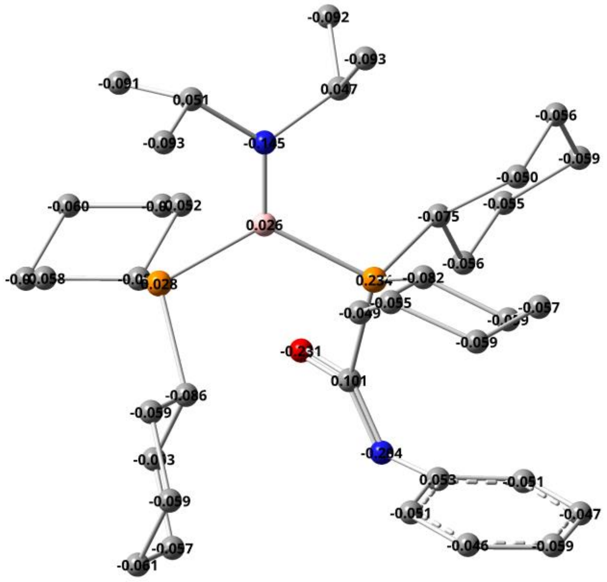

FIG. S118. OPTIMIZED STRUCTURE **2D_I1_A**

P	-0.56425200	-0.73284900	-0.19485900
P	2.13153600	1.26230600	0.73440400
N	1.43124200	-1.30366100	1.92797800
C	3.20714000	0.87931900	-0.78782900
H	2.65226900	1.24718600	-1.65740000
C	4.55183800	1.62891800	-0.67662400
H	4.37568000	2.70386400	-0.53036900
H	5.09605500	1.27594400	0.21481100
C	-0.47000600	-2.44999100	-1.03431000

H	0.54342200	-2.80707600	-0.79252500
C	0.85748800	2.57096300	0.21343500
H	-0.09289700	2.07091900	-0.02892800
C	-1.90096200	-0.74014300	1.14190500
H	-1.64281100	-1.60411500	1.77521000
C	0.62022000	3.47766400	1.44397500
H	0.30135500	2.87068600	2.30467200
H	1.57832800	3.94071400	1.72859000
C	0.82284900	-2.65794600	2.03642200
H	0.06482900	-2.69870800	1.25558200
C	-1.49192500	-3.48198000	-0.50092100
H	-1.52564300	-3.51423100	0.59661600
H	-2.49536400	-3.18998300	-0.83906200
C	1.26515700	3.40892700	-1.01260700
H	2.25339000	3.86342500	-0.83267000
H	1.34997600	2.76100800	-1.89277500
C	-1.72560600	0.53912800	1.99457400
H	-0.68236400	0.63931400	2.32709200
H	-1.95410300	1.41358900	1.36929900
C	2.42140200	-1.05290200	3.04022700
H	2.39840500	-1.97086900	3.63796500
C	-0.57619200	-2.40411000	-2.57718200
H	-1.58945400	-2.07767800	-2.85667000
H	0.12872600	-1.67482600	-2.98498900
C	-4.28957600	-0.93910000	1.94034200
H	-5.33210200	-1.04529800	1.60995100
H	-4.05034000	-1.82831500	2.54749900
C	3.44252000	-0.62733100	-1.01898500
H	3.94585400	-1.07334200	-0.14898800
H	2.47291800	-1.12832200	-1.14089200
C	3.87463200	-0.88755800	2.57417300
H	4.17822900	-1.72723400	1.93840900
H	4.52811800	-0.86993300	3.45728000
H	4.01268800	0.04986700	2.02560000
C	-0.00598100	5.41451700	-0.07008000
H	-0.77337300	6.16998600	-0.29242000
H	0.92187800	5.96057900	0.16708100
C	-3.36961500	-0.88861500	0.70492800
H	-3.65061700	-0.03143400	0.08765000
H	-3.51346600	-1.78539700	0.09404000
C	5.41629300	1.39694100	-1.93177300
H	4.90511400	1.83380200	-2.80450700
H	6.37867900	1.91925700	-1.82821100
C	0.23306200	4.51755300	-1.29764700
H	0.57079500	5.12267200	-2.15130200
H	-0.71186500	4.04491800	-1.60374100
C	-1.17291800	-4.88331600	-1.05765300
H	-0.17029400	-5.18915800	-0.71761100
H	-1.88749000	-5.61267100	-0.65027200
C	-1.21955300	-4.88084000	-2.59511300
H	-2.25294200	-4.67890000	-2.91971700
H	-0.94790700	-5.87052000	-2.98825900
C	-4.12873300	0.32759600	2.79871400
H	-4.77406900	0.27644700	3.68695100
H	-4.45357400	1.19812000	2.20882800
C	0.10232800	-2.90255800	3.37624800
H	0.79965400	-3.03610000	4.21241100

H	-0.49572800	-3.81966900	3.29803200
H	-0.57032700	-2.07169300	3.61834700
C	4.30366500	-0.85639900	-2.27586700
H	4.47836500	-1.93347000	-2.41955000
H	3.74403600	-0.49970900	-3.15408000
C	-0.41681700	4.57804300	1.15520600
H	-1.39861100	4.11330200	0.96891800
H	-0.53641700	5.22189700	2.03900700
C	1.95267100	0.08857800	3.95768700
H	1.99712900	1.04849100	3.43260000
H	2.60569600	0.14090400	4.83910300
H	0.92519100	-0.08897600	4.29862400
C	-2.66089100	0.52129700	3.21762100
H	-2.36190100	-0.30115700	3.88856800
H	-2.53976800	1.45515300	3.78463900
C	-0.28849500	-3.79901400	-3.16751700
H	-0.37967900	-3.75420900	-4.26155900
H	0.75811700	-4.06625100	-2.94785000
C	1.85616300	-3.75500700	1.72589500
H	2.32142400	-3.57621100	0.74925100
H	1.36357500	-4.73566700	1.70807900
H	2.64886700	-3.79180000	2.48423100
C	5.64434900	-0.10538400	-2.18024600
H	6.23431500	-0.52261700	-1.34719400
H	6.23355900	-0.25633400	-3.09627900
B	1.08896100	-0.38287600	0.91012900
C	-0.43784300	0.47206500	-1.81807800
O	0.74375000	0.28627000	-2.18099400
N	-1.40142400	1.15520700	-2.33350400
C	-2.71282800	1.25992900	-1.88459400
C	-3.09534300	2.25472600	-0.95530100
C	-3.72669100	0.45556600	-2.45811400
C	-4.43717300	2.41685600	-0.58964000
H	-2.32684600	2.90020500	-0.53835900
C	-5.06581900	0.62107700	-2.08713100
H	-3.44162500	-0.28878900	-3.19917800
C	-5.43189000	1.59901900	-1.14746100
H	-4.70592900	3.18761900	0.13204000
H	-5.82877300	-0.01468700	-2.53480700
H	-6.47466800	1.72810300	-0.86408800

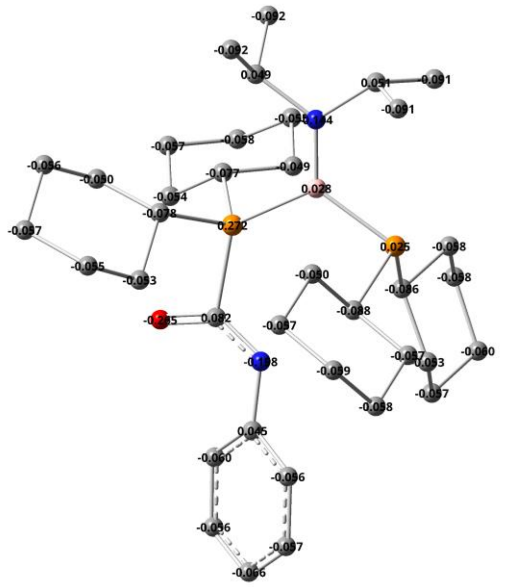

FIG. S119. OPTIMIZED STRUCTURE **2D_I1_B**

P	0.90468200	0.06667000	0.82350400
P	-0.34333500	-0.04288200	-2.40592100
N	2.49437300	-0.34446600	-1.62816200
C	-1.31566800	-1.53915400	-1.75205900
H	-1.72513100	-1.25294000	-0.77125600
C	-2.45031400	-1.90867600	-2.73387700
H	-3.13620200	-1.06304400	-2.86472400
H	-2.01021900	-2.11418900	-3.72367000
C	1.55429900	-1.50313500	1.64933300
H	2.09699000	-2.03020000	0.84942700
C	-1.21869400	1.49569100	-1.72804700
H	-0.99954900	1.58317800	-0.65388900
C	1.92839600	1.58022400	1.28642500
H	2.97931900	1.26552000	1.19368600
C	-0.68011500	2.73919900	-2.46906000
H	0.41484000	2.79934300	-2.38902800
H	-0.91033400	2.64223100	-3.54230500
C	3.67599500	-0.51549400	-0.73270700
H	3.29417700	-0.44977200	0.28773300
C	2.52539100	-1.26614000	2.82257200
H	3.34815300	-0.59687300	2.52986500
H	1.97412600	-0.77437300	3.63529900
C	-2.75109500	1.42989100	-1.85537300
H	-3.02987800	1.31020200	-2.91574500
H	-3.12875500	0.56386400	-1.30383800
C	1.66232900	2.67435000	0.22456400
H	1.81928600	2.27210300	-0.78598500
H	0.60756800	2.97855800	0.28749600
C	2.89201400	-0.39397300	-3.08420200
H	3.97849800	-0.53520800	-3.07018200
C	0.39647500	-2.42564000	2.09694300
H	-0.14436800	-1.93921000	2.91844800
H	-0.32032200	-2.56947300	1.28067000
C	2.69015700	3.35846800	2.90568600
H	2.56219000	3.75405400	3.92280400
H	3.73646200	3.01879100	2.82426600
C	-0.38302300	-2.75674300	-1.56967900

H	0.10214300	-2.99968000	-2.52796700
H	0.42987100	-2.51840000	-0.86546200
C	2.30058900	-1.59901500	-3.82968900
H	2.48251900	-2.52680700	-3.27446600
H	2.78072700	-1.68275300	-4.81406900
H	1.22291000	-1.47516200	-3.97825100
C	-2.85648500	3.96704700	-1.99707300
H	-3.29650200	4.87353400	-1.55714800
H	-3.16031400	3.94509000	-3.05658100
C	1.73852800	2.16198400	2.70428500
H	0.69942300	2.48662500	2.82959700
H	1.91259300	1.39788000	3.46718000
C	-3.23204600	-3.14883000	-2.25561100
H	-3.73662200	-2.91742400	-1.30449900
H	-4.01900700	-3.39600100	-2.98279600
C	-3.39436600	2.70990500	-1.29067200
H	-4.48761900	2.65168700	-1.38881000
H	-3.17529100	2.76466200	-0.21322100
C	3.09517600	-2.61331200	3.31164700
H	3.69625100	-3.06303800	2.50409200
H	3.77447300	-2.44125000	4.15844500
C	1.97131500	-3.58673700	3.71327300
H	1.44146700	-3.18089700	4.58903200
H	2.39870100	-4.55326800	4.01484800
C	2.42912200	4.46113200	1.86541900
H	3.11984200	5.30345600	2.01109100
H	1.40911400	4.85262000	2.00472600
C	4.70833300	0.61363600	-0.90095900
H	5.21460000	0.57337300	-1.87316900
H	5.47655800	0.52093900	-0.12275700
H	4.23073700	1.59453500	-0.80098300
C	-1.16029900	-3.99135200	-1.07464000
H	-0.47394100	-4.84196000	-0.94918600
H	-1.59098500	-3.77465200	-0.08464600
C	-1.31992500	4.02932800	-1.91865600
H	-1.02013400	4.16003100	-0.86609700
H	-0.94077700	4.90249500	-2.47001200
C	2.62412000	0.94233800	-3.79345400
H	1.54865700	1.11263100	-3.90875300
H	3.08246000	0.92373700	-4.79126100
H	3.06050900	1.77467400	-3.22734600
C	2.56611000	3.90217000	0.43942900
H	3.61544600	3.61195600	0.26586800
H	2.31961500	4.67079000	-0.30647200
C	0.96427900	-3.77797900	2.56471800
H	0.14002800	-4.43043400	2.88404100
H	1.46098100	-4.28091900	1.71842100
C	4.30705900	-1.91103000	-0.88641900
H	3.55088600	-2.69421900	-0.75481900
H	5.08484200	-2.04580500	-0.12426700
H	4.77498500	-2.04649400	-1.86940600
C	-2.29739000	-4.35274000	-2.04646200
H	-1.86446600	-4.64924800	-3.01594800
H	-2.86165100	-5.21716700	-1.66862600
B	1.17573800	-0.14008000	-1.17146100
C	-0.77421700	0.26902500	1.75792800
C	-3.12406900	0.13231000	1.61264200

C	-3.44743000	0.83912000	2.79742000
C	-4.17233500	-0.51217800	0.91449300
C	-4.77411800	0.90382000	3.24143700
H	-2.65385900	1.32379400	3.35770100
C	-5.49170000	-0.45444900	1.37233300
H	-3.92704700	-1.06231700	0.00809900
C	-5.80479300	0.26026400	2.53935200
H	-5.00278200	1.45855100	4.15115000
H	-6.27697400	-0.96454700	0.81530900
H	-6.83209900	0.31240200	2.89583600
N	-1.84381300	0.06521800	1.02654200
O	-0.53244600	0.46284800	2.97622900

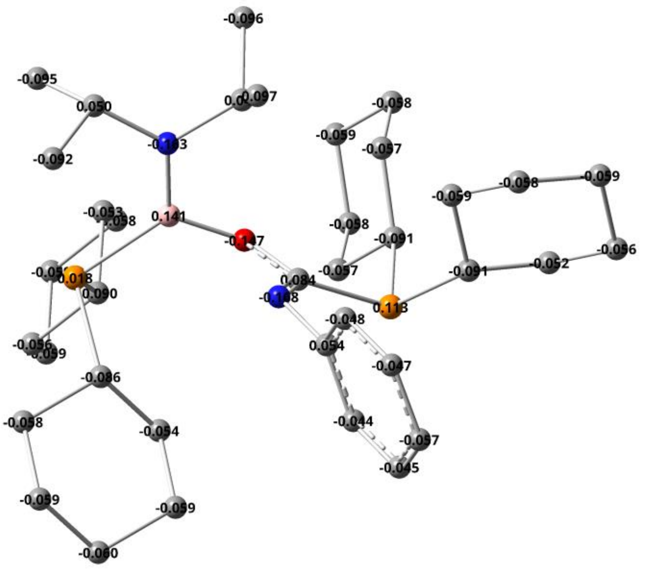

FIG. S120. OPTIMIZED STRUCTURE **2D_I2_A**

P	-1.69952700	-0.57679600	-1.56674200
P	2.87562600	0.82900100	0.81442300
N	0.76358100	-0.26266900	2.62865400
C	3.38658300	-0.40746800	-0.52183100
H	2.61659300	-0.43776500	-1.30837200
C	4.73558800	0.00456700	-1.14969600
H	4.65046900	0.99360300	-1.62041200
H	5.48348200	0.10168300	-0.34641000
C	-3.46969000	-0.50908000	-0.89860700
H	-3.79533500	0.50557400	-1.17692400
C	2.26857300	2.35725300	-0.15269000
H	1.39170600	2.70888700	0.41453600
C	-1.19027600	-2.38918300	-1.51666900
H	-1.93495200	-2.84229500	-2.19561700
C	3.35431300	3.45745700	-0.09352500
H	3.64182600	3.63777000	0.95142300
H	4.26007100	3.10011100	-0.61010300
C	-0.55735000	-0.89022100	2.91241600
H	-1.02725700	-1.05280400	1.94006600
C	-4.35764300	-1.50432900	-1.67984600
H	-4.19280400	-1.39959400	-2.76217300
H	-4.08243000	-2.53546100	-1.40711900
C	1.81753300	2.12152600	-1.60756200
H	2.66181000	1.73424100	-2.19857500

H	1.02689400	1.36237200	-1.64931000
C	0.19081300	-2.54249400	-2.19976600
H	0.18948100	-2.01642100	-3.16466300
H	0.95127100	-2.06250500	-1.56909400
C	1.55923900	0.07188600	3.84912900
H	0.93785200	-0.24597600	4.69532600
C	-3.69976900	-0.66125900	0.61881700
H	-3.41571000	-1.67318000	0.94115600
H	-3.07010700	0.04402000	1.17183400
C	-0.85374300	-4.64328100	-0.38322300
H	-0.86120400	-5.16001200	0.58757600
H	-1.61728400	-5.13156900	-1.01085100
C	3.49650800	-1.81735700	0.10114700
H	4.19254400	-1.78166200	0.95467000
H	2.52013700	-2.12932300	0.50111800
C	2.86933300	-0.73003400	3.92346000
H	2.67030600	-1.80295200	3.81174000
H	3.35168600	-0.56314400	4.89620600
H	3.56135800	-0.41324900	3.13502800
C	2.41769800	4.52020700	-2.20287800
H	2.04708400	5.45359700	-2.65049600
H	3.28223900	4.19613400	-2.80550300
C	-1.23198600	-3.16158600	-0.18264000
H	-0.52499900	-2.69617300	0.51493400
H	-2.23051200	-3.09657400	0.26648900
C	5.22191800	-1.03066400	-2.18181800
H	4.50688800	-1.07099000	-3.01972900
H	6.18908200	-0.71736100	-2.60084900
C	1.32838300	3.43485900	-2.25147000
H	1.01689000	3.24773800	-3.29009800
H	0.44044300	3.79257300	-1.70870800
C	-5.84452000	-1.27455200	-1.34301700
H	-6.13818000	-0.27249400	-1.69351400
H	-6.46688500	-2.00075900	-1.88528000
C	-6.09755200	-1.37776500	0.17251100
H	-5.89956200	-2.41142000	0.50077700
H	-7.15345400	-1.16909100	0.39559100
C	0.52106700	-4.78577200	-1.05868200
H	0.76354400	-5.84674700	-1.21333500
H	1.29609000	-4.37187800	-0.39427100
C	-1.47238800	0.05374900	3.71030600
H	-1.10435200	0.22550900	4.73040900
H	-2.47656300	-0.38293700	3.78721600
H	-1.54557000	1.01668400	3.19363200
C	3.99739900	-2.85384400	-0.92296100
H	4.09112500	-3.83929300	-0.44361200
H	3.24854600	-2.95759800	-1.72294900
C	2.87374500	4.76389900	-0.75331800
H	2.02810900	5.16846300	-0.17363100
H	3.67387100	5.51787300	-0.72221500
C	1.78160700	1.58688000	3.99369200
H	2.46244500	1.95337600	3.21620900
H	2.22855500	1.80620200	4.97285800
H	0.82959900	2.12471200	3.91264200
C	0.54880600	-4.02727200	-2.39703800
H	-0.17548000	-4.48647300	-3.08967700
H	1.53843800	-4.11422600	-2.86769200

C	-5.18340000	-0.41733000	0.95510800
H	-5.34523500	-0.53127400	2.03700600
H	-5.43628700	0.62397400	0.70041400
C	-0.39786500	-2.26589700	3.58258100
H	0.22945300	-2.92231000	2.96743300
H	-1.38281400	-2.73641300	3.69967500
H	0.05542800	-2.18868900	4.57967300
C	5.33781500	-2.42792300	-1.54721900
H	6.11297800	-2.40547300	-0.76407500
H	5.66029500	-3.16529900	-2.29631800
B	1.16244800	0.01962300	1.31111600
C	-0.76515400	0.22183000	-0.16172700
O	0.32239000	-0.47767800	0.24001900
N	-1.00095900	1.36333000	0.40165700
C	-2.01699300	2.22801900	-0.05005100
C	-2.91558000	2.76700200	0.89423100
C	-2.12132100	2.63796600	-1.39780200
C	-3.92768100	3.64560100	0.49196500
H	-2.81692600	2.47504900	1.93754900
C	-3.12930000	3.52540200	-1.79114600
H	-1.41662800	2.24931200	-2.12865200
C	-4.04494100	4.02716700	-0.85349500
H	-4.62227600	4.03819500	1.23309000
H	-3.19645900	3.82676700	-2.83546900
H	-4.82915800	4.71483900	-1.16380500

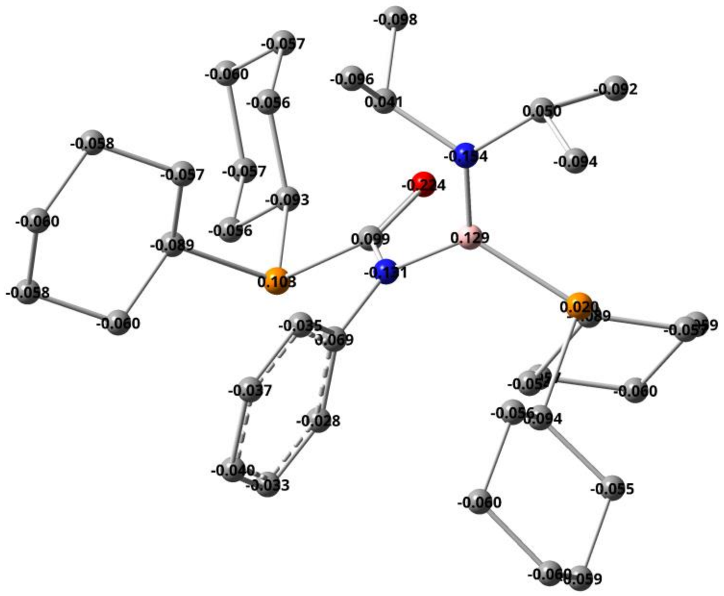

FIG. S121. OPTIMIZED STRUCTURE **2D_I2_B**

P	-2.18029300	0.29470500	-1.24420400
P	2.83947900	0.95871000	0.76736200
N	0.95899800	-0.39862900	2.62793200
C	3.46417300	-0.09262700	-0.66879200
H	2.65956200	-0.24341300	-1.40300300
C	4.65724400	0.57754400	-1.38326300
H	4.36900900	1.56119100	-1.77716100
H	5.46006900	0.75273900	-0.64907200
C	-3.35478700	1.63394900	-0.64168900
H	-2.69644900	2.51066300	-0.53834700
C	2.02022900	2.47232700	-0.04702900

H	1.16489900	2.68836900	0.61196400
C	-3.13582200	-1.29421900	-0.84094200
H	-4.18446300	-0.95684100	-0.92449700
C	2.97156300	3.69013500	-0.00160700
H	3.35072700	3.82925400	1.02015200
H	3.84891900	3.49683900	-0.63975700
C	-0.32152400	-1.05361300	3.01943900
H	-0.83353800	-1.26645200	2.07970700
C	-4.39371200	1.92610200	-1.74900300
H	-3.88371900	2.11461100	-2.70411500
H	-5.03147900	1.03808100	-1.89800800
C	1.46478000	2.28440600	-1.47144400
H	2.28947300	2.06483700	-2.16741800
H	0.78221200	1.42922700	-1.50629800
C	-2.92012200	-2.39113000	-1.90612900
H	-3.07294700	-1.97488300	-2.91127400
H	-1.88121200	-2.74389000	-1.86046500
C	1.83311100	-0.05944800	3.79674900
H	1.30208100	-0.45056400	4.67266000
C	-4.05358100	1.40600900	0.71369300
H	-4.69448100	0.51159800	0.65285900
H	-3.30108800	1.23217600	1.49083100
C	-3.87492700	-3.07065600	0.82121800
H	-3.69619400	-3.48176200	1.82584100
H	-4.91938100	-2.71928500	0.80065500
C	3.89835000	-1.47368000	-0.13073800
H	4.69759600	-1.32910000	0.61437300
H	3.06348700	-1.96255900	0.38980700
C	3.18752200	-0.78266400	3.73013200
H	3.04163600	-1.86105400	3.59066600
H	3.73790900	-0.62296600	4.66720000
H	3.79239900	-0.39555800	2.90277800
C	1.66261600	4.77979200	-1.89598800
H	1.12130900	5.68549800	-2.20563300
H	2.48439300	4.63623200	-2.61698200
C	-2.93313000	-1.87726600	0.57051200
H	-1.89420200	-2.22773100	0.64938800
H	-3.07906100	-1.10363500	1.33411400
C	5.18098000	-0.31160200	-2.52882600
H	4.39135400	-0.41387100	-3.29105600
H	6.03802600	0.17278000	-3.01910200
C	0.73196500	3.55465100	-1.94503500
H	0.34347000	3.40677900	-2.96350000
H	-0.13668700	3.72576900	-1.28979400
C	-5.28586300	3.12521200	-1.37177200
H	-4.66080100	4.03136900	-1.32341900
H	-6.03639500	3.29540500	-2.15717500
C	-5.97121800	2.91104300	-0.01018600
H	-6.66743700	2.05947200	-0.08641200
H	-6.57258600	3.79190700	0.25645500
C	-3.67956800	-4.16060500	-0.24828700
H	-4.37685500	-4.99323500	-0.07828400
H	-2.65984700	-4.56928700	-0.16120400
C	-1.21788600	-0.10234500	3.83118700
H	-0.78581000	0.12527600	4.81511600
H	-2.19569900	-0.57332000	3.99865600
H	-1.36466600	0.83143800	3.27873100

C	4.40878100	-2.37622800	-1.26838500
H	4.71973500	-3.35143600	-0.86559700
H	3.58050300	-2.56744500	-1.96738200
C	2.25510200	4.96629400	-0.48683000
H	1.44115400	5.20541600	0.21669100
H	2.95082300	5.81813700	-0.47382200
C	1.97171500	1.45902500	4.00205400
H	2.58681400	1.90425000	3.21191400
H	2.45524800	1.65552400	4.96876000
H	0.98626900	1.93892400	3.99766800
C	-3.86652000	-3.58254600	-1.66309000
H	-4.91073300	-3.25018200	-1.78447800
H	-3.68849800	-4.36026900	-2.41955000
C	-4.93327500	2.61579100	1.08709800
H	-5.43425100	2.43042800	2.04846300
H	-4.28755600	3.49725400	1.22702300
C	-0.09466900	-2.39385700	3.74244700
H	0.57274100	-3.04745200	3.16682400
H	-1.05777900	-2.90571500	3.86596100
H	0.33935400	-2.26151300	4.74196000
C	5.57624900	-1.71023000	-2.01979000
H	6.43598100	-1.61385000	-1.33675100
H	5.90342200	-2.34281600	-2.85765400
B	1.21838200	-0.03305900	1.29527300
C	-0.88285100	0.47977700	0.12285000
C	0.37856300	-1.51077000	-0.65316800
C	0.42513200	-1.35300400	-2.05161700
C	0.58161700	-2.78788000	-0.09916300
C	0.63367800	-2.46456400	-2.87661600
H	0.29729100	-0.36674200	-2.48710300
C	0.79949700	-3.89499300	-0.92836900
H	0.56617400	-2.90276900	0.98193900
C	0.81736900	-3.73960500	-2.32181900
H	0.66187700	-2.32839800	-3.95609800
H	0.95242400	-4.87689300	-0.48415000
H	0.98350200	-4.59978500	-2.96713800
N	0.17652800	-0.40105100	0.22714700
O	-0.91263800	1.47423900	0.87705700

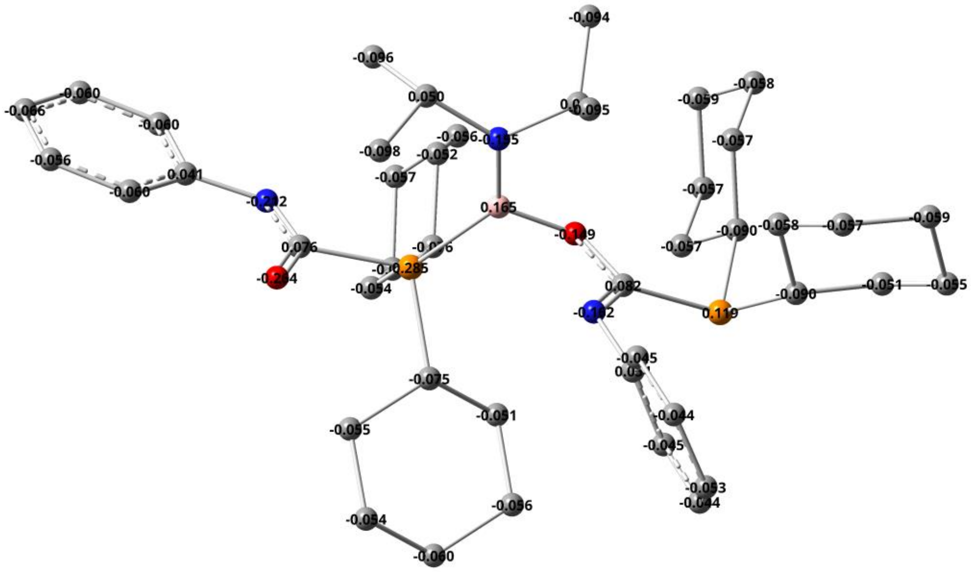

FIG. S122. OPTIMIZED STRUCTURE **2D_I3_A1**

P	3.14336200	0.53800400	-1.16934500
P	-1.83516000	-0.48473900	-0.37613300
N	-0.31963900	0.18249600	2.23769100
C	-2.06723900	1.08303900	-1.35761300
H	-1.09943300	1.23609300	-1.86159600
C	-3.18023700	0.95991400	-2.42141300
H	-2.95690700	0.13737700	-3.11384700
H	-4.11803400	0.71569500	-1.90623900
C	4.66737300	0.20138200	-0.09750300
H	4.97228500	-0.80763200	-0.41597300
C	-1.14581600	-1.80029200	-1.52780800
H	-0.51666800	-2.42880500	-0.87984200
C	2.80757200	2.38664500	-1.04866600
H	3.72843100	2.79948400	-1.49841200
C	-2.27432600	-2.67691700	-2.12746900
H	-2.87876400	-3.10285400	-1.32168200
H	-2.93676800	-2.04745600	-2.74122300
C	0.94454800	0.69081500	2.85651300
H	1.64618400	0.84563400	2.03530000
C	5.80392300	1.17083400	-0.49581100
H	5.92528100	1.19328600	-1.58865000
H	5.54619000	2.19262200	-0.17591300
C	-0.26449000	-1.21740300	-2.65731800
H	-0.87926500	-0.56970900	-3.30011100
H	0.54398600	-0.59480300	-2.25792900
C	1.63988000	2.75787500	-1.99533500
H	1.80465800	2.31213400	-2.98628900
H	0.71283600	2.32759400	-1.59387300
C	-1.38776500	-0.13769800	3.24021800
H	-0.95353300	0.14334000	4.20704700
C	4.49766700	0.16499700	1.43495100
H	4.20695600	1.15938500	1.80281000
H	3.69907600	-0.53071200	1.71347400
C	2.45591400	4.56870200	0.20902900
H	2.29850700	5.00518200	1.20584800
H	3.38885900	5.00252700	-0.18632000
C	-2.32305800	2.27812900	-0.41087300
H	-3.23445900	2.06827800	0.16436200
H	-1.49309700	2.37724800	0.30527400
C	-2.62924700	0.74343600	3.04680900
H	-2.34966600	1.80375100	3.09269400
H	-3.35033700	0.53943800	3.84863500
H	-3.13382300	0.55728700	2.09365100
C	-0.78535200	-3.23205000	-4.11881600
H	-0.34579900	-4.04756300	-4.71033300
H	-1.39801700	-2.62830800	-4.80803800
C	2.61942600	3.04048400	0.33428600
H	1.72291400	2.61695000	0.80326800
H	3.47209000	2.81429600	0.98593200
C	-3.32726600	2.27770600	-3.20572000
H	-2.40237300	2.47566400	-3.77296300
H	-4.13809300	2.17532100	-3.94036000
C	0.32365500	-2.35185500	-3.51962200
H	0.95056900	-1.91826500	-4.31202300
H	0.97813000	-2.97672800	-2.89382800
C	7.12593500	0.75180200	0.17811800

H	7.42631600	-0.23486700	-0.20835000
H	7.92203100	1.45967600	-0.09284600
C	6.97387800	0.67478400	1.70837600
H	6.77123900	1.68451700	2.10159900
H	7.91427300	0.33727900	2.16604600
C	1.29050600	4.93101400	-0.72709300
H	1.20060000	6.02211800	-0.82271800
H	0.34835600	4.56859500	-0.28643100
C	1.57131900	-0.34891500	3.79879800
H	0.95746800	-0.52910600	4.69009300
H	2.55224100	0.01105600	4.13420900
H	1.70923200	-1.29641900	3.26748600
C	-2.48557600	3.58347400	-1.21067500
H	-2.69322900	4.41337700	-0.52027600
H	-1.53854500	3.82007400	-1.72137400
C	-1.68032100	-3.79569000	-3.00293000
H	-1.08617500	-4.47098600	-2.36657100
H	-2.49759200	-4.39504300	-3.42767700
C	-1.67102200	-1.64505600	3.29908000
H	-2.11504200	-2.02320100	2.37280000
H	-2.37637300	-1.85015700	4.11527200
H	-0.74214800	-2.19396300	3.49732100
C	1.48155700	4.28553200	-2.11098300
H	2.37947600	4.70938200	-2.58912300
H	0.63045800	4.52137000	-2.76578800
C	5.81816800	-0.26181200	2.10450800
H	5.69369900	-0.27654800	3.19691600
H	6.05447900	-1.29215500	1.79528300
C	0.72190600	2.05270400	3.53558400
H	1.68321700	2.44505600	3.89146000
H	0.04975300	1.97887300	4.39980500
H	0.29485200	2.77149100	2.82597600
C	-3.60342600	3.45964300	-2.26019800
H	-4.56520500	3.29673600	-1.74946800
H	-3.69402900	4.39404800	-2.83188400
B	-0.37557200	0.02818400	0.85280900
C	1.81032700	-0.26733400	-0.14603200
O	0.71544200	0.52606600	0.05044900
N	1.75298400	-1.46994800	0.32102000
C	2.75043400	-2.43962600	0.09203300
C	3.17710900	-3.22140200	1.18500800
C	3.27650900	-2.71346400	-1.18897700
C	4.14765400	-4.21404600	1.01152900
H	2.74357300	-3.03256200	2.16456800
C	4.23628900	-3.71800600	-1.35777000
H	2.93692500	-2.13154800	-2.04248100
C	4.68564900	-4.46691600	-0.25968200
H	4.47600900	-4.79836800	1.86942200
H	4.63215800	-3.91551700	-2.35274500
H	5.43397300	-5.24519200	-0.39466200
C	-3.47392300	-1.17288300	0.33279700
O	-3.42369600	-2.36946300	0.71439800
N	-4.37929800	-0.21528600	0.29540900
C	-5.68464200	-0.41048800	0.77864600
C	-6.54216000	0.71348300	0.69467700
C	-6.19969900	-1.60800300	1.33847700
C	-7.86132600	0.65312600	1.15132200

H	-6.14080700	1.62986200	0.26389400
C	-7.52398800	-1.65703400	1.79289000
H	-5.55359200	-2.47754100	1.40632100
C	-8.36339000	-0.53597600	1.70549600
H	-8.49967600	1.53281900	1.07529500
H	-7.90375900	-2.58505900	2.21985000
H	-9.39108900	-0.58733300	2.06139000

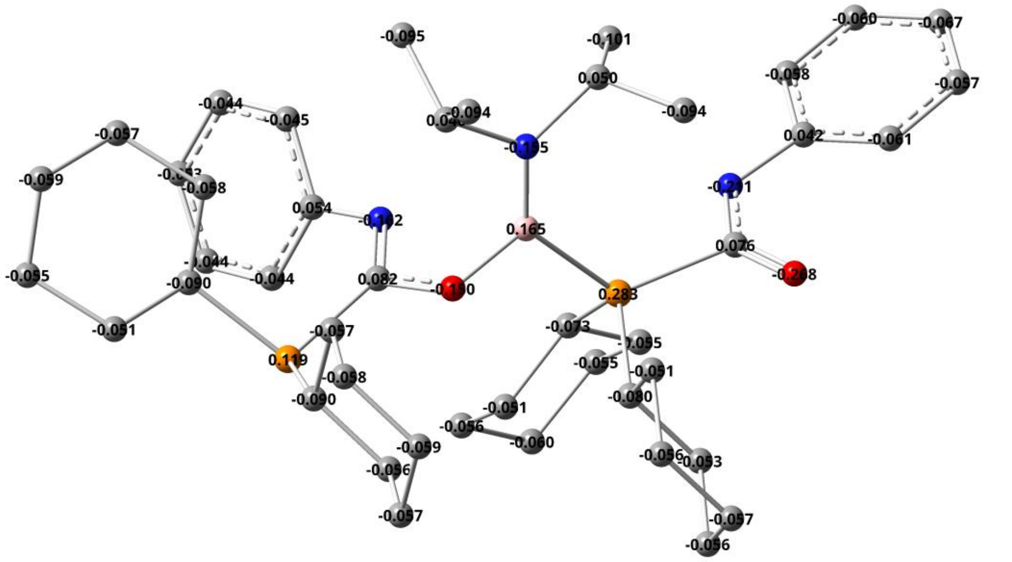

FIG. S123. OPTIMIZED STRUCTURE **2D_I3_A2**

P	-3.17546900	0.31966700	1.03898200
P	1.92940200	0.48962700	0.43226600
N	0.41806400	0.00348900	-2.22628400
C	1.63465700	2.23635100	1.00032900
H	0.63735800	2.22558100	1.46702300
C	2.67604800	2.70142100	2.04425500
H	2.65483100	2.04199200	2.92211400
H	3.67564100	2.62927600	1.59751800
C	-4.50173000	-0.69711600	0.14972900
H	-4.53687100	-1.62502400	0.74147300
C	1.60291700	-0.66591500	1.87972900
H	1.22266500	-1.58316900	1.40590500
C	-3.35379400	2.08517200	0.41124400
H	-4.37678900	2.32736900	0.75104900
C	2.89936600	-1.00750600	2.65261100
H	3.64459200	-1.39951400	1.95409500
H	3.31289100	-0.08890900	3.09665400
C	-0.90047800	-0.00292200	-2.93376800
H	-1.65673300	0.19652900	-2.17277500
C	-5.87522100	-0.00404600	0.30278100
H	-6.04730000	0.28295500	1.35043700
H	-5.89003600	0.92079600	-0.29490600
C	0.52656800	-0.12654900	2.85083200
H	0.89059400	0.80003800	3.31910900
H	-0.40173300	0.12431200	2.32582600
C	-2.39010400	3.00912500	1.19486900
H	-2.47649900	2.81389100	2.27292700
H	-1.35921500	2.77033700	0.90122300
C	1.57636800	-0.33152300	-3.11706900

H	1.13565200	-0.45079600	-4.11408500
C	-4.25962300	-1.10109100	-1.31887900
H	-4.23651900	-0.20435400	-1.95472500
H	-3.28943400	-1.59821100	-1.42273800
C	-3.55533100	3.85914800	-1.39828900
H	-3.46881000	4.04154800	-2.47910000
H	-4.59398100	4.09663300	-1.11597000
C	1.59510200	3.18863100	-0.21932000
H	2.55578600	3.12084300	-0.74698600
H	0.80757300	2.86969600	-0.91924500
C	2.57309100	0.83080700	-3.22215400
H	2.05940500	1.73776100	-3.56578900
H	3.35283000	0.57550200	-3.95102800
H	3.07101400	1.05474500	-2.27604000
C	1.51963500	-1.51953700	4.73242200
H	1.30015300	-2.27587600	5.49919700
H	1.88928900	-0.62473600	5.25925700
C	-3.27515100	2.37217700	-1.10094100
H	-2.26893400	2.11578800	-1.45468500
H	-3.98873000	1.74507500	-1.64915100
C	2.39456000	4.15340700	2.47541000
H	1.41796400	4.20329300	2.98539700
H	3.15348400	4.47176300	3.20364600
C	0.24114900	-1.16153600	3.95726500
H	-0.52899800	-0.76602400	4.63524700
H	-0.16892500	-2.07348200	3.49842400
C	-7.00205500	-0.93616500	-0.18602200
H	-7.04024300	-1.82246700	0.46675300
H	-7.97186100	-0.42687700	-0.09454500
C	-6.76588600	-1.38419800	-1.64009300
H	-6.82800800	-0.50516400	-2.30241800
H	-7.55746700	-2.07794900	-1.95603400
C	-2.59500000	4.77457100	-0.61975200
H	-2.82177500	5.83025300	-0.82426700
H	-1.56501100	4.59436700	-0.96604000
C	-1.21746400	-1.38011100	-3.53796800
H	-0.53898000	-1.64353700	-4.35883700
H	-2.23959300	-1.37261700	-3.93722900
H	-1.15093000	-2.15108000	-2.76336500
C	1.33941300	4.63953400	0.22878700
H	1.34657400	5.30162800	-0.64870600
H	0.33544700	4.71014600	0.67626900
C	2.60548800	-2.02685700	3.76849300
H	2.27211800	-2.97219600	3.31088000
H	3.53458600	-2.24634400	4.31266400
C	2.19766200	-1.67983100	-2.72899800
H	2.64277200	-1.64182300	-1.72800200
H	2.99237300	-1.93924700	-3.44041100
H	1.43386200	-2.46689800	-2.75482600
C	-2.67519800	4.49182300	0.89087900
H	-3.68199200	4.75069700	1.25682800
H	-1.96424300	5.12671300	1.43871400
C	-5.38232200	-2.03975800	-1.80117800
H	-5.20971100	-2.31811300	-2.85080800
H	-5.34293500	-2.96982400	-1.21256300
C	-0.97581100	1.13211400	-3.96874900
H	-1.98163300	1.16247800	-4.40711900

H	-0.25775600	0.99213600	-4.78676500
H	-0.77434000	2.09983100	-3.49376300
C	2.38191300	5.09985400	1.26228500
H	3.37912200	5.10332800	0.79613500
H	2.16913600	6.12951300	1.58289600
B	0.44610000	0.25495700	-0.85491500
C	-1.62817500	-0.33515200	0.23456300
O	-0.77596900	0.64459600	-0.19182600
N	-1.22422800	-1.55539600	0.10825200
C	-1.92625700	-2.66712700	0.61528600
C	-2.04377400	-3.80591200	-0.20792700
C	-2.43863200	-2.72050800	1.92970900
C	-2.70677600	-4.94888900	0.25180200
H	-1.61549700	-3.77546200	-1.20736800
C	-3.08730600	-3.87268900	2.38828300
H	-2.33189600	-1.85625300	2.58119100
C	-3.23515400	-4.98901700	1.55126500
H	-2.80081900	-5.81374300	-0.40273000
H	-3.47652800	-3.89657300	3.40497000
H	-3.74269600	-5.88184200	1.91068200
C	3.76014000	0.39035300	-0.14013600
O	4.34615800	1.48294500	-0.33799700
N	4.05702400	-0.89023600	-0.23915300
C	5.31375700	-1.33267400	-0.68593500
C	5.47977500	-2.73740600	-0.75294000
C	6.39763200	-0.50718800	-1.08116900
C	6.67710000	-3.30277900	-1.19971700
H	4.64312800	-3.36452900	-0.44818000
C	7.59310100	-1.08553500	-1.52701700
H	6.28384200	0.57112700	-1.03030100
C	7.74523600	-2.47908600	-1.59158800
H	6.77884800	-4.38674500	-1.24227600
H	8.41628800	-0.43704400	-1.82636500
H	8.67996500	-2.91625400	-1.93894600

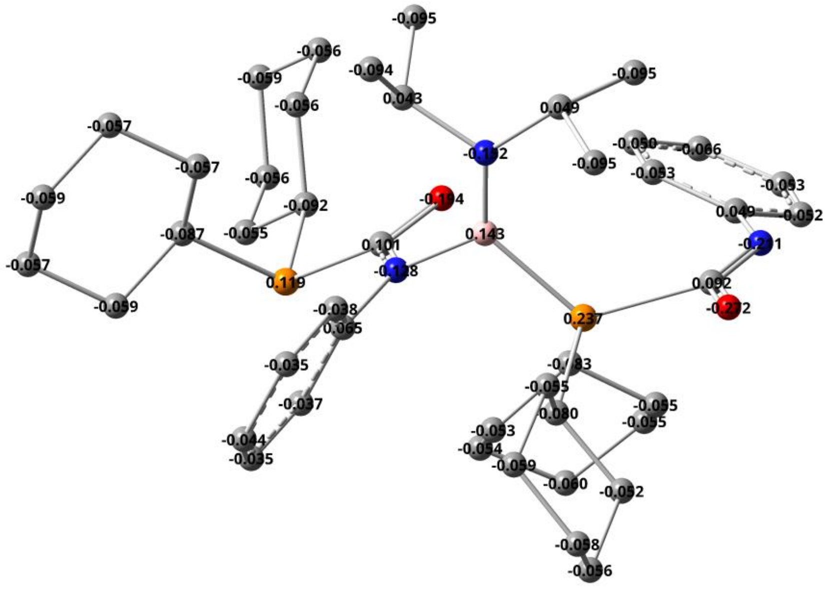

FIG. S124. OPTIMIZED STRUCTURE **2D_I3_B**

P	-2.69685400	0.39673000	-1.20371500
P	2.32255900	-0.11166100	-0.05493900
N	0.62392400	-0.80869500	2.42914800
C	2.71918500	-1.71365700	-0.93888800
H	1.80137600	-1.91757700	-1.51740700
C	3.90335500	-1.58540300	-1.92270700
H	3.73944000	-0.75736200	-2.62195400
H	4.81069100	-1.35930300	-1.34705300
C	-3.21372300	2.13147000	-0.69968500
H	-2.29508300	2.71958300	-0.84948800
C	1.76871900	1.07343600	-1.41520400
H	1.12550000	1.78236100	-0.87912800
C	-4.06267100	-0.67304600	-0.43323100
H	-4.89810100	0.04383000	-0.35125700
C	2.94490900	1.87150500	-2.02999200
H	3.60124800	2.27703100	-1.25766400
H	3.55391600	1.20744300	-2.66026200
C	-0.74730900	-1.02688200	3.00322100
H	-1.40012900	-1.18473600	2.14488100
C	-4.29021200	2.64259800	-1.68474200
H	-3.94337900	2.52078900	-2.72047500
H	-5.20451000	2.03417700	-1.57957200
C	0.94839800	0.44472700	-2.56011200
H	1.59280100	-0.20907600	-3.16720900
H	0.13655200	-0.17479500	-2.17316500
C	-4.52276500	-1.80163100	-1.38159300
H	-4.74604900	-1.38885800	-2.37517500
H	-3.70656100	-2.52527900	-1.50868700
C	1.67693200	-0.75227500	3.50552300
H	1.13331200	-1.02355500	4.41587500
C	-3.67073400	2.32741600	0.76006600
H	-4.57296700	1.72385400	0.94953500
H	-2.88600000	1.98397100	1.44281800
C	-5.00498900	-1.95981700	1.54616600
H	-4.76561900	-2.36870600	2.53866400
H	-5.82436300	-1.23651500	1.68710400
C	2.95220300	-2.90233100	0.01573800
H	3.86103600	-2.70741000	0.59756100
H	2.11925500	-2.99163700	0.72419000
C	2.75971600	-1.81667500	3.29781400
H	2.30827800	-2.80585000	3.14993400
H	3.39607000	-1.85709800	4.19149300
H	3.40605700	-1.57959200	2.44705400
C	1.47537600	2.46822700	-4.01503900
H	1.03327900	3.29386300	-4.59072500
H	2.08996000	1.88160000	-4.71746300
C	-3.77578300	-1.22691100	0.97520000
H	-2.93918600	-1.93828600	0.90935600
H	-3.46968300	-0.41377500	1.64594900
C	4.08040800	-2.89494500	-2.71739500
H	3.18953400	-3.05689100	-3.34669700
H	4.93893000	-2.79672000	-3.39666800
C	0.36657300	1.56434700	-3.44713900
H	-0.22694000	1.12132200	-4.25963100
H	-0.32584600	2.16697200	-2.83858400
C	-4.63571900	4.11867600	-1.40496000
H	-3.74632900	4.73608700	-1.60874600

H	-5.42374500	4.45460700	-2.09387700
C	-5.07314800	4.32769100	0.05604700
H	-6.01579200	3.78378300	0.23228900
H	-5.27874200	5.39106300	0.24288200
C	-5.46985500	-3.08111200	0.59968500
H	-6.36333200	-3.57627000	1.00486200
H	-4.67933400	-3.84583100	0.53224500
C	-1.22737800	0.23562300	3.73846000
H	-0.62985200	0.42643700	4.63937200
H	-2.27185200	0.10415500	4.05099800
H	-1.16135000	1.10601200	3.07731700
C	3.10231100	-4.20720200	-0.78582400
H	3.26288300	-5.04724500	-0.09460300
H	2.16503400	-4.41050800	-1.32811800
C	2.37851800	3.01377900	-2.89434200
H	1.80697300	3.69446800	-2.24530500
H	3.20632000	3.59752500	-3.32046900
C	2.26150600	0.64320000	3.75898100
H	2.95705000	0.95631200	2.97805900
H	2.82377800	0.61266100	4.70161900
H	1.47119500	1.39497500	3.85545700
C	-5.75553500	-2.52702300	-0.80818400
H	-6.60159100	-1.82226500	-0.75592200
H	-6.05758400	-3.33883400	-1.48485900
C	-4.00235200	3.80823600	1.03182200
H	-4.33905200	3.93001800	2.07133800
H	-3.08386800	4.40619200	0.92046600
C	-0.85596900	-2.27785300	3.89745600
H	-0.36464300	-3.14835400	3.44712500
H	-1.91842600	-2.51903800	4.02883300
H	-0.42950100	-2.12395900	4.89634900
C	4.26725500	-4.10536200	-1.78602600
H	5.21049700	-3.99055800	-1.22970500
H	4.34945800	-5.02976400	-2.37534800
B	0.73445800	-0.60146400	1.04437100
C	-1.21020500	0.25599200	-0.05746400
C	-0.71161000	-2.18954000	-0.40998800
C	-1.12870000	-2.37991600	-1.74146900
C	-0.45229300	-3.31862600	0.39100400
C	-1.29837100	-3.67533600	-2.24532600
H	-1.33136100	-1.52408800	-2.37991200
C	-0.61587400	-4.60913800	-0.12235100
H	-0.11386500	-3.17429400	1.41224300
C	-1.04400500	-4.79667000	-1.44401300
H	-1.62327800	-3.80167600	-3.27651500
H	-0.40514400	-5.46687000	0.51366600
H	-1.16998800	-5.80000100	-1.84510800
N	-0.48406100	-0.89722200	0.15099900
O	-0.74721200	1.29018600	0.48153100
C	4.05527600	0.50052800	0.85915600
N	4.26811300	1.75412200	1.13272800
C	3.39367200	2.82974000	1.00975100
C	1.99520900	2.79362000	1.23237900
C	3.97053000	4.08276100	0.67434500
C	1.20771500	3.94076200	1.06540500
H	1.50468000	1.87446900	1.53754200
C	3.18257700	5.22495700	0.52141300

H	5.04802500	4.12051200	0.52643700
C	1.78971100	5.16357800	0.70737700
H	0.13279000	3.86278500	1.22081100
H	3.65525600	6.16936600	0.25392100
H	1.17587000	6.05463800	0.58759200
O	4.76060900	-0.51212800	1.02856200

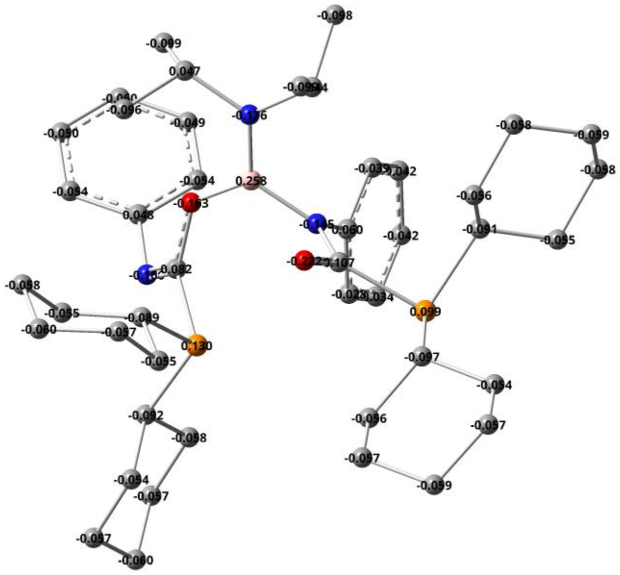

FIG. S125. OPTIMIZED STRUCTURE **2C**

P	1.55532400	1.45097000	-0.18730300
P	-2.73602600	0.60459700	-1.20582500
O	1.69077100	-1.18125400	0.67075500
O	-1.39776000	0.65080300	1.23747800
N	3.33831800	-0.57202500	-0.83367800
N	-0.63565700	-0.94824100	-0.18537500
N	-0.05977700	-2.24995800	1.93124000
C	2.28992900	-0.25505400	-0.16559300
C	-4.53069400	-0.75783000	0.64710800
H	-5.21049300	0.10574200	0.70424500
H	-3.75472300	-0.59803100	1.40644100
C	3.77345600	-1.92080500	-0.85911700
C	5.02112500	-2.25727700	-0.30275700
H	5.61396200	-1.47897800	0.17387500
C	5.47730700	-3.58088600	-0.35430700
H	6.43833100	-3.83398400	0.09104200
C	4.70903300	-4.57498300	-0.97889500
H	5.06964000	-5.60099200	-1.02207100
C	3.47621000	-4.23187300	-1.55519500
H	2.87299100	-4.99174200	-2.05034000
C	3.00636200	-2.91534400	-1.49768500
H	2.05311100	-2.64632800	-1.94557900
C	1.71922700	1.78433500	1.66445300
H	1.20774800	0.91221700	2.10220800
C	0.90262000	3.02192500	2.09569400
H	-0.13169400	2.90718700	1.75071700
H	1.31057800	3.93137600	1.62986600
C	0.93684600	3.18144400	3.62777300

H	0.37469100	4.07944400	3.92186900
H	0.42659900	2.31871200	4.08553100
C	2.38223200	3.25462400	4.15230900
H	2.38771400	3.35603800	5.24699900
H	2.87011800	4.15552800	3.74494700
C	3.18270800	2.00938500	3.72979700
H	4.22145000	2.08024200	4.08375400
H	2.74141400	1.12020800	4.20590500
C	3.16387900	1.82354900	2.19888000
H	3.69390500	0.90189200	1.91971000
H	3.71009300	2.65713500	1.73161400
C	2.95377200	2.36441700	-1.04242100
H	3.91198000	1.97568700	-0.66579100
C	2.86622900	2.07269400	-2.56034600
H	2.94760300	0.99304500	-2.73124900
H	1.87923200	2.40025500	-2.92770700
C	3.96917200	2.81831400	-3.33406500
H	4.95144400	2.43460800	-3.01570900
H	3.87696800	2.60697400	-4.40918500
C	3.90287600	4.33411900	-3.07726500
H	2.95634300	4.72791100	-3.48233500
H	4.71667500	4.85098400	-3.60574800
C	3.96975100	4.63643500	-1.57004700
H	4.95422900	4.32845800	-1.18238000
H	3.87907800	5.71741200	-1.38996200
C	2.87088700	3.88622800	-0.79138800
H	1.88103600	4.25476300	-1.10709200
H	2.96678900	4.10800800	0.27905300
C	-1.46637800	0.11475400	0.11804100
C	-0.50892900	-1.52002600	-1.49780800
C	-0.72121000	-2.89999400	-1.65457200
H	-1.01996200	-3.49161000	-0.79173500
C	-0.53632800	-3.49946200	-2.90723500
H	-0.69875700	-4.56958500	-3.02245800
C	-0.14070500	-2.72224100	-4.00564900
H	0.00791200	-3.18765700	-4.97802400
C	0.07412100	-1.34458800	-3.84460900
H	0.39528600	-0.73937400	-4.69017300
C	-0.10212000	-0.74097100	-2.59451900
H	0.08101000	0.32017700	-2.44984300
C	-3.39820800	2.10606700	-0.30448900
H	-3.47994000	1.86300600	0.76533900
C	-2.36140600	3.24250200	-0.46777600
H	-2.23876700	3.46293500	-1.54099000
H	-1.38385200	2.91521500	-0.09296300
C	-2.82294300	4.51516500	0.26474000
H	-2.87790500	4.30771900	1.34534400
H	-2.08137600	5.31591400	0.12936100
C	-4.20330500	4.97223400	-0.24184700
H	-4.11657800	5.27061600	-1.29939300
H	-4.54320500	5.85837100	0.31317700
C	-5.24084400	3.84042000	-0.12272900
H	-6.20898800	4.16496400	-0.53092700
H	-5.40313700	3.60900600	0.94239900
C	-4.77013000	2.56097200	-0.84402700
H	-5.52104600	1.76921400	-0.71770400
H	-4.69168600	2.75676100	-1.92571200

C	-3.90620300	-0.82416400	-0.76020200
H	-3.23980700	-1.70389200	-0.79691300
C	-4.98970500	-1.04039900	-1.83846800
H	-5.66849700	-0.17388700	-1.86079600
H	-4.52157600	-1.10713200	-2.82983200
C	-5.80011300	-2.31722800	-1.53782900
H	-5.13049600	-3.18923300	-1.61790300
H	-6.58820300	-2.44935000	-2.29291000
C	-6.41273800	-2.27883100	-0.12521300
H	-6.95516800	-3.21268100	0.08000300
H	-7.15115000	-1.46219300	-0.07401200
C	-5.32997400	-2.04160000	0.94391800
H	-5.78410200	-1.98024500	1.94362600
H	-4.63935200	-2.90082700	0.95745000
C	0.90962400	-2.77123600	2.93817900
H	0.30494300	-3.30091600	3.68533000
C	1.64635900	-1.63779600	3.67147100
H	2.32832800	-1.11429500	2.99411300
H	2.23529300	-2.05185300	4.50060100
H	0.92999900	-0.91403200	4.07876900
C	1.87765600	-3.78814000	2.30766900
H	1.32056100	-4.60321600	1.82993000
H	2.52544800	-4.21899800	3.08292000
H	2.50997100	-3.31216600	1.55119000
C	-1.48568000	-2.58776000	2.15850700
H	-2.02352100	-2.17798900	1.29658100
C	-1.72311100	-4.10872500	2.17552100
H	-1.33833700	-4.57632600	1.26064800
H	-2.79910300	-4.31310700	2.24510500
H	-1.23837200	-4.59087800	3.03441900
C	-2.02765500	-1.90085800	3.42393500
H	-1.52293000	-2.26970400	4.32736700
H	-3.10029200	-2.10807100	3.53368800
H	-1.88145600	-0.81801500	3.34884600
B	0.32805300	-1.43556900	0.85551000

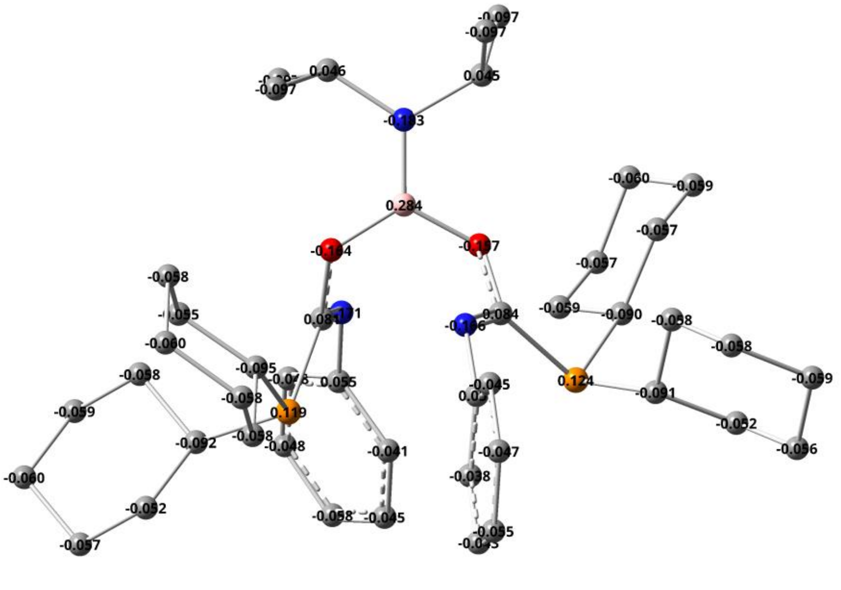

FIG. S126. OPTIMIZED STRUCTURE **2C’**

P	2.38844800	0.20478200	-1.47094300

P	-2.25673200	-0.55183600	-0.69805000
N	0.58673900	1.04740500	3.26948300
C	-2.49078000	-1.86024100	0.62695000
H	-1.49311400	-1.86192400	1.09565100
C	-3.53830100	-1.62120800	1.73045400
H	-3.38512100	-0.63824000	2.19326000
H	-4.54609100	-1.62093700	1.28758600
C	3.72660200	-1.13108300	-1.50357200
H	3.20677200	-1.98455700	-1.96713400
C	-3.91688100	0.22849500	-1.14374600
H	-3.64606600	0.88462100	-1.98708100
C	3.24106300	1.83558300	-1.07735400
H	3.89369100	1.96260100	-1.96014900
C	-4.87930000	-0.84580200	-1.69875400
H	-4.36951000	-1.46261400	-2.45258100
H	-5.18860300	-1.51946800	-0.88508100
C	1.99947700	1.13147300	3.71436500
H	2.59647200	0.88294900	2.83084900
C	4.86038200	-0.70855900	-2.46564800
H	4.44285300	-0.35883900	-3.42118300
H	5.41942700	0.13350600	-2.02802600
C	-4.62324800	1.10616300	-0.09129300
H	-4.93443200	0.49021800	0.76307800
H	-3.93203000	1.86711900	0.29215800
C	2.18067100	2.96507700	-1.13262300
H	1.62754600	2.90457900	-2.07907900
H	1.44925300	2.81743500	-0.32781900
C	-0.45658500	1.29214400	4.30220300
H	0.08417800	1.58527900	5.21168900
C	4.30895500	-1.62418900	-0.16282600
H	4.86184700	-0.81021500	0.32680700
H	3.50333200	-1.92262200	0.51792300
C	4.74791900	3.34466300	0.31598200
H	5.33857700	3.39789100	1.24235000
H	5.44823500	3.51272700	-0.51882500
C	-2.69223900	-3.24802300	-0.02852900
H	-3.67257100	-3.28482500	-0.52927200
H	-1.92758800	-3.41357100	-0.79764500
C	-1.37139000	2.46573400	3.91102700
H	-0.77675800	3.36712700	3.71988900
H	-2.07858000	2.67774000	4.72416500
H	-1.94131300	2.23241900	3.00540900
C	-6.83423700	0.73035300	-1.28631900
H	-7.69931500	1.22385800	-1.75183300
H	-7.22447300	0.11232500	-0.46101000
C	4.12532200	1.93968900	0.18212200
H	3.51250700	1.72555900	1.06614100
H	4.92455400	1.18885100	0.14582700
C	-3.46411500	-2.73515000	2.79453900
H	-2.48119700	-2.69146200	3.28966200
H	-4.22304100	-2.56301300	3.57186000
C	-5.86232500	1.77881500	-0.71391100
H	-6.36976700	2.40047500	0.03809600
H	-5.53266600	2.45248600	-1.52087300
C	5.82633200	-1.88612600	-2.70584800
H	5.28314600	-2.69137400	-3.22543600
H	6.64375100	-1.56915700	-3.36934400

C	6.39114200	-2.42769800	-1.37957600
H	7.01892600	-1.65139100	-0.91203200
H	7.04262800	-3.29226200	-1.57058100
C	3.67191900	4.44394800	0.29651900
H	4.13847000	5.43576800	0.38242400
H	3.01169800	4.32152100	1.16995800
C	2.31817800	0.08925800	4.80132400
H	1.75402600	0.27674700	5.72486000
H	3.38634700	0.12585100	5.05188800
H	2.07693800	-0.91953700	4.44552600
C	-2.62407900	-4.35982200	1.03562100
H	-2.78757800	-5.33963900	0.56422800
H	-1.60887100	-4.37524000	1.46251200
C	-6.12959400	-0.18099600	-2.30827000
H	-5.82347400	0.41963700	-3.17959900
H	-6.82210200	-0.95207300	-2.67560800
C	-1.24911000	0.01288600	4.62023600
H	-1.80920800	-0.31692500	3.74011100
H	-1.95970400	0.19844000	5.43700800
H	-0.57297900	-0.79560700	4.92296500
C	2.83047900	4.35320400	-0.98809200
H	3.47913200	4.54746500	-1.85836500
H	2.04817200	5.12527700	-0.99262400
C	5.25969100	-2.81170500	-0.40818300
H	5.67811100	-3.15949800	0.54742100
H	4.68031100	-3.64899400	-0.82870700
C	2.37136600	2.56031900	4.14892700
H	2.16710500	3.26999300	3.33867400
H	3.43898800	2.61327900	4.39929100
H	1.80540500	2.87555500	5.03593200
C	-3.64951600	-4.12542900	2.15956500
H	-4.66720800	-4.19779200	1.74152600
H	-3.56338300	-4.90889400	2.92622500
B	0.28328900	0.75658500	1.93025800
C	1.48533000	-0.25350500	0.09054000
O	1.32253900	0.78445600	0.96222600
N	0.97226200	-1.39157500	0.41817800
C	0.97818700	-2.52199800	-0.42028400
C	1.33167800	-3.76061500	0.15447300
C	0.55426700	-2.48809200	-1.76696800
C	1.31358500	-4.93015100	-0.61329800
H	1.62393000	-3.78239500	1.20277700
C	0.52788500	-3.66456500	-2.52444300
H	0.22786700	-1.54646600	-2.19957500
C	0.91427100	-4.88930100	-1.95840900
H	1.60123600	-5.87633800	-0.15751200
H	0.19426900	-3.62300100	-3.56030400
H	0.89138200	-5.80086600	-2.55249700
C	-1.47419600	0.82427300	0.27984600
O	-1.03089400	0.46704400	1.53102300
N	-1.24004200	2.03100400	-0.10441200
C	-1.59069400	2.45469900	-1.40474300
C	-1.02309800	1.86608600	-2.55439400
C	-2.47190100	3.54432100	-1.55109600
C	-1.37546600	2.33198200	-3.82606400
H	-0.30480600	1.05681400	-2.44010200
C	-2.82866400	3.99487300	-2.82760900

H	-2.87959100	4.01309400	-0.65767600
C	-2.28851200	3.38805500	-3.97226800
H	-0.93161300	1.86751200	-4.70554500
H	-3.52543400	4.82577900	-2.92772700
H	-2.56376200	3.74329100	-4.96347400

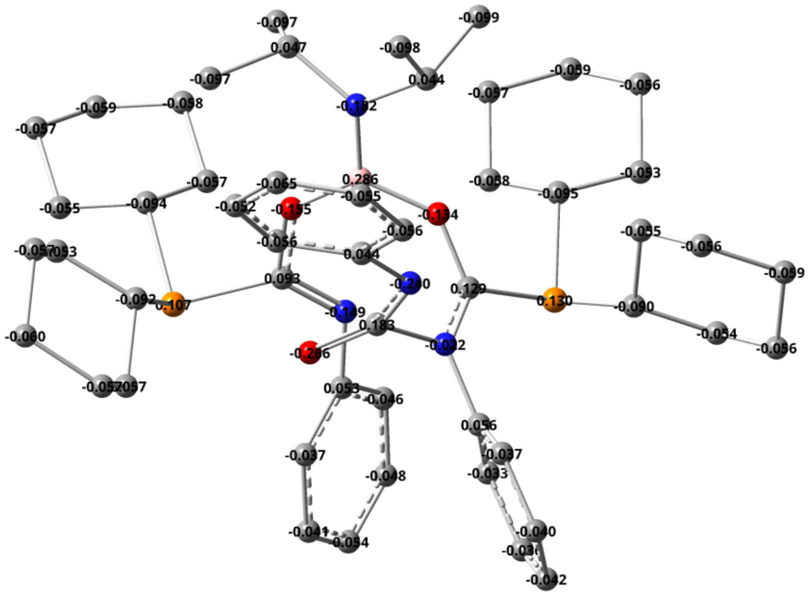

FIG. S127. OPTIMIZED STRUCTURE **2D_I5**

P	-3.24229800	-1.08687600	1.34779600
P	2.96322400	-1.44963500	0.27262300
O	-0.63977000	1.75560000	1.50986500
O	0.94570400	-0.07421400	-0.94208000
O	-1.49509100	-0.32417200	-0.60751000
N	1.47631900	0.80859800	1.09179100
N	0.90820900	2.79189000	0.00699300
N	-0.44411700	-1.52705100	1.03679900
N	-0.60041900	0.77161900	-2.57628600
C	0.40009900	1.92669200	0.84995900
C	0.13043000	3.87246700	-0.44343000
C	-1.28429900	3.92542700	-0.42032500
C	-1.96163900	5.01366400	-0.98321200
H	-3.05136300	5.03068500	-0.96188700
C	-1.25679100	6.07703800	-1.56823100
H	-1.79068000	6.92097100	-2.00137100
C	0.14728200	6.03790800	-1.58453900
H	0.70995200	6.85479100	-2.03494700
C	0.83065400	4.94921400	-1.03463800
H	1.91810400	4.89807700	-1.06182100
C	1.62959000	-0.13813900	0.19173600
C	2.24486700	0.91072900	2.30338200
C	2.42226600	-0.18202300	3.16374900
H	1.95751100	-1.13684000	2.94399700
C	3.19827600	-0.01865400	4.31809400
H	3.33159400	-0.86517400	4.98854600
C	3.77555200	1.22287800	4.61927500
H	4.37214000	1.34279700	5.52155900
C	3.56969600	2.31456200	3.76138400

H	4.00658100	3.28392500	3.99315700
C	2.80291700	2.16531700	2.60100800
H	2.63438900	2.99119300	1.91346500
C	-1.51568300	-0.96663100	0.60727800
C	-0.34979200	-2.05325200	2.33783200
C	0.25097400	-3.31449800	2.51734000
H	0.55409900	-3.88338200	1.64150700
C	0.44954300	-3.81614600	3.80892000
H	0.90546600	-4.79619400	3.93988900
C	0.07585900	-3.05853100	4.93071600
H	0.24182800	-3.44794600	5.93345000
C	-0.49664300	-1.79006500	4.75098500
H	-0.76721400	-1.18456300	5.61434700
C	-0.70644500	-1.28341300	3.46455600
H	-1.08580900	-0.27648300	3.31110100
C	0.53531800	1.45381100	-3.25653600
H	1.27457700	1.63717800	-2.46965100
C	1.15563300	0.54923800	-4.33602900
H	1.48902400	-0.40160800	-3.90307500
H	2.01960100	1.04333300	-4.80035700
H	0.42751400	0.32977900	-5.12960800
C	0.13275000	2.82390800	-3.83064600
H	-0.49237800	2.72978700	-4.72900900
H	1.04002100	3.36933500	-4.11811800
H	-0.39970300	3.42267600	-3.08597500
C	-1.87481700	0.66567100	-3.33760200
H	-1.67261800	1.11953800	-4.31481200
C	-3.01117000	1.46292300	-2.67807800
H	-3.26207600	1.03423200	-1.70275700
H	-3.90808500	1.43679100	-3.31166800
H	-2.71294700	2.50624800	-2.53022600
C	-2.25515600	-0.80397900	-3.58627700
H	-1.43823000	-1.33357900	-4.09201200
H	-3.15227100	-0.86258000	-4.21695800
H	-2.46667300	-1.31493500	-2.64154900
C	-4.01616900	-1.92946700	-0.16545800
H	-4.03776800	-1.22488700	-1.01134500
C	-3.18752700	-3.17125800	-0.57020100
H	-2.17243200	-2.87634100	-0.86700000
H	-3.08081000	-3.83104300	0.30615300
C	-3.85308100	-3.94902800	-1.72243700
H	-3.86080900	-3.31714800	-2.62455500
H	-3.25181700	-4.83825100	-1.96165000
C	-5.29728200	-4.35111500	-1.37812700
H	-5.76283300	-4.86797900	-2.22935100
H	-5.28598000	-5.06278700	-0.53683500
C	-6.12294900	-3.11571000	-0.98048900
H	-7.14436200	-3.40947800	-0.69927700
H	-6.21034500	-2.44034500	-1.84711800
C	-5.45936100	-2.35977100	0.18677700
H	-6.06672200	-1.48929900	0.46246700
H	-5.42350200	-3.01500700	1.07169300
C	-3.71651100	0.74632300	1.19303900
H	-2.96771200	1.20632700	0.53367200
C	-5.11312600	1.02200300	0.60386600
H	-5.21863700	0.56433800	-0.38892200
H	-5.88662600	0.57966800	1.25221800

C	-5.35159600	2.54240500	0.50268000
H	-4.60886800	2.97151200	-0.18875400
H	-6.34374100	2.73622400	0.06929400
C	-5.22184300	3.22089800	1.87846900
H	-6.02678100	2.85188800	2.53529000
H	-5.36173200	4.30705300	1.78100300
C	-3.85785500	2.91247400	2.52038300
H	-3.05560900	3.38170100	1.93362300
H	-3.80575500	3.34371400	3.53048100
C	-3.59349700	1.39698800	2.58958900
H	-4.31561600	0.91859600	3.27116100
H	-2.58977900	1.22491200	2.99414800
C	4.38189600	-0.20856000	0.04045400
H	4.20645500	0.50949000	0.85659400
C	4.36540900	0.57819000	-1.28471200
H	3.39162400	1.06664700	-1.42212900
H	4.50983300	-0.11051000	-2.13130100
C	5.48652800	1.63664900	-1.29564800
H	5.48757500	2.16656200	-2.25880000
H	5.27427500	2.38665200	-0.51754500
C	6.86194700	0.99896600	-1.02881100
H	7.10816100	0.30989400	-1.85340700
H	7.64342800	1.77159500	-1.01489800
C	6.86187400	0.21847500	0.29865100
H	6.69182800	0.91908300	1.13177200
H	7.84094300	-0.25150300	0.46857500
C	5.75639200	-0.85617000	0.31667700
H	5.73618100	-1.36979900	1.28760900
H	5.98120800	-1.61616100	-0.44591800
C	2.67712100	-2.20593000	-1.42580300
H	2.47868500	-1.39626800	-2.14132600
C	3.90404600	-3.00434600	-1.91649700
H	4.78535100	-2.35520900	-1.99162500
H	4.14639700	-3.79627000	-1.18974700
C	3.61662100	-3.63345000	-3.29578600
H	3.47182700	-2.82381800	-4.02929500
H	4.49028000	-4.21221100	-3.62759700
C	2.36085300	-4.52355200	-3.27166500
H	2.15862600	-4.92025500	-4.27656000
H	2.54502500	-5.39001400	-2.61608100
C	1.14347100	-3.74501000	-2.74232400
H	0.26455200	-4.40236500	-2.67728700
H	0.88703400	-2.93777900	-3.44694700
C	1.44100500	-3.13335500	-1.36191000
H	1.64796600	-3.94292300	-0.64308600
H	0.57061700	-2.59023100	-0.97747500
B	-0.40044900	0.15903300	-1.32907700
H	-1.83415100	3.10678000	0.03234400

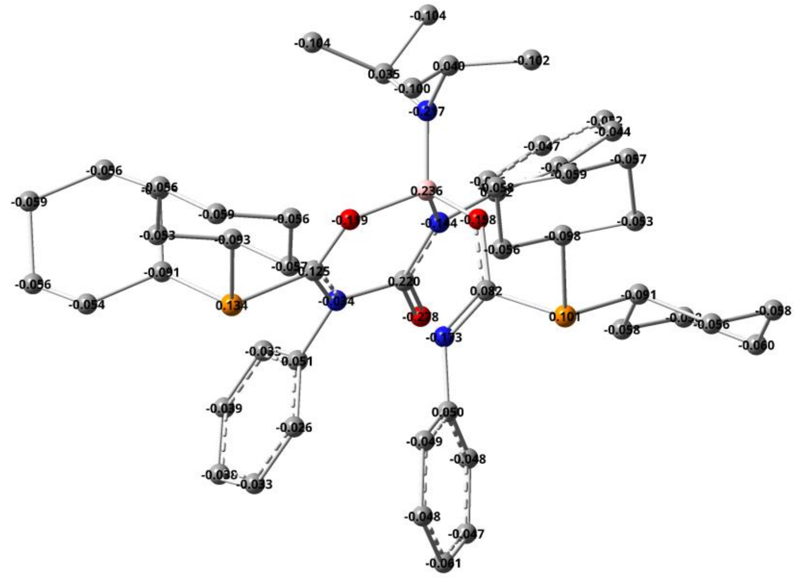

FIG. S128. OPTIMIZED STRUCTURE **2D**

P	3.20667900	0.77180400	1.53321300
P	-3.14211500	0.79882700	0.91134400
O	-0.58911200	-3.53326500	0.03008200
O	-0.99120200	0.42562500	-0.70446000
O	1.44714000	0.39980400	-0.56176900
N	-1.71266900	-1.53429800	0.23763900
N	0.19312400	-1.71524100	-1.19804300
N	0.62736300	-0.35589100	1.43480300
N	0.42181900	0.24255900	-2.76654500
C	-0.62281400	-2.35242100	-0.31189300
C	1.08061900	-2.53726000	-1.97762200
C	2.38375400	-2.08681100	-2.24097100
C	3.22361800	-2.81760400	-3.08911400
H	4.22848900	-2.44901500	-3.28948500
C	2.77830600	-4.00991100	-3.67576700
H	3.43291700	-4.57824900	-4.33375100
C	1.47888600	-4.46349600	-3.40436300
H	1.11706600	-5.38657800	-3.85393900
C	0.63048300	-3.73288600	-2.56451600
H	-0.37589800	-4.08775300	-2.36367700
C	-1.82021200	-0.20109700	0.04524600
C	-2.61621400	-2.22638400	1.13682300
C	-2.60058600	-1.92985700	2.50336900
H	-1.88800000	-1.21120300	2.89522300
C	-3.50859800	-2.58061000	3.35047900
H	-3.50110600	-2.35261000	4.41418100
C	-4.40626000	-3.52387500	2.83361000
H	-5.10664300	-4.02905900	3.49600300
C	-4.39516100	-3.82850500	1.46271500
H	-5.08146000	-4.57078900	1.06027100
C	-3.49754600	-3.17920000	0.60896700
H	-3.46967700	-3.40501000	-0.45432700
C	1.56706900	0.22644500	0.77114200
C	0.66695000	-0.52583600	2.83115100
C	0.48693100	0.56471400	3.70621000
H	0.41107000	1.56740200	3.29016900
C	0.40635800	0.35182500	5.08735300

H	0.27280600	1.20298000	5.75353300
C	0.49983400	-0.94566300	5.61402200
H	0.43766100	-1.10748800	6.68848100
C	0.66752500	-2.03322600	4.74213200
H	0.73343500	-3.04549800	5.13822600
C	0.73900400	-1.83125400	3.35918800
H	0.83859800	-2.67053900	2.67395000
C	-0.54095400	-0.34249800	-3.72015500
H	-1.00666600	-1.18736500	-3.19422700
C	-1.67173300	0.63779400	-4.10744000
H	-2.14436400	1.05230500	-3.20948400
H	-2.43868200	0.12733900	-4.70678500
H	-1.28794100	1.47463200	-4.70722500
C	0.15448500	-0.91124500	-4.97335900
H	0.63723800	-0.11304400	-5.55474800
H	-0.57761800	-1.40195000	-5.62946800
H	0.91754700	-1.64338200	-4.68871600
C	1.12858800	1.44784300	-3.24341400
H	0.79480500	1.60614100	-4.27841000
C	2.65415000	1.24192000	-3.31289500
H	3.06777500	1.05200800	-2.31681700
H	3.14688300	2.13260700	-3.72884600
H	2.88667900	0.38057900	-3.95040500
C	0.74107800	2.71725000	-2.46070900
H	-0.34443600	2.86895900	-2.50436200
H	1.23443800	3.60293800	-2.88573000
H	1.03134500	2.62979400	-1.40876300
C	3.51321900	2.27143800	0.43328700
H	3.17724600	2.02384900	-0.58243400
C	2.63742700	3.42401700	0.97701500
H	1.57955600	3.12468800	0.95715400
H	2.89665200	3.60271000	2.03300000
C	2.83244600	4.72501300	0.17614700
H	2.46832200	4.57650000	-0.85140200
H	2.22554800	5.52878900	0.61754000
C	4.31557500	5.13141700	0.13392000
H	4.44500700	6.04404800	-0.46516200
H	4.65863100	5.36590500	1.15481200
C	5.17345500	3.98985300	-0.43863400
H	6.23510200	4.27556900	-0.45235600
H	4.87677000	3.80445500	-1.48351600
C	4.99571000	2.68888200	0.36987400
H	5.59044500	1.89043100	-0.09314300
H	5.38645300	2.83647300	1.39007500
C	4.27701100	-0.59865300	0.77822500
H	4.38336300	-0.42146100	-0.30553700
C	5.67266800	-0.57371000	1.44724500
H	6.16198000	0.39742400	1.30305200
H	5.53696300	-0.70015600	2.53309900
C	6.57452200	-1.70285000	0.91237900
H	6.74668200	-1.54977200	-0.16570900
H	7.55742500	-1.65561400	1.40320300
C	5.92426400	-3.07922400	1.13442300
H	5.83983700	-3.26552000	2.21743600
H	6.55925200	-3.87567700	0.72060600
C	4.52525700	-3.12557900	0.49792600
H	4.61862100	-3.04548500	-0.59543300

H	4.03799200	-4.08971400	0.70045600
C	3.62700100	-1.98354200	1.00724300
H	3.44438800	-2.11131700	2.08614200
H	2.65017300	-2.04150300	0.51278900
C	-4.63959500	0.11909600	-0.02453000
H	-4.55903600	-0.96503600	0.14320100
C	-4.64344000	0.36101300	-1.54526500
H	-3.71329900	-0.01777800	-1.98816400
H	-4.68104100	1.44013600	-1.75646400
C	-5.85781900	-0.32639300	-2.20148700
H	-5.86780700	-0.11267700	-3.27961000
H	-5.75406500	-1.41792500	-2.09115400
C	-7.17687800	0.12378200	-1.54800500
H	-7.32129300	1.20144700	-1.72883100
H	-8.02723600	-0.39437400	-2.01282400
C	-7.15799700	-0.13685300	-0.03060000
H	-7.08620500	-1.22120400	0.15108000
H	-8.09581800	0.20462600	0.42967400
C	-5.96123500	0.56372400	0.64251500
H	-5.93108400	0.32678400	1.71454900
H	-6.08661500	1.65239500	0.55532200
C	-2.73254800	2.47239800	0.15882900
H	-2.47177000	2.34626000	-0.90204200
C	-3.92610700	3.44553100	0.28121400
H	-4.78171200	3.07893600	-0.29949800
H	-4.25033500	3.50141200	1.33321100
C	-3.54269900	4.85558100	-0.20980300
H	-3.30460600	4.80709500	-1.28456800
H	-4.40491100	5.52902900	-0.10264000
C	-2.32706200	5.40749700	0.55376300
H	-2.05704400	6.40219300	0.17212900
H	-2.59335800	5.53233500	1.61594100
C	-1.13235700	4.44633800	0.43821100
H	-0.28163300	4.82077300	1.02392700
H	-0.80072000	4.39862300	-0.60943900
C	-1.50388300	3.03159700	0.91880400
H	-1.74091500	3.06377300	1.99454500
H	-0.65192900	2.35261700	0.79525800
B	0.32742100	-0.16992000	-1.35955900
H	2.72516200	-1.16046000	-1.79280700

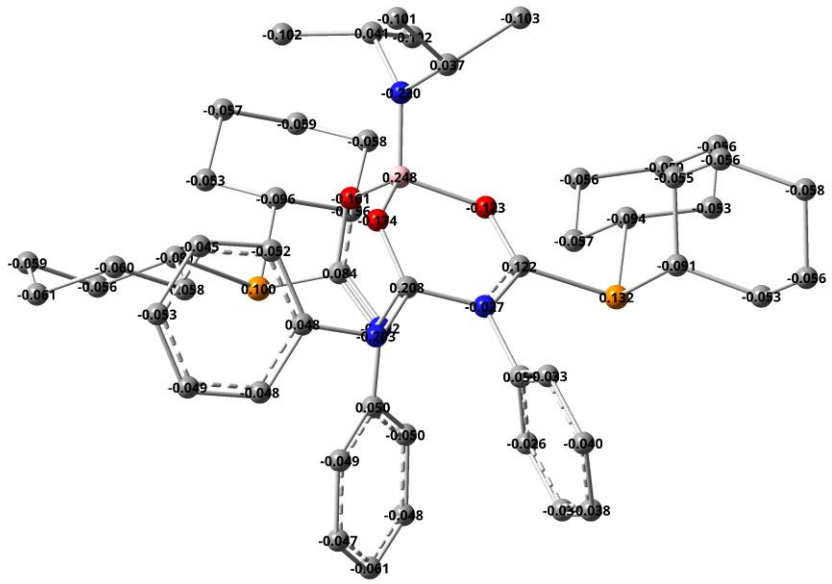

FIG. S129. OPTIMIZED STRUCTURE **2D’**

P	3.28776800	1.03091700	1.27944900
P	-3.24949400	0.56261900	1.02056900
O	-1.09833100	0.67495100	-0.64003900
O	1.35083300	0.84672700	-0.66061300
N	-1.54515600	-1.46526200	0.07339600
N	0.78013100	-0.25807000	1.25530100
N	0.17707800	0.81620100	-2.79061900
C	-0.38372400	-2.00871500	-0.58941500
C	-1.82313400	-0.14071300	0.03583400
C	-2.30732200	-2.35891700	0.92566200
C	-2.21023500	-2.23497800	2.31484700
H	-1.54703900	-1.49272600	2.74818100
C	-2.96822400	-3.09077400	3.12565000
H	-2.89654100	-3.00170700	4.20750300
C	-3.80105700	-4.05809300	2.54749800
H	-4.38533100	-4.72221200	3.18158200
C	-3.87499000	-4.18070900	1.15028800
H	-4.51067300	-4.93988300	0.69918700
C	-3.12451500	-3.32943000	0.33287300
H	-3.15343300	-3.41769400	-0.75073000
C	1.61397200	0.48419300	0.61252400
C	0.97804100	-0.65116300	2.59241700
C	0.76553000	0.25009000	3.65480300
H	0.53898100	1.29007500	3.42684800
C	0.84692000	-0.19497400	4.97991600
H	0.68822700	0.51176200	5.79320700
C	1.13432400	-1.53913800	5.26237200
H	1.19747000	-1.88165500	6.29349400
C	1.33584000	-2.43854600	4.20325900
H	1.55443300	-3.48516000	4.41066200
C	1.24856100	-2.00510900	2.87576500
H	1.37308700	-2.69887200	2.04764100
C	-0.89092600	0.30384300	-3.66529500
H	-1.39219900	-0.48988300	-3.09245500
C	-1.95715300	1.37001500	-4.00158300
H	-2.34823400	1.82152700	-3.08207800
H	-2.79267400	0.92075300	-4.55633300

H	-1.53947900	2.17161200	-4.62566300
C	-0.32676100	-0.34805800	-4.94387500
H	0.20683300	0.38947400	-5.55932700
H	-1.13297100	-0.77414900	-5.55724900
H	0.37740400	-1.14562400	-4.67960700
C	0.93575900	1.98014400	-3.28342700
H	0.57936500	2.16599000	-4.30672900
C	2.44446300	1.68153900	-3.39308800
H	2.86651000	1.46042400	-2.40785500
H	2.98029400	2.54074400	-3.82172600
H	2.60782900	0.80854200	-4.03702100
C	0.65047500	3.25951800	-2.47012400
H	-0.42268900	3.48749600	-2.48549800
H	1.19594500	4.11890300	-2.88561800
H	0.95598500	3.12533700	-1.42728800
C	3.32387000	2.74004300	0.48438300
H	3.00393700	2.63529000	-0.56232600
C	2.31518000	3.64629300	1.22508800
H	1.30495900	3.22092500	1.14065200
H	2.56709100	3.66153100	2.29793300
C	2.32638900	5.08629100	0.67750500
H	1.96364100	5.08123100	-0.36162800
H	1.62976900	5.71033500	1.25580800
C	3.74279700	5.68601900	0.71596400
H	3.73902600	6.70103400	0.29362100
H	4.07214800	5.77553600	1.76404400
C	4.73249200	4.79111200	-0.05004400
H	5.74720500	5.21202300	-0.00397000
H	4.44470300	4.76136000	-1.11332800
C	4.73857900	3.35411200	0.50798400
H	5.42668100	2.73557900	-0.08349800
H	5.11970400	3.36470400	1.54226100
C	4.35241200	0.00677300	0.08905000
H	4.36925900	0.52006200	-0.88665100
C	5.79082500	-0.07643500	0.65300000
H	6.21200500	0.92672500	0.80268200
H	5.74745800	-0.54992000	1.64666400
C	6.70804400	-0.90435800	-0.26747400
H	6.79396100	-0.39859900	-1.24320100
H	7.72033600	-0.95011700	0.15994100
C	6.14315800	-2.32013800	-0.47619500
H	6.14601200	-2.85393200	0.48829100
H	6.78562900	-2.89314800	-1.16039900
C	4.70439000	-2.26193500	-1.01620500
H	4.71195400	-1.82017800	-2.02607500
H	4.28669200	-3.27130000	-1.11517400
C	3.78730300	-1.41731700	-0.11301000
H	3.68805900	-1.90732900	0.86892300
H	2.78574700	-1.37784300	-0.55543000
C	-4.64367600	-0.14286400	-0.05058700
H	-4.43220500	-1.22283600	-0.04666700
C	-4.65806800	0.33355400	-1.51462300
H	-3.68223400	0.14731000	-1.98150600
H	-4.82765500	1.41987800	-1.55493100
C	-5.77009200	-0.38094000	-2.30836100
H	-5.79095700	-0.00343200	-3.34047700
H	-5.53676000	-1.45651400	-2.36449600

C	-7.14269100	-0.19545000	-1.63641600
H	-7.41332200	0.87283900	-1.65581400
H	-7.91811500	-0.73127600	-2.20140600
C	-7.11345000	-0.68362600	-0.17640600
H	-6.91643100	-1.76760100	-0.15978200
H	-8.09142400	-0.52799800	0.30034300
C	-6.01730700	0.03621800	0.63409200
H	-5.97448600	-0.35933400	1.65781100
H	-6.26839700	1.10413200	0.71045000
C	-3.02373700	2.35288100	0.49673800
H	-2.77182400	2.38634500	-0.57309100
C	-4.29700200	3.19030800	0.74493000
H	-5.13499700	2.80649300	0.14931900
H	-4.59124200	3.11188400	1.80402800
C	-4.05179600	4.67007800	0.38632200
H	-3.84872900	4.74442700	-0.69415800
H	-4.96315400	5.25296600	0.58155700
C	-2.86113700	5.25426500	1.16655500
H	-2.68592000	6.29707400	0.86676600
H	-3.10498100	5.26615700	2.24129100
C	-1.59335500	4.41126400	0.94609900
H	-0.76292600	4.80516700	1.54783900
H	-1.28270400	4.47804800	-0.10784000
C	-1.83861200	2.93503300	1.30637100
H	-2.07026000	2.85367700	2.38070400
H	-0.93273200	2.34570900	1.12309900
B	0.23487200	0.28774400	-1.43013600
O	0.23964800	-1.20944500	-1.43890900
N	-0.12409100	-3.24088500	-0.33251700
C	0.92903000	-3.94234300	-0.94475300
C	1.51367400	-4.97829500	-0.18346300
C	1.37165400	-3.74165800	-2.27322600
C	2.52417900	-5.78004900	-0.72364200
H	1.15092500	-5.13879200	0.83008600
C	2.36349100	-4.56763100	-2.81439900
H	0.93484500	-2.94803200	-2.87098600
C	2.95101800	-5.58447100	-2.04651300
H	2.96930300	-6.56685400	-0.11649600
H	2.68804800	-4.40573100	-3.84144700
H	3.72941900	-6.21482600	-2.47247600

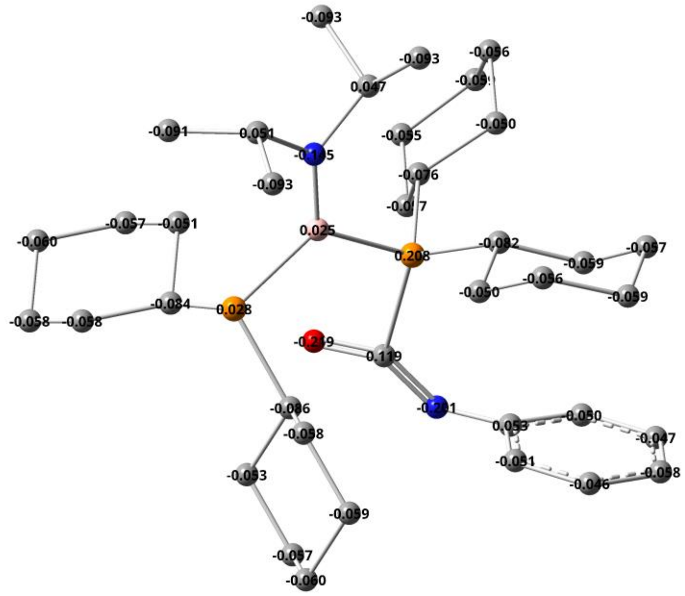

FIG. S130. OPTIMIZED STRUCTURE **2D_TS1_A**

P	-0.51688200	-0.73672200	-0.20282700
P	2.03732400	1.41174000	0.76319600
N	1.45967400	-1.17479800	1.98019300
C	3.13368400	1.06825000	-0.75524800
H	2.57802900	1.42993500	-1.62656000
C	4.45543600	1.85508500	-0.62790200
H	4.24513900	2.92361100	-0.47963600
H	5.00058500	1.51532800	0.26799100
C	-0.19444900	-2.44830000	-1.00899100
H	0.80185400	-2.73537400	-0.63845100
C	0.68491000	2.62546100	0.22175400
H	-0.20040000	2.05433600	-0.09944300
C	-1.88584600	-0.90498800	1.09680500
H	-1.58937900	-1.76401100	1.71871100
C	0.30054500	3.45146200	1.47235900
H	-0.01834200	2.78210800	2.28505800
H	1.19975900	3.97435800	1.83471700
C	0.91736700	-2.55567900	2.11562700
H	0.17574800	-2.65778500	1.32458700
C	-1.20054400	-3.54432300	-0.58815200
H	-1.35328000	-3.57304100	0.49961100
H	-2.17650100	-3.31937700	-1.04030700
C	1.09419600	3.55349200	-0.93678300
H	2.02527700	4.08027600	-0.66960600
H	1.29358700	2.95999100	-1.83663800
C	-1.84360800	0.35753600	1.98935400
H	-0.82676100	0.51484500	2.37678400
H	-2.09857900	1.23326500	1.37669300
C	2.43088700	-0.85647800	3.09221700
H	2.45276000	-1.76479500	3.70450300
C	-0.10249700	-2.42114700	-2.55288500
H	-1.08614700	-2.15537800	-2.96828600
H	0.60376400	-1.65333500	-2.87979400
C	-4.28701600	-1.30238900	1.78371500
H	-5.30251500	-1.48089500	1.40395000
H	-4.00790000	-2.18257800	2.38706400
C	3.41116800	-0.42950800	-0.99614600

H	3.91114000	-0.87084300	-0.12149000
H	2.45695900	-0.95500300	-1.13560000
C	3.87470200	-0.62615100	2.62418500
H	4.21860800	-1.45731600	1.99763200
H	4.52721500	-0.56608000	3.50614300
H	3.96699200	0.31041500	2.06461000
C	-0.39147100	5.40313100	0.00564700
H	-1.20371800	6.10511400	-0.23160100
H	0.47428000	6.00876900	0.32000600
C	-3.31894900	-1.15964900	0.59232700
H	-3.64052800	-0.31939600	-0.02864500
H	-3.36241900	-2.05725700	-0.03275400
C	5.34014000	1.65320200	-1.87399000
H	4.82678800	2.07870700	-2.75109700
H	6.28605700	2.20212300	-1.75755000
C	-0.01018900	4.58515100	-1.24115200
H	0.32260000	5.25371000	-2.04806200
H	-0.89684500	4.05412200	-1.61964800
C	-0.73068000	-4.92486200	-1.08645800
H	0.23994700	-5.16585200	-0.62346200
H	-1.44310500	-5.69605800	-0.76030500
C	-0.58744500	-4.93199600	-2.61765700
H	-1.58233900	-4.79257200	-3.07009600
H	-0.21184500	-5.90549000	-2.96291300
C	-4.26161700	-0.04514500	2.66946600
H	-4.94310100	-0.16145800	3.52400400
H	-4.62109100	0.81103700	2.07909800
C	0.18750200	-2.79499900	3.45115100
H	0.87582100	-2.85648100	4.30308600
H	-0.35474200	-3.74781100	3.39695700
H	-0.53657800	-1.99681600	3.65024900
C	4.29524100	-0.62826100	-2.24228200
H	4.50098200	-1.69935900	-2.38918100
H	3.73802000	-0.28201600	-3.12625100
C	-0.80266900	4.48179100	1.16822200
H	-1.73408100	3.95525100	0.90547300
H	-1.02011200	5.07346400	2.06961300
C	1.90310500	0.27306000	3.99229500
H	1.90117000	1.22733100	3.45542500
H	2.54970400	0.36923700	4.87484000
H	0.88463500	0.04903100	4.33295500
C	-2.83381000	0.24445300	3.16350100
H	-2.50974800	-0.56998100	3.83251100
H	-2.80804100	1.17113500	3.75432900
C	0.34297000	-3.79917700	-3.08139400
H	0.39128900	-3.76676900	-4.17863600
H	1.36661000	-4.00128900	-2.72520000
C	2.00982000	-3.60817300	1.85628900
H	2.48882500	-3.43192000	0.88576200
H	1.56463600	-4.61132200	1.85205800
H	2.78590900	-3.58938900	2.63215800
C	5.61326100	0.15890800	-2.12510500
H	6.20331800	-0.24546800	-1.28585000
H	6.21843000	0.02795500	-3.03372700
B	1.07838800	-0.28511000	0.94800800
C	-0.48468600	0.47482000	-1.95247800
O	0.71556600	0.46126700	-2.25426800

N	-1.55462900	0.87978300	-2.53461900
C	-2.83242900	1.01627500	-2.01369500
C	-3.12402700	1.96666200	-1.00771000
C	-3.90427600	0.28436300	-2.57670000
C	-4.43669200	2.15705700	-0.56034500
H	-2.30979400	2.56184900	-0.60402500
C	-5.21254400	0.47409400	-2.11931700
H	-3.68653000	-0.42917300	-3.36897000
C	-5.48977000	1.40797700	-1.10621200
H	-4.63597200	2.89561800	0.21559700
H	-6.02263100	-0.10807700	-2.55665900
H	-6.51013400	1.55607500	-0.75805700

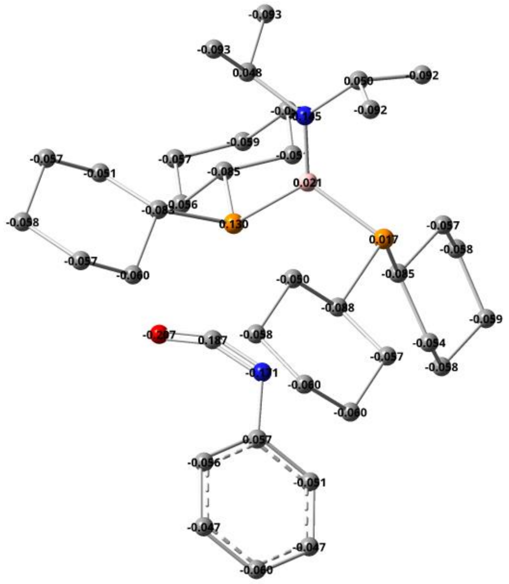

FIG. S131. OPTIMIZED STRUCTURE **2D_TS1_B**

P	-0.90362600	-0.42114400	-0.70040400
P	0.22256400	1.27391600	2.01694000
N	-2.42281700	-0.06244600	1.85917700
C	1.62455800	0.00722800	1.83444200
H	1.85355800	-0.08734100	0.76227500
C	2.88576200	0.47595300	2.59745100
H	3.21115600	1.46162000	2.24769300
H	2.62768400	0.58925700	3.66339100
C	-1.14545700	-2.31229000	-0.57378900
H	-1.47466000	-2.48364300	0.46323600
C	0.51014700	2.49851800	0.59668500
H	0.31458600	1.98185800	-0.35819300
C	-2.33372500	0.27325800	-1.72953000
H	-3.24651600	-0.29602100	-1.49116200
C	-0.47653200	3.67611300	0.76214300
H	-1.51231100	3.31129400	0.79804600
H	-0.28225700	4.16267500	1.73158400
C	-3.51692700	-0.88509300	1.26779800
H	-3.16170000	-1.17057700	0.27668500
C	-2.20151300	-2.93191500	-1.51106400
H	-3.16750400	-2.41358000	-1.43058100
H	-1.86409600	-2.82487800	-2.55193700
C	1.95310600	3.02945500	0.53231900
H	2.21548600	3.50485200	1.49185100

H	2.64314500	2.19453400	0.37420100
C	-2.57577100	1.74613400	-1.33833700
H	-2.75505100	1.82739100	-0.25766600
H	-1.66454000	2.32308200	-1.55340500
C	-2.75937400	0.41238600	3.24847000
H	-3.75853100	0.00906100	3.45037600
C	0.19134100	-3.06747600	-0.74566400
H	0.51473300	-2.98411100	-1.79093700
H	0.97178100	-2.60877900	-0.12731700
C	-3.29188200	0.75161700	-4.02673700
H	-3.10635100	0.66987700	-5.10694300
H	-4.19062400	0.15057400	-3.81027000
C	1.19948500	-1.37352600	2.38094300
H	0.92199100	-1.27688900	3.44285600
H	0.30036000	-1.73771300	1.85995300
C	-1.81920000	-0.17009100	4.31446000
H	-1.73715400	-1.25819600	4.20584200
H	-2.21941200	0.05191900	5.31302800
H	-0.82065100	0.27280600	4.23548000
C	1.12845500	5.22486900	-0.45535900
H	1.23503900	5.92882800	-1.29313200
H	1.37084900	5.78102600	0.46480000
C	-2.09223700	0.17319900	-3.25131500
H	-1.18451200	0.73733800	-3.50717900
H	-1.90548200	-0.86342400	-3.55179000
C	4.03688300	-0.53766100	2.44902400
H	4.32112100	-0.60977100	1.38798000
H	4.92161500	-0.18002200	2.99565600
C	2.11175100	4.05121700	-0.60934100
H	3.14710100	4.42259400	-0.63847000
H	1.92427100	3.54095200	-1.56779400
C	-2.39318200	-4.42694400	-1.17980200
H	-2.80250900	-4.51490200	-0.16030300
H	-3.13568000	-4.86580300	-1.86172900
C	-1.06586800	-5.20339500	-1.26236400
H	-0.71732600	-5.20831200	-2.30730600
H	-1.22210200	-6.25174200	-0.97132100
C	-3.55329700	2.21727300	-3.63716300
H	-4.42726100	2.61011100	-4.17566400
H	-2.68804000	2.82892200	-3.93902600
C	-4.81658100	-0.08305400	1.07701500
H	-5.26838000	0.20688100	2.03418500
H	-5.54769900	-0.69949700	0.53838600
H	-4.63270400	0.82427900	0.49174300
C	2.34026700	-2.40266600	2.24822200
H	2.01531000	-3.37694500	2.64272000
H	2.57274000	-2.54698700	1.18169600
C	-0.31994500	4.71211600	-0.36797900
H	-0.59249600	4.24678500	-1.32892200
H	-1.01613800	5.54886600	-0.20970300
C	-2.87158200	1.94310700	3.32109100
H	-1.88828300	2.41119600	3.20562200
H	-3.28258100	2.23419100	4.29720500
H	-3.54049800	2.31863100	2.53677300
C	-3.75795300	2.35044600	-2.11790400
H	-4.68433200	1.82652500	-1.83008100
H	-3.88592400	3.40537200	-1.83582000

C	0.01486800	-4.55218800	-0.37987200
H	0.97132400	-5.08188200	-0.49352900
H	-0.27351000	-4.63646500	0.68082600
C	-3.75507300	-2.18065400	2.06539100
H	-2.81690500	-2.73104700	2.20340800
H	-4.45628800	-2.82224900	1.51691900
H	-4.18752600	-1.98411100	3.05480300
C	3.61601100	-1.92498100	2.96369100
H	3.42517500	-1.86801000	4.04798300
H	4.42653000	-2.65330100	2.81743300
B	-1.22379200	0.23000300	1.17739200
C	1.13394100	-0.40338000	-2.10222500
C	3.39629300	-0.07969700	-1.57121700
C	3.86584600	-1.40658200	-1.67372500
C	4.32649100	0.97288100	-1.47092000
C	5.24049400	-1.66859900	-1.67136900
H	3.14887300	-2.22241800	-1.74251500
C	5.69965700	0.70005500	-1.47443600
H	3.96293900	1.99443900	-1.40224800
C	6.16667800	-0.61944500	-1.56829100
H	5.58756400	-2.69828300	-1.74518200
H	6.40689900	1.52479800	-1.40015000
H	7.23474700	-0.82722700	-1.56155900
N	2.01632400	0.21372500	-1.44559200
O	0.73446300	-1.00451300	-3.07341700

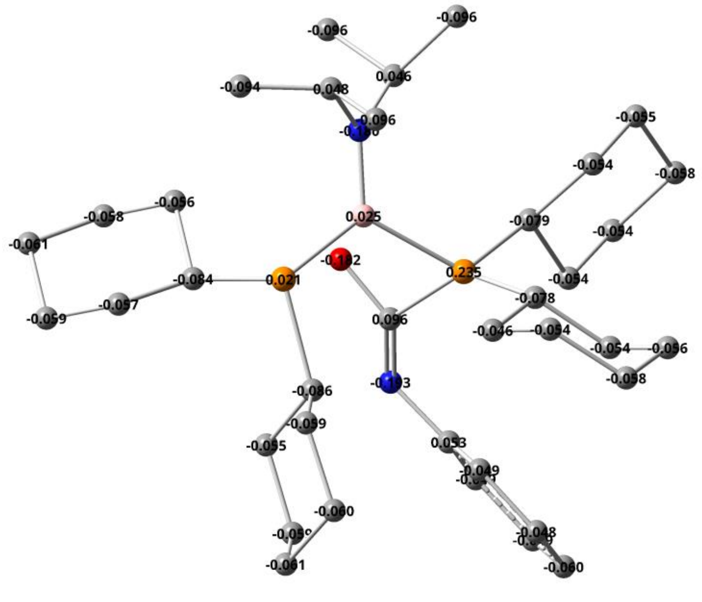

FIG. S132. OPTIMIZED STRUCTURE **2D_TS2_A**

P	-0.99920300	-0.60569300	0.02600600
P	2.21207800	0.24116700	0.87354700
N	1.31212500	-2.50904400	0.58736500
C	3.40369400	0.35020800	-0.60812300
H	2.88730400	0.96504500	-1.35639700
C	4.68848200	1.08102300	-0.15410500
H	4.44579900	2.00283500	0.39346100
H	5.23032200	0.43365200	0.55425200
C	-2.15536000	-1.79746300	-0.87332300
H	-1.47837100	-2.33791400	-1.55147200
C	1.37534800	1.96439600	0.78989400

H	0.29204100	1.77775600	0.75222300
C	-1.91547100	-0.21638800	1.63046000
H	-1.97700400	-1.24834400	2.02658300
C	1.68227000	2.69655600	2.11783100
H	1.40694400	2.05897200	2.96927400
H	2.77179700	2.84597400	2.18946700
C	0.72049600	-3.62897400	-0.18395900
H	-0.03475200	-3.17085800	-0.82729200
C	-2.84817500	-2.81697900	0.05308300
H	-2.11875800	-3.31175500	0.70239700
H	-3.56109300	-2.29536100	0.70777400
C	1.71480100	2.87075200	-0.41398500
H	2.80226400	3.02503500	-0.46385500
H	1.40837800	2.39212500	-1.35170900
C	-1.07421800	0.59750600	2.63118800
H	-0.04613800	0.21456000	2.67642300
H	-1.01809700	1.64190200	2.29410300
C	2.17333500	-2.95567500	1.72906200
H	2.05530200	-4.04501500	1.74678500
C	-3.19178800	-1.08419100	-1.77685800
H	-3.88415600	-0.48502300	-1.16970400
H	-2.68580600	-0.39430700	-2.45854200
C	-4.00426300	0.33090300	2.94386300
H	-5.01539000	0.75624200	2.87662000
H	-4.11786500	-0.71357600	3.27747900
C	3.78483000	-0.95528500	-1.32805400
H	4.35384500	-1.60840100	-0.65583500
H	2.88290000	-1.49025700	-1.63551900
C	3.68404500	-2.70260800	1.60299000
H	4.08087000	-3.14399000	0.68273200
H	4.19309500	-3.17268300	2.45668500
H	3.91164800	-1.63084900	1.61927800
C	1.35789500	4.95606600	1.02011600
H	0.83484300	5.92147800	1.07817700
H	2.43778400	5.17173200	1.07105400
C	-3.34754100	0.34470100	1.54799500
H	-3.31211200	1.37007300	1.16153100
H	-3.95986500	-0.23277200	0.84688600
C	5.58922400	1.40335800	-1.36234600
H	5.05959000	2.10952800	-2.02232800
H	6.50806200	1.90596200	-1.02606600
C	1.03585000	4.25327700	-0.30856200
H	1.35403100	4.87347000	-1.15900300
H	-0.05290800	4.13445000	-0.39938000
C	-3.62058300	-3.85999200	-0.77704600
H	-2.90818100	-4.42970200	-1.39512100
H	-4.11000700	-4.57907800	-0.10518100
C	-4.65613700	-3.17595900	-1.68683700
H	-5.41535000	-2.68181200	-1.05904900
H	-5.18257400	-3.92386700	-2.29591400
C	-3.16313800	1.10394200	3.97526000
H	-3.63047100	1.04642900	4.96801800
H	-3.13694600	2.16910600	3.69429900
C	0.03057500	-4.70910500	0.68284200
H	0.74963000	-5.43398800	1.08604500
H	-0.68367600	-5.27210000	0.06804700
H	-0.51137500	-4.26553500	1.52672700

C	4.65770000	-0.63386500	-2.55827700
H	4.92170000	-1.56531300	-3.08095200
H	4.07139000	-0.02505300	-3.26458100
C	0.97137200	4.05863300	2.20753300
H	-0.12095300	3.89988700	2.19872000
H	1.21384300	4.54839000	3.16199600
C	1.63080900	-2.42829100	3.07187400
H	1.78311500	-1.34504700	3.14605800
H	2.15708300	-2.90833400	3.90831400
H	0.55856600	-2.64507900	3.16421000
C	-1.72272800	0.56470300	4.02724200
H	-1.72916400	-0.47234400	4.39968200
H	-1.11527900	1.15362200	4.72822400
C	-3.98410200	-2.12756700	-2.59038800
H	-4.73406600	-1.61314400	-3.20699700
H	-3.29421800	-2.63520000	-3.28354300
C	1.76576900	-4.28107100	-1.10901400
H	2.18117300	-3.54254600	-1.80164000
H	1.30637900	-5.09254100	-1.69044500
H	2.58805000	-4.71172400	-0.52167000
C	5.93310300	0.12906400	-2.15526100
H	6.55864300	-0.52576000	-1.52638200
H	6.52848700	0.38175000	-3.04463800
B	0.92364100	-1.14782000	0.30974000
C	-0.25317600	0.22041800	-1.56832900
O	0.72637200	-0.61019300	-1.69704300
N	-0.63345300	1.16091600	-2.36034900
C	-1.73263400	1.98461000	-2.09823300
C	-2.04554800	2.50411500	-0.82301400
C	-2.54746100	2.35756700	-3.19252100
C	-3.14778400	3.35195500	-0.64737600
H	-1.39816800	2.27646500	0.02025600
C	-3.65124900	3.19385000	-3.00899800
H	-2.29170900	1.97167300	-4.17734900
C	-3.96204600	3.69687600	-1.73345300
H	-3.35836500	3.75707400	0.34230300
H	-4.26884400	3.46285200	-3.86465700
H	-4.81340700	4.36027600	-1.59469000

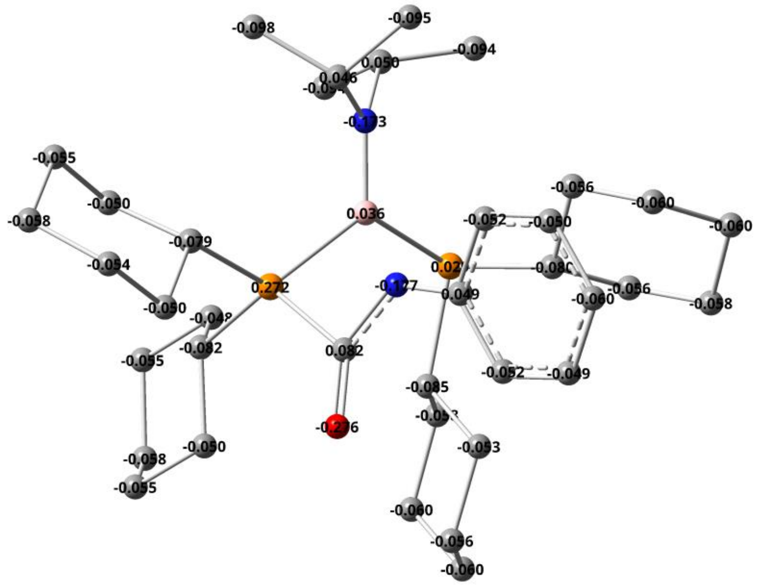

FIG. S133. OPTIMIZED STRUCTURE **2D_TS2_B**

P	-1.54984100	-0.23504900	-0.19847200
P	1.06909100	1.21375000	1.39215000
N	0.01797900	-1.39710800	2.13309000
C	2.75347700	0.65637300	0.70835100
H	2.64465300	0.67946100	-0.38407100
C	3.83160600	1.67932800	1.13101600
H	3.50671900	2.70724600	0.91752700
H	3.96910800	1.61542900	2.22242800
C	-2.61957900	-1.74390300	-0.55093700
H	-1.97343600	-2.59488900	-0.29960800
C	0.59062400	2.54346100	0.11232300
H	-0.43852200	2.30682100	-0.18796700
C	-2.80304800	1.15370000	-0.07578400
H	-3.76193900	0.61660400	-0.09529500
C	0.56839800	3.92537100	0.80812100
H	-0.05246500	3.87904100	1.71462400
H	1.58814900	4.16729700	1.14846200
C	-0.23110900	-2.81229600	1.75917600
H	-0.32264600	-2.80120700	0.66674600
C	-3.91063700	-1.83598200	0.29271000
H	-3.70928200	-1.68040700	1.36046100
H	-4.60895900	-1.04386900	-0.01640500
C	1.42004800	2.61263600	-1.18739000
H	2.47111900	2.83129800	-0.94903000
H	1.39302100	1.65113400	-1.70868900
C	-2.75402400	1.90569200	1.27348100
H	-2.77388200	1.18923100	2.10558100
H	-1.81350400	2.46542700	1.36178900
C	0.19779600	-1.17150600	3.60338700
H	-0.07152900	-2.13228300	4.05994400
C	-2.97374600	-1.85669400	-2.05303800
H	-3.62343000	-1.01453900	-2.33965600
H	-2.07106600	-1.78865300	-2.66946100
C	-4.02387700	3.07687300	-1.15475100
H	-4.02257100	3.78044900	-1.99887800
H	-4.96138400	2.50039900	-1.22134200
C	3.22381700	-0.76219100	1.05661900
H	3.39191000	-0.85209400	2.13699300
H	2.45130900	-1.48463100	0.77628400
C	1.62422200	-0.84910100	4.07338900
H	2.32791200	-1.62749100	3.76108500
H	1.63471300	-0.79487100	5.17123700
H	1.95738500	0.11810900	3.67919300
C	0.88361900	5.08704400	-1.43365300
H	0.48199600	5.85682300	-2.10823900
H	1.91932300	5.37903700	-1.19434400
C	-2.82662900	2.11568900	-1.28551800
H	-1.89734100	2.69661500	-1.32746600
H	-2.87468200	1.54894600	-2.22270700
C	5.16509100	1.38675500	0.41552500
H	5.02410200	1.52363500	-0.66881200
H	5.93039400	2.10936900	0.73464700
C	0.89258800	3.71415500	-2.12677600
H	1.51239900	3.74545100	-3.03426800
H	-0.12561200	3.45256300	-2.45242600
C	-4.60217500	-3.19225300	0.05228900

H	-3.93331100	-4.00494100	0.37536400
H	-5.51073200	-3.25908200	0.66698700
C	-4.94638900	-3.36316100	-1.43860200
H	-5.69583900	-2.60603800	-1.72060200
H	-5.40479900	-4.34633200	-1.61371600
C	-3.99322000	3.84306000	0.18092800
H	-4.86920000	4.50175300	0.26145500
H	-3.10349300	4.49092400	0.20558000
C	-1.50915300	-3.40720200	2.38116600
H	-1.38002800	-3.61233700	3.45207800
H	-1.75417700	-4.35984600	1.89310700
H	-2.35999400	-2.73026300	2.26732400
C	4.54031800	-1.06975100	0.31637900
H	4.86972900	-2.09185600	0.55320800
H	4.35832200	-1.03951300	-0.76777900
C	0.06206500	5.03347700	-0.13421600
H	-0.99162500	4.83342100	-0.38765700
H	0.08587300	6.00497100	0.38070300
C	-0.81007500	-0.13086000	4.12467700
H	-0.58141400	0.86213100	3.71971800
H	-0.76338000	-0.07521500	5.22055100
H	-1.83124200	-0.40835200	3.83326500
C	-3.94486000	2.87754500	1.37908600
H	-4.88020000	2.29515600	1.41388700
H	-3.88220600	3.43821100	2.32218400
C	-3.69761100	-3.19198400	-2.32177900
H	-3.96892700	-3.25423100	-3.38461100
H	-2.99539500	-4.01744500	-2.12335700
C	0.98235500	-3.70080500	2.10091100
H	1.89789300	-3.31079100	1.64596900
H	0.81020200	-4.71968200	1.72895400
H	1.13514100	-3.76552600	3.18640300
C	5.63616000	-0.05393600	0.68608800
H	5.88094200	-0.15618600	1.75636900
H	6.55818300	-0.26461100	0.12534900
B	-0.06853700	-0.35236900	1.15239200
C	-0.35143900	-0.23630900	-1.65983800
C	1.62862900	-1.58918800	-1.89735000
C	2.24156500	-0.89568000	-2.96866000
C	2.14789000	-2.84973400	-1.52573500
C	3.35061800	-1.44921500	-3.62044200
H	1.83272000	0.05831700	-3.28695800
C	3.25010800	-3.39771700	-2.18757800
H	1.66909600	-3.38554000	-0.70887900
C	3.86517100	-2.69738500	-3.23733500
H	3.81303100	-0.89988700	-4.43972800
H	3.63302200	-4.37011700	-1.88045800
H	4.72774000	-3.11880300	-3.75018200
N	0.54936000	-1.08837500	-1.14521800
O	-0.50461200	0.33251800	-2.75531700

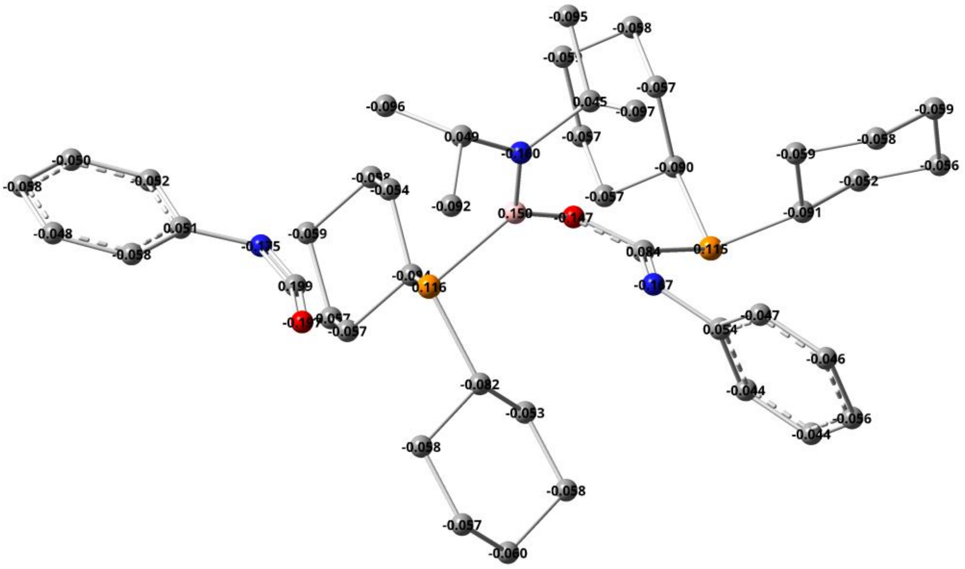

FIG. S134. OPTIMIZED STRUCTURE **2D_TS3_A1**

P	-3.14907500	-0.65957800	-1.26979800
P	1.79873500	0.82613100	-0.09618700
N	0.18409500	-0.21578800	2.24108100
C	2.02544100	-0.46232100	-1.44576700
H	1.07198600	-0.54191600	-1.99143000
C	3.14082300	-0.06110700	-2.43591000
H	2.89123000	0.89072400	-2.92405400
H	4.07141300	0.09411800	-1.87407700
C	-4.72150200	-0.58131400	-0.21899100
H	-5.11716500	0.42064600	-0.44780400
C	0.99626500	2.32777300	-0.93461200
H	0.29555700	2.71775500	-0.17930000
C	-2.61465400	-2.46511200	-1.28449600
H	-3.49161000	-2.94453000	-1.75551000
C	2.06220900	3.41239700	-1.22117000
H	2.61044200	3.65332700	-0.30407100
H	2.80044300	3.01251300	-1.93487600
C	-1.04221600	-0.83547200	2.81945400
H	-1.71105500	-1.02441500	1.97717900
C	-5.74890300	-1.60989100	-0.74448400
H	-5.83742900	-1.54149000	-1.83855200
H	-5.40304900	-2.62871100	-0.50876400
C	0.18801100	2.01282800	-2.21129600
H	0.85746100	1.58969800	-2.97505300
H	-0.58224400	1.25920700	-2.01055400
C	-1.43020700	-2.63332600	-2.26717800
H	-1.66118400	-2.13521300	-3.21914500
H	-0.54774700	-2.13420600	-1.84443900
C	1.22906900	0.14046400	3.24935700
H	0.79713900	-0.12758800	4.22140100
C	-4.59646900	-0.68300400	1.31461900
H	-4.22984900	-1.68079500	1.59554500
H	-3.86959800	0.04699400	1.68641300
C	-1.99764900	-4.68033300	-0.19595900
H	-1.77062600	-5.16670100	0.76386200
H	-2.88350800	-5.18905400	-0.61023900
C	2.33138300	-1.83035400	-0.79260500

H	3.21667200	-1.72145200	-0.15091000
H	1.49352500	-2.13083300	-0.14468400
C	2.49373800	-0.71238200	3.06798100
H	2.24757700	-1.77815500	3.15208900
H	3.23025200	-0.46282000	3.84320600
H	2.95936500	-0.54363500	2.09217600
C	0.60394200	4.36695600	-3.07569500
H	0.12625300	5.27851700	-3.46232400
H	1.28158200	3.99996100	-3.86400200
C	-2.33152000	-3.19395100	0.04450800
H	-1.48270100	-2.70551100	0.53858600
H	-3.19490100	-3.11576300	0.71637100
C	3.35877800	-1.15543700	-3.49763200
H	2.44444300	-1.26825500	-4.10356300
H	4.16308300	-0.85215900	-4.18284400
C	-0.45688700	3.29410900	-2.77668500
H	-1.02775500	3.05021500	-3.68495900
H	-1.17398100	3.69074600	-2.04232200
C	-7.12292800	-1.37989500	-0.08349300
H	-7.50412000	-0.39261500	-0.38863000
H	-7.84125800	-2.12882800	-0.44632000
C	-7.02125600	-1.43403500	1.45201600
H	-6.73733400	-2.45429600	1.75819500
H	-8.00120000	-1.22640200	1.90437100
C	-0.81975700	-4.84037500	-1.17255500
H	-0.61131900	-5.90496800	-1.34952000
H	0.08635900	-4.40566900	-0.72165900
C	-1.76406100	0.13250200	3.77136200
H	-1.18319200	0.32931000	4.68179000
H	-2.72597300	-0.29939300	4.07596000
H	-1.95031100	1.08193600	3.25842100
C	2.58461100	-2.91647900	-1.85516200
H	2.83806800	-3.86613300	-1.36166200
H	1.65788100	-3.08944000	-2.42375400
C	1.42025400	4.68246900	-1.81084900
H	0.75510500	5.13123400	-1.05528400
H	2.20149500	5.42442200	-2.02970000
C	1.49094900	1.65392000	3.28099800
H	1.95234300	2.00594500	2.35310700
H	2.17579100	1.89725300	4.10379300
H	0.55002200	2.19626000	3.43341900
C	-1.11410500	-4.12214500	-2.50112800
H	-1.97592200	-4.60346700	-2.99166900
H	-0.26150900	-4.21983400	-3.18809500
C	-5.96772400	-0.44015700	1.97399100
H	-5.87566000	-0.51860500	3.06710200
H	-6.28862300	0.58978400	1.75194600
C	-0.73353500	-2.19085400	3.47769400
H	-1.66858400	-2.65699300	3.81433400
H	-0.07991900	-2.08371800	4.35315900
H	-0.24727600	-2.86515200	2.76257400
C	3.69662600	-2.50211600	-2.83454500
H	4.64845100	-2.40455700	-2.28901400
H	3.84046800	-3.28104600	-3.59697000
B	0.27931800	0.02998400	0.86617500
C	-1.93077900	0.19461800	-0.14469800
O	-0.76176200	-0.48149900	0.00644000

N	-2.04096400	1.35124500	0.42330900
C	-3.14822500	2.19531100	0.20226000
C	-3.79543000	2.76715200	1.31694700
C	-3.58143700	2.55303700	-1.09344500
C	-4.88460900	3.62807000	1.14161900
H	-3.44236400	2.51621300	2.31472700
C	-4.66415500	3.42348800	-1.26143900
H	-3.07164500	2.13847600	-1.95959000
C	-5.32934900	3.95850700	-0.14768600
H	-5.38201200	4.04735500	2.01478700
H	-4.98640600	3.68540300	-2.26809000
H	-6.17256900	4.63296900	-0.28237100
C	4.14171000	1.35272400	0.77479000
O	4.08858800	2.47450200	1.20926300
N	4.67405600	0.24711800	0.48631200
C	6.04649200	-0.01869100	0.73575400
C	6.50598200	-1.30966400	0.41043900
C	6.94711000	0.92258700	1.27668300
C	7.84474400	-1.65594600	0.62316700
H	5.79931600	-2.02467400	-0.00518300
C	8.28507400	0.56559200	1.48468700
H	6.59794000	1.92166500	1.52909500
C	8.74237800	-0.72094000	1.16091000
H	8.18693200	-2.65767600	0.36783200
H	8.97334100	1.29902800	1.90221600
H	9.78384300	-0.99050500	1.32515200

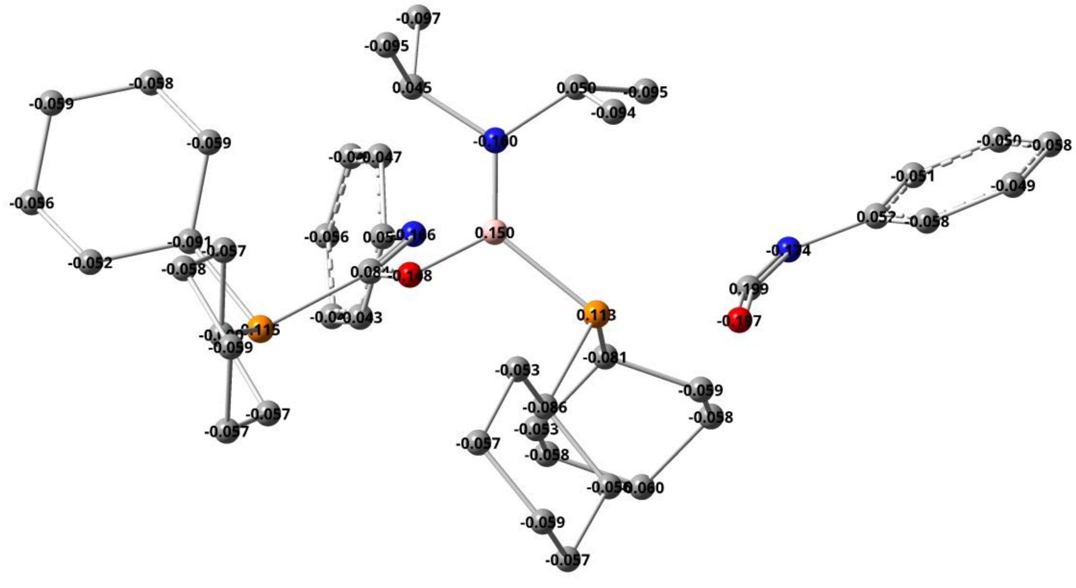

FIG. S135. OPTIMIZED STRUCTURE **2D_TS3_A2**

P	-3.36342200	0.39455500	0.96281700
P	1.91324600	0.41550600	0.41664700
N	0.35705800	-0.03141600	-2.13752000
C	1.50316400	2.03387500	1.27669900
H	0.50787300	1.93313000	1.73769300
C	2.53463100	2.37359100	2.37604600
H	2.53991500	1.59120800	3.14667000
H	3.53869100	2.39777800	1.93160600
C	-4.67732000	-0.57598300	0.00640300
H	-4.74823100	-1.51576200	0.57619100

C	1.60458100	-0.95956500	1.68476200
H	1.21037800	-1.79424000	1.08322400
C	-3.45489100	2.16584200	0.33147500
H	-4.49433900	2.43381700	0.59296700
C	2.93640200	-1.40915700	2.32979300
H	3.66373900	-1.65534200	1.54855400
H	3.36069800	-0.56942800	2.90356400
C	-0.91755300	-0.10045000	-2.90778500
H	-1.71322200	0.13459800	-2.19772600
C	-6.04263300	0.13823500	0.13060100
H	-6.24102300	0.41064600	1.17746800
H	-6.02300700	1.07332000	-0.45119000
C	0.56216300	-0.62786100	2.77351500
H	0.91978500	0.21872500	3.37852800
H	-0.38833800	-0.31968800	2.32370800
C	-2.53136900	3.05715400	1.19688700
H	-2.71399600	2.85950200	2.26238800
H	-1.48683100	2.78704200	0.99054500
C	1.57733100	-0.35972100	-2.93709200
H	1.20822600	-0.60761100	-3.93979700
C	-4.40005100	-0.95244000	-1.46292300
H	-4.34918600	-0.04342400	-2.07927900
H	-3.43253500	-1.45825700	-1.54985000
C	-3.45124700	3.95654000	-1.47677200
H	-3.26921300	4.14009600	-2.54565000
H	-4.50041300	4.23141100	-1.27961200
C	1.44105700	3.15730800	0.21462800
H	2.39673200	3.19076200	-0.32795700
H	0.65620100	2.93024400	-0.52307400
C	2.49517700	0.86308900	-3.08828000
H	1.96188100	1.67986800	-3.59025300
H	3.37759300	0.60042500	-3.68604300
H	2.83819100	1.22756300	-2.11615900
C	1.66372200	-2.29453400	4.34757500
H	1.49261200	-3.17288000	4.98625000
H	2.04267900	-1.49130600	5.00066300
C	-3.25178500	2.45848400	-1.16894100
H	-2.23383700	2.16199900	-1.45010300
H	-3.94794900	1.86366700	-1.77279800
C	2.22939800	3.74055700	3.01750400
H	1.25117800	3.69756700	3.52433800
H	2.98069500	3.96422100	3.78825100
C	0.34405600	-1.84129900	3.69964200
H	-0.39744900	-1.58833200	4.47201900
H	-0.07618700	-2.67107100	3.11146100
C	-7.17018300	-0.76533000	-0.40764000
H	-7.24200800	-1.66195800	0.22800400
H	-8.13408600	-0.24157300	-0.33608300
C	-6.89879000	-1.19276400	-1.86190800
H	-6.92880000	-0.30187500	-2.51054700
H	-7.69117200	-1.86909900	-2.21226300
C	-2.52837700	4.83501100	-0.61449500
H	-2.70081300	5.89869600	-0.83113600
H	-1.47888500	4.62036300	-0.87145800
C	-1.17919600	-1.52002500	-3.43760900
H	-0.45918300	-1.81347600	-4.21244400
H	-2.18288800	-1.56874000	-3.87896300

H	-1.12165200	-2.23665200	-2.61164100
C	1.16864600	4.52887700	0.86091900
H	1.16742100	5.30986500	0.08688100
H	0.16437400	4.52258900	1.31133900
C	2.71624700	-2.60872400	3.27009400
H	2.37676000	-3.47226600	2.67499900
H	3.67041700	-2.89624100	3.73463800
C	2.28401700	-1.61169000	-2.39612900
H	2.74478100	-1.42639400	-1.41948100
H	3.08740300	-1.91115600	-3.08217100
H	1.56857900	-2.43828600	-2.30780300
C	-2.74864200	4.54851600	0.88103600
H	-3.77741900	4.83155400	1.15776800
H	-2.07391600	5.16301300	1.49369800
C	-5.52066200	-1.86619000	-1.99495200
H	-5.32236500	-2.12854200	-3.04442500
H	-5.51145400	-2.80649000	-1.42151300
C	-0.96098700	0.96176400	-4.01928200
H	-1.94273600	0.94068300	-4.50988800
H	-0.19928600	0.78216900	-4.78922800
H	-0.80257100	1.96334300	-3.60177100
C	2.20199800	4.85234200	1.95401000
H	3.20045200	4.93985500	1.49751300
H	1.97231300	5.82216800	2.41811100
B	0.34959500	0.25820600	-0.76749000
C	-1.80057500	-0.30326000	0.22092200
O	-0.90206000	0.64335400	-0.15798600
N	-1.45897500	-1.54548300	0.10560300
C	-2.25268900	-2.59212000	0.61765000
C	-2.55411100	-3.68693300	-0.21833000
C	-2.68755400	-2.62193800	1.96099400
C	-3.32093000	-4.75554300	0.25958500
H	-2.19355700	-3.67942800	-1.24451800
C	-3.44448700	-3.69898700	2.43522900
H	-2.43540000	-1.79590900	2.62130300
C	-3.77547300	-4.76693000	1.58716900
H	-3.55764700	-5.58452100	-0.40556200
H	-3.77405600	-3.70302800	3.47307500
H	-4.36678200	-5.60142200	1.95871300
C	4.38497700	0.62443000	-0.23264300
O	4.56052500	1.81740900	-0.21153100
N	4.68086500	-0.58870400	-0.37806700
C	5.95989400	-1.01417400	-0.82193000
C	6.15901300	-2.40435700	-0.92698600
C	7.00770300	-0.13413800	-1.16448100
C	7.38795200	-2.90810400	-1.36717800
H	5.33989500	-3.06888100	-0.66028700
C	8.23303800	-0.64934100	-1.60470700
H	6.85883100	0.94075100	-1.08401900
C	8.43175100	-2.03458500	-1.70889000
H	7.53007800	-3.98500400	-1.44329400
H	9.03663800	0.03736600	-1.86675400
H	9.38725700	-2.42741100	-2.05097200

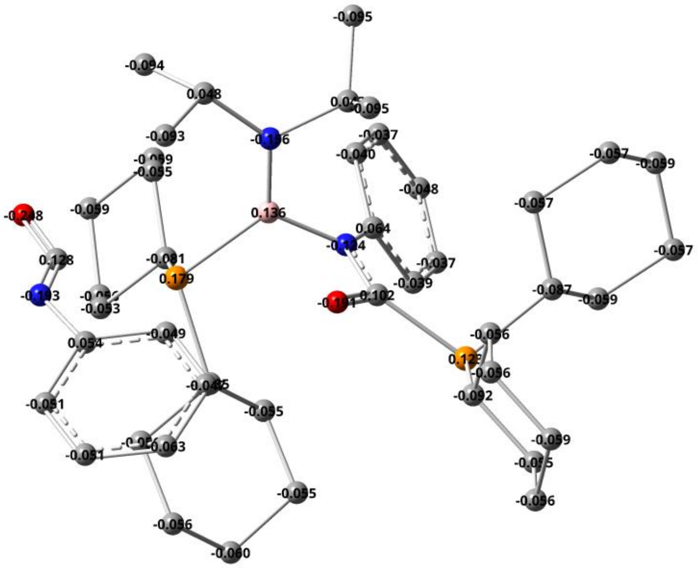

FIG. S136. OPTIMIZED STRUCTURE **2D_TS3_B**

P	-2.71378500	0.35663700	-1.16427000
P	2.30878300	-0.02010100	-0.01014000
N	0.63023600	-0.93505600	2.40127300
C	2.88285400	-1.60641000	-0.84658000
H	2.01004600	-1.93515100	-1.43634200
C	4.06549700	-1.36832200	-1.81254600
H	3.82568700	-0.57785600	-2.53285400
H	4.93276800	-1.03037000	-1.22935200
C	-3.22911100	2.06975900	-0.58907000
H	-2.31112900	2.66384400	-0.71847900
C	1.65453000	1.03282700	-1.44085500
H	0.90924600	1.67424400	-0.95266600
C	-4.07732800	-0.74295500	-0.43190900
H	-4.90008100	-0.02364800	-0.27828300
C	2.73602900	1.96037800	-2.04712000
H	3.28871600	2.48883300	-1.26758300
H	3.46454800	1.36261300	-2.61404800
C	-0.69519300	-1.37418800	2.95177400
H	-1.31716500	-1.59243000	2.08275500
C	-4.30746200	2.61827500	-1.55196700
H	-3.96288900	2.53596100	-2.59235300
H	-5.22165200	2.00643700	-1.46801000
C	0.96413200	0.26484600	-2.58678000
H	1.71035200	-0.32339600	-3.14213100
H	0.22554400	-0.44262700	-2.20387800
C	-4.58153100	-1.80077300	-1.43794700
H	-4.82546800	-1.32180700	-2.39636200
H	-3.78218600	-2.52838500	-1.63307400
C	1.64077400	-0.71731400	3.49368000
H	1.12251500	-1.03609400	4.40387900
C	-3.68344500	2.20953600	0.87805900
H	-4.58463800	1.59848600	1.04684700
H	-2.89686900	1.84092300	1.54507600
C	-4.98015500	-2.14196500	1.48745800
H	-4.71833200	-2.61958300	2.44286100
H	-5.78223900	-1.41709500	1.70114600
C	3.23750100	-2.73638200	0.14013000

H	4.11043700	-2.42925400	0.72957400
H	2.41109200	-2.90503500	0.84118600
C	2.87138900	-1.61696400	3.34335500
H	2.57606400	-2.66630800	3.22122900
H	3.48767400	-1.53395800	4.24823400
H	3.49369800	-1.31490400	2.49625800
C	1.29507800	2.26899500	-4.12011700
H	0.78072500	3.00399300	-4.75577800
H	2.01135800	1.73392600	-4.76507500
C	-3.75681400	-1.39086700	0.92820500
H	-2.93188400	-2.10617700	0.79188200
H	-3.41904900	-0.62654300	1.63960300
C	4.40533000	-2.66481100	-2.57486100
H	3.54948000	-2.94096300	-3.21287000
H	5.25860400	-2.48427400	-3.24397900
C	0.28431000	1.25904700	-3.54930400
H	-0.21266900	0.70793600	-4.36084200
H	-0.50587600	1.79583200	-3.00143600
C	-4.65147600	4.08274400	-1.21539700
H	-3.76169400	4.70675400	-1.39637400
H	-5.43995400	4.44566100	-1.88995400
C	-5.08694200	4.23574100	0.25308000
H	-6.02970100	3.68603400	0.40949600
H	-5.29166900	5.29129000	0.48088400
C	-5.49363200	-3.19074000	0.48442700
H	-6.38415200	-3.69598400	0.88363800
H	-4.72039700	-3.96303800	0.34371000
C	-1.36606800	-0.23330500	3.73613900
H	-0.80599900	0.01340800	4.64757500
H	-2.37699300	-0.53760000	4.03777500
H	-1.43617300	0.66441800	3.11273100
C	3.55213500	-4.03497300	-0.62447800
H	3.79707900	-4.83440600	0.09003600
H	2.65329600	-4.35704400	-1.17472400
C	2.06726700	2.97687300	-2.99255600
H	1.38037600	3.60361400	-2.40309800
H	2.83028100	3.64715600	-3.41266300
C	2.01574700	0.75462200	3.70457800
H	2.68432800	1.12511700	2.92496100
H	2.54719000	0.84950000	4.66064500
H	1.12390300	1.38942500	3.73962100
C	-5.81026800	-2.54160100	-0.87556500
H	-6.64092800	-1.82740600	-0.75227600
H	-6.14734600	-3.30250800	-1.59358700
C	-4.01523100	3.67875100	1.20680800
H	-4.35046800	3.76035100	2.25067800
H	-3.09710800	4.28110700	1.11774100
C	-0.60854300	-2.66559300	3.78761300
H	-0.03658100	-3.44490500	3.27050500
H	-1.62505900	-3.04436000	3.95344900
H	-0.15365900	-2.50375200	4.77272600
C	4.71322700	-3.82484100	-1.61224500
H	5.62885000	-3.59118100	-1.04676400
H	4.90875200	-4.74762600	-2.17696500
B	0.72861400	-0.64586300	1.02747900
C	-1.23156900	0.15896700	-0.02423500
C	-0.64788400	-2.25592800	-0.48552200

C	-1.19682600	-2.42609400	-1.77178700
C	-0.19149100	-3.39082700	0.21387200
C	-1.29870400	-3.70663800	-2.32793100
H	-1.54195700	-1.56428700	-2.33874900
C	-0.28548700	-4.66497200	-0.35440200
H	0.24668300	-3.26250600	1.19878600
C	-0.84474700	-4.83351900	-1.62864400
H	-1.72644400	-3.81587400	-3.32304100
H	0.08031100	-5.52502200	0.20343800
H	-0.91904800	-5.82408000	-2.07208600
N	-0.47855100	-0.98303500	0.12692500
O	-0.78408200	1.16858300	0.57869800
C	4.14907600	0.86037500	0.97111700
N	4.16838500	2.12308000	1.18003400
C	3.20678800	3.11155400	0.98206400
C	1.81117500	2.94503500	1.13820800
C	3.68762300	4.39654600	0.62797200
C	0.93313800	4.00891600	0.89267300
H	1.39266600	1.99283600	1.44447200
C	2.80663900	5.45583700	0.39847600
H	4.76277200	4.52852100	0.52598400
C	1.41882800	5.26923000	0.52102200
H	-0.13608800	3.83481600	1.00033000
H	3.20357800	6.43106100	0.11955300
H	0.73263700	6.09472200	0.33969700
O	4.89517700	-0.09961400	1.14061600

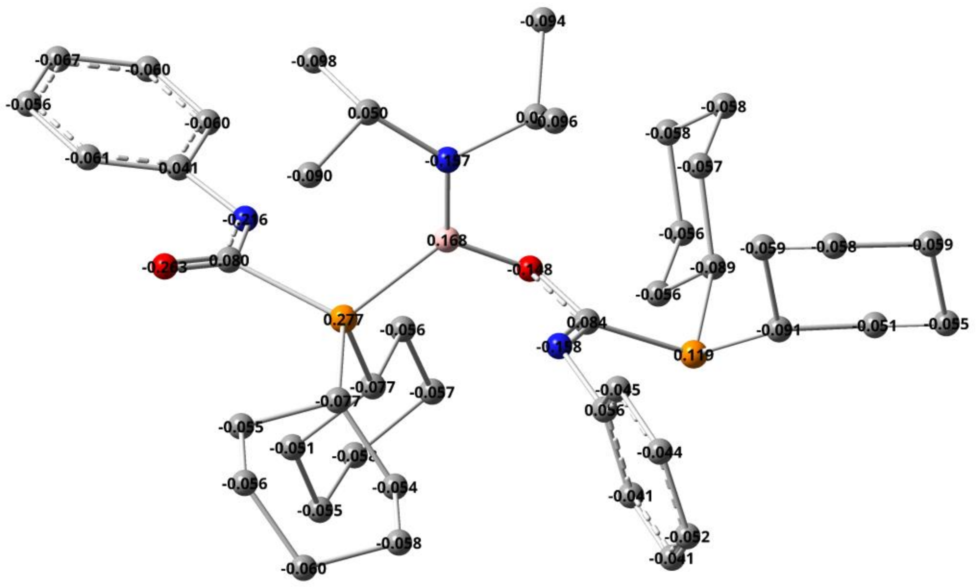

FIG. S137. OPTIMIZED STRUCTURE **2D_TS4_A1**

P	2.83213300	-1.01179700	1.31924700
P	-1.82892500	1.01662800	0.10980200
N	-0.41237400	-0.26576900	-2.25268000
C	-1.83498900	0.42330300	1.87506400
H	-0.83527200	0.67759200	2.26238100
C	-2.90716600	1.18307800	2.69131900
H	-2.79647300	2.26995600	2.58046900
H	-3.89556200	0.91505100	2.29167100
C	4.43107500	-0.82674400	0.32094000

H	4.79220300	0.16965200	0.61911900
C	-1.13532200	2.77313400	0.12105500
H	-0.49292500	2.79381100	-0.77226600
C	2.34796400	-2.82728900	1.18959800
H	3.25559900	-3.31852500	1.58301900
C	-2.20279600	3.88306600	-0.01589600
H	-2.86917200	3.68194500	-0.85893500
H	-2.83812700	3.88889700	0.88299100
C	0.78884900	-0.96529200	-2.80678200
H	1.40003200	-1.24415300	-1.94674900
C	5.47424700	-1.85516000	0.81477000
H	5.52917600	-1.85285700	1.91313600
H	5.16864000	-2.86701500	0.50540700
C	-0.22988500	3.05837200	1.34080600
H	-0.84033300	3.08039700	2.25550400
H	0.51847600	2.26869200	1.46070300
C	1.20656000	-3.11124300	2.19371800
H	1.46912100	-2.70742700	3.18111400
H	0.30590000	-2.58253900	1.85862700
C	-1.37901500	0.18850300	-3.30464700
H	-0.85660700	0.02022100	-4.25369200
C	4.35095900	-0.84331300	-1.21825300
H	4.02630100	-1.83514200	-1.56424000
H	3.60984500	-0.11800100	-1.56992900
C	1.73257800	-4.94803000	-0.07368500
H	1.49058800	-5.35176200	-1.06735600
H	2.63675800	-5.47408400	0.27401800
C	-2.04723700	-1.09734100	2.03153200
H	-3.04177400	-1.34433500	1.64460400
H	-1.32004000	-1.64563100	1.42204100
C	-2.64541600	-0.68124600	-3.31755400
H	-2.39332000	-1.72332600	-3.54640100
H	-3.33841600	-0.31510500	-4.08566400
H	-3.15484000	-0.66030100	-2.35071500
C	-0.55708900	5.55202000	0.99696700
H	-0.04407900	6.51113300	0.83765800
H	-1.14437500	5.65281900	1.92424500
C	2.03211600	-3.43966600	-0.18977400
H	1.15525600	-2.93009300	-0.60744400
H	2.86848900	-3.28109100	-0.88155700
C	-2.81752600	0.79227800	4.17924000
H	-1.84586200	1.12405300	4.58075200
H	-3.59597900	1.32161000	4.74636500
C	0.46968300	4.42005400	1.17089200
H	1.11591300	4.61073900	2.03903400
H	1.12892000	4.37516600	0.29066100
C	6.85831400	-1.54265500	0.21073600
H	7.19814200	-0.56430200	0.58549800
H	7.58981300	-2.28950400	0.55040500
C	6.80218700	-1.50798100	-1.32763600
H	6.56220300	-2.51668300	-1.70184400
H	7.78686700	-1.24204200	-1.73688400
C	0.58085000	-5.21358400	0.91109700
H	0.38989400	-6.29260500	0.99449000
H	-0.34123300	-4.75257700	0.52218400
C	1.63057200	-0.01501800	-3.67564600
H	1.12344900	0.25779900	-4.60975000

H	2.57597300	-0.50492200	-3.94089700
H	1.85383500	0.89859200	-3.11439800
C	-1.92535700	-1.48983600	3.51522700
H	-2.06884100	-2.57479800	3.62121000
H	-0.90870400	-1.26474300	3.87736200
C	-1.51070600	5.25126500	-0.17234000
H	-0.94055600	5.25566400	-1.11585900
H	-2.27388100	6.03743900	-0.25856500
C	-1.66468300	1.69540400	-3.22873400
H	-2.32262700	1.95952100	-2.39393700
H	-2.18519400	2.00761400	-4.14320700
H	-0.72664500	2.25906300	-3.15287700
C	0.90080300	-4.61721100	2.29293300
H	1.77368200	-5.13799100	2.71950300
H	0.06221700	-4.77763000	2.98520500
C	5.73084200	-0.51929100	-1.82246300
H	5.67111900	-0.53703400	-2.92037900
H	6.01109200	0.50573400	-1.53277400
C	0.41217100	-2.25995900	-3.54387400
H	1.32804500	-2.78572600	-3.84318500
H	-0.17131400	-2.06199900	-4.45197000
H	-0.17130600	-2.92091200	-2.89224200
C	-2.95584800	-0.72932300	4.36992100
H	-3.96859900	-1.03821100	4.06753200
H	-2.84206300	-0.98997700	5.43174700
B	-0.49571400	-0.05219800	-0.87411600
C	1.63886400	-0.08191800	0.23018200
O	0.45578500	-0.71441200	-0.01013700
N	1.77702200	1.10305700	-0.27182200
C	2.86362100	1.95003300	0.02242600
C	3.50820400	2.61207700	-1.04225000
C	3.26032600	2.24060300	1.34644600
C	4.55887000	3.50174700	-0.79235000
H	3.18061000	2.41194400	-2.06007700
C	4.30309500	3.14093100	1.58937900
H	2.74884100	1.75636600	2.17591200
C	4.96505000	3.76980400	0.52380000
H	5.05488400	3.99335800	-1.62758000
H	4.59606100	3.35380700	2.61623500
H	5.77658300	4.46837500	0.71675600
C	-3.66916100	0.90439100	-0.51009200
O	-4.21367800	1.89425800	-1.04109700
N	-4.00074100	-0.34204000	-0.23141300
C	-5.27840000	-0.85545200	-0.51629300
C	-5.46104900	-2.23400600	-0.24797000
C	-6.37321100	-0.12184700	-1.04030500
C	-6.68566500	-2.86141400	-0.49132700
H	-4.61553800	-2.79360000	0.14992600
C	-7.59694700	-0.76125300	-1.27812000
H	-6.24547200	0.93482600	-1.25363000
C	-7.76604500	-2.12781400	-1.00876400
H	-6.79906600	-3.92377200	-0.27749100
H	-8.42792100	-0.18207300	-1.68030900
H	-8.72191100	-2.61339700	-1.19831200

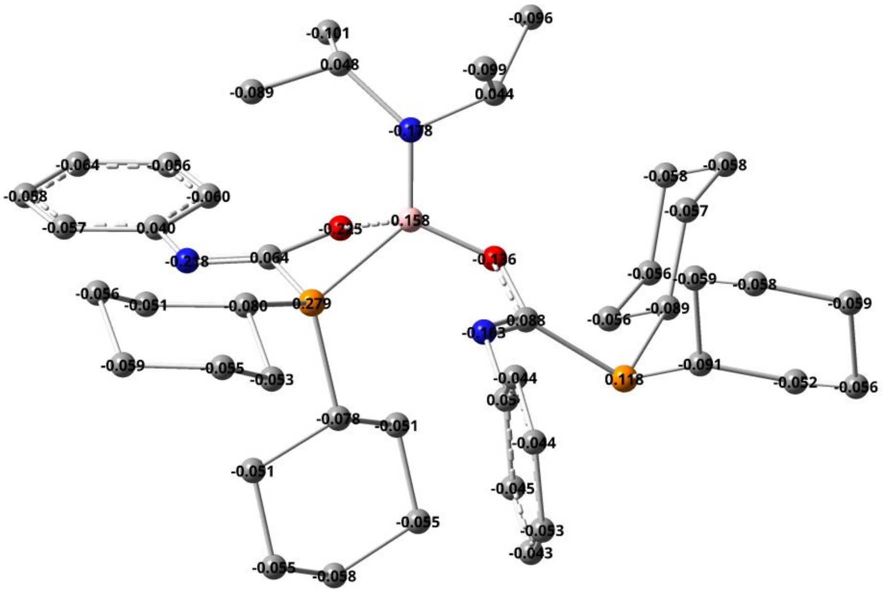

FIG. S138. OPTIMIZED STRUCTURE **2D_TS4_A2**

P	2.82374700	-0.76896300	1.35104000
P	-1.60180600	0.85376000	-0.06638300
N	-0.59500500	-0.93423600	-2.33280800
C	-1.12294500	1.21347400	1.70005400
H	-0.21468000	1.83102700	1.64057100
C	-2.24630300	2.02218300	2.39619500
H	-2.46985500	2.94093200	1.84177400
H	-3.16595500	1.41991700	2.40109200
C	4.43435200	-0.29475500	0.47594000
H	4.58961500	0.74705300	0.79908600
C	-1.84713400	2.49026900	-0.93355900
H	-1.56841600	2.25158700	-1.96990900
C	2.65316300	-2.63968700	1.20248800
H	3.50744500	-2.98264900	1.81406900
C	-3.31797700	2.96784000	-0.93158400
H	-3.98790300	2.16188500	-1.25154400
H	-3.62112900	3.22403500	0.09311400
C	0.67252300	-1.38524700	-2.98762600
H	1.44207600	-1.33989300	-2.21451300
C	5.59960300	-1.13104000	1.05251200
H	5.57405900	-1.11748600	2.15189200
H	5.48983500	-2.18074700	0.73816400
C	-0.88947500	3.60508100	-0.45635800
H	-1.12336900	3.88594000	0.58048000
H	0.14582200	3.25188500	-0.47501100
C	1.36370200	-3.09375800	1.93285900
H	1.33145700	-2.64990500	2.93813100
H	0.48894700	-2.72500000	1.38215600
C	-1.79548100	-1.05721200	-3.20925400
H	-1.39453000	-1.19408700	-4.22194800
C	4.46948600	-0.30527400	-1.06674600
H	4.33989100	-1.33121700	-1.43795100
H	3.64375400	0.29006900	-1.47315100
C	2.66100100	-4.83503300	-0.08848800
H	2.70895100	-5.26988100	-1.09744200
H	3.52929200	-5.22412900	0.46850200
C	-0.82552000	-0.06430700	2.51330100

H	-1.72552600	-0.69391700	2.53126800
H	-0.04289800	-0.65718300	2.03512800
C	-2.63718900	-2.30392000	-2.87164800
H	-2.01633600	-3.20594400	-2.86232800
H	-3.42082400	-2.43012500	-3.63150800
H	-3.10434800	-2.20696600	-1.88837300
C	-2.50478000	5.33424900	-1.39777500
H	-2.59867500	6.19755500	-2.07154800
H	-2.79439800	5.68017100	-0.39219700
C	2.76761100	-3.29947900	-0.18648500
H	1.96303700	-2.91815700	-0.82610100
H	3.72107000	-3.03114200	-0.65742200
C	-1.83346700	2.38179300	3.83706800
H	-0.95475400	3.04670400	3.80386200
H	-2.64457000	2.94694800	4.31727000
C	-1.04579600	4.84413400	-1.35964100
H	-0.37859600	5.64205700	-1.00455700
H	-0.72045000	4.58565100	-2.38040300
C	6.95146800	-0.59053700	0.54487500
H	7.09785700	0.42806100	0.93791100
H	7.77123800	-1.20820000	0.93848100
C	6.99823000	-0.55340300	-0.99367000
H	6.95274400	-1.58450200	-1.38078300
H	7.95261800	-0.12703500	-1.33340000
C	1.36904500	-5.26711200	0.62545400
H	1.32219400	-6.36275800	0.69953800
H	0.49916000	-4.94537000	0.03104200
C	1.09076300	-0.39633200	-4.09230200
H	0.37445500	-0.37631600	-4.92457900
H	2.06756000	-0.68643800	-4.50225500
H	1.17147600	0.61479100	-3.67584300
C	-0.40834200	0.29461500	3.95073300
H	-0.20528000	-0.62955900	4.50955300
H	0.53631500	0.86165800	3.92457600
C	-3.45866400	4.20701100	-1.83511900
H	-3.23284400	3.92129200	-2.87554100
H	-4.49984600	4.55842600	-1.81980100
C	-2.67513500	0.19724000	-3.26283900
H	-3.17368700	0.38953200	-2.30550300
H	-3.46724000	0.04552900	-4.00731000
H	-2.09179800	1.07687600	-3.55921700
C	1.28599100	-4.62919300	2.02319700
H	2.11590800	-5.00435100	2.64456400
H	0.35165800	-4.92004600	2.52332100
C	5.81690700	0.25134400	-1.56590000
H	5.84176900	0.23871400	-2.66517900
H	5.90306100	1.30461200	-1.25445700
C	0.62264700	-2.83737400	-3.49217200
H	1.61304200	-3.12437400	-3.86979300
H	-0.09576100	-2.96767800	-4.31171100
H	0.35118000	-3.52103600	-2.67926400
C	-1.49181500	1.12632100	4.65707800
H	-2.39929000	0.51282600	4.77156700
H	-1.16358400	1.40825200	5.66749500
B	-0.51360700	-0.52024100	-0.97808200
C	1.58281500	-0.08780100	0.14154300
O	0.63797000	-0.97224100	-0.24864100

N	1.49826900	1.11850900	-0.32698700
C	2.35406600	2.15526500	0.10061500
C	2.98475400	2.94406900	-0.88344800
C	2.51980500	2.49500000	1.46074000
C	3.80536600	4.01523300	-0.51341000
H	2.82622300	2.69525500	-1.93114600
C	3.33255500	3.57556400	1.82268800
H	2.02169500	1.90161000	2.22439800
C	3.98638700	4.33583500	0.84108100
H	4.29785700	4.60549800	-1.28442900
H	3.45413500	3.82313700	2.87603700
H	4.61926600	5.17351000	1.12691800
C	-2.96068800	-0.39477200	0.11748900
O	-2.29377000	-1.50740500	0.11521300
N	-4.21349300	-0.05421300	0.23861100
C	-5.23245700	-1.01215500	0.40872200
C	-6.55524100	-0.51159300	0.37560600
C	-5.04371200	-2.40210000	0.61823100
C	-7.65326300	-1.36181700	0.53242700
H	-6.69015200	0.55785300	0.22225200
C	-6.15113900	-3.24523200	0.77761400
H	-4.03364800	-2.79862100	0.65264100
C	-7.45853500	-2.73810100	0.73434900
H	-8.66221100	-0.95215400	0.49990500
H	-5.98847700	-4.31084900	0.93781900
H	-8.31181300	-3.40284400	0.85878600

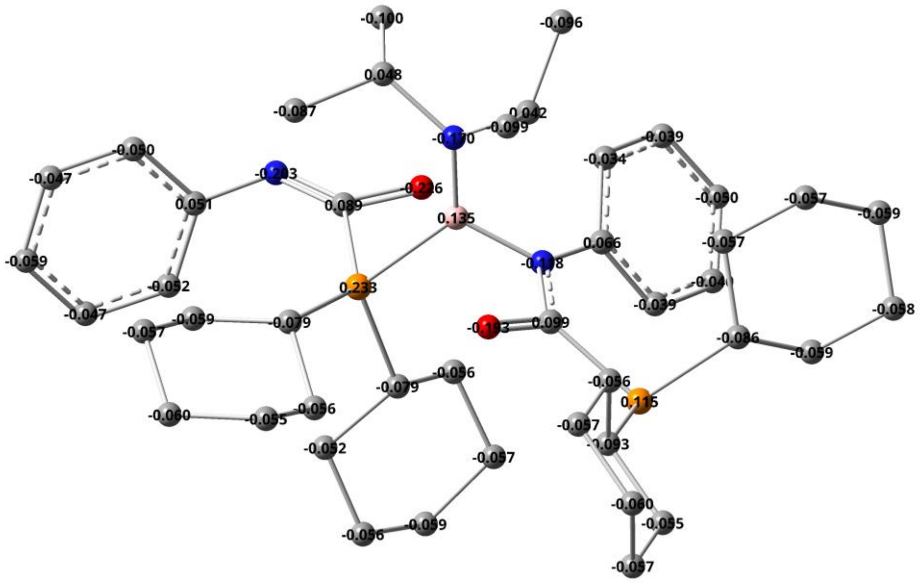

FIG. S139. OPTIMIZED STRUCTURE **2D_TS4_B**

P	2.97426500	0.89627600	1.02223800
P	-1.91500900	-0.06657700	0.20555100
N	-0.36964800	-1.35897100	-2.08384000
C	-1.42058800	0.30607100	1.96996800
H	-0.54427500	0.96654300	1.86076000
C	-2.51462700	1.06495900	2.75980000
H	-2.93774200	1.89972600	2.19252000
H	-3.34063900	0.37074000	2.96247400
C	3.12219600	2.60588600	0.25018600

H	2.13158400	3.05235200	0.42769400
C	-2.40243000	1.59094300	-0.57743300
H	-2.01584600	1.45505200	-1.59704800
C	4.45889900	0.00279500	0.24009000
H	5.10160300	0.84858300	-0.05884500
C	-3.92061000	1.84512100	-0.66340600
H	-4.44491000	0.97048200	-1.06279500
H	-4.32191900	2.01196000	0.34579800
C	0.95948700	-1.44935600	-2.77001700
H	1.70593400	-1.38755400	-1.97692300
C	4.17272900	3.41928000	1.04181200
H	3.94763600	3.38368500	2.11705400
H	5.16831300	2.96302100	0.90886800
C	-1.67630000	2.83352700	-0.00492600
H	-2.05169700	3.06792500	0.99984600
H	-0.60111900	2.64770100	0.06543100
C	5.26034100	-0.82758700	1.26559200
H	5.49331000	-0.21199700	2.14567100
H	4.64927500	-1.67112200	1.61098100
C	-1.50902200	-1.69243600	-2.99016800
H	-1.08132100	-1.66460500	-4.00030400
C	3.40812200	2.67513000	-1.26415400
H	4.38460100	2.21217600	-1.47860700
H	2.64248400	2.11502300	-1.81108500
C	5.44455000	-1.38268800	-1.64957900
H	5.19508300	-1.99546600	-2.52800100
H	6.06567800	-0.54570500	-2.00821900
C	-1.01199200	-0.92767900	2.80178100
H	-1.90552200	-1.54228100	2.98506600
H	-0.31125100	-1.56548900	2.26321500
C	-2.02892500	-3.12341000	-2.74901800
H	-1.20282300	-3.84252800	-2.74072700
H	-2.71595500	-3.40013900	-3.56096500
H	-2.56353800	-3.20360700	-1.79903000
C	-3.45661300	4.32244600	-1.04934500
H	-3.63460500	5.17104500	-1.72518800
H	-3.85312200	4.61050200	-0.06201700
C	4.15247600	-0.82242100	-1.02435600
H	3.49603100	-1.66136700	-0.75087700
H	3.61430400	-0.20353600	-1.75423800
C	-1.93421000	1.56534900	4.09720500
H	-1.11623300	2.27409700	3.88988000
H	-2.70538100	2.11867200	4.65191000
C	-1.94821900	4.04460100	-0.92065400
H	-1.42708800	4.92720100	-0.52211000
H	-1.52225000	3.84445000	-1.91698000
C	4.22294000	4.88029000	0.55272500
H	3.25888400	5.36375200	0.77879600
H	4.99585500	5.43329200	1.10544800
C	4.48479600	4.95885900	-0.96216100
H	5.49051000	4.56102700	-1.17588900
H	4.47897500	6.00642600	-1.29526800
C	6.24428800	-2.20851500	-0.62611900
H	7.17501000	-2.57844300	-1.07877300
H	5.65306400	-3.09128500	-0.33472500
C	1.15252700	-0.23159900	-3.69372400
H	0.43759700	-0.24296500	-4.52826400

H	2.16377300	-0.23881100	-4.12271200
H	1.01373500	0.69309400	-3.12352100
C	-0.40886800	-0.46681100	4.14195800
H	-0.10523800	-1.34545000	4.72815600
H	0.50510800	0.11574900	3.94126300
C	-4.20554800	3.07398000	-1.54733200
H	-3.89120000	2.84993600	-2.58004400
H	-5.28853000	3.25829000	-1.57634700
C	-2.65717000	-0.67762300	-2.99991700
H	-3.19540600	-0.64324000	-2.04658800
H	-3.38711700	-0.98031900	-3.76187100
H	-2.29660300	0.32572400	-3.25232800
C	6.55389300	-1.37274700	0.62954200
H	7.20873000	-0.53012300	0.35227700
H	7.10411300	-1.97627100	1.36532600
C	3.44315800	4.13956300	-1.74468100
H	3.65981500	4.17335300	-2.82218200
H	2.44546300	4.58569400	-1.60442800
C	1.21126500	-2.76955800	-3.52001200
H	1.11139100	-3.63760500	-2.85954900
H	2.23867700	-2.75777900	-3.90643700
H	0.53882400	-2.90364700	-4.37673100
C	-1.40221900	0.39403900	4.94337600
H	-2.25192200	-0.23578200	5.25197300
H	-0.93203500	0.77040300	5.86291000
B	-0.39849200	-0.92123400	-0.73557200
C	1.45715400	0.32741900	0.05788200
C	1.43037600	-2.10540500	0.69238800
C	2.13662300	-2.05554800	1.90753400
C	1.16994100	-3.35419300	0.09943300
C	2.62461100	-3.23429200	2.48454400
H	2.28701700	-1.10348200	2.41184800
C	1.65507500	-4.52634000	0.68582200
H	0.54824800	-3.39695100	-0.78863600
C	2.39797600	-4.47504700	1.87443500
H	3.16976100	-3.17638400	3.42530300
H	1.43730600	-5.48411100	0.21697900
H	2.77296500	-5.38914900	2.33014800
N	0.90988300	-0.93205700	0.06894300
O	0.79607000	1.19209400	-0.57672800
C	-2.75316200	-1.86246700	0.28393000
N	-3.97929200	-2.23195300	0.13649200
C	-5.05820300	-1.34882800	0.11367600
C	-5.86578300	-1.26855800	-1.04507600
C	-5.42496900	-0.58584900	1.24403900
C	-6.96455200	-0.40456600	-1.08585500
H	-5.60443500	-1.88315700	-1.90405600
C	-6.53041200	0.27330600	1.19840600
H	-4.84866700	-0.70043400	2.15837000
C	-7.29954400	0.38003500	0.03130600
H	-7.56439300	-0.34228100	-1.99293000
H	-6.79247900	0.85748400	2.07994800
H	-8.15733500	1.04893400	-0.00397300
O	-1.68579500	-2.53958400	0.40000200

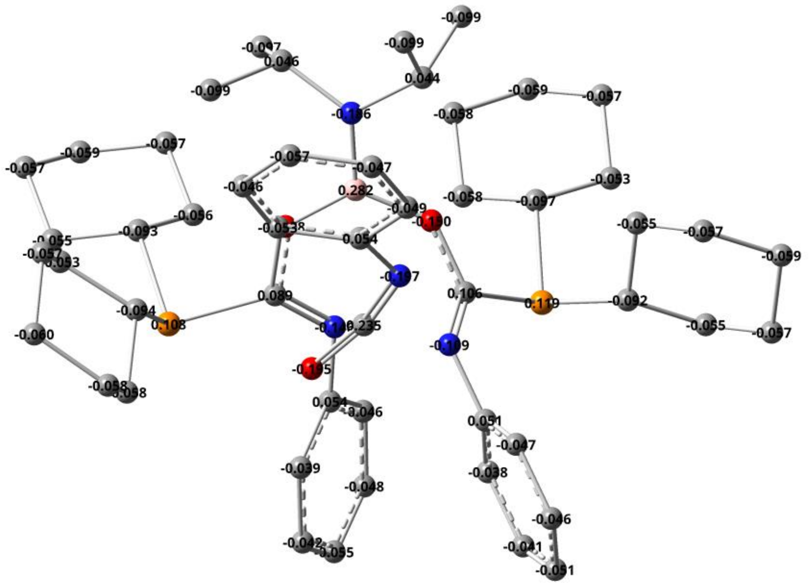

FIG. S140. OPTIMIZED STRUCTURE **2D_TS5**

P	-3.18410700	-1.27526600	1.27391900
P	3.07561700	-1.33097600	0.18713100
O	-0.77071400	1.83797900	1.92464400
O	1.01368700	0.09140900	-0.89886200
O	-1.42881600	-0.20880000	-0.53713000
N	1.53433100	0.82266700	1.19853100
N	0.50527400	3.03091800	0.25717000
N	-0.37209800	-1.56126800	0.98502000
N	-0.56400900	0.88729500	-2.51504200
C	0.01354900	2.24225700	1.11032000
C	-0.26478200	4.11368500	-0.23890800
C	-1.67437400	4.11271200	-0.26678800
C	-2.36610100	5.18925300	-0.83343700
H	-3.45508700	5.17238900	-0.85343100
C	-1.66820800	6.27919600	-1.37574700
H	-2.20975900	7.11367700	-1.81668600
C	-0.26481100	6.28128300	-1.34537200
H	0.28795900	7.12056000	-1.76451800
C	0.43507700	5.20588900	-0.78666800
H	1.52277800	5.18775700	-0.77110100
C	1.71993500	-0.02580800	0.24319700
C	2.28149200	0.83111500	2.40408700
C	2.45767700	-0.31343200	3.20378800
H	2.02045400	-1.25921000	2.90154800
C	3.19078700	-0.22013600	4.39307200
H	3.31742000	-1.10976600	5.00744200
C	3.74392700	1.00321300	4.79805000
H	4.31091700	1.06804800	5.72481200
C	3.54842300	2.14741700	4.00852000
H	3.96370300	3.10451900	4.31945800
C	2.81386000	2.06739400	2.82081400
H	2.64973300	2.94406400	2.19752700
C	-1.45105200	-0.99192200	0.59181100
C	-0.28923900	-2.24268600	2.21287200
C	0.32347800	-3.51035300	2.24422700
H	0.64970800	-3.96011500	1.30961100
C	0.50553600	-4.16947300	3.46533500

H	0.97130600	-5.15353800	3.47939900
C	0.10605400	-3.56303500	4.66693500
H	0.26118500	-4.07328500	5.61568900
C	-0.47738300	-2.28719300	4.63775600
H	-0.76909500	-1.79775700	5.56545600
C	-0.67383400	-1.62567000	3.42160800
H	-1.07545000	-0.61582900	3.39358000
C	0.54644300	1.58690500	-3.21393300
H	1.32890000	1.71457900	-2.45935100
C	1.11056900	0.73647100	-4.36649700
H	1.45128400	-0.23825500	-3.99835800
H	1.96056600	1.24758500	-4.83805100
H	0.35048100	0.56657500	-5.14185600
C	0.13388400	2.98922700	-3.69708500
H	-0.56549400	2.94572300	-4.54296700
H	1.02564600	3.53058800	-4.03809300
H	-0.32697600	3.56378300	-2.88835900
C	-1.83582200	0.75227500	-3.27302000
H	-1.65616700	1.23481900	-4.24142700
C	-3.00055500	1.49490200	-2.59896200
H	-3.23326300	1.04327100	-1.62976800
H	-3.89750400	1.44491200	-3.23125600
H	-2.74182200	2.54716900	-2.43877700
C	-2.16218400	-0.72415100	-3.55348100
H	-1.33580800	-1.20653000	-4.08962200
H	-3.07094600	-0.80680200	-4.16479500
H	-2.32801700	-1.26585100	-2.61726400
C	-3.85436500	-2.10565300	-0.29401100
H	-3.91443700	-1.36726100	-1.10857200
C	-2.92167900	-3.25954800	-0.73163300
H	-1.92670500	-2.87397400	-0.98984500
H	-2.78048200	-3.94724200	0.11780900
C	-3.49967000	-4.03369100	-1.93240100
H	-3.53656200	-3.36476600	-2.80655600
H	-2.82531900	-4.86203300	-2.19391300
C	-4.91533900	-4.56129600	-1.64336200
H	-5.31998300	-5.07267800	-2.52831100
H	-4.86742500	-5.30780000	-0.83422000
C	-5.84349300	-3.41261000	-1.21307400
H	-6.84479900	-3.79652500	-0.97087300
H	-5.96434800	-2.70684300	-2.05102300
C	-5.26647200	-2.66248000	0.00273400
H	-5.94663300	-1.85561100	0.30139800
H	-5.19782100	-3.35298700	0.85830700
C	-3.80843200	0.51714900	1.19870600
H	-3.06987600	1.07903400	0.60961400
C	-5.18852100	0.72137300	0.54442300
H	-5.20430300	0.31820300	-0.47676700
H	-5.95736000	0.18279300	1.12096600
C	-5.54274800	2.22185900	0.51665500
H	-4.80211200	2.74940900	-0.10602800
H	-6.52254900	2.36573600	0.03836300
C	-5.54314700	2.82239900	1.93426400
H	-6.35255500	2.35518800	2.51863500
H	-5.76108900	3.89916700	1.89162500
C	-4.19892000	2.57604800	2.64297900
H	-3.40437500	3.14330200	2.13614600

H	-4.23971300	2.94606000	3.67736100
C	-3.82242800	1.08262100	2.63749600
H	-4.55059500	0.51073800	3.23493700
H	-2.84056500	0.94832400	3.10675700
C	4.48189000	-0.06651400	0.04773900
H	4.29474400	0.58815000	0.91395600
C	4.46264000	0.81373200	-1.21718500
H	3.48138100	1.29477400	-1.32709100
H	4.61871000	0.18856700	-2.10982200
C	5.57081300	1.88373400	-1.15126700
H	5.56793500	2.48257700	-2.07358000
H	5.35214400	2.57276100	-0.31991300
C	6.95286200	1.24265600	-0.92948200
H	7.20550400	0.61747500	-1.80175100
H	7.72661000	2.02027400	-0.85967500
C	6.96000800	0.36811000	0.33796900
H	6.78304300	1.00531900	1.21921900
H	7.94432200	-0.10227200	0.47397700
C	5.86449900	-0.71497600	0.27735100
H	5.84916400	-1.29814000	1.20818100
H	6.09672900	-1.41616500	-0.53800500
C	2.79552700	-1.96695200	-1.56181100
H	2.56718600	-1.11492900	-2.21672000
C	4.03270300	-2.70185900	-2.12096300
H	4.89779600	-2.02814500	-2.16374600
H	4.30408100	-3.53256800	-1.44971300
C	3.74233700	-3.24924200	-3.53390900
H	3.56383200	-2.39915600	-4.21239900
H	4.62568600	-3.78151100	-3.91483700
C	2.51160200	-4.17398300	-3.54901100
H	2.30545400	-4.51477800	-4.57363700
H	2.72886800	-5.07305000	-2.94978900
C	1.28138800	-3.46330700	-2.95744300
H	0.42159400	-4.14805200	-2.92120200
H	0.99263500	-2.62390600	-3.60981100
C	1.58264200	-2.92669000	-1.54714500
H	1.81647800	-3.77291800	-0.88049300
H	0.70453800	-2.42820900	-1.12116300
B	-0.32683100	0.27129900	-1.26685000
H	-2.21796200	3.26440400	0.14222100

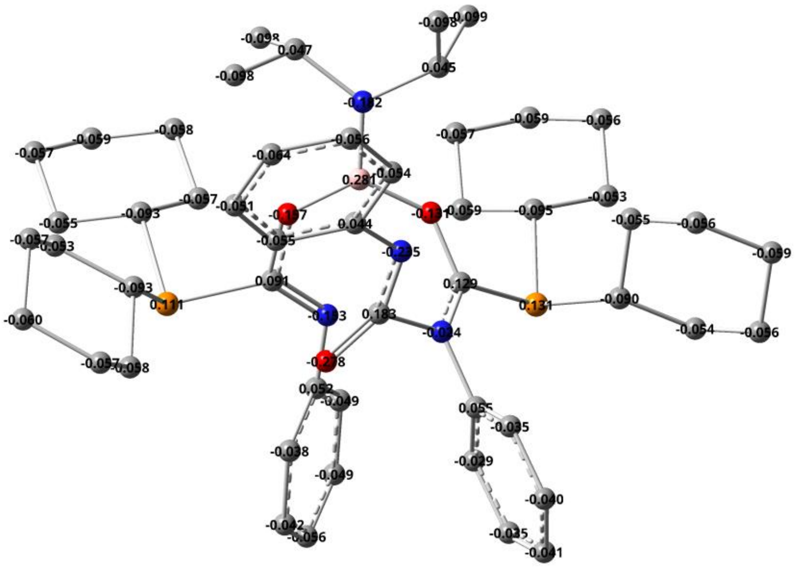

FIG. S141. OPTIMIZED STRUCTURE **2D_TS6_A**

P	3.19714500	1.09178400	1.23362500
P	-3.17833900	1.07168100	0.49725900
O	0.59857500	-2.01720500	1.76891000
O	-1.08157300	0.07011300	-0.91337900
O	1.38263400	0.10994800	-0.59815600
N	-1.42208800	-1.05116900	1.06649700
N	-0.41262300	-2.65300500	-0.30408000
N	0.39004700	1.29025300	1.11001200
N	0.38037000	-0.57068800	-2.71438300
C	-0.21872400	-2.02527300	0.83500300
C	0.52066100	-3.61369900	-0.73575300
C	1.85957800	-3.71219500	-0.29200400
C	2.72148200	-4.66732900	-0.84247300
H	3.75275400	-4.71859000	-0.49352500
C	2.27440200	-5.55606900	-1.83199200
H	2.95007400	-6.29735200	-2.25498800
C	0.93908700	-5.48292100	-2.26072800
H	0.57045300	-6.17356000	-3.01844700
C	0.07523500	-4.52461600	-1.72040500
H	-0.96304900	-4.46180300	-2.04184000
C	-1.72474500	-0.08055000	0.22843900
C	-2.14961200	-1.27803800	2.29033800
C	-2.31300800	-0.27141100	3.24980700
H	-1.87040300	0.70681700	3.09766700
C	-3.04201400	-0.55477500	4.41224300
H	-3.16751800	0.22388600	5.16179500
C	-3.58458100	-1.83067500	4.61862400
H	-4.14487500	-2.04424500	5.52684900
C	-3.38955600	-2.83819600	3.66067300
H	-3.79562300	-3.83478300	3.82184700
C	-2.66883400	-2.56609200	2.49315400
H	-2.50285400	-3.33027500	1.73648100
C	1.45317900	0.78364200	0.59954600
C	0.38150100	1.82581700	2.41258700
C	-0.15898600	3.10747700	2.62927800
H	-0.48421700	3.69612300	1.77478400
C	-0.26054100	3.61104000	3.93182400

H	-0.66696500	4.60865700	4.09084100
C	0.14858400	2.83535400	5.02811800
H	0.05880800	3.22732300	6.03952400
C	0.66071200	1.54658000	4.81158400
H	0.96255000	0.92897500	5.65597100
C	0.77682200	1.03784400	3.51417200
H	1.11655600	0.02062600	3.33287800
C	-0.82564900	-0.96951600	-3.48791800
H	-1.58813900	-1.19789600	-2.73711400
C	-1.32401800	0.18734300	-4.37257400
H	-1.52746400	1.07949000	-3.76936800
H	-2.24633800	-0.09950100	-4.89579100
H	-0.57465000	0.44887700	-5.13255600
C	-0.60504300	-2.24668900	-4.31824600
H	0.00228800	-2.06058200	-5.21411900
H	-1.57841400	-2.62372600	-4.65837600
H	-0.12103500	-3.02279900	-3.71859600
C	1.65368700	-0.47551300	-3.47793800
H	1.40676200	-0.78378000	-4.50053700
C	2.72234000	-1.44547500	-2.95026400
H	3.03233700	-1.16788200	-1.93778500
H	3.60514200	-1.41996600	-3.60360400
H	2.33585800	-2.46943900	-2.91855700
C	2.15424200	0.97626400	-3.54701700
H	1.38629000	1.63242300	-3.97503600
H	3.05611400	1.04041700	-4.17066600
H	2.40168700	1.34226900	-2.54679800
C	3.75014500	2.15464500	-0.23942500
H	3.89181000	1.51682500	-1.12562100
C	2.69308800	3.23845200	-0.55785300
H	1.74665700	2.77611500	-0.86620600
H	2.47935900	3.80800200	0.36114500
C	3.17369700	4.20007900	-1.66200100
H	3.28051900	3.63949100	-2.60400600
H	2.40822100	4.97052400	-1.83550500
C	4.52212100	4.84889200	-1.30717300
H	4.86167100	5.49689200	-2.12769300
H	4.39507700	5.49143600	-0.42096100
C	5.57505400	3.77090200	-0.99962200
H	6.52866700	4.23520600	-0.71028000
H	5.76915000	3.17938900	-1.90914500
C	5.09291500	2.83297200	0.12364300
H	5.86134600	2.08058200	0.33766500
H	4.95152400	3.41403900	1.04872200
C	3.95723200	-0.61688800	0.93907400
H	3.32405200	-1.14414900	0.21215700
C	5.39514600	-0.57538600	0.38522400
H	5.41546300	-0.07938400	-0.59493100
H	6.03807200	0.00973600	1.06279900
C	5.97313000	-1.99911400	0.26398500
H	5.36136000	-2.57537500	-0.44835900
H	6.99112900	-1.95225300	-0.14934000
C	5.97666100	-2.70862100	1.62949300
H	6.63917200	-2.15787200	2.31727900
H	6.38876000	-3.72335100	1.53271300
C	4.55676300	-2.76287000	2.21833100
H	3.93356200	-3.41912800	1.59560500

H	4.57327100	-3.21025900	3.22232400
C	3.91356700	-1.36424900	2.29466500
H	4.44992500	-0.75882800	3.04296300
H	2.87295600	-1.45664800	2.62984600
C	-4.49267600	-0.25327100	0.16052400
H	-4.22689000	-1.04446100	0.87908200
C	-4.46728500	-0.87093800	-1.25103600
H	-3.46483000	-1.26078200	-1.47294900
H	-4.69000700	-0.09741100	-2.00187800
C	-5.50746900	-2.00327400	-1.36606800
H	-5.50417300	-2.40885600	-2.38786800
H	-5.21492800	-2.82560500	-0.69409700
C	-6.91550700	-1.51152800	-0.98496900
H	-7.24061700	-0.74651500	-1.70910600
H	-7.63701700	-2.33804400	-1.04875400
C	-6.92448100	-0.90442800	0.43004300
H	-6.67269300	-1.68768500	1.16283800
H	-7.92876400	-0.53724200	0.68456500
C	-5.90199400	0.24258900	0.55347600
H	-5.88396100	0.63226300	1.58036300
H	-6.21036800	1.07127100	-0.10068400
C	-3.02105000	2.06262800	-1.09219700
H	-2.76637900	1.38119800	-1.91510700
C	-4.33044800	2.79914900	-1.44756100
H	-5.14499800	2.08294600	-1.61457300
H	-4.63386900	3.44380200	-0.60699700
C	-4.13766000	3.65687200	-2.71545100
H	-3.92591700	2.98867100	-3.56583000
H	-5.07248000	4.18562800	-2.94972100
C	-2.97928000	4.65856400	-2.55908100
H	-2.84331200	5.22890600	-3.48875700
H	-3.23511300	5.38557500	-1.77135000
C	-1.67609800	3.93739400	-2.17207400
H	-0.86799800	4.66450000	-2.00700700
H	-1.35454200	3.28806300	-3.00163000
C	-1.87413100	3.08472100	-0.90640900
H	-2.13242300	3.74552100	-0.06294200
H	-0.94546600	2.57046800	-0.63227000
B	0.24684900	-0.21558500	-1.36051900
H	2.19968800	-3.03525600	0.48289400

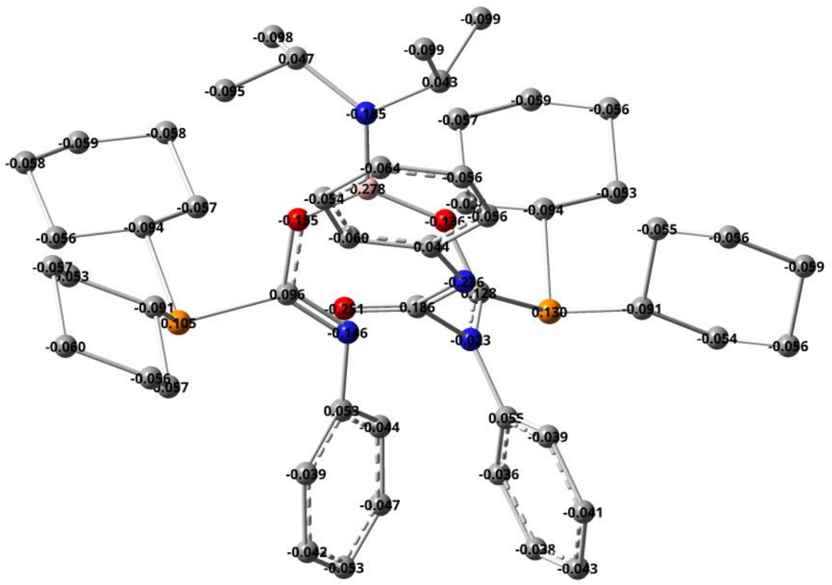

FIG. S142. OPTIMIZED STRUCTURE **2D_TS6_B**

P	-3.42178400	-0.86192400	1.32931000
P	2.62854600	-1.80907400	0.25440700
O	-0.45004700	1.83585600	0.81960500
O	0.83343500	-0.13744000	-0.94222300
O	-1.62127400	-0.26953700	-0.62777900
N	1.61895200	0.73252500	0.99876200
N	1.57498900	2.90761800	0.14054700
N	-0.64350600	-1.45334300	1.07725500
N	-0.63561000	0.93786300	-2.49768700
C	0.77272100	1.99980100	0.62685100
C	1.06069000	4.13296900	-0.32217000
C	-0.29548300	4.54276300	-0.26475900
C	-0.68273400	5.77615500	-0.80403700
H	-1.73063500	6.07193900	-0.75282800
C	0.25427500	6.62764000	-1.40864900
H	-0.05747900	7.58230600	-1.82879200
C	1.60135500	6.23295700	-1.46008300
H	2.34296200	6.88325000	-1.92269200
C	1.99844700	5.00586500	-0.92151200
H	3.03838500	4.68542500	-0.96332000
C	1.54795100	-0.28447100	0.16372100
C	2.39704400	0.73175000	2.20820800
C	2.31002100	-0.33813500	3.11197900
H	1.63741700	-1.16637100	2.91768200
C	3.09435300	-0.31791800	4.27168700
H	3.02259600	-1.14736900	4.97224400
C	3.94330800	0.76588200	4.53651700
H	4.54649300	0.77849000	5.44236800
C	4.00306200	1.84024000	3.63518200
H	4.65453000	2.68829400	3.83766200
C	3.23260100	1.83197700	2.46705900
H	3.25957500	2.64413800	1.74498600
C	-1.68512400	-0.87241800	0.60226100
C	-0.61418000	-2.03413700	2.35958400
C	-0.06139300	-3.32394300	2.49053900
H	0.25641500	-3.85142300	1.59494400
C	0.07639200	-3.90471600	3.75609000

H	0.49620600	-4.90545400	3.84387400
C	-0.30931700	-3.19951400	4.90703500
H	-0.19011500	-3.64904700	5.89101700
C	-0.83658800	-1.90571900	4.78000800
H	-1.12332800	-1.34220200	5.66626000
C	-0.98799000	-1.32204900	3.51767300
H	-1.35522200	-0.30470800	3.41928100
C	0.56703700	1.50450700	-3.17199500
H	1.35661100	1.50580500	-2.41443700
C	1.01050900	0.61220600	-4.34537000
H	1.20913300	-0.41039500	-4.00152800
H	1.92519000	1.01074600	-4.80423100
H	0.23606500	0.56999000	-5.12400000
C	0.36171300	2.96457200	-3.61201100
H	-0.32169600	3.04857500	-4.46786700
H	1.32730500	3.38404400	-3.91975800
H	-0.02216000	3.57305400	-2.78786600
C	-1.91731800	1.04341200	-3.24390700
H	-1.66770800	1.54578400	-4.18589000
C	-2.94469400	1.91987400	-2.50977200
H	-3.29311000	1.42101700	-1.60001600
H	-3.81310800	2.10594000	-3.15609600
H	-2.49958200	2.88098500	-2.22873300
C	-2.48147100	-0.34262000	-3.60134700
H	-1.74104900	-0.92833800	-4.16009700
H	-3.38213300	-0.23701000	-4.22109600
H	-2.74947200	-0.89657600	-2.69619300
C	-4.29067600	-1.56060200	-0.20321100
H	-4.28014200	-0.80521100	-1.00482600
C	-3.57167500	-2.83470000	-0.70578300
H	-2.54276300	-2.60121900	-1.00773500
H	-3.50471800	-3.55792600	0.12325500
C	-4.32038500	-3.47733500	-1.88993600
H	-4.29517000	-2.78574300	-2.74676900
H	-3.79688600	-4.39214200	-2.20394600
C	-5.78495800	-3.78890400	-1.53721900
H	-6.30751500	-4.20494600	-2.41030800
H	-5.81298400	-4.55848400	-0.74894400
C	-6.50214300	-2.52540500	-1.03114500
H	-7.53611600	-2.76043700	-0.74078900
H	-6.55794800	-1.78561400	-1.84625200
C	-5.75316200	-1.90683800	0.16472500
H	-6.28674800	-1.01780900	0.52243400
H	-5.74264400	-2.62582000	0.99931200
C	-3.68684800	1.01296600	1.20235700
H	-3.04684100	1.37667500	0.38794900
C	-5.14518700	1.40551200	0.89429300
H	-5.46158900	0.97630700	-0.06621900
H	-5.81444800	0.99947200	1.67050600
C	-5.29912900	2.93913100	0.84352000
H	-4.69524000	3.32979600	0.00893000
H	-6.34659100	3.19900300	0.63212300
C	-4.83213100	3.59335700	2.15531600
H	-5.48686700	3.26127800	2.97774500
H	-4.92856400	4.68662500	2.09093300
C	-3.37857000	3.20103400	2.46838900
H	-2.71309900	3.60860300	1.69359600

H	-3.05793200	3.63829900	3.42468000
C	-3.20176800	1.67178500	2.51256100
H	-3.76567300	1.25390900	3.36226300
H	-2.13931000	1.44422700	2.66189000
C	4.25007800	-0.84936500	0.02469900
H	4.20690200	-0.11285700	0.84123600
C	4.38347100	-0.06700000	-1.29677000
H	3.51334800	0.58805400	-1.43914200
H	4.41020700	-0.76566300	-2.14690400
C	5.67413400	0.77655000	-1.29200000
H	5.77790200	1.30259400	-2.25164400
H	5.59016500	1.54827200	-0.51083300
C	6.91262600	-0.09579400	-1.01808200
H	7.04071200	-0.81347800	-1.84510400
H	7.81807000	0.52665000	-0.99316600
C	6.76325300	-0.87056600	0.30439900
H	6.71226900	-0.15501100	1.14055200
H	7.64249000	-1.50692700	0.47861700
C	5.48508300	-1.73282600	0.30692700
H	5.36540500	-2.23860900	1.27485100
H	5.57928900	-2.51771900	-0.45755100
C	2.22368600	-2.50271500	-1.45088900
H	2.16204000	-1.66690200	-2.16172700
C	3.30832200	-3.48591400	-1.94239100
H	4.28415400	-2.98925100	-2.00602500
H	3.41272000	-4.31300400	-1.22192800
C	2.93438600	-4.04769500	-3.33001500
H	2.92980400	-3.21856200	-4.05597700
H	3.70575100	-4.75742800	-3.66115500
C	1.55073300	-4.72208700	-3.32489700
H	1.29502200	-5.06974500	-4.33575900
H	1.58599400	-5.61402700	-2.67859000
C	0.47251900	-3.76131000	-2.79379200
H	-0.50230600	-4.26698000	-2.74108500
H	0.35600300	-2.91537200	-3.49002500
C	0.85517400	-3.22096800	-1.40483200
H	0.92399800	-4.06187100	-0.69569100
H	0.08045600	-2.55080900	-1.01904600
B	-0.49635200	0.24129700	-1.28440900
H	-1.02517300	3.88028900	0.18866500

References

(1) Frisch, M. J.; Trucks, G. W.; Schlegel, H. B.; Scuseria, G. E.; Robb, M. A.; Cheeseman, J. R.; Scalmani, G.; Barone, V.; Petersson, G. A.; Nakatsuji, H.; Li, X.; Caricato, M.; Marenich, A.; Bloino, J.; Janesko, B. G.; Gomperts, R.; Mennucci, B.; Hratchian, H. P.; Ortritz, J. V.; Izmaylov, A. F.; Sonnenberg, J. L.; Williams-Young, D.; Ding, F.; Lipparini, F.; Egidi, F.; Goings, J.; Peng, B.; Petrone, A.; Henderson, T.; Ranasinghe, D.; Zakrzewski, V. G.; Gao, J.; Rega, N.; Zheng, G.; Liang, W.; Hada, M.; Ehara, M.; Toyota, K.; Fukuda, R.; Hasegawa, J.; Ishida, M.; Nakajima, T.; Honda, Y.; Kitao, O.; Nakai, H.; Vreven, T.; Throsse, K.; Foresman, J. B.; Fox, D. J. Gaussian09 Revision D.01. Gaussian, Inc.: Wallingford CT 2016.

(2) Tao, J.; Perdew, J. P.; Staroverov, V. N.; Scuseria, G. E. Climbing the Density Functional Ladder: Nonempirical Meta–Generalized Gradient Approximation Designed for Molecules and Solids. *Phys. Rev. Lett.* **2003**, *91* (14), 146401. https://doi.org/10.1103/PhysRevLett.91.146401.

(3) Grimme, S.; Ehrlich, S.; Goerigk, L. Effect of the Damping Function in Dispersion Corrected Density Functional Theory. *J. Comput. Chem.* **2011**, *32* (7), 1456–1465. https://doi.org/10.1002/jcc.21759.

(4) Kozuch, S.; Shaik, S. How to Conceptualize Catalytic Cycles? The Energetic Span Model. *Acc. Chem. Res.* **2011**, *44* (2), 101–110. https://doi.org/10.1021/ar1000956.

(5) Marenich, A. V.; Cramer, C. J.; Truhlar, D. G. Universal Solvation Model Based on Solute Electron Density and on a Continuum Model of the Solvent Defined by the Bulk Dielectric Constant and Atomic Surface Tensions. *J. Phys. Chem. B* **2009**, *113* (18), 6378–6396. https://doi.org/10.1021/jp810292n.

(6) Jana, K.; Ganguly, B. DFT Studies on Quantum Mechanical Tunneling in Tautomerization of Three-Membered Rings. *Phys. Chem. Chem. Phys.* **2018**, *20* (44), 28049–28058. https://doi.org/10.1039/C8CP03963A.

(7) Kyriakou, G.; Davidson, E. R. M.; Peng, G.; Roling, L. T.; Singh, S.; Boucher, M. B.; Marcinkowski, M. D.; Mavrikakis, M.; Michaelides, A.; Sykes, E. C. H. Significant Quantum Effects in Hydrogen Activation. *ACS Nano* **2014**, *8* (5), 4827–4835. https://doi.org/10.1021/nn500703k.

(8) Parr, R. G.; Yang, W. Density Functional Approach to the Frontier-Electron Theory of Chemical Reactivity. *J. Am. Chem. Soc.* **1984**, *106* (14), 4049–4050. https://doi.org/10.1021/ja00326a036.

(9) Morell, C.; Grand, A.; Toro-Labbé, A. New Dual Descriptor for Chemical Reactivity. *J. Phys. Chem. A* **2005**, *109* (1), 205–212. https://doi.org/10.1021/jp046577a.

(10) Glendening, E. D.; Reed, A. E.; Carpenter, J. E.; Weinhold, F. NBO Version 3.1.
